# Galactosylsphingamides: new α-GalCer analogues to probe the F’-pocket of CD1d

**DOI:** 10.1038/s41598-017-04461-7

**Published:** 2017-06-27

**Authors:** Joren Guillaume, Jing Wang, Jonas Janssens, Soumya G. Remesh, Martijn D. P. Risseeuw, Tine Decruy, Mathy Froeyen, Dirk Elewaut, Dirk M. Zajonc, Serge Van Calenbergh

**Affiliations:** 1 Laboratory for Medicinal Chemistry (FFW), Faculty of Pharmaceutical Sciences, UGent, Ottergemsesteenweg 460, B-9000 Ghent, Belgium; 2 Division of Cell Biology, La Jolla Institute for Allergy and Immunology, 9420 Athena Circle, La Jolla, CA 92037 USA; 3 Department of Internal Medicine, Faculty of Medicine and Health Sciences, Ghent University, B-9000 Ghent, Belgium; 4 Unit Molecular Immunology and Inflammation, VIB Inflammation Research Center, Ghent University, Ghent, Belgium; 5 KU Leuven, Rega Institute for Medical Research, Laboratory of Virology and Chemotherapy, Herestraat 49, 3000 Leuven, Belgium

## Abstract

Invariant Natural Killer T-cells (*i*NKT-cells) are an attractive target for immune response modulation, as upon CD1d-mediated stimulation with KRN7000, a synthetic α-galactosylceramide, they produce a vast amount of cytokines. Here we present a synthesis that allows swift modification of the phytosphingosine side chain by amidation of an advanced methyl ester precursor. The resulting KRN7000 derivatives, termed α-galactosylsphingamides, were evaluated for their capacity to stimulate *i*NKT-cells. While introduction of the amide-motif in the phytosphingosine chain is tolerated for CD1d binding and TCR recognition, the studied α-galactosylsphingamides showed compromised antigenic properties.

## Introduction

Invariant natural killer T-cells (*i*NKT-cells) are a small set of glycolipid reactive T-cells that bridge innate and adaptive immunity. They display characteristics of both the NK cell lineage (mouse NK1.1, human CD161) and conventional thymus-derived T-cells^1^. Their semi-invariant T-cell receptor (TCR) is composed of an invariant Vα14-Jα18 chain in mice (homologous Vα24-Jα18 chain in humans), preferentially paired with Vβ8.2, Vβ7 or Vβ2 (Vβ11 in humans). Whereas conventional T-cells are activated by peptide antigens presented by MHC proteins, *i*NKT-cells typically recognize glycolipid antigens presented by MHC class 1 like CD1d molecules on antigen presenting cells^2^.

**Figure 1:**
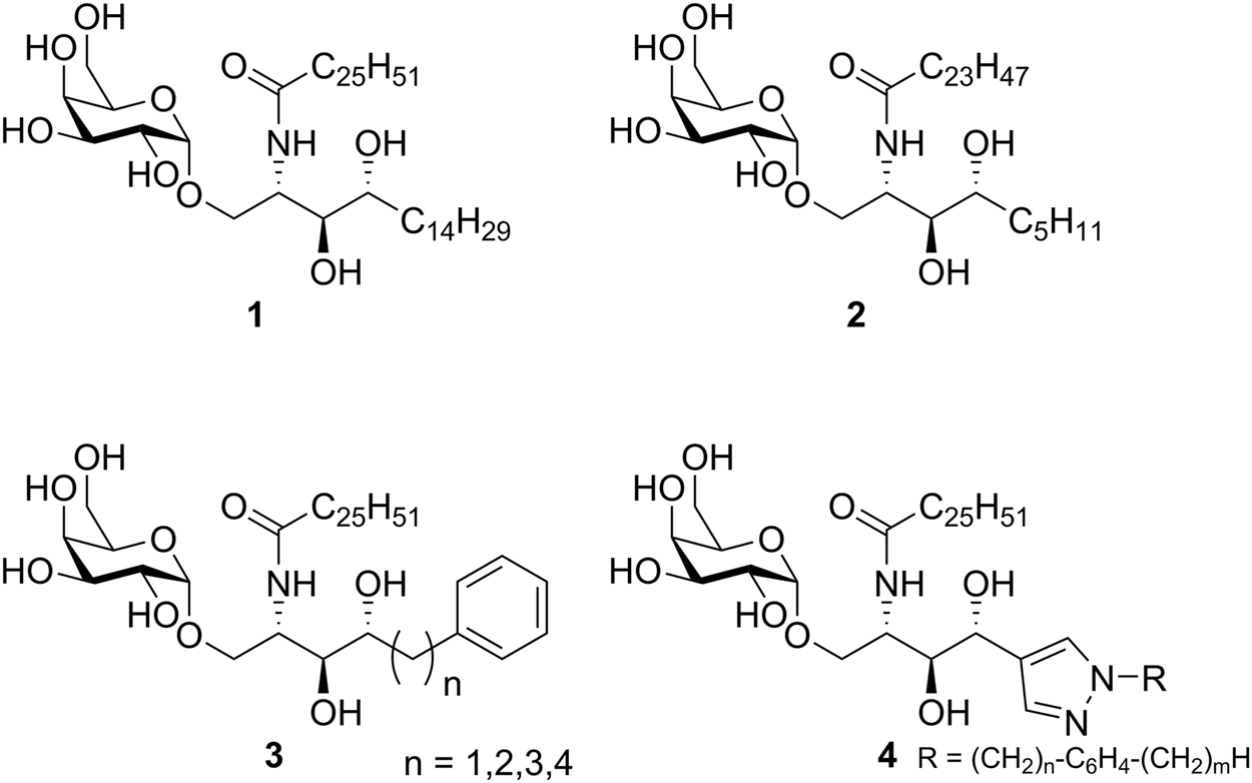
Structures of α-GalCer (**1**) and OCH (**2**) and known phytosphingosine-modified α-GalCer derivatives **3** and **4**.

The prototypical antigen for *i*NKT-cell stimulation is KRN7000, also known as α-GalCer (**1**), a synthetic glycolipid derived from agelasphins found in marine sponge extracts (Fig. 1)^3, 4^. Structural data illustrated that the lipid chains of α-GalCer are bound in two hydrophobic pockets deeply buried in CD1d^5, 6^. The larger A’-pocket binds the fatty acid part while the linear F’-pocket accommodates the phytosphingosine chain. Consequently, the galactose sugar is positioned at the surface of the CD1d binding groove and is available for recognition by the TCR of *i*NKT-cells^7^.

Upon stimulation, *i*NKT-cells rapidly secrete large amounts of pro-inflammatory Th1- (e.g., IFN-γ) and anti-inflammatory Th2-cytokines (e.g., IL-4). These cytokines mediate immune responses against tumors^8^, microbial infections^1, 9^ and auto-immunity^10^. In addition, *i*NKT-cells transactivate other immune cells including dendritic cells, B- and T-cells, macrophages and NK-cells and are thus able to orchestrate complex immune functions^1^. Despite the potent immune response triggered by α-GalCer, this antigen showed limited therapeutic efficacy in several clinical studies^11^. The lack of therapeutic effect is attributed to the antagonizing activities of the secreted pro-inflammatory Th1- and anti-inflammatory Th2-cytokines. Accordingly, the search for improved α-GalCer analogues capable of inducing a biased cytokine release is ongoing.

To date, the growing library of α-GalCer analogues mainly consists of glycolipids with an altered fatty acid chain^12, 13^ and/or carbohydrate part^14–17^. In addition, a vast amount of non-glycosidic analogues, including *C*-glycosides^18–20^ and cyclitol derivatives^21^ exist. However, analogues with modifications in the phytosphingosine chain remain relatively scarce, due to the more challenging syntheses required to access these analogs.

A common strategy for the synthesis of α-GalCer analogues modified in the phytosphingosine moiety relies on the Wittig olefination on L-Garner’s aldehyde, followed by stereoselective dihydroxylation of the Z-double bond furnishing the required d-ribo stereochemistry^14, 22^. Although this approach may afford high stereoselectivity, it inevitably leads to minor unwanted isomers thereby lowering the overall yield and forcing demanding purification steps ahead in the synthesis.

Hitherto, a small number of phytosphingosine modified analogues have been reported, some of which exhibit interesting biological properties. Most notably, OCH (**2**), a glycolipid with a truncated phytosphingosine chain and a slightly trimmed fatty acid residue, became a prototypical Th2-polarizing *i*NKT-cell ligand^23, 24^. Wong and coworkers reported derivatives with a terminal phenyl group in the sphingosine chain (**3**)^25^. Interestingly, the immunological properties of these analogues were strongly dependent on the relative position of the aromatic ring^12, 22, 26^. More recently, Park *et al*. demonstrated that selected derivatives bearing a pyrazole moiety in the phytosphingosine chain (**4**) give rise to a Th2-biased cytokine profile^27, 28^. These examples demonstrate that alterations of the sphingosine chain are tolerated and may afford analogues with interesting cytokine profiles.

Up to now, the mechanism of cytokine polarization is poorly understood, although there is ample of evidence suggesting that the stability of the CD1d/glycolipid/TCR-complex is an important contributing factor^29^. The potent Th1-response observed for α-GalCer derivatives featuring aromatic moieties in the acyl chain, is attributed to the formation of additional Van der Waals interactions with amino acid residues lining the A’-pocket^30^. Consequently these glycolipids exhibit an enhanced affinity for CD1d resulting in the observed cytokine bias. In analogy, the potent Th1-polarizing sugar-modified galactosylceramides previously synthesized in our lab, also feature a greater CD1d-affinity due to the formation of an extra binding pocket in CD1d via induced-fit^31^.

**Figure 2:**
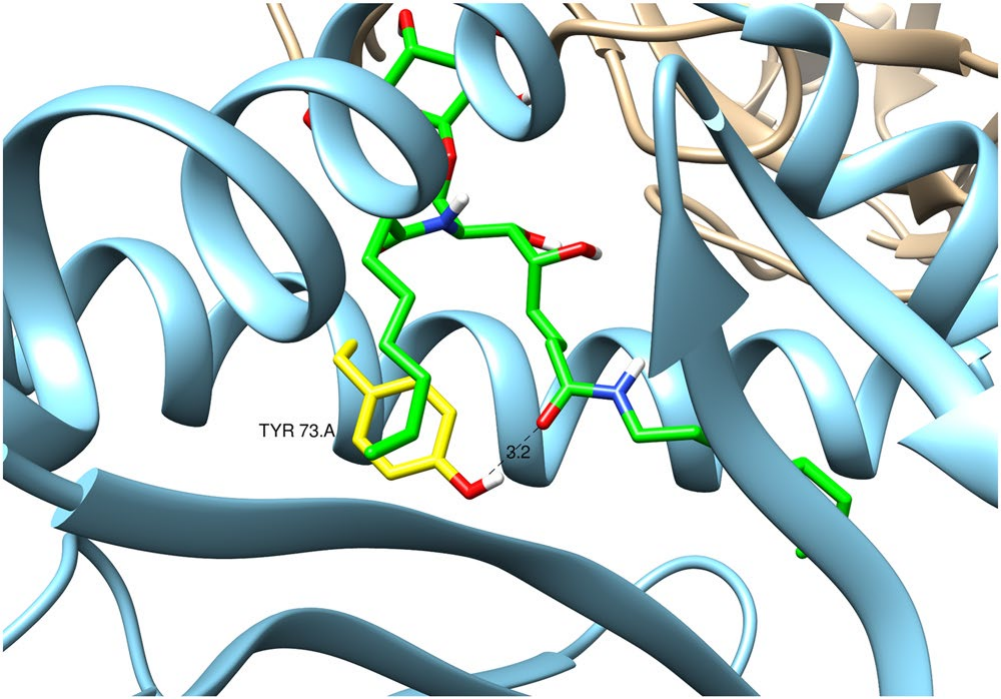
Model of the binding of **5a** to mouse CD1d. The **5a** carbons are depicted in green, while the Tyr73.A carbons are colored in yellow. The possible hydrogen bond with the amide oxygen atom is highlighted. Ribbons are colored as skyblue for the CD1d (chain A) and the mouse NKT TCR chains are colored in grey.

A study by McCarthy *et al*. indicates that although both the acyl chain and phytosphingosine contribute to the stability of the glycolipid/CD1d-complex, it is the phytosphingosine chain that controls TCR-binding and *i*NKT-cell activation, since ligand-induced conformational changes of the F’-pocket allosterically influence the CD1d/glycolipid footprint^32^. The aforementioned arguments excited us to explore a synthetic strategy for the preparation of phytosphingosine modified α-GalCer analogues, allowing us to introduce variations in the later stages of the synthesis via amide coupling. Molecular modeling studies indicate that the amide moiety, when placed at the right position, can potentially form a hydrogen bond with Tyr73 in the CD1d-binding groove and may consequently enhance the glycolipid/CD1d-complex stability (Fig. 2). The selected amide substituents are aimed at reinforcing the interactions (e.g., by π-π-stacking) with aromatic residues lining the F’-pocket.

To ensure acceptable solubility of the envisioned analogues, we opted to equip these initially with an octanoyl moiety, which was shown to retain most of the antigenic properties when replacing the *N*-hexacosanoyl group of α-GalCer^33^.

## Results and Discussion

### Chemistry

An overview of the synthesized α-galactosylsphingamides is shown in Table 1.

**Table 1:** Overview of the synthesized α-galactosylsphingamides.

R^1^	R^1^ = C_7_H_15_	R^1^ = C_11_H_23_	R^1^ = C_15_H_31_	R^1^ = C_19_H_39_	R^1^ = C_25_H_51_
R^2^					
C_9_H_19_	5a				
(CH_2_)_2_Ph	5b				
(CH_2_)_4_Ph	5c				
(CH_2_)_6_Ph	5d	6d	7d	8d	9d
(CH_2_)_8_Ph	5e				
Ph-*m*-C_5_H_11_	5f				9f
Ph-*p*-C_5_H_11_	5g				
((CH_2_)_2_O)_2_C_2_H_5_	5h				

**Figure 3:**
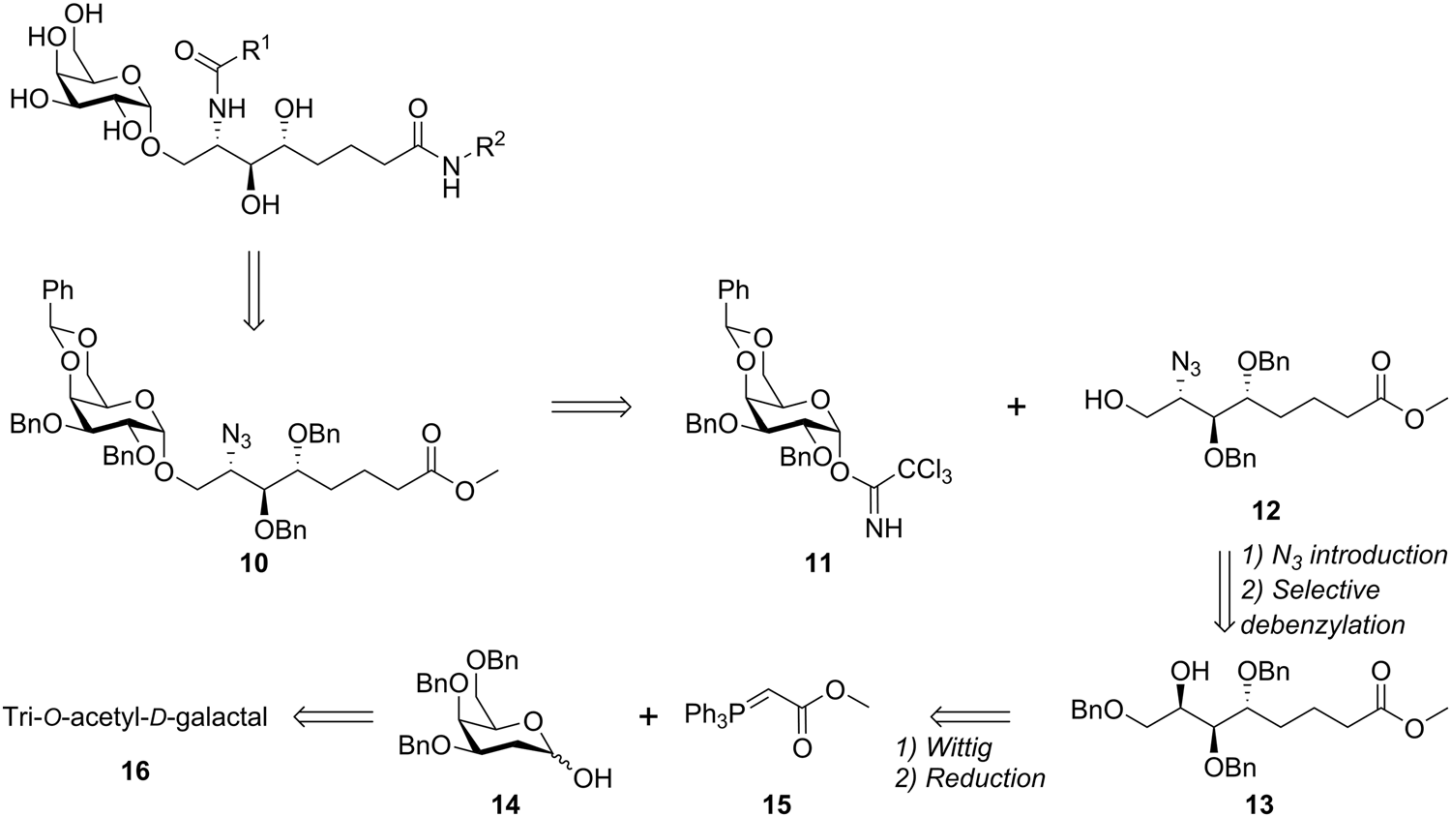
Retrosynthesis of the target α-galactosylsphingamides.

As shown in the retrosynthetic analysis (Fig. 3), the α-galactosylsphingamides (**5a-h**, **6**–**9d** and **9f**) can be accessed from methyl ester **10**, which is obtained through Lewis acid catalyzed glycosylation of glycosyl acceptor **12**. The latter would be obtained by conversion of alcohol **13** to an azide with an inversion of stereochemistry at the carbon centre, followed by selective benzyl ether cleavage to afford the primary alcohol. The 2-deoxygalactose intermediate **14**, prepared from commercially available tri-*O*-acetyl-d-galactal (**16**), would serve as a substrate for a Wittig olefination with phosphonium ylid **15**.

**Figure 4:**
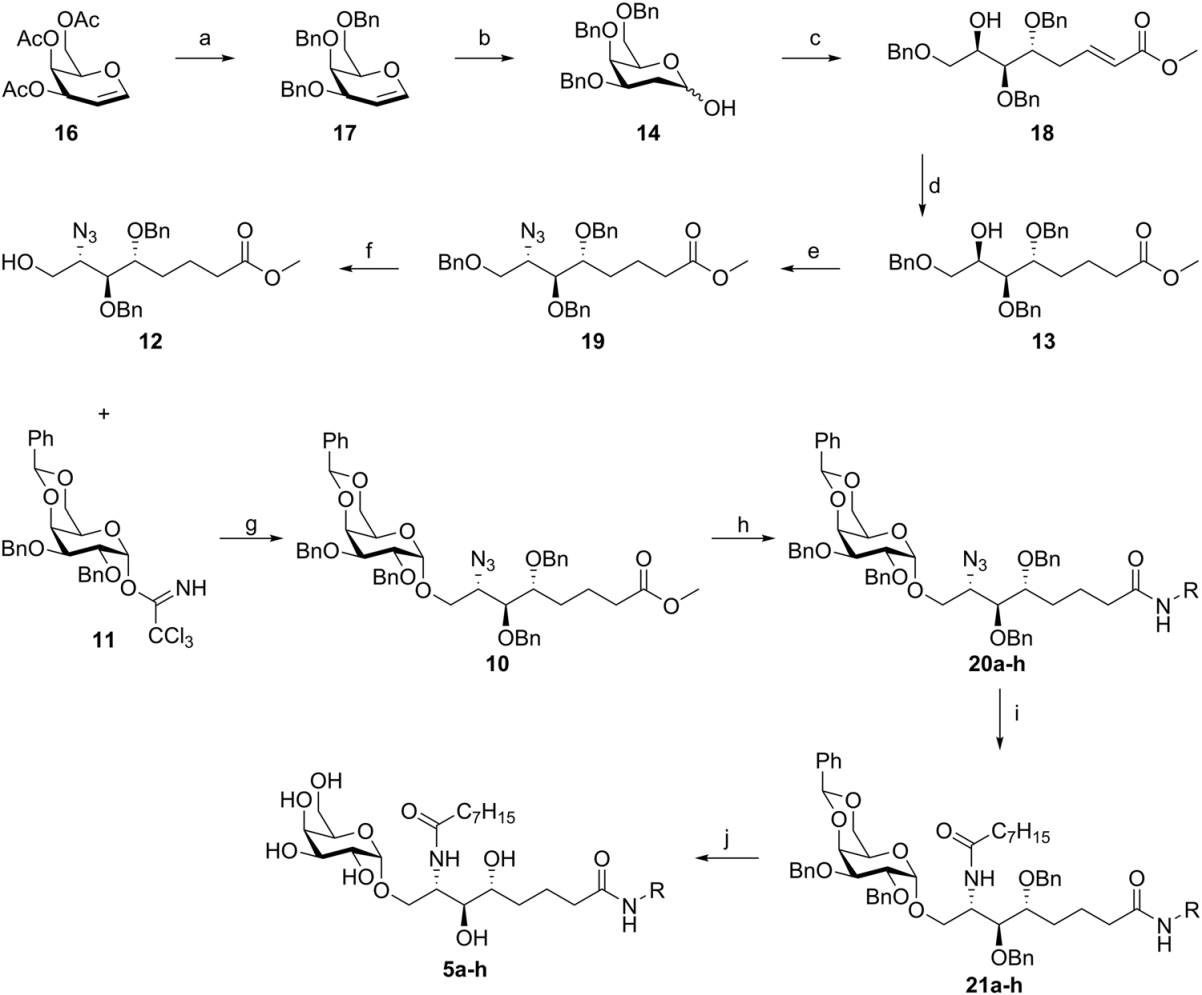
Reagents and conditions: (**a**) (i) Et_3_N, H_2_O, MeOH, RT, 4d; (ii) NaH, BnBr, DMF, RT, overnight, 94% (over 2 steps); (**b**) 4 m H_2_SO_4_, DMF, 0 °C → RT, overnight, 85%; (**c**) methyl (triphenylphosphoranylidene)acetate, toluene, 85 °C, 6 h, 79%; (**d**) NiCl_2_∙6 H_2_O, NaBH_4_, MeOH/THF, 0 °C, 1 h, 94%; (**e**) PPh_3_, diethylazodicarboxylate, diphenylphosphorylazide, THF, −20 °C → RT, 7 h, 95%; (**f**) (i) ZnCl_2_, AcOH/Ac_2_O, 3.5 h, RT; (ii) MeOH, NaOMe, pH 10, RT, overnight, 67% over 2 steps; (**g**) TMSOTf, THF, −30 °C, 2 h, 67%; (**h**) AlMe_3_, amine, CH_2_Cl_2_, reflux, overnight, 56%-85%; (**i**) (i) PMe_3_, H_2_O, THF, RT, 7 h; (ii) EDC, octanoic acid, CH_2_Cl_2_, RT, overnight, 51%–69% over 2 steps; (**j**) H_2_, Pd black, MeOH, CHCl_3_, RT, overnight, 37%–58%.

The synthetic route towards glycosyl acceptor **12** is outlined in Fig. 4. It was decided to start from commercially available tri-*O*-acetyl-d-galactal (**16**), which was readily converted into the 3,4,6-tri-*O*-benzyl-protected derivative **17** ^34^. Hydrolysis of the enol ether was achieved upon treatment with 4 m sulfuric acid, furnishing 2-deoxygalactose intermediate **14** ^35^. Next, the Wittig reaction on **14** with methyl(triphenylphosphoranylidene)acetate was investigated. When the reaction was carried out in THF, α,β-unsaturated ester **18** was obtained exclusively as the *E*-isomer in moderate yield (64%). Performing the reaction in refluxing toluene increased the yield to 79%, while also providing solely the *E*-isomer. To avoid Michael type side reactions and to eliminate the possibility for a 1,3-dipolar cycloaddition upon introduction of the azide, saturation of the double bond was accomplished by conjugate reduction with nickel(II) chloride and sodium borohydride. Mitsunobu reaction with diphenyl phosphoryl azide (DPPA) converts the C2-OH to an azido group with the appropriate stereochemistry (**19**). Next, selective deprotection of the primary hydroxyl was achieved by treatment of **19** with zinc chloride in acetic anhydride followed by Zemplén deacetylation of the intermediate acetate to furnish acceptor **12**.

Next, TMSOTf-promoted glycosylation of acceptor **12** with galactosyl trichloroacetimidate **11** ^36^ afforded α-galactoside **10** in good yield and without notable formation of the β-glycoside. The ability to diversify the methyl ester after glycosylation is convenient as it reduces the number of linear steps towards the target α-GalCer analogues. A Lewis acid-catalyzed amidation with the appropriate amines gave intermediates **20a-h**. Next, the azido group was subjected to Staudinger reduction with trimethylphosphine and the resulting amine was coupled with octanoic acid using EDC. Finally, catalytic hydrogenolysis afforded the desired compounds **5a-h**. Analogues **6-9d** and **9f** with alternative acyl moieties were synthesized according to similar procedures.

### Biological evaluation

**Figure 5:**
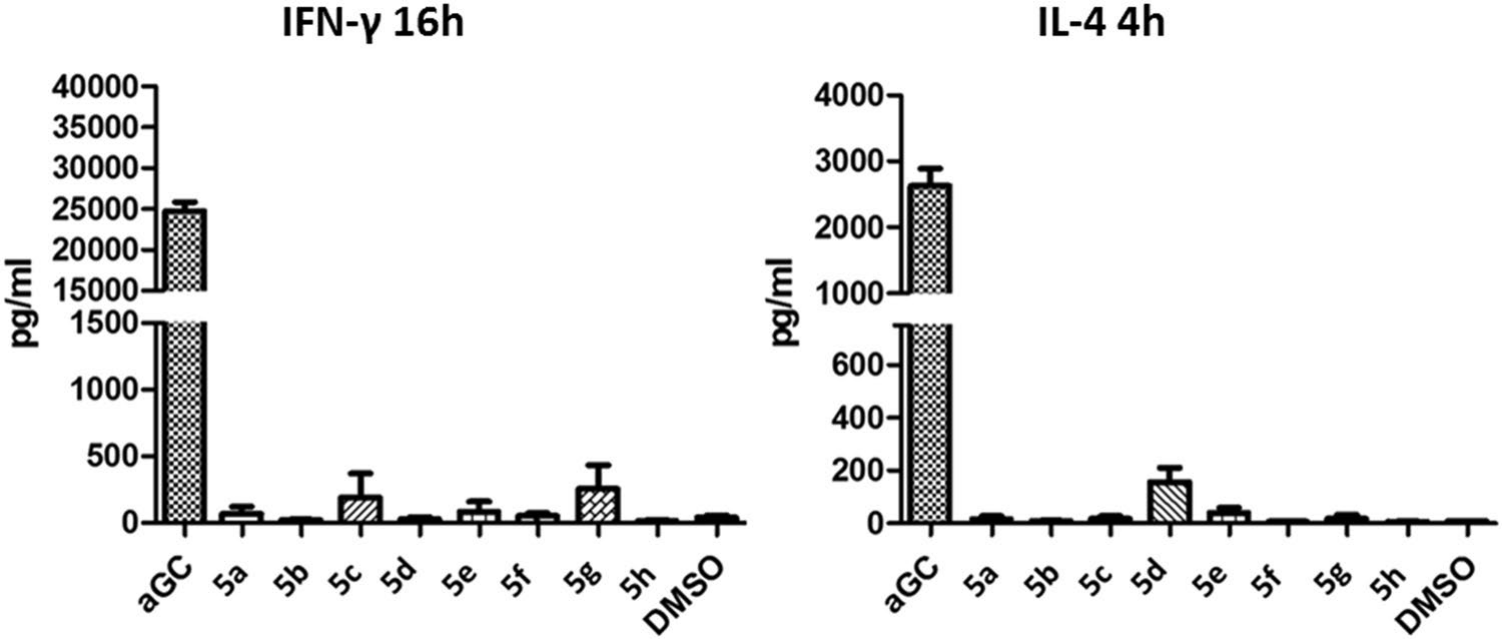
IL-4 and IFN-γ secretion, measured at respectively 4 h and 16 h, after intraperitoneal injection of 5 µg of the glycolipids in mice. Data for one individual experiment using 8 mice for each glycolipid.

To assess the biological activity of the α-galactosylsphingamides, we analyzed the IFN-γ- and IL-4-levels after intraperitoneal injection of 5 µg of the galactosylsphingamides **5a-h** in mice (Fig. 5).

The marginal cytokine release induced by this series indicates that the introduction of an amide moiety in the phytosphingosine chain compromises the antigenic potency.

**Figure 6:**
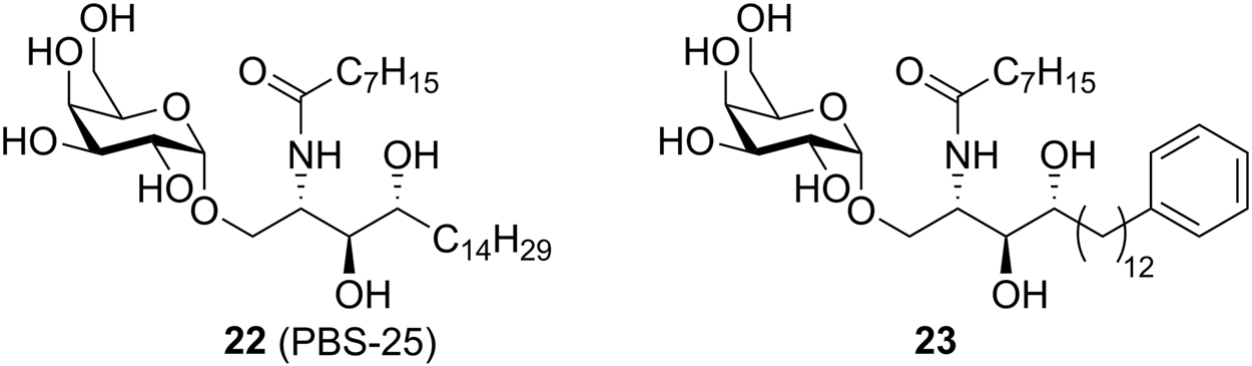
Structures of reference compounds PBS-25 (**22**) and **23**.

**Figure 7:**
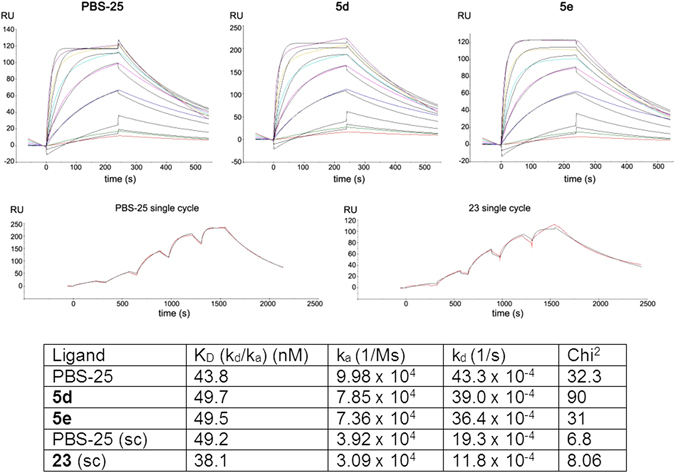
Real-time TCR-binding kinetics to mCD1d-presented ligands. Each curve represents the TCR binding sensorgram to CD1d-glycolipid complexes at a different TCR concentration after reference substraction (binding response to CD1d without added glycolipid). Sensorgrams (top) indicate similar lipid loading levels for **5d-e**. Single cycle TCR kinetics were measured for PBS-25 and 23. Here, TCR with increasing concentrations (3-fold) was sequentially added to CD1d-glycolipid with a final dissociation step of 10 min. Colored curves represent actual binding responses and black curves the fitted data. Kinetic values derived from the sensorgrams are listed below.

In an effort to explain the poor antigenicity of **5a-h**, we next tested the binding of the Vα14Vβ8.2 TCR of the murine iNKT-cell hybridoma 2C12 to mCD1d-presenting ligands **5d,e** using surface plasmon resonance (SPR) and compared it with the binding of PBS-25^33^, an α-GalCer analogue that also features an octanoic acid residue. It should be noted that in our hands, PBS-25 was inactive in the *in vivo* cytokine secretion assay (Supporting Figure S1). To further evaluate whether the low antigenicity of **5d** is resulting from the amide functionality in the phytosphingosine chain, we synthesized glycolipid **23** (Fig. 6), which is the amide-deleted counterpart of α-galactosphingamides **5d** and **5e**. Surprisingly, all ligands were bound with similarly high TCR affinities (K_D_ = 38–50 nM) (Fig. 7). Therefore, the TCR binding kinetics cannot explain the inability of the galactosylsphingamides to induce robust cytokine production *in vivo*. In addition, the maximal response (RU 120–220) observed for the individual sensorgrams of the α-galactosylsphingamides are comparable between the individual CD1d-ligand complexes, which were immobilized at similar levels, suggesting a similar CD1d ligand binding efficiency *in vitro*.

**Figure 8:**
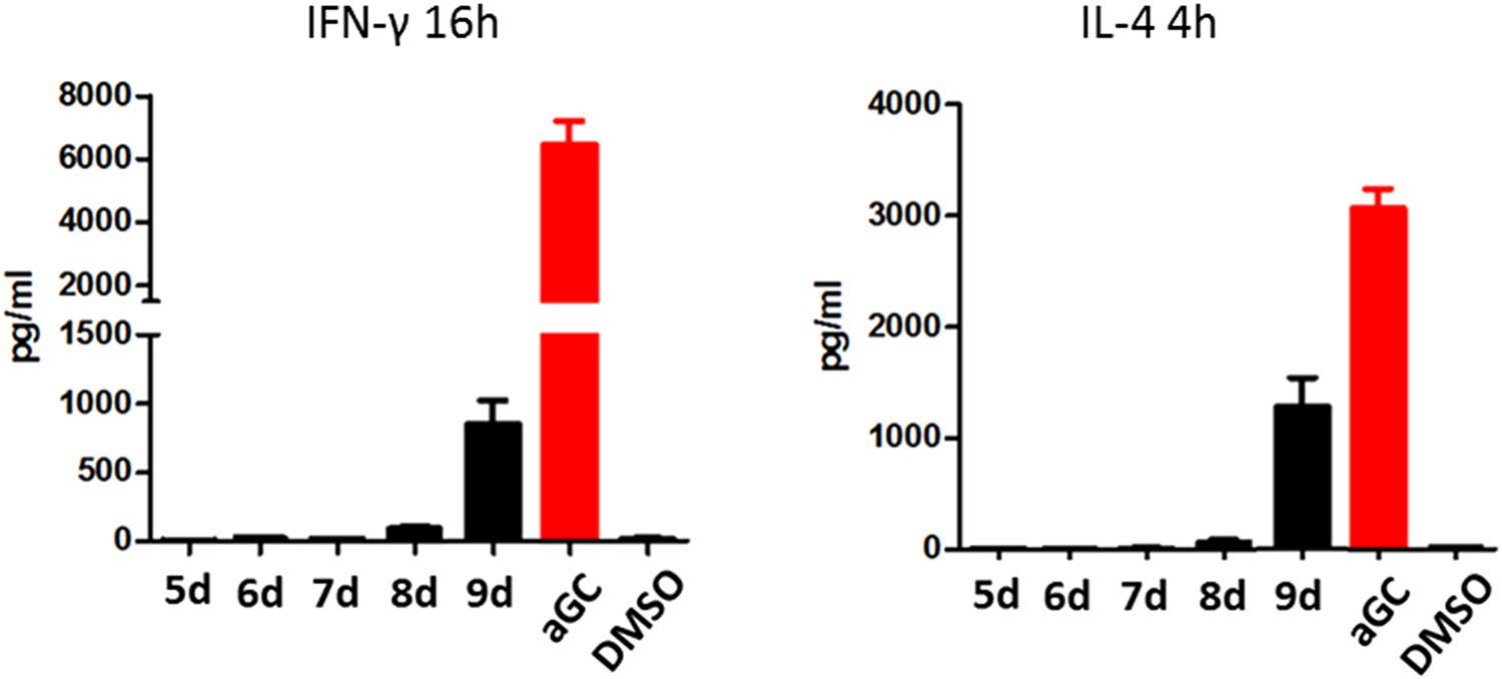
IFN-γ and IL-4 secretion, measured at 16 h and 4 h respectively, after intraperitoneal injection of 5 µg of the glycolipids in mice. Data for one individual experiment using 8 mice for each glycolipid.

Since the current galactosylsphingamides are based on a C8 fatty acid, the short acyl chain will not fully occupy the A’ pocket of CD1d. Likely similar to PBS-25^37^, free fatty acid will be recruited to fill the remainder of the pocket. To assess whether these spacer lipids can potentially influence the ligand potency, we synthesized ligands based on **5d** that contained longer acyl chains of 12, 16, 20, and 26 carbon atoms (**6d-9d**). Since the acyl chain is introduced at the final stages of the synthesis, the elongated fatty acid chains could easily be introduced, demonstrating the versatility of our synthetic approach. We next analyzed antigenicity by measuring IFN-γ- and IL-4-levels after intraperitoneal injection of 5 µg of the acyl-elongated galactosylsphingamides **6-9d** in mice (Fig. 8). Surprisingly, cytokine levels are still inferior to those observed for α-GalCer but increase slightly upon elongating the acyl chain. Overall, the introduction of an amide functionality in the phytosphingosine chain appears to result in poor *i*NKT-cell stimulation. The recruitment of spacer lipids occupying the A’ pocket was observed for **5a-d**, but the spacer lipid had no impact on the antigenic potency of the galactosylsphingamides since the antigenic potency of **8d** and **9d**, which have no space to fit a spacer lipid, also perform poorly.

### Structural data

**Figure 9:**
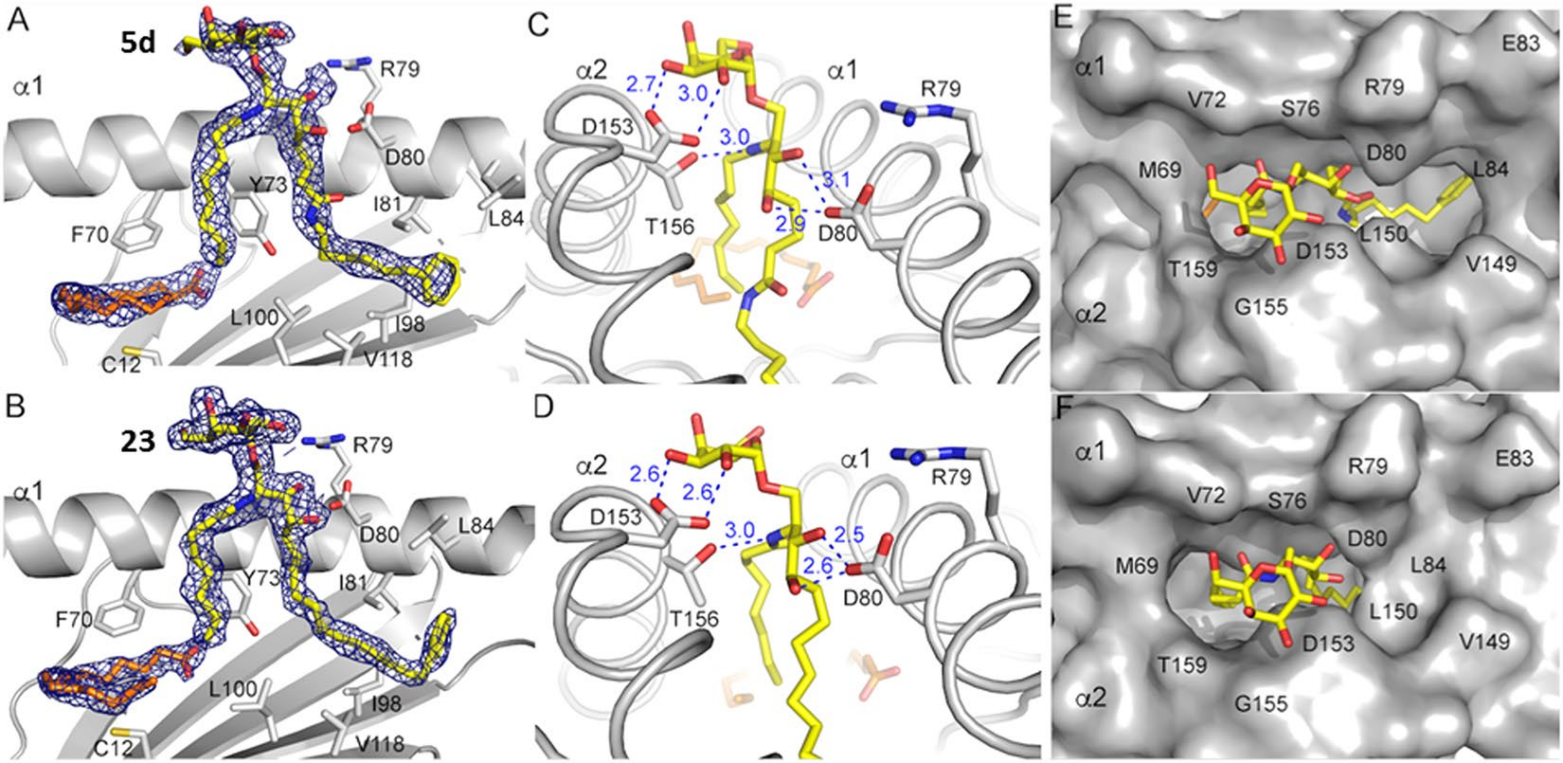
(**A**,**B**) Electron density map of **5d** and **23** with CD1d. Spacer lipid (palmitic acid) is depicted in orange. (**C**,**D**) H-bond interactions between **5d** and **23** (yellow) with CD1d (grey). (**E**,**F**) Glycolipid ligand presentation shown in “TCR view” from top with molecular surface of CD1d in grey and ligands as yellow sticks.

To gain insights into the poor antigenic potency of the glycolipids but high affinity TCR binding, we determined the crystal structure of the CD1d/**5d**-complex as well as that of the complex of CD1d with compound **23**, which resembles **5d** but lacks the amide group. As expected, **23** gives rise to well-defined electron density and is rigidly presented using the conserved H-bond network involving CD1d-residues Asp80 and the 3’- and 4’-OH of phytosphingosine, as well as CD1d Thr156 and the glycosidic oxygen, and most importantly, CD1d-residue Asp153 and the 2”-, and 3”-OH of the galactose (Fig. 9A–D). Surprisingly, galactosylsphingamide **5d** is presented less well ordered by CD1d, as judged by the less contoured electron density (Fig. 9A,B). This slight disorder of the galactose presentation correlates well with the increase in the H-bond distance between CD1d and **5d**. Especially the 2”-OH interaction with CD1d Asp153 is increased compared to **23** (3.0 vs. 2.6 Å, Fig. 9C,D), while the 3’-OH of phytosphingosine is also less intimately contacted by CD1d Asp80 (3.1 vs. 2.5 Å). Notewhorthy, the computationally modelled hydrogen bond between the amide oxygen and Tyr73 was not observed in the glycolipid/CD1d crystal structure. When looking down into the binding groove of CD1d (TCR view), we noticed lack of the F’-roof closure by **5d**, while **23** induced the F’-roof formation (Fig. 9E,F). This is likely the result of the amide group perturbing the hydrophobic nature of the F’-pocket in general. Based on our previous work on microbial glycolipids, we postulated that the TCR-dissociation rate is increased for glycolipids that do not close the F’-roof before TCR-binding^38^. However, since we have not observed any differences in the binding kinetics, especially the dissociation rate, we speculate that a compensatory mechanism will stabilize the TCR-interaction and this could likely be driven by the amide group and a novel interaction within the F’-pocket of CD1d. This will have to be investigated in the future using TCR-co-crystal structures.

**Figure 10:**
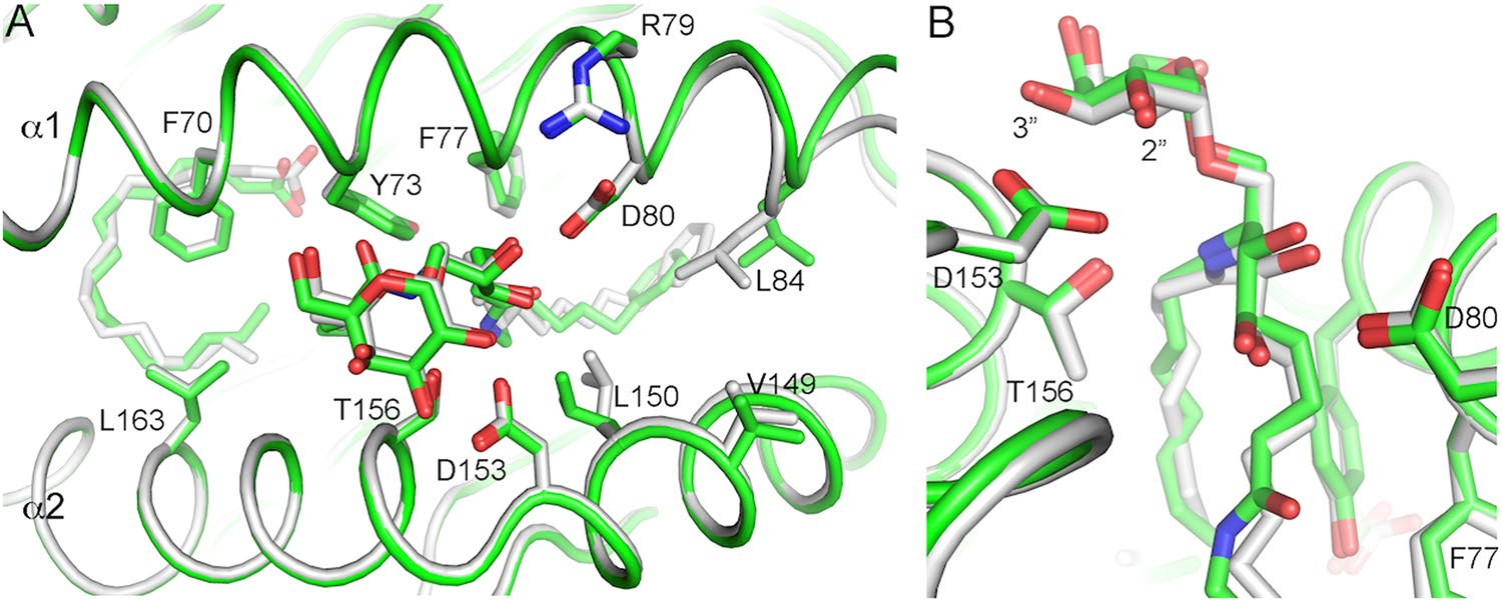
Comparison between **5d** (green) and **23** (grey)presented by CD1d.

We next compared the precise presentation of both ligand headgroups by CD1d. As suggested by the changes in hydrogen bond distances, the galactose of **5d** is slightly elevated compared to **23**, correlating with the increased distance in H-bonding interactions with CD1d (Fig. 10).

This slightly flexible presentation of **5d** is reminiscent to the presentation of the microbial glycosphingolipid GalA-GSL, which was also slightly shifted in the binding groove of CD1d compared to **22** but had comparable TCR association rates^39^. Therefore, the fine presentation of the galactose is likely not the reason for the biological inactivity of these galactosylsphingamides.

Since the only difference between both glycolipids is the presence (**5d**) or absence (**23**) of the amide group, this indicates that the amide is indeed responsible for the slight elevation of the galactose but more importantly for the failure in closure of the F’ roof in the binary complex, the latter of which is likely the main reason for the low antigenicity of the α-galactosylsphingamides (Fig. 10).

## Conclusion

In summary, a series of α-GalCer analogues featuring an amide bond in the phytosphingosine were synthesized. The presented synthesis allows to alter an advanced methyl ester precursor after glycosylation, thereby reducing the number of linear steps towards the aimed α-GalCer analogues. Furthermore, the ability to diversify the acyl moiety in the penultimate step of the synthesis adds to the versatility of the presented synthesis route. Also, methyl ester **10** is an interesting intermediate for alternative derivatization of the phytosphingosine chain.

Biological evaluation revealed that the novel α-galactosylsphingamides are weak *i*NKT-cell antigens and that the main compromising factor is the implemented amide functionality. While extending the acyl chain offered partial restoration of the antigenic effect, overall *i*NKT-cell stimulation remained poor.

Crystal structure analysis revealed a slightly flexible presentation of the α-galactosylsphingamides to CD1d, where the sugar head group is slightly shifted in the CD1d binding groove. A similar binding has been observed for the microbial glycosphingolipid GalA-GSL, but never for known α-GalCer analogues^39^. These crystal structures support the hypothesis that although both the acyl chain and phytosphingosine contribute to the stability of the CD1d/glycolipid complex, it is the phytosphingosine chain that controls the CD1d/glycolipid footprint. How the amide group affects the biological activity of these compounds and how its presence compensates for the lack of the pre-formed F’ roof formation upon TCR binding is currently unknown and will have to be characterized by determining CD1d-galactosylsphingamide-TCR structures.

### Experimental part

#### General

Precoated Macherey-Nagel SIL G/UV254 plates were used for TLC, and spots were examined under UV light at 254 nm and further visualized by sulfuric acid-anisaldehyde spray or by spraying with a solution of (NH_4_)_6_Mo_7_O_24_∙4 H_2_O (25 g/L) and (NH_4_)_4_Ce(SO_4_)_4_∙2 H_2_O (10 g/L) in H_2_SO_4_ (10%) followed by charring. Column chromatography was performed on Biosolve silica gel (32–63 µm, 60 Å). NMR spectra were obtained with a Varian Mercury 300 Spectrometer. Chemical shifts are given in ppm (δ) relative to the residual solvent signals, in the case of CDCl_3_: δ = 7.26 ppm for ^1^H and δ = 77.4 ppm for ^13^C and in the case of pyridine-d_5_: δ = 8.74, 7.58 and 7.22 ppm for ^1^H and δ = 149.9, 135.5 and 123.5 ppm for ^13^C. Exact mass measurements were performed on a Waters LCT Premier XE TOF equipped with an electrospray ionization interface and coupled to a Waters Alliance HPLC system. Samples were infused in a in a CH_3_CN/HCOOH (1000:1) mixture at 100 µL/min.

#### 3,4,6-Tri-O-benzyl-D-galactal (**17**)

3,4,6-Tri-*O*-acetyl-d-galactal (22.05 g, 81 mmol) was dissolved in MeOH (250 mL), Et_3_N (67 mL) and H_2_O (8 mL) and the reaction mixture was stirred for 5 days at room temperature. After completion of the reaction the solvent was removed under reduced pressure. The crude product was dried by making azeotropic mixture with toluene to afford pure d-galactal in quantitative yield. This material was dissolved in dry DMF (250 mL) and cooled to 0 °C. Sodium hydride (60% dispersion, 13 g, 324 mmol) was added portionwise over a period of 15 min. After 30 minutes benzyl bromide (38.8 mL, 324 mmol) was added dropwise and the reaction mixture was stirred overnight, allowing the temperature to rise to room temperature. Upon completion of the reaction, the reaction mixture was quenched with MeOH at 0 °C. The mixture was extracted with EtOAc (4 × 200 mL) and the combined organic layers were washed with brine, dried over anhydrous Na_2_SO_4_ and the solvents were evaporated under reduced pressure. The crude was purified by silica gel chromatography (hexane/EtOAc: 9/1) furnishing 3,4,6-tri-*O*-benzyl-d-galactal (31.67 g, 94%) as a white solid.

^1^H-NMR (300 MHz, CDCl_3_): δ 3.65 (dd, *J* = 10.1 and 5.1 Hz, 1 H, Ha-6) 3.78 (dd, *J* = 10.1 and 7.2 Hz, 1 H, Hb-6) 3.92–3.96 (m, 1 H, H-4) 4.15–4.21 (m, 2 H, H-3 and H-5) 4.41 (d, *J* = 12.2, 1 H, CH_2_Ph) 4.50 (d, *J* = 12.2, 1 H, CH_2_Ph) 4.57–4.68 (m, 3 H, H-2, CH_2_Ph) 4.82–4.90 (m, 2 H, H-2, CH_2_Ph) 6.36 (d, *J* = 6.4 Hz, 1 H, H-1) 7.24–7.36 (m, 15 H, arom. H).

^13^C-NMR (75 MHz, CDCl_3_): δ 68.38, 70.70, 70.82, 71.21, 73.26, 73.35, 75.63, 99.90, 127.37, 127.48, 127.62, 127.83, 128.08, 128.24, 128.32, 137.94, 138.30, 138.44, 144.12.

Exact mass (ESI-MS) for C_27_H_28_NaO_4_ [M + Na]^+^ found, 439.1878; calcd, 439.1880.

#### 2-Deoxy-3,4,6-tri-O-benzyl-D-galactose (**14**)

To a solution of 3,4,6-tri-*O*-benzyl-d-galactal (31.7 g, 76 mmol) in DMF (250 mL) maintained at 0 °C, H_2_SO_4_ (80 mL, 4.0 M) was added dropwise. The reaction mixture was allowed to rise to room temperature and was stirred for 16 h. Upon completion of the reaction, the mixture was neutralized with NaHCO_3_ (400 mL, sat. aq.) and extracted with Et_2_O (3 × 200 mL). The combined organic layers were washed with brine, dried over MgSO_4_, filtered and evaporated. The crude residue was purified by silica gel chromatography (hexane/EtOAc: 85/15) furnishing **14** (28.13 g, 85%) as a white solid.

^1^H-NMR (α-anomer) (300 MHz, CDCl_3_): δ 1.90–2.09 (m, 1 H, Ha-2) 2.23 (td, *J* = 12.4, 3.6 Hz, 1 H, Hb-2) 3.41–3.73 (m, 2 H, CH_2_-6) 3.73–3.92 (m, 1 H, H-5) 4.00 (ddd, *J* = 12.0, 4.6 and 2.5 Hz, 1 H, H-3) 4.14 (t, *J* = 6.5 Hz, 1 H, H-4) 4.44 (d, *J* = 12.0 Hz, 1 H, CH_2_Ph) 4.52 (d, *J* = 12.0 Hz, 1 H, CH_2_Ph) 4.59–4.66 (m, 3 H, CH_2_Ph) 4.94 (d, *J* = 11.8 Hz, 1 H, CH_2_Ph) 5.47 (d, *J* = 3.1 Hz, 1 H, H-1) 7.20–7.43 (m, 15 H, arom. H).

^13^C-NMR (75 MHz, CDCl_3_): δ 30.92, 34.24, 69.0, 69.79, 70.08, 70.31, 71.65, 72.97, 73.29, 73.99, 74.19, 92.39, 94.54, 127.16, 127.40, 127.44, 127.53, 127.61, 128.02, 128.08, 128.22, 128.27, 137.67, 138.36, 138.45.

Exact mass (ESI-MS) for C_27_H_30_NaO_5_ [M + Na]^+^ found, 457.1987; calcd, 457.1985.

Data consistent with literature^40^.

#### (5R,6S,7R,E)-methyl 5,6,8- tri-O-benzyl-7-hydroxyoct-2-enoate (**18**)

2-Deoxy-3,4,6-tri-*O*-benzyl-d-galactose **14** (3.7 g, 8.52 mmol) and methyl(triphenylphosphoranylidene)acetate (5.68 g, 17.04 mmol) were dissolved in anhydrous toluene (150 mL) and stirred for 6 h at 85 °C. The reaction mixture was evaporated under reduced pressure, and the residue was purified by silica gel chromatography (hexane/EtOAc: 8/2) to give **18** (3.29 g, 79%) as a slightly yellow oil.

^1^H-NMR (300 MHz, CDCl_3_): δ 2.58 (ddd, *J* = 7.2, 5.7 and 1.3 Hz, 2 H, H-4) 3.48–3.60 (m, 2 H, H-8) 3.62–3.70 (m, 1 H, H-6) 3.73 (s, 3 H, OCH_3_) 3.76–3.84 (m, 1 H, H-5) 3.96–4.05 (m, 1 H, H-7) 4.49–4.52 (m, 2 H, CH_2_Ph) 4.55–4.57 (m, 3 H, CH_2_Ph) 4.67 (d, *J* = 11.4 Hz, 1 H, CH_2_Ph) 5.88 (dt, *J* = 15.7 and 1.3 Hz, 1 H, H-2) 7.01 (dt, *J* = 15.7 and 7.4 Hz, 1 H, H-3) 7.23–7.40 (m, 15 H, arom. H).

^13^C NMR (75 MHz, CDCl_3_) δ 33.81, 51.44, 69.66, 71.08, 72.56, 73.44, 74.06,78.48, 79.00, 123.21, 127.77, 127.86, 127.90, 127.94, 127.99, 128.07, 128.42, 128.45, 137.68, 137.75, 137.87, 145.57, 166.64.

Exact mass (ESI-MS) for C_30_H_35_O_6_ [M + H]^+^ found, 491.2427; Calcd., 491.2428.

#### (5R,6S,7R)-methyl 5,6,8- tri-O-benzyl -7-hydroxyoctanoate (**13**)

NiCl_2_.6 H_2_O (800 mg, 3.36 mmol) and NaBH_4_ (508 mg, 13.42 mmol) were added to a stirred solution of unsaturated ester **18** (3.29 g, 6.71 mmol) in MeOH (37 mL) and THF (3.7 mL) at 0 °C. After 1 h, the reaction mixture was filtered through Celite and the filter cake rinsed with Et_2_O. Evaporation of the filtrate yielded **13** (3.09 g, 94% yield) as a yellow oil which could be used in the next step without further purification.

^1^H-NMR (300 MHz CDCl_3_) δ 1.52–1.88 (m, 4 H, H-2 and H-4) 2.16–2.50 (m, 2 H, H-3) 3.01 (br. s, 1 H, -OH) 3.55 (d, *J* = 5.8 Hz, 2 H, H-8) 3.60–3.73 (m, 5 H, OCH_3_, H-5 and H-6) 3.98–4.05 (m, 1 H, H-7) 4.51–4.54 (m, 3 H, CH_2_Ph) 4.56–4.58 (m, 1 H, CH_2_Ph) 4.66 (d, *J* = 11.4 Hz, 1 H, CH_2_Ph) 4.72 (d, *J* = 11.4 Hz, 1 H, CH_2_Ph) 7.25–7.41 (m, 15 H, arom. H).

^13^C NMR (75 MHz, CDCl_3_) δ 20.99, 30.27, 33.94, 51.48, 69.86, 71.06, 72.71, 73.42, 73.78, 78.97, 79.41, 127.71, 127.74, 127.85, 127.89, 127.96, 128.09, 128.38, 128.41, 138.03, 138.08, 173.83.

Exact mass (ESI-MS) for C_30_H_36_KO_6_ [M + K]^+^ found, 531.2151; Calcd., 531.2143.

#### (5R,6S,7S)-methyl 7-azido-5,6,8- tri-O-benzyl-octanoate (**19**)

To a solution of compound **13** (3.09 g, 6.27 mmol) in anhydrous THF (100 mL) were added PPh_3_ (3.29 g, 12.54 mmol), diethylazodicarboxylate (2.18 g, 5.7 mL, 12.54 mmol), and diphenylphosphorylazide (3.45 g, 2.71 mL, 12.54 mmol) at −20 °C. After addition, the reaction mixture was allowed to warm to room temperature and stirred for 7 h. The solvent was evaporated under reduced pressure to give an orange residue. Purification by silica gel chromatography (hexane/EtOAc: 9/1) afforded **19** (3.19 g, 95% yield) as a yellow oil.

^1^H-NMR (300 MHz CDCl_3_) δ 1.52–1.86 (m, 4 H, H-2 and H-4) 2.18–2.39 (m, 2 H, H-3) 3.55–3.83 (m, 8 H, OCH_3_, H-5, H-6, H-7 and H-8) 4.57–4.62 (m, 3 H, CH_2_Ph) 4.65 (d, *J* = 11.5 Hz, 1 H, CH_2_Ph) 4.68 (d, *J* = 11.3 Hz, 1 H, CH_2_Ph) 4.78 (d, *J* = 11.3 Hz, 1 H, CH_2_Ph) 7.18–7.46 (m, 15 H, arom. H).

^13^C NMR (75 MHz, CDCl_3_) δ 20.72, 29.07, 33.94, 51.47, 61.93, 70.08, 71.95, 73.34, 73.83, 78.65, 78.74, 120.18, 120.24, 126.09, 127.66, 127.69, 127.71, 127.77, 127.91, 128.00, 128.37, 128.38, 128.41, 130.03, 130.05, 137.81, 137.91, 138.09, 173.84.

Exact mass (ESI-MS) for C_30_H_35_KN_3_O_5_ [M + K]^+^ found, 556.2210; Calcd., 556.2208.

#### (5R,6S,7S)-methyl 7-azido-5,6-di-O-benzyl-8-hydroxyoctanoate (**12**)

Azide **19** (1.4 g, 2.7 mmol) and ZnCl_2_ (7.38 g, 54 mmol) were dissolved in Ac_2_O/AcOH (2:1, 21 mL). The reaction was allowed to stir for 3.5 h at room temperature. Next H_2_O (10 mL) was added and the reaction mixture was extracted with CH_2_Cl_2_. The combined organic layers were washed with H_2_O, NaHCO_3_ (sat. aq.) and brine. Then the organic phase was dried over Na_2_SO_4_, filtered, and evaporated to afford 598 mg of crude acetate. The crude acetate was dissolved in MeOH (20 mL) and NaOMe (5.4 M solution in MeOH) was added till pH 10. After stirring overnight, Amberlite 120R (H^+^ form) was added to neutralize the reaction mixture. The mixture was diluted with MeOH (20 mL) and the exchange resin was filtered off and rinsed with MeOH. Evaporation of the filtrate under reduced pressure yielded a solid residue that was purified by silica gel chromatography (hexane/EtOAc: 7/3) to afford **12** (771 mg, 67% yield) as a colorless oil.

^1^H-NMR (300 MHz CDCl_3_) δ 1.56–1.87 (m, 4 H, H-5 and H-7) 2.25–2.34 (m, 2 H, H-6) 2.38 (t, *J* = 6.3 Hz, 1 H, OH) 3.58–3.96 (m, 8 H, OCH_3_, H-1, H-2, H-3 and H-4) 4.58 (dd, *J* = 20.1 and 11.4 Hz, 2 H, CH_2_Ph) 4.68 (dd, *J* = 17.8 and 11.2 Hz, 2 H, CH_2_Ph) 7.24–7.41 (m, 10 H, arom. H).

^13^C NMR (75 MHz, CDCl_3_) δ 20.79, 29.38, 33.82, 51.51, 62.31, 63.11, 72.38, 73.71, 78.60, 79.84, 127.88, 128.00, 128.02, 128.11, 128.46, 128.52, 137.53, 137.75, 173.77.

Exact mass (ESI-MS) for C_23_H_30_N_3_O_5_ [M + H]^+^ found, 428.2177; Calcd., 428.2180.

#### (5R,6S,7S)-methyl-7-azido-5,6-di-O-benzyl-8-O-(2,3-di-O-benzyl-4,6-O-benzylidene-α-D-galactopyranosyl)-octanoate (**10**)

To a mixture of **11** (670 mg, 1.13 mmol) in THF (9 mL), a solution of **12** (323 mg, 0.76 mmol) in THF (6 mL) was added. The reaction mixture was cooled to −30 °C and TMSOTf (0.3 mL, 0.11 mmol) was added dropwise. After stirring for 2 h. at −30 °C, the reaction mixture was neutralized with Et_3_N and evaporated to dryness. The residue was purified by silica gel chromatography (hexane/EtOAc: 8/2 + 1% v/v Et_3_N) to afford **10** (438 mg, 67% yield) as colorless liquid which solidified upon storage.

^1^H-NMR (300 MHz CDCl_3_) δ 1.46–1.79 (m, 4 H, CH_2_) 2.20 (t, *J* = 6.6 Hz, 2 H, CH_2_) 3.52 (br s., 1 H, H-4”) 3.56–3.71 (m, 7 H, H-2, H-2”, H-4, Hb-6”, OCH_3_) 3.84 (dd, *J* = 12.5 and 1.5 Hz, 1 H, H-3”) 3.93 (dd, *J* = 9.8 and 2.9 Hz, 1 H, Ha-1) 3.97–4.06 (m, 3 H, Hb-1, H-3 and Hb-6”) 4.13 (dd, *J* = 3.2 and 1.0 Hz, 1 H, H-5”) 4.43 (d, *J* = 11.5 Hz, 1 H, CH_2_Ph) 4.50–4.63 (m, 4 H, CH_2_Ph) 4.69 (d, *J* = 12.3 Hz, 1 H, CH_2_Ph) 4.76 (d, *J* = 12.5 Hz, 1 H, CH_2_Ph) 4.80 (d, *J* = 11.8 Hz, 1 H, CH_2_Ph) 4.92 (d, *J* = 3.2 Hz, 1 H, H-1”) 5.41 (s, 1 H, H-8”) 7.14–7.36 (m, 23 H, arom. H) 7.44–7.48 (m, 2 H, arom. H).

^13^C NMR (75 MHz, CDCl_3_) δ 20.83, 29.23, 33.89, 51.47, 61.65, 63.00, 68.31, 69.31, 71.95, 72.00, 73.54, 73.84, 74.59, 75.41, 77.42, 78.42, 78.95, 99.14, 101.05, 126.33, 127.46, 127.50, 127.65, 127.69, 127.79, 127.83, 127.92, 128.09, 128.22, 128.26, 128.39, 128.85, 137.82, 137.91, 138.11, 138.73, 138.75, 173.80.

Exact mass (ESI-MS) for C_50_H_56_N_3_O_10_ [M + H]^+^ found, 858.3948; Calcd., 858.3960.

#### General procedure for AlMe_3_ assisted amide coupling

To a solution of glycoside **10** (500 mg, 0.58 mmol) in anhydrous CH_2_Cl_2_ (10 mL) was added the appropriate amine (5 eq.) and AlMe_3_ (2 M in heptanes) (2.5 eq.). The reaction mixture was stirred overnight at reflux temperature. Next the reaction mixture was cooled to 0 °C and water was added. The mixture was extracted with CH_2_Cl_2_ and the combined organic phase was washed with H_2_O and brine, dried over Na_2_SO_4_, filtered, and evaporated. Purification by column chromatography gave the desired amides **20a** (61%), **20b** (64%), **20c** (64%), **20d** (52%), **20e** (51%), **20 f** (68%), **20 g** (81%), **20 h** (60%).

#### (5R,6S,7S)-7-azido-5,6-di-O-benzyl-8-O-(2,3-di-O-benzyl-4,6-O-benzylidene-α-D-galactopyranosyl)-N-nonyloctanamide (**20a**)

^1^H-NMR (300 MHz CDCl_3_) δ 0.89 (t, *J* = 6.5 Hz, 3 H, terminal CH_3_) 1.23–1.35 (m, 12 H, CH_2_) 1.45 (dt, *J* = 13.4 and 7.8 Hz, 2 H, CH_2_) 1.53–1.81 (m, 4 H, CH_2_) 2.09 (t, *J* = 6.6 Hz, 2 H, CH_2_) 3.14–3.24 (m, 2 H, NHCH_2_) 3.59–3.81 (m, 5 H, H-2, H-3, H-4, H-5” and Ha-6”) 3.93 (dd, *J* = 12.6 and 1.5 Hz, 1 H, Ha-1) 3.98–4.04 (m, 1 H, Hb-6”) 4.06 (d, *J* = 3.2 Hz, 1 H, H-3”) 4.08–4.14 (m, 2 H, Hb-1 and H-2”) 4.20 (dd, *J* = 3.5 and 1.2 Hz, 1 H, H-4”) 4.49 (d, *J* = 11.5 Hz, 1 H, CH_2_Ph) 4.59–4.72 (m, 4 H, CH_2_Ph) 4.75 (d, *J* = 12.3 Hz, 1 H, CH_2_Ph) 4.82 (d, *J* = 12.3 Hz, 1 H, CH_2_Ph) 4.88 (d, *J* = 12.0 Hz, 1 H, CH_2_Ph) 4.98 (d, *J* = 3.3 Hz, 1 H, H-1”) 5.32 (t, *J* = 5.1 Hz, 1 H, (CO)NH) 5.48 (s, 1 H, H-8”) 7.22–7.43 (m, 23 H, arom. H) 7.51–7.56 (m, 2 H, arom. H). ^13^C NMR (75 MHz, CDCl_3_) δ 14.10, 21.81, 22.65, 26.93, 29.25, 29.29, 29.50, 29.67, 31.84, 36.57, 39.50, 61.55, 63.03, 68.35, 69.33, 71.96, 72.02, 73.59, 73.87, 74.58, 75.38, 75.84, 78.35, 79.20, 99.19, 101.06, 126.34, 127.47, 127.52, 127.68, 127.71, 127.75, 127.78, 127.89, 127.98, 128.11, 128.23, 128.27, 128.40, 128.43, 128.49, 128.87, 137.83, 137.98, 138.14, 138.74, 172.41.

Exact mass (ESI-MS) for C_58_H_76_N_5_O_9_ [M + NH_4_]^+^ found, 986.5641; Calcd., 986.5638.

#### (5R,6S,7S)-7-azido-5,6-di-O-benzyl-8-O-(2,3-di-O-benzyl-4,6-O-benzylidene-α-D-galactopyranosyl)-N-2-phenylethyl)octanamide (**20b**)

^1^H-NMR (300 MHz CDCl_3_) δ 1.52–1.76 (m, 4 H, CH_2_) 2.02–2.07 (m, 2 H, (CO)CH_2_) 2.73 (t, *J* = 6.9 Hz, 2 H, H-11) 3.44 (q, *J* = 6.8 Hz, 2 H, NHCH_2_) 3.56 (app. s, 1 H, H-2) 3.57–3.63 (m, 2 H, H-4 and H-3) 3.66–3.75 (m, 2 H, H-5” and Ha-6”) 3.88 (dd, *J* = 12.3 and 1.3 Hz, 1 H, H-3”) 3.95 (d, *J* = 3.3 Hz, 1 H, Ha-1) 3.97–4.06 (m, 2 H, H-2” and Hb-6”) 4.07–4.10 (m, 1 H, Hb-1) 4.15 (d, *J* = 2.9 Hz, 1 H, H-4”) 4.43 (d, *J* = 11.6 Hz, 1 H, CH_2_Ph) 4.54–4.66 (m, 4 H, CH_2_Ph) 4.71 (d, *J* = 12.4 Hz, 1 H, CH_2_Ph) 4.78 (d, *J* = 12.4 Hz, 1 H, CH_2_Ph) 4.83 (d, *J* = 11.8 Hz, 1 H, CH_2_Ph) 4.93 (d, *J* = 3.2 Hz, 1 H, H-1”) 5.29 (t, *J* = 5.6 Hz, 1 H, (CO)NH) 5.43 (s, 1 H, H-8”) 7.12–7.38 (m, 28 H, arom. H) 7.48 (dd, *J* = 7.3 and 2.3 Hz, 2 H, arom. H).

^13^C NMR (75 MHz, CDCl_3_) δ 21.71, 29.25, 35.70, 36.49, 40.49, 61.55, 63.03, 68.34, 69.32, 71.94, 72.01, 73.57, 73.85, 74.56, 75.37, 75.83, 77.86, 78.28, 78.35, 78.70, 79.08, 79.63, 99.17, 101.04, 126.32, 126.49, 127.46, 127.50, 127.66, 127.69, 127.76, 127.87, 127.95, 128.09, 128.21, 128.26, 128.38, 128.40, 128.61, 128.72, 128.85, 128.94, 128.97, 129.15, 129.23, 129.37, 129.49, 129.54, 129.68, 129.89, 130.00, 137.81, 137.96, 138.12, 138.72, 138.87, 140.00, 172.47.

Exact mass (ESI-MS) for C_57_H_62_N_4_NaO_9_ [M + Na]^+^ found, 969.4413; Calcd., 969.4409.

#### (5R,6S,7S)-7-azido-5,6-di-O-benzyl-8-O-(2,3-di-O-benzyl-4,6-O-benzylidene-α-D-galactopyranosyl)-N-(4-phenylbutyl)octanamide (**20c**)

^1^H-NMR (300 MHz CDCl_3_) δ 1.43–1.78 (m, 8 H, CH_2_) 2.07 (t, *J* = 6.8 Hz, 2 H, CH_2_) 2.62 (t, *J* = 7.5 Hz, 2 H, CH_2_) 3.21 (dd, *J* = 13.0 and 7.3 Hz, 2 H, NHCH_2_) 3.56 (app. s, 1 H, H-2) 3.60–3.67 (m, 2 H, H-4 and Ha-6”) 3.69–3.79 (m, 2 H, H-5” and H-3) 3.91 (dd, *J* = 13.0 and 2.0 Hz, 1 H, Ha-1) 3.97–4.02 (m, 1 H, Hb-6”) 4.04 (d, *J* = 2.9 Hz, 1 H, H-3”) 4.07–4.10 (m, 2 H, H-2” and Hb-1) 4.18 (d, *J* = 2.9 Hz, 1 H, H-4”) 4.47 (d, *J* = 11.5 Hz, 1 H, CH_2_Ph) 4.57–4.69 (m, 4 H, CH_2_Ph) 4.74 (d, *J* = 12.6 Hz, 1 H, CH_2_Ph) 4.81 (d, *J* = 12.6 Hz, 1 H, CH_2_Ph) 4.86 (d, *J* = 11.9 Hz, 1 H, CH_2_Ph) 4.98 (d, *J* = 3.2 Hz, 1 H, H-1”) 5.29 (t, *J* = 5.2 Hz, 1 H, (CO)NH) 5.47 (s, 1 H, H-8”) 7.14–7.41 (m, 28 H, arom. H) 7.49–7.53 (m, 2 H, arom. H).

^13^C NMR (75 MHz, CDCl_3_) δ 21.79, 28.64, 29.22, 35.44, 36.54, 39.27, 61.55, 63.03, 68.35, 69.33, 71.95, 72.02, 73.59, 73.87, 74.57, 75.38, 75.84, 77.21, 78.32, 79.18, 99.18, 101.05, 125.82, 126.33, 127.47, 127.52, 127.67, 127.70, 127.75, 127.77, 127.87, 127.98, 128.10, 128.22, 128.27, 128.33, 128.37, 128.39, 128.42, 128.86, 137.81, 137.98, 138.11, 138.72, 142.05, 172.45.

Exact mass (ESI-MS) for C_59_H_67_N_4_O_9_ [M + H]^+^ found, 975.4905; Calcd., 975.4903.

#### (5R,6S,7S)-7-azido-5,6-di-O-benzyl-8-O-(2,3-di-O-benzyl-4,6-O-benzylidene-α-D-galactopyranosyl)-N-(6-phenylhexyl)octanamide (**20d**)

^1^H-NMR (300 MHz CDCl_3_) δ 1.26–1.48 (m, 6 H, CH_2_) 1.52–1.78 (m, 6 H, CH_2_) 2.08 (t, *J* = 6.7 Hz, 2 H, CH_2_) 2.59 (t, *J* = 7.5 Hz, 2 H, CH_2_) 3.18 (dd, *J* = 13.4 and 7.2 Hz, 2 H, NHCH_2_) 3.59 (app. s, 1 H, H-2) 3.61–3.67 (m, 2 H, H-3 and H-4) 3.68–3.79 (m, 2 H, H-5” and Ha-6”) 3.91 (dd, *J* = 12.7 and 1.1 Hz, 1 H, Ha-1) 3.98–4.02 (m, 1 H, Hb-6”) 4.04 (d, *J* = 3.5 Hz, 1 H, H-3”) 4.07–4.12 (m, 2 H, Hb-1 and H-2”) 4.19 (d, *J* = 2.7 Hz, 1 H, H-4”) 4.47 (d, *J* = 11.7 Hz, 1 H, CH_2_Ph) 4.57–4.72 (m, 4 H, CH_2_Ph) 4.75 (d, *J* = 12.4 Hz, 1 H, CH_2_Ph) 4.81 (d, *J* = 12.4 Hz, 1 H, CH_2_Ph) 4.86 (d, *J* = 12.1 Hz, 1 H, CH_2_Ph) 4.97 (d, *J* = 3.5 Hz, 1 H, H-1”) 5.30 (t, *J* = 5.4 Hz, 1 H, (CO)NH) 5.47 (s, 1 H, H-8”) 7.15–7.42 (m, 28 H, arom. H) 7.50–7.54 (m, 2 H, arom. H).

^13^C NMR (75 MHz, CDCl_3_) δ 21.80, 26.77, 28.89, 29.23, 29.58, 31.31, 35.84, 36.55, 39.43, 61.54, 63.02, 68.34, 69.32, 71.95, 72.02, 73.57, 73.86, 74.56, 75.37, 75.83, 77.20, 78.33, 79.19, 99.17, 101.04, 125.62, 126.32, 127.46, 127.51, 127.67, 127.70, 127.74, 127.76, 127.87, 127.96, 128.09, 128.22, 128.24, 128.26, 128.35, 128.38, 128.42, 128.86, 137.81, 137.97, 138.11, 138.71, 142.59, 172.42.

Exact mass (ESI-MS) for C_61_H_71_N_4_O_9_ [M + H]^+^ found, 1003.5232; Calcd., 1003.5216.

#### (5R,6S,7S)-7-azido-5,6-di-O-benzyl-8-O-(2,3-di-O-benzyl-4,6-O-benzylidene-α-D-galactopyranosyl)-N-(8-phenyloctyl)octanamide (**20e**)

^1^H-NMR (300 MHz CDCl_3_) δ 1.23–1.49 (m, 10 H, CH_2_) 1.51–1.78 (m, 6 H, CH_2_) 2.08 (t, *J* = 6.0 Hz, 2 H, CH_2_) 2.59 (t, *J* = 7.5 Hz, 2 H, CH_2_) 3.18 (dd, *J* = 13.0 and 6.5 Hz, 2 H, NHCH_2_) 3.59 (app. s, 1 H, H-2) 3.61–3.68 (m, 2 H, H-3 and H-4) 3.68–3.80 (m, 2 H, H-5” and Ha-6”) 3.91 (dd, *J* = 12.4 and 1.4 Hz, 1 H, Ha-1) 4.98–4.03 (m, 1 H, Hb-6”) 4.04 (d, *J* = 3.2 Hz, 1 H, H-3”) 4.06–4.13 (m, 2 H, H-2” and Hb-1) 4.19 (dd, *J* = 3.1 and 0.9 Hz, 1 H, H-4”) 4.47 (d, *J* = 11.4 Hz, 1 H, CH_2_Ph) 4.56–4.71 (m, 4 H, CH_2_Ph) 4.75 (d, *J* = 12.4 Hz, 1 H, CH_2_Ph) 4.82 (d, *J* = 12.4 Hz, 1 H, CH_2_Ph) 4.86 (d, *J* = 11.8 Hz, 1 H, CH_2_Ph) 4.98 (d, *J* = 3.2 Hz, 1 H, H-1”) 5.31 (t, *J* = 5.1 Hz, 1 H, (CO)NH) 5.47 (s, 1 H, H-8”) 7.14–7.43 (m, 28 H, arom. H) 7.49–7.54 (m, 2 H, arom. H).

^13^C NMR (75 MHz, CDCl_3_) δ 21.82, 26.90, 29.22, 29.39, 29.66, 31.46, 35.95, 36.58, 39.48, 61.55, 63.03, 68.36, 69.34, 71.96, 72.04, 73.58, 73.87, 74.59, 75.38, 75.84, 77.21, 78.34, 79.21, 99.20, 101.06, 110.00, 125.57, 126.34, 127.48, 127.53, 127.68, 127.71, 127.76, 127.79, 127.89, 127.98, 128.11, 128.23, 128.27, 128.40, 128.43, 128.87, 137.83, 137.98, 138.14, 138.73, 142.82, 172.42.

Exact mass (ESI-MS) for C_63_H_75_N_4_O_9_ [M + H]^+^ found, 1031.5575; Calcd., 1031.5529.

#### (5R,6S,7S)-7-azido-5,6-di-O-benzyl-8-O-(2,3-di-O-benzyl-4,6-O-benzylidene-α-D-galactopyranosyl)-N-(3-pentylphenyl)octanamide (**20f**)

^1^H-NMR (300 MHz CDCl_3_) δ 0.88 (t, *J* = 6.7 Hz, 3 H, terminal CH_3_) 1.26–1.36 (m, 4 H, CH_2_) 1.54–1.65 (m, 4 H, CH_2_) 1.70–1.87 (m, 2 H, CH_2_) 2.26 (t, *J* = 6.9 Hz, 2 H, CH_2_) 2.56 (t, *J* = 8.0 Hz, 2 H, CH_2_) 3.54–3.59 (m, 2 H, H-2 and H-3) 3.62–3.69 (m, 2 H, H-4 and Ha-6”) 3.75 (dd, *J* = 6.7 and 3.6 Hz, 1 H, H-5”) 3.85 (dd, *J* = 12.4 and 1.2 Hz, 1 H, Ha-1) 3.93–4.07 (m, 4 H, H-2”, H-3”, Hb-1 and Hb-6”) 4.13 (d, *J* = 2.7 Hz, 1 H, H-4”) 4.43 (d, *J* = 11.5 Hz, 1 H, CH_2_Ph) 4.64–4.52 (m, 4 H, CH_2_Ph) 4.68 (d, *J* = 12.5 Hz, 1 H, CH_2_Ph) 4.75 (d, *J* = 12.5 Hz, 1 H, CH_2_Ph) 4.81 (d, *J* = 11.8 Hz, 1 H, CH_2_Ph) 4.91 (d, *J* = 3.2 Hz, 1 H, H-1”) 5.40 (s, 1 H, H-8”) 6.85 (d, *J* = 7.2 Hz, 1 H, arom. H) 6.99 (s, 1 H, (CO)NH) 7.08–7.37 (m, 26 H, arom. H) 7.46 (dd, *J* = 7.3 and 2.2 Hz, 2 H, arom. H).

^13^C NMR (75 MHz, CDCl_3_) δ 14.03, 21.78, 22.52, 28.95, 31.09, 31.52, 35.93, 37.45, 61.49, 63.04, 68.27, 69.32, 71.91, 72.06, 73.61, 73.95, 74.54, 75.38, 75.84, 77.21, 78.19, 79.37, 99.17, 101.05, 116.85, 116.95, 119.63, 124.29, 126.33, 127.48, 127.52, 127.67, 127.71, 127.79, 127.85, 127.94, 128.10, 128.23, 128.27, 128.39, 128.50, 128.75, 128.86, 137.78, 137.82, 137.91, 138.01, 138.72, 143.97, 170.72.

Exact mass (ESI-MS) for C_60_H_68_N_4_NaO_9_ [M + Na]^+^ found, 1011.4886; Calcd., 1011.4879.

#### (5R,6S,7S)-7-azido-5,6-di-O-benzyl-8-O-(2,3-di-O-benzyl-4,6-O-benzylidene-α-D-galactopyranosyl)-N-(4-pentylphenyl)octanamide (**20g**)

^1^H-NMR (300 MHz CDCl_3_) δ 0.88 (t, *J* = 6.8 Hz, 3 H, terminal CH_3_) 1.29–1.31 (m, 3 H, CH_2_) 1.55–1.67 (m, 5 H, CH_2_) 1.71–1.89 (m, 2 H, CH_2_) 2.26 (t, *J* = 6.8 Hz, 2 H, CH_2_) 2.57 (t, *J* = 7.6 Hz, 2 H, CH_2_) 3.59–3.67 (m, 2 H, H-2 and H-5”) 3.67–3.77 (m, 2 H, H-4 and Ha-6”) 3.82 (dd, *J* = 6.8 and 3.6 Hz, 1 H, H-3) 3.92 (dd, *J* = 12.5 and 1.5 Hz, 1 H, Ha-1) 3.98–4.14 (m, 4 H, Hb-1, H-2”, H-3” and Hb-6”) 4.20 (d, *J* = 2.5 Hz, 1 H, H-4”) 4.50 (d, *J* = 11.4 Hz, 1 H, CH_2_Ph) 4.58–4.72 (m, 4 H, CH_2_Ph) 4.75 (d, *J* = 12.3 Hz, 1 H, CH_2_Ph) 4.82 (d, *J* = 12.3 Hz, 1 H, CH_2_Ph) 4.88 (d, *J* = 11.7 Hz, 1 H, CH_2_Ph) 4.98 (d, *J* = 3.2 Hz, 1 H, H-1”) 5.47 (s, 1 H, H-8”) 7.04–7.14 (m, 3 H, arom. H) 7.22–7.43 (m, 25 H, (CO)NH and arom. H) 7.51–7.56 (m, 2 H, arom. H).

^13^C NMR (75 MHz, CDCl_3_) δ 14.02, 21.77, 22.52, 28.96, 31.18, 31.41, 35.30, 37.36, 61.50, 63.03, 68.26, 69.32, 71.91, 72.05, 73.60, 73.93, 74.54, 75.38, 75.83, 77.20, 78.21, 79.30, 99.16, 101.04, 119.77, 126.33, 127.48, 127.51, 127.67, 127.71, 127.78, 127.84, 127.94, 128.10, 128.22, 128.26, 128.39, 128.49, 128.81, 128.86, 135.43, 137.82, 137.91, 138.01, 138.71, 138.91, 170.66.

Exact mass (ESI-MS) for C_60_H_68_N_4_NaO_9_ [M + Na]^+^ found, 1011.4874; Calcd., 1011.4879.

#### (5R,6S,7S)-7-azido-5,6-di-O-benzyl-8-O-(2,3-di-O-benzyl-4,6-O-benzylidene-α-D-galactopyranosyl)-N-(2-(2-ethoxyethoxy)ethyl)octanamide (**20 h**)

^1^H-NMR (300 MHz CDCl_3_) δ 1.17 (t, *J* = 7.0 Hz, 3 H, terminal CH_3_) 1.46–1.75 (m, 4 H, CH_2_) 1.99–2.08 (m, 2 H, CH_2_) 3.39 (q, 2 H, *J* = 5.2 Hz, NHCH_2_) 3.43–3.62 (m, 11 H, CH_2_, H-2, H-3 and H-4) 3.63–3.73 (m, 2 H, H-5” and Ha-6”) 3.85 (dd, *J* = 12.6 and 1.8 Hz, 1 H, Ha-1) 3.91–3.97 (m, 1 H, Hb-6”) 3.99 (d, *J* = 3.0 Hz, 1 H, H-3”) 4.00–4.07 (m, 2 H, Hb-1 and H-2”) 4.13 (d, *J* = 3.0 Hz, 1 H, H-4”) 4.43 (d, *J* = 11.5 Hz, 1 H, CH_2_Ph) 4.54 (d, *J* = 11.5 Hz, 1 H, CH_2_Ph) 4.59–4.64 (m, 3 H, CH_2_Ph) 4.69 (d, *J* = 12.6 Hz, 1 H, CH_2_Ph) 4.76 (d, *J* = 12.6 Hz, 1 H, CH_2_Ph) 4.81 (d, *J* = 11.8 Hz, 1 H, CH_2_Ph) 4.92 (d, *J* = 3.2 Hz, 1 H, H-1”) 5.41 (s, 1 H, H-8”) 5.86 (t, *J* = 5.5 Hz, 1 H, (CO)NH) 7.16–7.37 (m, 23 H, arom. H) 7.44–7.48 (m, 2 H, arom. H).

^13^C NMR (75 MHz, CDCl_3_) δ 15.60, 22.06, 29.82, 36.89, 39.50, 62.02, 63.43, 67.07, 68.78, 69.75, 70.14, 70.29, 70.71, 72.39, 72.45, 73.97, 74.26, 75.01, 75.82, 76.24, 77.63, 78.87, 79.50, 99.57, 101.47, 126.76, 127.88, 127.93, 128.10, 128.12, 128.19, 128.33, 128.52, 128.64, 128.69, 128.81, 128.82, 129.27, 138.25, 138.40, 138.58, 139.16, 172.98.

Exact mass (ESI-MS) for C_55_H_67_N_4_O_11_ [M + H]^+^ found, 959.4799; Calcd., 959.4801.

#### Representative procedure for Staudinger reduction and acylation with EDC

To a solution of azide **20c** (350 mg, 0.36 mmol, 1eq.) in THF (5 mL) at room temperature, PMe_3_ (1 M solution of in THF, 5.38 mL, 5.38 mmol, 15 eq.) was added dropwise. After stirring for 3 h at room temperature, H_2_O (2 mL) was added and the reaction mixture was allowed to stir overnight at room temperature. Then the solvent was removed under reduced pressure. The crude product was dried by making azeotropic mixture with toluene to afford the crude amine. A mixture of the crude amine, EDC (112 mg, 0.72 mmol, 2 eq.) and octanoic acid (78 mg, 0.09 mL, 0.54 mmol, 1.5 eq.) in CH_2_Cl_2_ (10 mL) was stirred for 24 h at room temperature. The reaction mixture was diluted with CH_2_Cl_2_, washed with H_2_O (2 × 10 mL) and brine (1 × 10 mL), dried over Na_2_SO_4_, filtered, and evaporated to dryness. Purification by silica gel chromatography gave the desired the desired amides. **21a** (74%), **21b** (76%), **21c** (82%), **21d** (80%), **21e** (81%), **21 f** (87%), **21 g** (87%), **21 h** (70%).

#### (5R,6S,7S)-5,6-di-O-benzyl-8-O-(2,3-di-O-benzyl-4,6-O-benzylidene-α-D-galactopyranosyl)-7-octanamido-N-nonyloctanamide (**21a**)

^1^H-NMR (300 MHz CDCl_3_) δ 0.85 (app. td, *J* = 6.7 and 2.8 Hz, 6 H, 2 x terminal CH_3_) 1.19–1.35 (m, 18 H, CH_2_) 1.39–1.54 (m, 4 H, CH_2_) 1.60–1.83 (m, 6 H, CH_2_) 1.90–1.97 (m, 2 H, CH_2_) 2.02–2.10 (m, 2 H, CH_2_) 3.17 (dd, *J* = 12.9 and 6.9 Hz, 2 H, NHCH_2_)3.44 (m, 1 H, H-4) 3.52 (app. s, 1 H, H-5”) 3.74 (dd, *J* = 7.0 and 2.0 Hz, 1 H, H-3) 3.82 (d, *J* = 4.8 Hz, 2 H, H-1) 3.89–3.96 (m, 2 H, H-4” and Ha-6”) 4.04 (d, *J* = 3.6 Hz, 1 H, H-2”) 4.07–4.14 (m, 2 H, H-2 and Hb-6”) 4.18 (d, *J* = 3.2 Hz, 1 H, H-3”) 4.39 (d, *J* = 11.6 Hz, 1 H, CH_2_Ph) 4.47 (d, *J* = 11.6 Hz, 1 H, CH_2_Ph) 4.55 (d, *J* = 11.8 Hz, 1 H, CH_2_Ph) 4.62 (d, *J* = 11.8 Hz, 1 H, CH_2_Ph) 4.72–4.77 (m, 3 H, CH_2_Ph) 4.84 (d, *J* = 11.8 Hz, 1 H, CH_2_Ph) 4.93 (d, *J* = 3.4 Hz, 1 H, H-1”) 5.45 (s, 1 H, H-8”) 5.98 (d, *J* = 8.4 Hz, 1 H, NH(CO)) 6.05 (t, *J* = 5.7 Hz, 1 H, (CO)NH) 7.19–7.40 (m, 23 H, arom. H) 7.47–7.52(m, 2 H, arom. H).

^13^C NMR (75 MHz, CDCl_3_) δ 14.09, 21.84, 22.64, 25.65, 26.99, 28.82, 29.07, 29.26, 29.33, 29.52, 29.60, 31.76, 31.84, 36.03, 36.65, 39.48, 50.02, 63.00, 68.08, 69.36, 71.52, 71.72, 73.28, 73.98, 74.18, 75.61, 76.10, 78.88, 99.60, 101.01, 126.27, 127.57, 127.63, 127.70, 127.75, 127.86, 127.89, 127.92, 128.11, 128.31, 128.38, 128.41, 128.88, 137.73, 138.23, 138.26, 138.39, 138.55, 172.81, 173.01.

Exact mass (ESI-MS) for C_66_H_89_N_2_O_10_ [M + H]^+^ found, 1069.6512; Calcd., 1069.6517.

#### (5R,6S,7S)-5,6-di-O-benzyl-8-O-(2,3-di-O-benzyl-4,6-O-benzylidene-α-D-galactopyranosyl)-7-octanamido-N-(2-phenylethyl)octanamide (**21b**)

^1^H-NMR (300 MHz CDCl_3_) δ 0.89 (t, *J* = 6.6 Hz, 3 H, terminal CH_3_) 1.20–1.33 (m, 7 H, CH_2_) 1.44–1.82 (m, 7 H, CH_2_) 1.88–1.98 (m, 2 H, CH_2_) 2.02–2.07 (m, 2 H, CH_2_) 2.78 (t, *J* = 7.3 Hz, 2 H, CH_2_) 3.40–3.54 (m, 4 H, H-4, H-5” and CH_2_) 3.75 (dd, *J* = 7.0 and 2.4 Hz, 1 H, H-3) 3.79–3.94 (m, 3 H, H-1 and Ha-6”) 3.96 (d, *J* = 3.3 Hz, 1 H, H-4”) 4.07 (d, *J* = 3.3 Hz, 1 H, H-2”) 4.08–4.16 (m, 2 H, H-2 and Hb-6”) 4.18 (d, *J* = 3.2 Hz, 1 H, H-3”) 4.41 (d, *J* = 11.7 Hz, 1 H, CH_2_Ph) 4.50 (d, *J* = 11.7 Hz, 1 H, CH_2_Ph) 4.57 (d, *J* = 11.7 Hz, 1 H, CH_2_Ph) 4.64 (d, *J* = 11.4 Hz, 1 H, CH_2_Ph) 4.74–4.79 (m, 3 H, CH_2_Ph) 4.86 (d, *J* = 11.3 Hz, 1 H, CH_2_Ph) 4.96 (d, *J* = 3.6 Hz, 1 H, H-1”) 5.46 (s, 1 H, H-8”) 5.98 (d, *J* = 8.5 Hz, 1 H, NH(CO)) 6.14 (t, *J* = 5.6 Hz, 1 H, (CO)NH) 7.15–7.42 (m, 28 H, arom. H) 7.50–7.54 (m, 2 H, arom. H).

^13^C NMR (75 MHz, CDCl_3_) δ 14.09, 21.77, 22.63, 25.67, 28.83, 29.08, 29.32, 31.77, 35.70, 36.00, 36.66, 40.60, 50.03, 62.99, 68.06, 69.35, 71.50, 71.73, 73.31, 74.02, 74.14, 75.64, 76.09, 76.57, 77.20, 77.43, 78.85, 78.88, 99.56, 101.01, 126.27, 126.33, 127.59, 127.64, 127.71, 127.79, 127.85, 127.91, 127.93, 128.11, 128.33, 128.36, 128.39, 128.42, 128.50, 128.71, 128.89, 137.73, 138.24, 138.27, 138.36, 138.54, 139.09, 172.92, 173.08.

Exact mass (ESI-MS) for C_65_H_79_N_2_O_10_ [M + H]^+^ found, 1047.5768; Calcd., 1047.5729.

#### (5R,6S,7S)-5,6-di-O-benzyl-8-O-(2,3-di-O-benzyl-4,6-O-benzylidene-α-D-galactopyranosyl)-7-octanamido-N-(4-phenylbutyl)octanamide (**21c**)

^1^H-NMR (300 MHz CDCl_3_) δ 0.86 (t, *J* = 6.6 Hz, 3 H, terminal CH_3_) 1.17–1.28 (m, 9 H, CH_2_) 1.40–1.51 (m, 4 H, CH_2_) 1.53–1.81 (m, 7 H, CH_2_) 1.82–2.08 (m, 2 H, CH_2_) 2.57 (t, *J* = 7.5 Hz, 2 H, CH_2_) 3.19 (q, *J* = 6.6 Hz, 2 H, NHCH_2_) 3.43–3.51 (m, 2 H, H-4 and H-5”) 3.72 (dd, *J* = 6.8 and 1.6 Hz, 1 H, H-3) 3.80 (d, *J* = 4.8 Hz, 2 H, H-1) 3.87–3.95 (m, 2 H, H-4” and Ha-6”) 4.04 (d, *J* = 3.2 Hz, 1 H, H-2”) 4.06–4.14 (m, 2 H, H-2 and Hb-6”) 4.17 (d, *J* = 3.0 Hz, 1 H, H-3”) 4.38 (d, *J* = 11.5 Hz, 1 H, CH_2_Ph) 4.46 (d, *J* = 11.5 Hz, 1 H, CH_2_Ph) 4.54 (d, *J* = 11.7 Hz, 1 H, CH_2_Ph) 4.61 (d, *J* = 11.6 Hz, 1 H, CH_2_Ph) 4.71–4.77 (m, 3 H, CH_2_Ph) 4.84 (d, *J* = 11.6 Hz, 1 H, CH_2_Ph) 4.92 (d, *J* = 3.4 Hz, 1 H, H-1”) 5.44 (s, 1 H, H-8”) 5.91 (d, *J* = 8.5 Hz, 1 H, NH(CO)) 6.08 (t, *J* = 5.4 Hz, 1 H, (CO)NH) 7.09–7.40 (m, 28 H, arom. H) 7.46–7.52 (m, 2 H, arom. H).

^13^C NMR (75 MHz, CDCl_3_) δ 14.08, 21.80, 22.63, 25.65, 28.70, 28.77, 29.07, 29.18, 29.31, 31.76, 35.49, 35.96, 36.65, 39.24, 50.03, 63.00, 68.10, 69.36, 71.51, 71.74, 73.30, 73.99, 74.16, 75.61, 76.10, 77.20, 78.86, 99.64, 101.01, 125.72, 126.27, 127.56, 127.63, 127.72, 127.76, 127.88, 127.89, 127.91, 128.11, 128.27, 128.32, 128.34, 128.39, 128.42, 128.89, 129.96, 137.72, 138.22, 138.24, 138.39, 138.55, 142.18, 172.94, 173.06.

Exact mass (ESI-MS) for C_67_H_83_N_2_O_10_ [M + H]^+^ found, 1075.6052; Calcd., 1075.6042.

#### (5R,6S,7S)-5,6-di-O-benzyl-8-O-(2,3-di-O-benzyl-4,6-O-benzylidene-α-D-galactopyranosyl)-7-octanamido-N-(6-phenylhexyl)octanamide (**21d**)

^1^H-NMR (300 MHz CDCl_3_) δ 0.86 (t, *J* = 6.6 Hz, 3 H, terminal CH_3_) 1.16–1.37 (m, 12 H, CH_2_) 1.38–1.83 (m, 10 H, CH_2_) 1.87–2.11 (m, 4 H, CH_2_) 2.56 (t, *J* = 7.5 Hz, 2 H, CH_2_) 3.16 (q, *J* = 7.5 Hz, 2 H, NHCH_2_) 3.44–3.56 (m, 2 H, H-4 and H-5”) 3.74 (dd, *J* = 6.8 and 2.4 Hz, 1 H, H-3) 3.82 (d, *J* = 4.6 Hz, 2 H, H-1) 3.88–3.99 (m, 2 H, H-4” and Ha-6”) 4.05 (d, *J* = 3.3 Hz, 1 H, H-2”) 4.07–4.15 (m, 2 H, H-2 and Hb-6”) 4.18 (d, *J* = 3.2 Hz, 1 H, H-3”) 4.39 (d, *J* = 11.5 Hz, 1 H, CH_2_Ph) 4.48 (d, *J* = 11.4 Hz, 1 H, CH_2_Ph) 4.55 (d, *J* = 11.4 Hz, 1 H, CH_2_Ph) 4.62 (d, *J* = 11.6 Hz, 1 H, CH_2_Ph) 4.72–4.79 (m, 3 H, CH_2_Ph) 4.84 (d, *J* = 11.6 Hz, 1 H, CH_2_Ph) 4.93 (d, *J* = 3.5 Hz, 1 H, H-1”) 5.45 (s, 1 H, H-8”) 5.93 (d, *J* = 8.4 Hz, 1 H, NH(CO)) 6.04 (t, *J* = 5.7 Hz, 1 H, (CO)NH) 7.11–7.43 (m, 28 H, arom. H) 7.48–7.52 (m, 2 H, arom. H).

^13^C NMR (75 MHz, CDCl_3_) δ 14.08, 21.83, 22.63, 25.65, 26.82, 28.83, 28.94, 29.07, 29.31, 29.53, 31.35, 31.75, 35.85, 36.03, 36.66, 39.42, 50.04, 63.00, 68.14, 69.36, 71.53, 71.75, 73.28, 73.96, 74.19, 75.61, 76.11, 77.21, 78.90, 99.66, 101.01, 125.58, 126.27, 127.55, 127.62, 127.71, 127.74, 127.88, 127.91, 128.11, 128.21, 128.32, 128.34, 128.39, 128.42, 128.89, 137.73, 138.23, 138.25, 138.42, 138.56, 142.64, 172.82, 173.00.

Exact mass (ESI-MS) for C_69_H_86_N_2_NaO_10_ [M + Na]^+^ found, 1125.6173; Calcd., 1125.6175.

#### (5R,6S,7S)-5,6-di-O-benzyl-8-O-(2,3-di-O-benzyl-4,6-O-benzylidene-α-D-galactopyranosyl)-7-octanamido-N-(8-phenyloctyl)octanamide (**21e**)

^1^H-NMR (300 MHz CDCl_3_) δ 0.87 (t, *J* = 6.9 Hz, 3 H, terminal CH_3_) 1.17–1.35 (m, 14 H, CH_2_) 1.37–1.84 (m, 12 H, CH_2_) 1.87–2.00 (m, 2 H, CH_2_) 2.02–2.11 (m, 2 H, CH_2_) 2.58 (t, *J* = 7.6 Hz, 2 H, CH_2_) 3.16 (dd, *J* = 12.8 and 7.5 Hz, 2 H, NH-CH_2_) 3.45–3.51 (m, 1 H, H-4) 3.53 (br. s, 1 H, H-5”) 3.75 (dd, *J* = 6.8 and 2.3 Hz, 1 H, H-3) 3.84 (d, *J* = 4.9 Hz, 2 H, H-1) 3.88–3.97 (m, 2 H, H-4” and Ha-6”) 4.05 (d, *J* = 3.4 Hz, 1 H, H-2”) 4.07–4.17 (m, 2 H, H-2 and Hb-6”) 4.19 (d, *J* = 2.9 Hz, 1 H, H-3”) 4.42 (d, *J* = 11.6 Hz, 1 H, CH_2_Ph) 4.54 (dd, *J* = 22.3 and 11.4 Hz, 2 H, CH_2_Ph) 4.65 (d, *J* = 11.6 Hz, 1 H, CH_2_Ph) 4.75–4.80 (m, 3 H, CH_2_Ph) 4.87 (d, *J* = 11.6 Hz, 1 H, CH_2_Ph) 4.96 (d, *J* = 3.5 Hz, 1 H, H-1”) 5.48 (s, 1 H, H-8”) 5.93 (d, *J* = 8.5 Hz, 1 H, NH(CO)) 6.04 (t, *J* = 5.6 Hz, 1 H, (CO)NH) 7.14–7.21 (m, 2 H, arom. H) 7.21–7.42 (m, 26 H, arom. H) 7.49–7.55 (m, 2 H, arom. H).

^13^C NMR (75 MHz, CDCl_3_) δ 14.08, 21.85, 22.63, 25.65, 26.96, 28.82, 29.08, 29.25, 29.31, 29.42, 29.60, 31.46, 31.77, 35.94, 36.04, 36.65, 39.46, 50.03, 63.00, 68.07, 69.37, 71.52, 71.73, 73.31, 73.99, 74.18, 75.61, 76.10, 77.20, 78.88, 78.91, 99.61, 101.03, 125.54, 126.28, 127.59, 127.63, 127.71, 127.77, 127.86, 127.91, 128.12, 128.20, 128.32, 128.35, 128.40, 128.43, 128.90, 137.74, 138.24, 138.38, 138.56, 142.82, 172.82, 173.02.

Exact mass (ESI-MS) for C_71_H_90_N_2_NaO_10_ [M + Na]^+^ found, 1153.6510; Calcd., 1153.6488.

#### (5R,6S,7S)-5,6-di-O-benzyl-8-O-(2,3-di-O-benzyl-4,6-O-benzylidene-α-D-galactopyranosyl)-7-octanamido-N-(3-pentylphenyl)octanamide (**21f**)

^1^H-NMR (300 MHz CDCl_3_) δ 0.83–0.88 (m, 6 H, 2 x terminal CH_3_) 1.22–1.33 (m, 12 H, CH_2_) 1.47–1.83 (m, 7 H, CH_2_) 1.85–2.03 (m, 3 H, CH_2_) 2.11–2.25 (m, 2 H, CH_2_) 2.54 (t, *J* = 7.4 Hz, 2 H, CH_2_) 3.46–3.53 (m, 2 H, H-4 and H-5”) 3.73 (dd, *J* = 7.7 and 1.5 Hz, 1 H, H-3) 3.80 (d, *J* = 4.9 Hz, 2 H, H-1) 3.85–3.93 (m, 1 H, Ha-6”) 3.95 (d, *J* = 3.3 Hz, 1 H, H-4”) 4.05 (d, *J* = 3.5 Hz, 1 H, H-2”) 4.07–4.14 (m, 2 H, H-2 and Hb-6”) 4.17 (d, *J* = 3.3 Hz, 1 H, H-3”) 4.38 (d, *J* = 11.6 Hz, 1 H, CH_2_Ph) 4.48 (d, *J* = 11.6 Hz, 1 H, CH_2_Ph) 4.57 (d, *J* = 11.6 Hz, 1 H, CH_2_Ph) 4.62 (d, *J* = 11.5 Hz, 1 H, CH_2_Ph) 4.74–4.80 (m, 3 H, CH_2_Ph) 4.86 (d, *J* = 11.4 Hz, 1 H, CH_2_Ph) 4.94 (d, *J* = 3.6 Hz, 1 H, H-1”) 5.44 (s, 1 H, H-8”) 6.00 (d, *J* = 8.6 Hz, 1 H, NH(CO)) 6.86 (d, *J* = 7.6 Hz, 1 H, arom. H) 7.12 (t, *J* = 7.9 Hz, 1 H, arom. H) 7.21–7.41 (m, 24 H, arom. H) 7.44–7.53 (m, 3 H, arom. H) 8.36 (s, 1 H, (CO)NH).

^13^C NMR (75 MHz, CDCl_3_) δ 14.03, 21.67, 22.53, 22.63, 25.70, 29.09, 29.33, 31.10, 31.54, 31.78, 36.01, 36.72, 50.00, 63.04, 67.99, 69.32, 71.49, 71.70, 73.26, 74.02, 74.13, 75.59, 76.10, 78.63, 99.55, 101.01, 116.86, 119.69, 123.80, 126.27, 127.56, 127.65, 127.73, 127.78, 127.82, 127.90, 127.93, 128.00, 128.12, 128.34, 128.37, 128.41, 128.45, 128.90, 137.74, 138.10, 138.14, 138.44, 138.56, 143.74, 171.59, 173.24.

Exact mass (ESI-MS) for C_68_H_84_KN_2_O_10_ [M + K]^+^ found, 1127.5773; Calcd., 1127.5758.

#### (5R,6S,7S)-5,6-di-O-benzyl-8-O-(2,3-di-O-benzyl-4,6-O-benzylidene-α-D-galactopyranosyl)-7-octanamido-N-(4-pentylphenyl)octanamide (**21g**)

^1^H-NMR (300 MHz CDCl_3_) δ 0.86–0.93 (m, 6 H, 2 x terminal CH_3_) 1.22–1.38 (m, 12 H, CH_2_) 1.50–1.84 (m, 6 H, CH_2_) 1.87–2.07 (m, 3 H, CH_2_) 2.09–2.28 (m, 2 H, CH_2_) 2.54 (t, *J* = 7.1 Hz, 2 H, CH_2_) 3.46–3.54 (m, 2 H, H-4 and H-5”) 3.75 (dd, *J* = 8.0, 1.2 Hz, 1 H, H-3) 3.79–3.83 (m, 2 H, H-1) 3.89 (dd, *J* = 12.5 and 1.3 Hz, 1 H, Ha-6”) 3.95 (dd, *J* = 10.0 and 3.5 Hz, 1 H, H-4”) 4.08 (d, *J* = 3.5 Hz, 1 H, H-2”) 4.09–4.16 (m, 2 H, H-2 and Hb-6”) 4.19 (d, *J* = 2.8 Hz, 1 H, H-3”) 4.39 (d, *J* = 11.6 Hz, 1 H, CH_2_Ph) 4.49 (d, *J* = 11.6 Hz, 1 H, CH_2_Ph) 4.59 (d, *J* = 11.6 Hz, 1 H, CH_2_Ph) 4.64 (d, *J* = 11.3 Hz, 1 H, CH_2_Ph) 4.76–4.83 (m, 3 H, CH_2_Ph) 4.88 (d, *J* = 11.3 Hz, 1 H, CH_2_Ph) 4.95 (d, *J* = 3.5 Hz, 1 H, H-1”) 5.46 (s, 1 H, H-8”) 6.16 (d, *J* = 8.7 Hz, 1 H, NH(CO)) 7.06 (d, *J* = 8.5 Hz, 2 H, arom. H) 7.22–7.37(m, 22 H, arom. H) 7.38–7.46 (m, 3 H, arom. H) 7.49–7.5(m, 2 H, arom. H) 8.49 (s, 1 H, (CO)NH).

^13^C NMR (75 MHz, CDCl_3_) δ 14.02, 14.04, 14.08, 21.68, 22.52, 22.58, 22.63, 24.75, 25.69, 28.11, 28.90, 29.03, 29.09, 29.34, 31.23, 31.43, 31.62, 31.78, 33.64, 35.34, 36.51, 36.70, 49.97, 63.01, 67.87, 69.31, 71.50, 71.66, 73.27, 74.07, 74.12, 75.60, 76.07, 77.20, 78.55, 99.40, 101.01, 119.67, 126.27, 127.59, 127.68, 127.71, 127.82, 127.90, 127.94, 127.98, 128.12, 128.35, 128.37, 128.41, 128.44, 128.59, 128.90, 136.28, 137.72, 138.09, 138.14, 138.28, 138.35, 138.53, 171.57, 173.39.

Exact mass (ESI-MS) for C_68_H_85_N_2_O_10_ [M + H]^+^ found, 1089.6204; Calcd., 1089.6199.

#### (5R,6S,7S)-5,6-di-O-benzyl-8-O-(2,3-di-O-benzyl-4,6-O-benzylidene-α-D-galactopyranosyl)-7-octanamido-N-(2-(2-ethoxyethoxy)ethyl)octanamide (**21 h**)

^1^H-NMR (300 MHz CDCl_3_) δ 0.89 (t, *J* = 6.8 Hz, 3 H, terminal CH_3_) 1.18–1.35 (m, 12 H, CH_2_ and terminal CH_3_) 1.45–1.56 (m, 2 H, CH_2_) 1.60–1.88 (m, 3 H, CH_2_) 1.89–1.97 (m, 2 H, CH_2_) 2.10 (t, *J* = 6.2 Hz, 2 H, CH_2_) 3.40–3.48 (m, 2 H, CH_2_) 3.50–3.62 (m, 10 H, H-4, H-5” and CH_2_) 3.78 (dd, *J* = 6.1 and 2.4 Hz, 1 H, H-3) 3.80–3.99 (m, 4 H, CH_2_-1, H-2 and Ha-6”) 4.07 (d, *J* = 3.3 Hz, 1 H, H-4”) 4.10–4.18 (m, 1 H, Hb-6”) 4.19–4.25 (m, 2 H, H-2” and H-3”) 4.46 (d, *J* = 11.6 Hz, 1 H, CH_2_Ph) 4.51 (d, *J* = 11.6 Hz, 1 H, CH_2_Ph) 4.59 (d, *J* = 11.6 Hz, 1 H, CH_2_Ph) 4.65 (d, *J* = 11.6 Hz, 1 H, CH_2_Ph) 4.73–4.78 (m, 3 H, CH_2_Ph) 4.87 (d, *J* = 11.4 Hz, 1 H, CH_2_Ph) 4.96 (d, *J* = 3.4 Hz, 1 H, H-1”) 5.48 (s, 1 H, H-8”) 5.89 (d, *J* = 8.4 Hz, 1 H, NH(CO)) 6.26 (t, *J* = 5.3 Hz, 1 H, (CO)NH) 7.22–7.44 (m, 23 H, arom. H) 7.51–7.55 (m, 2 H, arom. H).

^13^C NMR (75 MHz, CDCl_3_) δ 14.08, 15.16, 21.82, 22.63, 25.67, 29.06, 29.16, 29.31, 31.75, 36.13, 36.68, 39.04, 50.12, 62.96, 66.61, 68.11, 69.38, 69.70, 69.81, 70.22, 71.57, 71.78, 73.30, 73.90, 74.23, 75.64, 76.12, 77.20, 78.94, 79.16, 99.58, 101.00, 126.28, 127.56, 127.60, 127.67, 127.70, 127.72, 127.88, 127.90, 128.09, 128.30, 128.33, 128.38, 128.41, 128.85, 137.77, 138.28, 138.30, 138.45, 138.60, 172.91, 173.00.

Exact mass (ESI-MS) for C_63_H_83_N_2_O_12_ [M + H]^+^ found, 1059.5945; Calcd., 1059.5941.

#### General procedure for debenzylation

A solution of the protected amide (0.06 mmol) in CHCl_3_ (3 mL) and EtOH (9 mL) was hydrogenated under atmospheric pressure in the presence of palladium black (35 mg). Upon reaction completion, the mixture was filtered through celite. The filter cake was rinsed with CHCl_3_ and EtOH and the filtrate was evaporated to dryness. After purification by silica gel chromatography (10% → 18% MeOH in CH_2_Cl_2_), final compounds **5a** (43%), **5b** (30%), **5c** (42%), **5d** (56%), **5e** (56%), **5f** (78%), **5g** (71%), **5h** (53%) were obtained as white powders.

#### (5R,6S,7S)-5,6-dihydroxy-8-O-(α-D-galactopyranosyl)-7-octanamido-N-nonyloctanamide (**5a**)

^1^H-NMR (300 MHz, pyridine-d_5_) δ 0.77–0.91 (m, 6 H, 2 x terminal CH_3_) 1.08–1.38 (m, 20 H, CH_2_) 1.59 (dt, *J* = 14.8 and 7.6 Hz, 2 H, CH_2_) 1.77 (dt, *J* = 14.8 and 7.6 Hz, 2 H, CH_2_) 1.92–2.04 (m, 1 H, CH_2_) 2.17–2.29 (m, 1 H, CH_2_) 2.34–2.60 (m, 6 H, CH_2_) 3.46 (dd, *J* = 13.0 and 6.8 Hz, 2 H, NHCH_2_) 4.26–4.37 (m, 3 H, Ha-1, H-3 and H-4) 4.37–4.45 (m, 3 H, H-4” and H-6”) 4.48 (q, *J* = 6.2 Hz, 1 H, H-3”) 4.55 (d, *J* = 2.9 Hz, 1 H, H-5”) 4.60–4.69 (m, 2 H, Hb-1 and H-2”) 5.19–5.28 (m, 1 H, H-2) 5.56 (d, *J* = 3.8 Hz, 1 H, H-1”) 6.36 (br. s, 6 H, OH) 8.30 (t, *J* = 5.2 Hz, 1 H, NH(CO)) 8.46 (d, *J* = 8.6 Hz, 1 H, (CO)NH).

^13^C NMR (75 MHz, pyridine-d_5_) δ 14.60, 14.65, 23.23, 23.28, 23.47, 26.71, 27.78, 29.73, 29.88, 30.01, 30.18, 30.38, 30.66, 32.27, 32.43, 34.38, 37.14, 37.35, 40.11, 51.78, 63.03, 68.98, 70.68, 71.37, 71.96, 72.61, 73.42, 77.19, 101.92, 173.65.

Exact mass (ESI-MS) for C_31_H_61_N_2_O_10_ [M + H]^+^ found, 621.4347; Calcd., 621.4326; m.p.: 95–97 °C.

#### (5R,6 S,7S)-5,6-dihydroxy-8-O-(α-D-galactopyranosyl)-7-octanamido-N-(2-phenylethyl)octanamide (**5b**)

^1^H-NMR (300 MHz, pyridine-d_5_) δ 0.80 (t, *J* = 6.2 Hz, 3 H, terminal CH_3_) 1.11–1.35 (m, 8 H, CH_2_) 1.77 (dt, *J* = 14.6 and 7.3 Hz, 2 H, CH_2_) 1.89–2.04 (m, 1 H, CH_2_) 2.11–2.26 (m, 1 H, CH_2_) 2.31–2.55 (m, 6 H, CH_2_) 2.93 (t, *J* = 7.2 Hz, 2 H, CH_2_Ph) 3.68 (q, *J* = 6.8 Hz, 2 H, NHCH_2_) 4.26–4.32 (m, 1 H, H-3) 4.32–4.54 (m, 6 H, H-4, Ha-1, H-4”, H-5” and CH_2_-6”) 4.56 (d, *J* = 2.5 Hz, 1 H, H-3”) 4.61–4.69 (m, 2 H, H-2” and Hb-1) 5.20–5.30 (m, 1 H, H-2) 5.57 (d, *J* = 3.4 Hz, 1 H, H-1”) 7.24–7.35 (m, 3 H, arom. H) 8.43–8.54 (m, 2 H, arom. H).

^13^C NMR (75 MHz, pyridine-d_5_) δ 29.03, 37.66, 37.83, 41.15, 44.16, 44.44, 46.70, 48.82, 51.21, 51.58, 51.69, 56.18, 66.16, 77.46, 83.33, 85.09, 85.81, 86.38, 87.06, 87.83, 91.60, 116.30, 141.31, 143.62, 144.10, 155.13, 188.15, 188.27.

Exact mass (ESI-MS) for C_30_H_51_N_2_O_10_ [M + H]^+^ found, 599.3541; Calcd., 599.3538; m.p. 56–58 °C.

#### (5R,6S,7S)-5,6-dihydroxy-8-O-(α-D-galactopyranosyl)-7-octanamido-N-(4-phenylbutyl)octanamide (**5c**)

^1^H-NMR (300 MHz, pyridine-d_5_) δ 0.80 (t, *J* = 6.6 Hz, 3 H, terminal CH_3_) 1.07–1.42 (m, 10 H, CH_2_) 1.54–1.67 (m, 4 H, CH_2_) 1.76 (dt, *J* = 14.9 and 7.5 Hz, 2 H, CH_2_) 1.90–2.04 (m, 1 H, CH_2_) 2.12–2.27 (m, 1 H, CH_2_) 2.30–2.58 (m, 6 H, CH_2_) 3.44 (q, *J* = 5.8 Hz, 2 H, NHCH_2_) 4.25–4.44 (m, 6 H, Ha-1, H-3, H-4, H-4” and CH_2_-6”) 4.48 (q, *J* = 5.7 Hz, 1 H, H-3”) 4.55 (d, *J* = 2.6 Hz, 1 H, H-5”) 4.60–4.68 (m, 2 H, Hb-1 and H-2”) 5.19–5.28 (m, 1 H, H-2) 5.56 (d, *J* = 3.8 Hz, 1 H, H-1”) 6.47 (br. s, 6 H, OH) 7.17–7.25 (m, 3 H, arom. H) 7.28–7.35 (m, 2 H, arom. H) 8.28 (t, *J* = 5.3 Hz, 1 H, (CO)NH) 8.46 (d, *J* = 8.6 Hz, 1 H, NH(CO)).

^13^C NMR (75 MHz, pyridine-d_5_) δ 14.60, 23.23, 23.46, 26.72, 29.58, 29.72, 30.01, 30.21, 30.39, 32.27, 34.41, 36.07, 37.14, 37.33, 39.79, 51.78, 63.04, 68.98, 70.68, 71.38, 71.97, 72.62, 73.43, 77.21, 101.92, 126.46, 129.09, 129.23, 136.58, 143.22, 173.66, 173.67.

Exact mass (ESI-MS) for C_32_H_55_N_2_O_10_ [M + H]^+^ found, 627.3860; Calcd., 627.3851; m.p. 57–59 °C.

#### (5R,6S,7S)-5,6-dihydroxy-8-O-(α-D-galactopyranosyl)-7-octanamido-N-(6-phenylhexyl)octanamide(**5d**)

^1^H-NMR (300 MHz, pyridine-d_5_) δ 0.77–0.87 (m, 3 H, terminal CH_3_) 1.09–1.43 (m, 13 H, CH_2_) 1.54 (dt, *J* = 12.6 and 6.3 Hz, 3 H, CH_2_) 1.77 (dt, *J* = 14.4 and 7.1 Hz, 2 H, CH_2_) 1.91–2.05 (m, 1 H, CH_2_) 2.16–2.31 (m, 1 H, CH_2_) 2.33–2.60 (m, 6 H, CH_2_) 3.03 (q, *J* = 7.0 Hz, 2 H, CH_2_) 3.43 (d, *J* = 5.8 Hz, 2 H, NHCH_2_) 4.27–4.46 (m, 6 H, Ha-1, H-3, H-4, H-4” and CH_2_-6”) 4.46–4.53 (m, 1 H, H-5”) 4.56 (app. s, 1 H, H-3”) 4.60–4.69 (m, 2 H, Hb-1 and H-2”) 5.19–5.29 (m, 1 H, H-2) 5.56 (d, *J* = 2.4 Hz, 1 H, H-1”) 6.40 (br. s, 6 H, OH) 7.19–7.29 (m, 3 H, arom. H) 7.30–7.39 (m, 2 H, arom. H) 8.29 (t, *J* = 5.7, 1 H, (CO)NH) 8.49 (d, *J* = 8.2 Hz, 1 H, NH(CO)).

^13^C NMR (75 MHz, pyridine-d_5_) δ 8.91, 14.59, 23.22, 23.45, 26.70, 27.58, 29.58, 29.71, 29.99, 30.35, 30.53, 32.08, 32.26, 34.37, 36.40, 37.13, 37.33, 40.05, 46.18, 51.78, 63.00, 68.92, 70.68, 71.34, 71.95, 72.56, 73.39, 77.17, 101.89, 126.41, 129.07, 129.21, 143.51, 173.66, 173.68.

Exact mass (ESI-MS) for C_34_H_59_N_2_O_10_ [M + H]^+^ found, 655.4161; Calcd., 655.4164; m.p. 135–137 °C.

#### (5R,6S,7S)-5,6-dihydroxy-8-O-(α-D-galactopyranosyl)-7-octanamido-N-(8-phenyloctyl)octanamide (**5e**)

^1^H-NMR (300 MHz, pyridine-d_5_) δ 0.80 (t, *J* = 6.3 Hz, 3 H, terminal CH_3_) 1.09–1.36 (m, 16 H, CH_2_) 1.47–1.64 (m, 4 H, CH_2_) 1.69–1.83 (m, 2 H, CH_2_) 1.91–2.05 (m, 1 H, CH_2_) 2.14–2.30 (m, 1 H, CH_2_) 2.34–2.60 (m, 8 H, CH_2_) 3.45 (dd, *J* = 13.1 and 6.8 Hz, 2 H, NHCH_2_) 4.26–4.45 (m, 6 H, Ha-1, H-3, H-4, H-4” and CH_2_-6”) 4.49 (q, *J* = 6.0 Hz, 1 H, H-5”) 4.56 (d, *J* = 3.0 Hz, 1 H, H-3”) 4.61–4.69 (m, 2 H, Hb-1 and H-2”) 5.19–5.29 (m, 1 H, H-2) 5.57 (d, *J* = 3.5 Hz, 1 H, H-1”) 6.56 (br. s, 6 H, OH) 7.21–7.29 (m, 3 H, arom. H) 7.33–7.40 (m, 2 H, arom. H) 8.30 (t, *J* = 5.4, 1 H, (CO)NH) 8.48 (d, *J* = 8.5 Hz, 1 H, NH(CO)).

^13^C NMR (75 MHz, pyridine-d_5_) δ 8.91, 14.58, 23.21, 23.45, 26.69, 27.73, 29.71, 29.80, 29.89, 29.99, 30.03, 30.61, 32.15, 32.26, 34.37, 36.47, 37.12, 37.33, 40.08, 46.18, 51.78, 63.00, 68.93, 70.67, 71.34, 71.95, 72.58, 73.40, 77.17, 101.88, 126.42, 129.09, 129.24, 143.59, 173.66.

Exact mass (ESI-MS) for C_36_H_63_N_2_O_10_ [M + H]^+^ found, 683.4477; Calcd., 683.4477; m.p. 128–130 °C.

#### (5R,6S,7S)-5,6-dihydroxy-8-O-(α-D-galactopyranosyl)-7-octanamido-N-(3-pentylphenyl)octanamide (**5f**)

^1^H-NMR (300 MHz, pyridine-d_5_) δ 0.81 (t, *J* = 6.5 Hz, 6 H, 2 x terminal CH_3_) 1.11–1.34 (m, 14 H, CH_2_) 1.55 (dt, *J* = 14.8 and 7.4 Hz, 2 H, CH_2_) 1.77 (dt, *J* = 14.8 and 7.4 Hz, 2 H, CH_2_) 1.90–2.03 (m, 1 H, CH_2_) 2.17–2.31 (m, 1 H, CH_2_) 2.41 (t, *J* = 7.6 Hz, 2 H, CH_2_) 2.55 (t, *J* = 7.6 Hz, 2 H, CH_2_) 2.68 (dd, *J* = 14.2 and 7.0 Hz, 2 H, CH_2_) 4.24–4.31 (m, 1 H, H-3) 4.31–4.45 (m, 5 H, H-4, Ha-1, H-4” and H-6”) 4.48 (q, *J* = 5.4 Hz, 1 H, H-3”) 4.55 (d, *J* = 2.4 Hz, 1 H, H-5”) 4.60–4.69 (m, 2 H, H-2” and Hb-1) 5.20–5.29 (m, 1 H, H-2) 5.57 (d, *J* = 3.4 Hz, 1 H, H-1”) 6.48 (br. s., 6 H, OH) 6.99 (d, *J* = 7.5 Hz, 1 H, arom. H) 7.32 (t, *J* = 7.7 Hz, 1 H, arom. H) 7.88 (d, *J* = 8.0 Hz, 1 H, arom. H) 7.98 (s, 1 H, arom. H) 8.47 (d, *J* = 8.7 Hz, 1 H, NH(CO)) 10.60 (s, 1 H, (CO)NH).

^13^C NMR (75 MHz, pyridine-d_5_) δ 14.51, 14.57, 23.10, 23.21, 23.41, 26.70, 29.70, 29.98, 31.76, 31.98, 32.25, 34.30, 36.57, 37.11, 38.21, 51.65, 63.02, 68.80, 70.63, 71.37, 71.93, 72.61, 73.40, 77.15, 101.82, 117.89, 120.54, 124.09, 129.41, 141.00, 144.22, 172.70, 173.69.

Exact mass (ESI-MS) for C_33_H_57_N_2_O_10_ [M + H]^+^ found, 641.4001; Calcd., 641.4008; m.p. 165–167 °C.

#### (5R,6S,7S)-5,6-dihydroxy-8-O-(α-D-galactopyranosyl)-7-octanamido-N-(4-pentylphenyl)octanamide (**5g**)

^1^H-NMR (300 MHz, pyridine-d_5_) δ 0.81 (dt, *J* = 9.1 and 7.0 Hz, 6 H, 2 x terminal CH_3_) 1.12–1.34 (m, 14 H, CH_2_) 1.55 (dt, *J* = 14.8 and 7.4 Hz, 2 H, CH_2_) 1.77 (dt, *J* = 14.9 and 7.5 Hz, 2 H, CH_2_) 1.89–2.05 (m, 1 H, CH_2_) 2.17–2.33 (m, 1 H, CH_2_) 2.42 (t, *J* = 7.8 Hz, 2 H, CH_2_) 2.53 (t, *J* = 7.8 Hz, 2 H, CH_2_) 2.61–2.71 (m, 2 H, CH_2_) 4.24–4.45 (m, 6 H, H-3, H-4, H-4”, H-6” and Ha-1) 4.49 (q, *J* = 5.9 Hz, 1 H, H-3”) 4.56 (d, *J* = 2.9 Hz, 1 H, H-5”) 4.61–4.69 (m, 2 H, Hb-1 and H-2”) 5.20–5.29 (m, 1 H, H-2) 5.57 (d, *J* = 3.7 Hz, 1 H, H-1”) 6.41 (br. s, 6 H, OH) 7.23 (app. s, 2 H, arom. H) 8.01 (d, *J* = 8.4 Hz, 2 H, arom. H) 8.48 (d, *J* = 8.7 Hz, 1 H, NH(CO)) 10.63 (s, 1 H, (CO)NH).

^13^C NMR (75 MHz, pyridine-d_5_) δ 14.55, 14.58, 23.14, 23.22, 23.42, 26.70, 29.71, 29.99, 31.95, 32.26, 34.31, 35.86, 37.13, 38.14, 51.66, 63.04, 68.81, 70.66, 71.38, 71.95, 72.62, 73.42, 77.18, 101.85, 120.56, 129.43, 138.26, 138.75, 172.58, 173.68.

Exact mass (ESI-MS) for C_33_H_57_N_2_O_10_ [M + H]^+^ found, 641.4011; Calcd., 641.4008; m.p. 147–149 °C.

#### (5R,6S,7S)-5,6-dihydroxy-8-O-(α-D-galactopyranosyl)-7-octanamido-N-(2-(2-ethoxyethoxy)ethyl)octanamide (**5h**)

^1^H-NMR (300 MHz, pyridine-d_5_) δ 0.80 (t, *J* = 6.5 Hz, 3 H, terminal CH_3_) 1.08–1.22 (m, 8 H, terminal CH_3_ and CH_2_) 1.23–1.41 (m, 5 H, CH_2_) 1.76 (dt, *J* = 14.8 and 7.4 Hz, 2 H, CH_2_) 1.87–2.01 (m, 1 H, CH_2_) 2.11–2.24 (m, 1 H, CH_2_) 2.31–2.47 (m, 4 H, CH_2_) 2.47–2.58 (m, 2 H, CH_2_) 3.41 (q, *J* = 7.0 Hz, 2 H, CH_2_) 3.49–3.54 (m, 2 H, CH_2_) 3.56–3.61 (m, 2 H, CH_2_) 3.62–3.70 (m, 2 H, CH_2_) 4.22–4.44 (m, 6 H, Ha-1, H-3, H-4, H-4” and CH_2_-6”) 4.48 (q, *J* = 5.8 Hz, 1 H, H-5”) 4.55 (d, *J* = 2.7 Hz, 1 H, H-3”) 4.60–4.69 (m, 2 H, Hb-1 an H-2”d) 5.19–5.29 (m, 1 H, H-2) 5.56 (d, *J* = 3.7 Hz, 1 H, H-1”) 6.43 (br. s, 6 H, OH) 8.43 (d, *J* = 8.4 Hz, 1 H, NH(CO)) 8.47–8.52 (m, 1 H, (CO)NH).

^13^C NMR (75 MHz, pyridine-d_5_) δ 14.59, 15.84, 23.23, 23.39, 26.71, 29.72, 30.00, 30.37, 32.27, 34.46, 37.14, 37.21, 40.14, 51.74, 63.03, 66.84, 68.93, 70.50, 70.67, 70.85, 71.01, 71.38, 71.96, 72.64, 73.42, 77.21, 101.90, 173.64, 173.86.

Exact mass (ESI-MS) for C_28_H_55_N_2_O_12_ [M + H]^+^ found, 611.3750; Calcd., 611.3750; m.p. could not be determined due to hygroscopy.

#### (5R,6S,7S)-5,6-dihydroxy-8-O-(α-D-galactopyranosyl)-7-undecanamino-N-(6-phenylhexyl)octanamide (**6d**)

^1^H-NMR (300 MHz, pyridine-d_5_) δ 0.87 (t, *J* = 6.6 Hz, 3 H, terminal CH_3_) 1.13–1.41 (m, 20 H, CH_2_) 1.45–1.61 (m, 4 H, CH_2_) 1.73–1.87 (m, 2 H, CH_2_) 1.91–2.05 (m, 1 H, CH_2_) 2.18–2.60 (m, 9 H, CH_2_) 3.43 (q, *J* = 6.8 Hz, 2 H, NHCH_2_) 4.26–4.53 (m, 7 H, Ha-1, H-3, H-4, H-4”, H-5” and CH_2_-6”) 4.56 (d, *J* = 3.1 Hz, 1 H, H-3”) 4.61–4.71 (m, 2 H, Hb-1 and H-2”) 5.21–5.31 (m, 1 H, H-2) 5.57 (d, *J* = 3.8 Hz, 1 H, H-1”) 5.96 (br. s, 6 H, OH) 7.17–7.27 (m, 3 H, arom. H) 7.31–7.38 (m, 2 H, arom. H) 8.27 (t, *J* = 5.8 Hz, 1 H, (CO)NH) 8.48 (d, *J* = 8.7 Hz, 1 H, NH(CO)).

^13^C NMR (75 MHz, pyridine-d_5_) δ 14.69, 23.33, 23.47, 26.77, 27.59, 29.62, 29.99, 30.14, 30.20, 30.26, 30.32, 30.55, 32.11, 32.51, 34.41, 36.43, 37.18, 37.34, 40.08, 51.77, 63.05, 68.96, 70.70, 71.40, 71.98, 72.62, 73.45, 77.22, 101.93, 126.45, 129.10, 129.24, 143.53, 173.68, 173.72.

Exact mass (ESI-MS) for C_38_H_67_N_2_O_10_ [M + H]^+^ found, 711.4781; Calcd., 711.4790; m.p. 91–93 °C.

#### (5R,6S,7S)-5,6-dihydroxy-8-O-(α-D-galactopyranosyl)-7-pentadecanamino-N-(6-phenylhexyl)octanamide (**7d**)

^1^H-NMR (300 MHz, pyridine-d_5_) δ 0.88 (t, *J* = 6.7 Hz, 3 H, terminal CH_3_) 1.16–1.41 (m, 28 H, CH_2_) 1.45–1.61 (m, 4 H, CH_2_) 1.74–1.87 (m, 2 H, CH_2_) 1.91–2.04 (m, 1 H, CH_2_) 2.16–2.60 (m, 9 H, CH_2_) 3.43 (q, *J* = 6.7 Hz, 2 H, NHCH_2_) 4.26–4.53 (m, 7 H, Ha-1, H-3, H-4, H-4”, H-5” and CH_2_-6”) 4.56 (d, *J* = 3.2 Hz, 1 H, H-3”) 4.61–4.71 (m, 2 H, Hb-1 and H-2”) 5.21–5.30 (m, 1 H, H-2) 5.57 (d, *J* = 3.9 Hz, 1 H, H-1”) 5.93 (br. s, 6 H, OH) 7.19–7.30 (m, 3 H, arom. H) 7.30–7.38 (m, 2 H, arom. H) 8.27 (t, *J* = 5.6 Hz, 1 H, (CO)NH) 8.47 (d, *J* = 8.6 Hz, 1 H, NH(CO)).

^13^C NMR (75 MHz, pyridine-d_5_) δ 14.69, 23.35, 23.47, 26.77, 27.59, 29.60, 30.02, 30.15, 30.18, 30.25, 30.34, 30.40, 30.57, 32.11, 32.52, 34.43, 36.43, 37.18, 37.34, 40.06, 51.77, 63.07, 68.99, 70.70, 71.40, 71.98, 72.64, 73.46, 77.25, 101.95, 126.43, 129.10, 129.24, 143.53, 173.61, 173.68.

Exact mass (ESI-MS) for C_42_H_75_N_2_O_10_ [M + H]^+^ found, 767.5412; Calcd., 767.5416; m.p. 148–150 °C.

#### (5R,6S,7S)-5,6-dihydroxy-8-O-(α-D-galactopyranosyl)-7-nonadecanamino-N-(6-phenylhexyl)octanamide (**8d**)

^1^H-NMR (300 MHz, pyridine-d_5_) δ 0.88 (t, *J* = 6.6 Hz, 3 H, terminal CH_3_) 1.14–1.40 (m, 36 H, CH_2_) 1.45–1.61 (m, 4 H, CH_2_) 1.70–1.88 (m, 2 H, CH_2_) 1.90–2.05 (m, 1 H, CH_2_) 2.18–2.58 (m, 9 H, CH_2_) 3.44 (q, *J* = 6.7 Hz, 2 H, NHCH_2_) 4.23–4.54 (m, 7 H, Ha-1, H-3, H-4, H-4”, H-5” and CH_2_-6”) 4.57 (d, *J* = 3.1 Hz, 1 H, H-3”) 4.61–4.72 (m, 2 H, Hb-1 and H-2”) 5.21–5.31 (m, 1 H, H-2) 5.57 (d, *J* = 3.8 Hz, 1 H, H-1”) 5.83 (br. s, 6 H, OH) 7.20–7.27 (m, 3 H, arom. H) 7.31–7.38 (m, 2 H, arom. H) 8.28 (t, *J* = 5.6 Hz, 1 H, (CO)NH) 8.49 (d, *J* = 9.0 Hz, 1 H, NH(CO)).

^13^C NMR (75 MHz, pyridine-d_5_) δ 14.69, 23.35, 23.47, 26.78, 27.61, 29.62, 30.02, 30.20, 30.26, 30.32, 30.43, 30.55, 32.11, 32.52, 34.41, 36.43, 37.19, 37.34, 40.08, 51.77, 63.07, 68.96, 70.68, 71.40, 71.97, 72.64, 73.45, 77.22, 101.93, 126.43, 129.10, 129.24, 143.53, 173.66, 173.71.

Exact mass (ESI-MS) for C_46_H_83_N_2_O_10_ [M + H]^+^ found, 823.6036; Calcd., 823.6042; m.p. 136–138 °C.

#### (5R,6S,7S)-5,6-dihydroxy-8-O-(α-D-galactopyranosyl)-7-hexacosylamino-N-(6-phenylhexyl)octanamide (**9d**)

^1^H-NMR (300 MHz, pyridine-d_5_) δ 0.88 (t, *J* = 6.4 Hz, 3 H, terminal CH_3_) 1.11–1.43 (m, 42 H, CH_2_) 1.43–1.62 (m, 6 H, CH_2_) 1.81 (quint, *J* = 7.3 Hz, 2 H, CH_2_) 1.91–2.07 (m, 2 H, CH_2_) 2.12–2.30 (m, 2 H, CH_2_) 2.34–2.61 (m, 8 H, CH_2_) 3.03 (q, *J* = 7.4 Hz, 2 H, CH_2_) 3.43 (q, *J* = 6.6 Hz, 2 H, NHCH_2_) 4.26–4.45 (m, 6 H, Ha-1, H-3, H-4, H-4” and CH_2_-6”) 4.49 (q, *J* = 5.9 Hz, 1 H, H-5”) 4.56 (d, *J* = 2.9 Hz, 1 H, H-3”) 4.60–4.70 (m, 2 H, Hb-1 and H-2”) 5.19–5.30 (m, 1 H, H-2) 5.57 (d, *J* = 3.8 Hz, 1 H, H-1”) 6.33 (br. s, 6 H, OH) 7.17–7.27 (m, 3 H, arom. H) 7.30–7.39 (m, 2 H, arom. H) 8.29 (t, *J* = 5.4 Hz, 1 H, (CO)NH) 8.49 (d, *J* = 8.5 Hz, 1 H, NH(CO)).

^13^C NMR (75 MHz, pyridine-d_5_) δ 8.89, 14.66, 23.32, 23.45, 26.75, 27.57, 29.59, 29.99, 30.14, 30.17, 30.25, 30.29, 30.37, 30.41, 30.52, 32.08, 32.49, 34.37, 36.40, 37.15, 37.31, 40.05, 46.15, 51.78, 63.00, 68.91, 70.67, 71.32, 71.95, 72.55, 73.40, 77.17, 101.87, 126.40, 129.07, 129.21, 143.50, 173.66, 173.69.

Exact mass (ESI-MS) for C_52_H_95_N_2_O_10_ [M + H]^+^ found, 907.6976; Calcd., 907.6981; m.p. 116–118 °C.

#### (5R,6S,7S)-5,6-dihydroxy-8-O-(α-D-galactopyranosyl)-7-hexacosylamino-N-(3-pentylphenyl)octanamide (**9f**)

^1^H-NMR (300 MHz, pyridine-d_5_) δ 0.80 (t, *J* = 6.9 Hz, 3 H, terminal CH_3_) 0.88 (t, *J* = 6.5 Hz, 3 H, terminal CH_3_) 1.14–1.47 (m, 48 H, CH_2_) 1.47–1.64 (m, 2 H, CH_2_) 1.74–1.89 (m, 2 H, CH_2_) 1.89–2.16 (m, 1 H, CH_2_) 2.16–2.33 (m, 1 H, CH_2_) 2.33–2.51 (m, 4 H, CH_2_) 2.51–2.60 (m, 2 H, CH_2_) 2.60–2.74 (m, 2 H, CH_2_) 4.23–4.46 (m, 6 H, Ha-1, H-3”, H-4, H-4” and CH_2_-6”) 4.46–4.53 (m, 1 H, H-3) 4.56 (br. s, 1 H, H-5”) 4.60–4.71 (m, 2 H, Hb-1 and H-2”) 5.20–5.31 (m, 1 H, H-2) 5.57 (d, *J* = 3.8 Hz, 1 H, H-1”) 6.27 (d, *J* = 6.1 Hz, 1 H, OH) 6.32 (d, *J* = 3.7 Hz, 1 H, OH) 6.46–6.58 (m, 2 H, OH) 6.72 (br. s, 1 H, OH) 6.97–7.08 (m, 2 H, arom. H and OH) 7.33 (t, *J* = 7.8 Hz, 1 H, arom. H) 7.88 (d, *J* = 7.9 Hz, 1 H, arom. H) 7.97 (s, 1 H, arom. H) 8.46 (d, *J* = 8.5 Hz, 1 H, NH(CO)) 10.59 (s, 1 H, (CO)NH).

^13^C NMR (75 MHz, pyridine-d_5_) δ 14.54, 14.69, 23.13, 23.35, 23.44, 26.78, 30.02, 30.17, 30.20, 30.26, 30.32, 30.40, 30.44, 31.80, 32.02, 32.52, 34.35, 36.61, 37.18, 38.24, 51.68, 63.07, 68.87, 70.68, 71.42, 71.98, 72.65, 73.46, 77.22, 101.88, 117.91, 120.55, 124.11, 129.45, 141.05, 144.26, 172.71, 173.68.

Exact mass (ESI-MS) for C_51_H_93_N_2_O_10_ [M + H]^+^ found, 893.6830; Calcd., 893.6825; m.p. 127–129 °C.

#### N-((2S,3S,4R)-1-O-(α-D-galactopyranosyl)-3,4-dihydroxy-16-phenylhexadecan-2-yl)octanamide(**23**)

Compound **23** was synthesized in an analogous way as described in scheme 2 and 3. A Wittig olefination between **14** and triphenyl(10-decylphenyl)phosphonium bromide was followed by selective reduction of the double bond. The azide moiety on C2 was installed using a Mitsunobu inversion with hydrazoic acid (HN_3_). Next, the primary hydroxyl group was selectively deprotected to give the corresponding acceptor, which was glycosylated with trichloroacetimidate donor **11**. Staudinger reduction of the azide, followed by amide formation with octanoic acid, yielded, after overall deprotection, compound **23**.

^1^H-NMR (300 MHz, pyridine-d_5_) δ 0.81 (t, *J* = 6.3 Hz, 3 H, terminal CH_3_) 1.11–2.01 (m, 32 H, CH_2_) 2.44 (t, *J* = 7.6 Hz, 2 H, CH_2_) 2.60 (t, *J* = 7.6 Hz, 2 H, CH_2_) 4.31–4.35 (m, 2 H, H-2” and H3) 4.38–4.46 (m, 3 H, Hb1 and CH_2_-6”) 4.47 (m, 1 H, H-4) 4.53 (m, 1 H, H-4”) 4.57 (m, 1 H, H-5”) 4.63–4.72 (m, 2 H, Ha1 and H-3”) 5.24–5.34 (m, 1 H, H-2) 5.60 (d, *J* = 3.7 Hz, 1 H, H-1”) 5.94 (br. s, 6 H, OH) 7.22–7.29 (m, 3 H, arom. H) 7.31–7.39 (m, 2 H, arom. H) 8.49 (d, *J* = 8.6 Hz, 1 H, NH).

^13^C NMR (75 MHz, pyridine-d_5_) δ 14.23, 22.89, 26.37, 26.50, 29.38, 29.56, 29.66, 29.82, 29.98, 30.01, 30.14, 30.38, 31.89, 34.42, 36.11, 36.77, 51.64, 62.68, 68.65, 70.30, 71.03, 71.67, 71.71, 72.50, 73.05, 76.72, 101.45, 128.73, 128.88.

#### Recombinant proteins, SPR studies and crystallography

Soluble mCD1-β2 M protein was expressed and purified from insect cells as described^41^. Glycolipid loading and SPR studies were performed as previously reported^27^. In brief, increasing concentrations of TCR (two-fold dilutions from 500 nM to 15.625 nM) were passed over streptavidin immobilized *CD1d* using a CAP chip on a Biacore T200. A reference substraction included the TCR binding response to CD1d to which no lipid has been added. In addition, single cycle kinetics were carried out with 3-fold dilutions of TCR (900nM-11nM), without reference substraction. Running buffer contained no detergent in an attempt to not extract these short-acyl chain ligands during the course of the experiment. CD1d-ligand-TCR complex formation, purification, crystallization and structure determination was performed as reported previously^19, 25^. Data collection and refinement statistics are shown in Table 2. Structure of mCD1d-**5d** and mCD1d-**23** have been deposited in the Protein Data Bank (http://www.rcsb.org/) under accession codes 5TW2 and 5TW5, respectively.

**Table 2:** Data collection and refinement statistics of crystal structures of the CD1d-**23** and CD1d-**5d** complexes.

Data collection statistics	CD1d-23	CD1d-5d
Space group	P212121	P212121
Cell dimension		
*a, b, c*, (Å)	42.2, 106.2, 106.9	42.2, 107.5, 109.6
a, b, g (°)	90, 90, 90	90, 90, 90
Resolution range (Å) [outer shell]	45–1.85 [1.92–1.85]	40–1.75 [1.81–1.75]
No. reflections	40,100	51,068
R_meas_ (%)	13.3 [97.9]	6.9 [58.1]
R_pim_ (%)	5.5 [41.3]	3.3 [27.5]
Multiplicity	5.4 [5.2]	4.3 [4.3]
Average I/sI	17.1 [2.5]	35.8 [3.1]
Completeness (%)	95.2 [98.6]	99.7 [100.0]
**Refinement statistics**		
No. atoms	3,435	3,385
Protein	2,992	2,929
Ligand (spacer/antigen)	18/46	18/46
Carbohydrate	56	52
Water	323	340
R/R_free_ (%)	21.4/24.3	20.3/22.1
Ramachandran plot (%)		
Favored	97.5	98.3
Allowed	100	100
R.m.s. deviations		
Bonds (Å)	0.008	0.008
Angles (°)	1.37	1.33
B-factors (Å^2^)		
Protein	24.4	31.8
Spacer/Lipid	40.8/29.4	52.5/60.7
Carbohydrate	44.7	46.8
Water	31.0	39.7

#### *In vivo* cytokine secretion assay

Experiments were approved by and conducted according to the guidelines of the Ethical Committee of Laboratory Animal Welfare of Ghent University.

The different glycolipids were dissolved in DMSO at a concentration of 1 mg/mL, heated for 20 minutes at 80 °C and subsequently sonicated for 10 minutes at the same temperature. Next, 11 µL of the prepared solution was diluted with PBS in order to obtain a 10 µg/mL solution and the resulting solution was again warmed to 80 °C for 20 minutes followed by sonication for 10 minutes at this temperature. Then, 500 µL (5 µg) was injected i.p. in 8 different C57BL/6 mice and blood was collected 4 h (IL-4) and 16 h (IFN-γ) post injection. The levels of IL-4 and INF-γ were determined by ELISA (eBioscience). KRN7000 was employed as control.

## Electronic supplementary material

Supplementary Information
