# Supplementary material for: Galactosylsphingamides: new α-GalCer analogues to probe the F’-pocket of CD1d
Source: Sci Rep. 2017 Jun 27;7:4276. doi: 10.1038/s41598-017-04461-7 (PMC5487351; doi:10.1038/s41598-017-04461-7)
Supplement: Supplementary file 1 — Supplementary Information [file 41598_2017_4461_MOESM1_ESM.pdf]

Supporting information:

Galactosylsphingamides: new α-GalCer analogues to probe the F'-pocket of CD1d

Joren Guillaume, Jing Wang, Jonas Janssens, Soumya G. Remesh, Martijn D. P. Risseeuw, Tine Decruy, Mathy Froeyen, Dirk Elewaut, Dirk M. Zajonc and Serge Van Calenbergh

Content

1) ^{1}H and ^{13}C spectra of all synthesized molecules....... S1-72

2) Purity assessment of the final compounds S73-93

3) Supporting figure S1....... S94

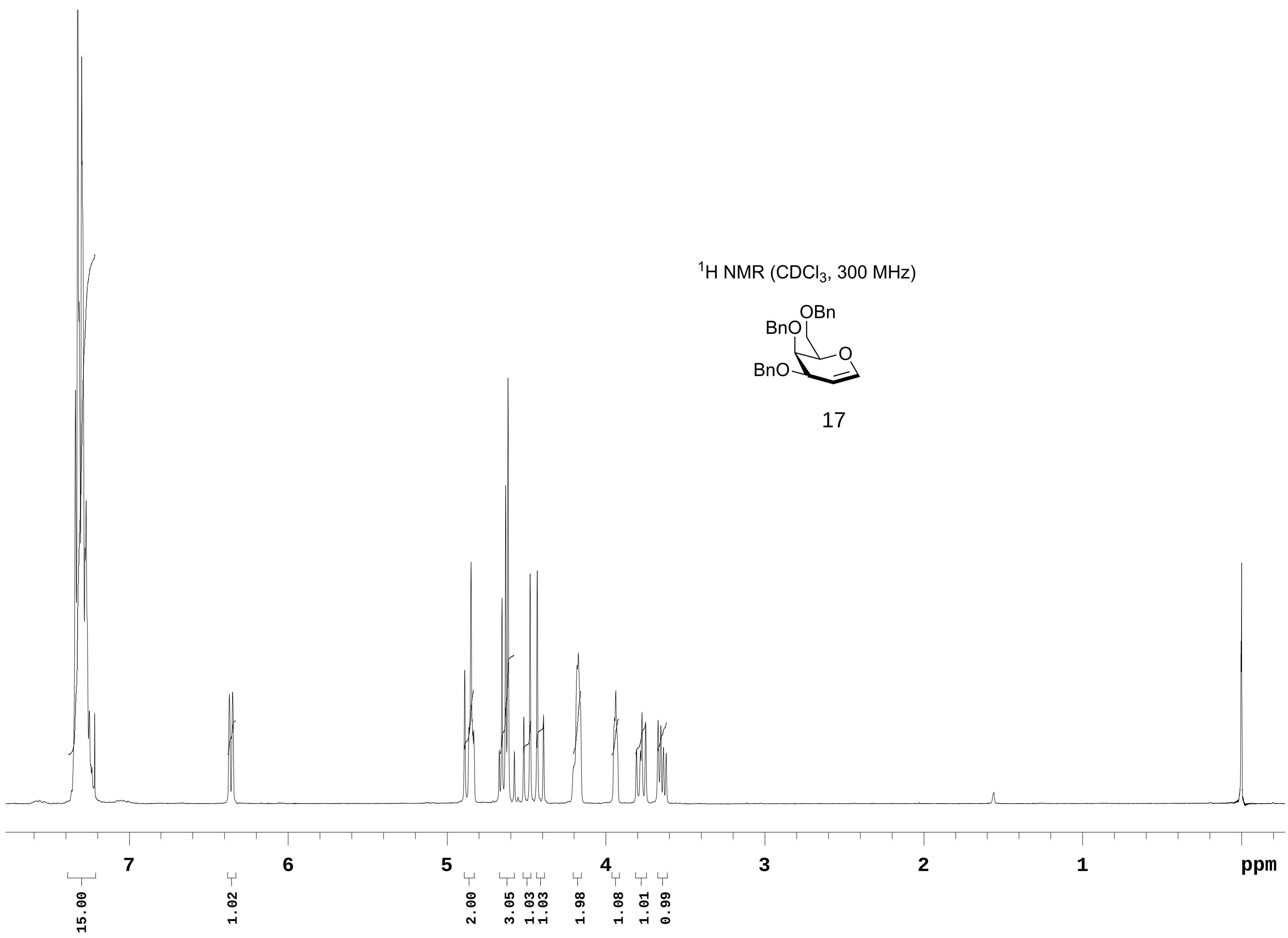

^{1}H NMR ($CDCl_3$, 300 MHz)
OBn
BnO
O
BnO
17
7
6
5
4
3
2
1
ppm
15.00
1.02
2.00
3.05
1.03
1.03
1.98
1.08
1.01
0.99

^{13}C NMR ($CDCl_3$, 75 MHz)

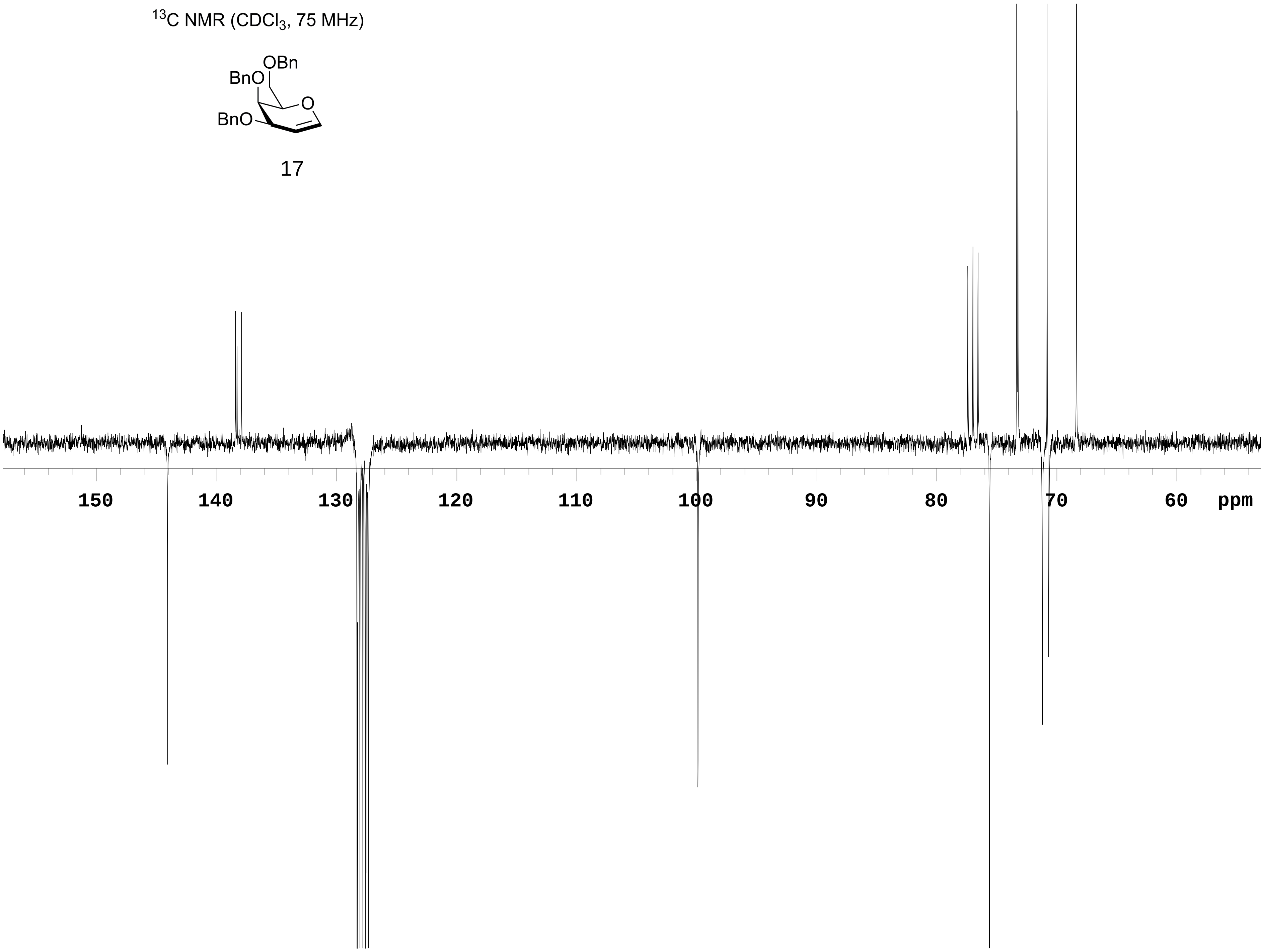

17

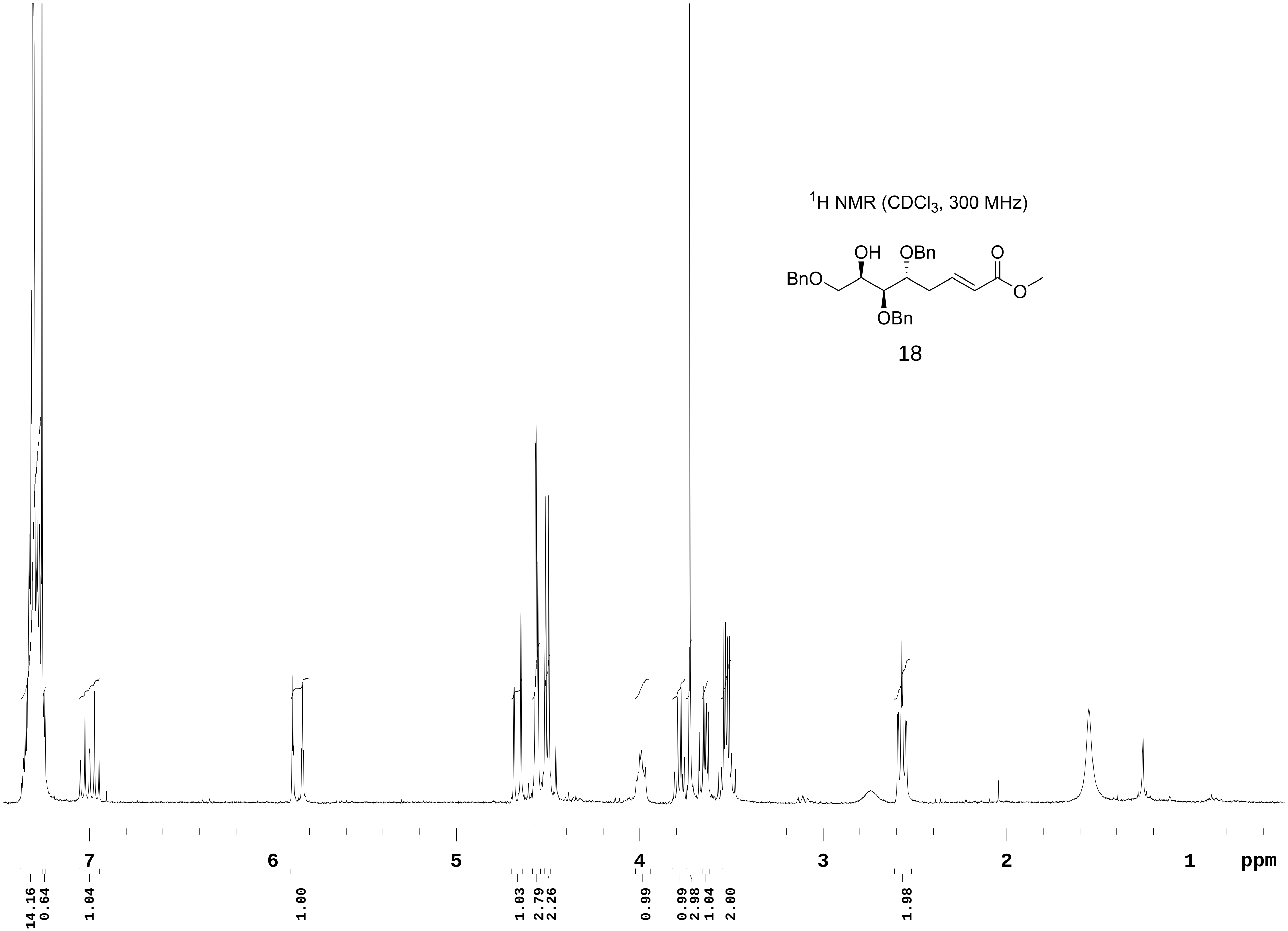

^{1}H NMR ($CDCl_3$, 300 MHz)
OH
OBn
O
BnO
O
OBn
18
7
6
5
4
3
2
1
ppm
14.16
0.64
1.04
1.00
1.03
2.79
2.26
0.99
0.99
2.98
1.04
2.00
1.98

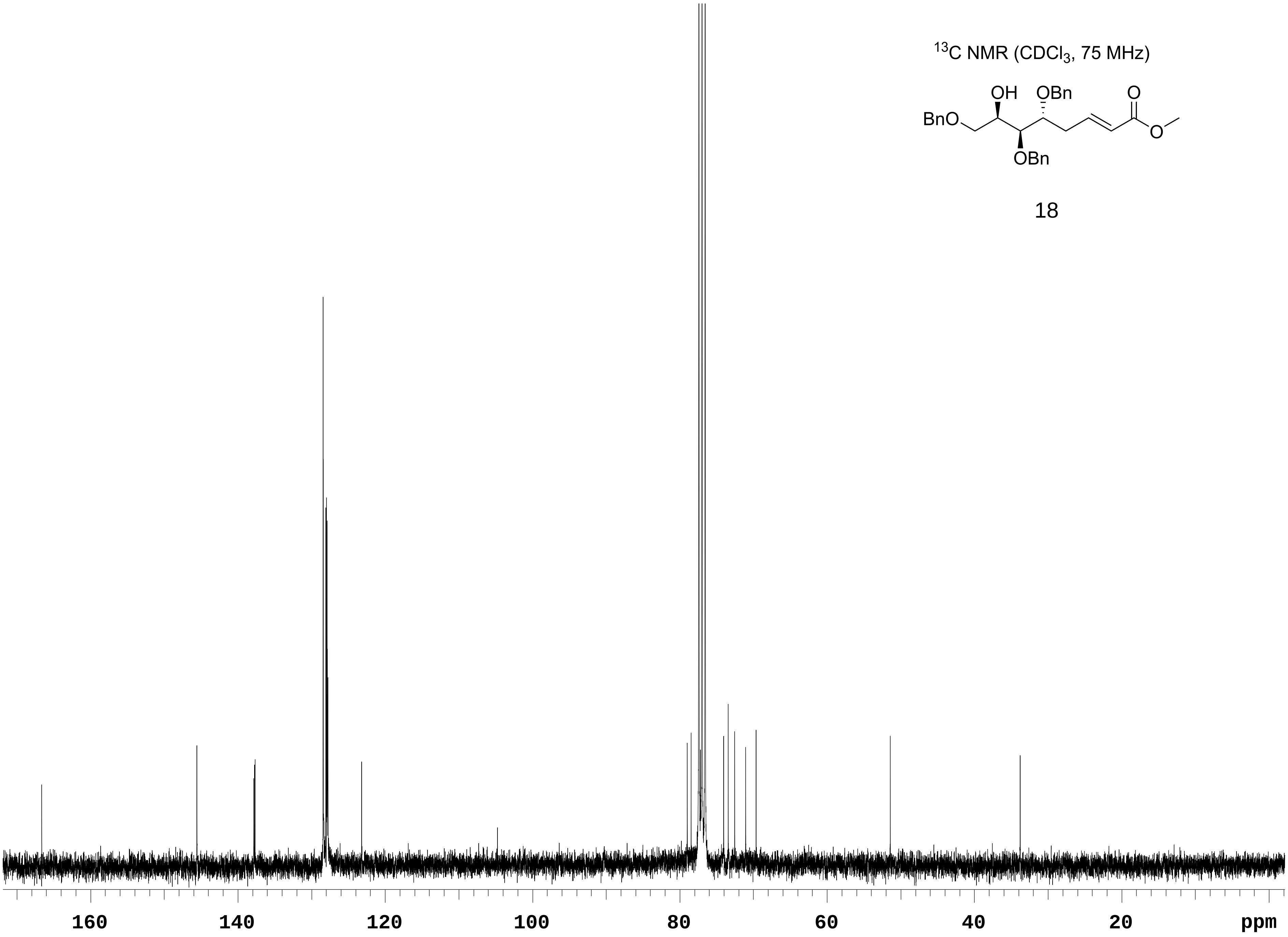

13C NMR (CDCl3, 75 MHz)
OH
OBn
BnO
O
O
OBn
18
160
140
120
100
80
60
40
20
ppm

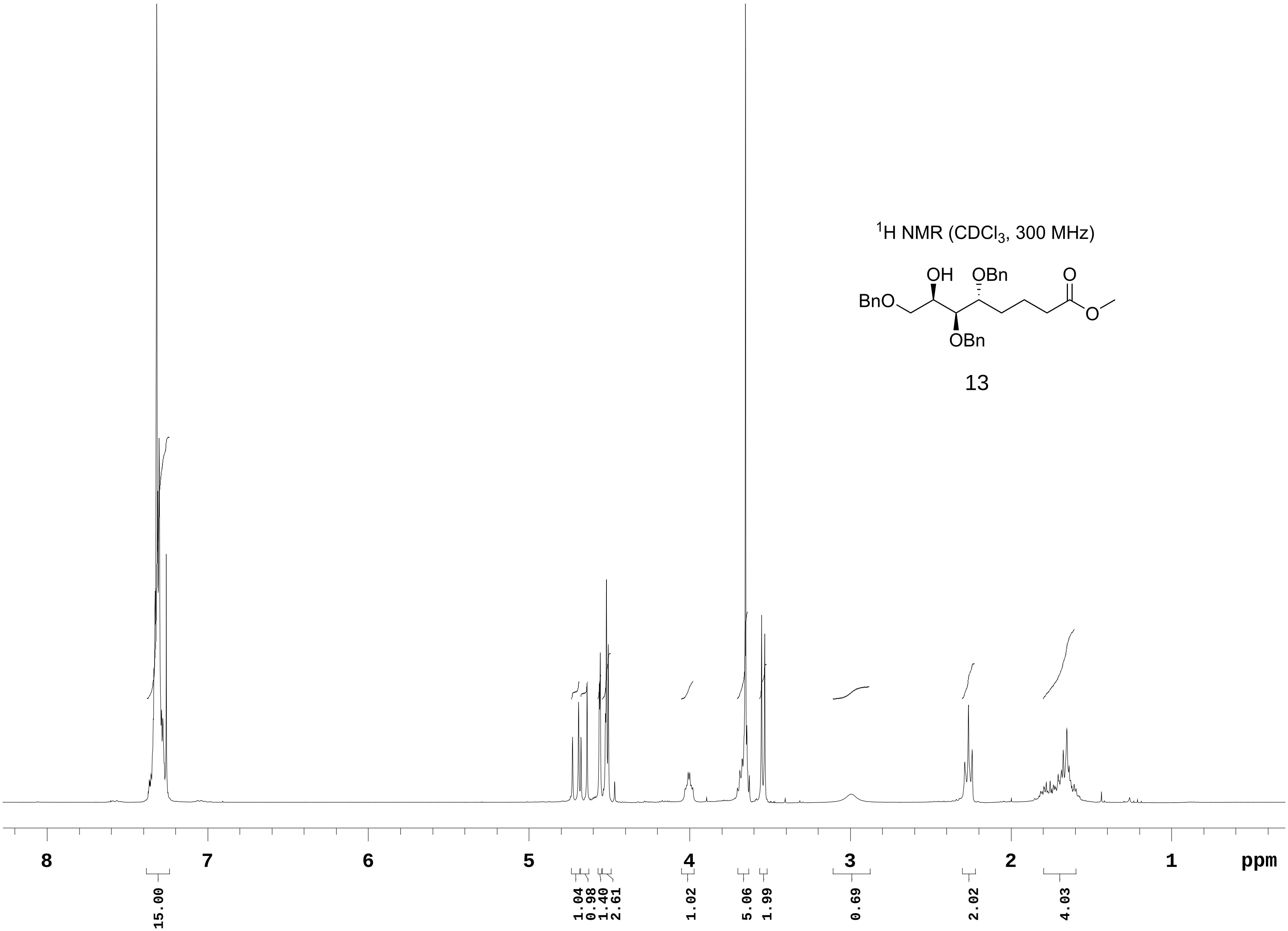

^{1}H NMR ($CDCl_3$, 300 MHz)
BnO
OH
OBn
O
O
OBn
13
8
7
6
5
4
3
2
1
ppm
15.00
1.04
0.98
1.40
2.61
1.02
5.06
1.99
0.69
2.02
4.03

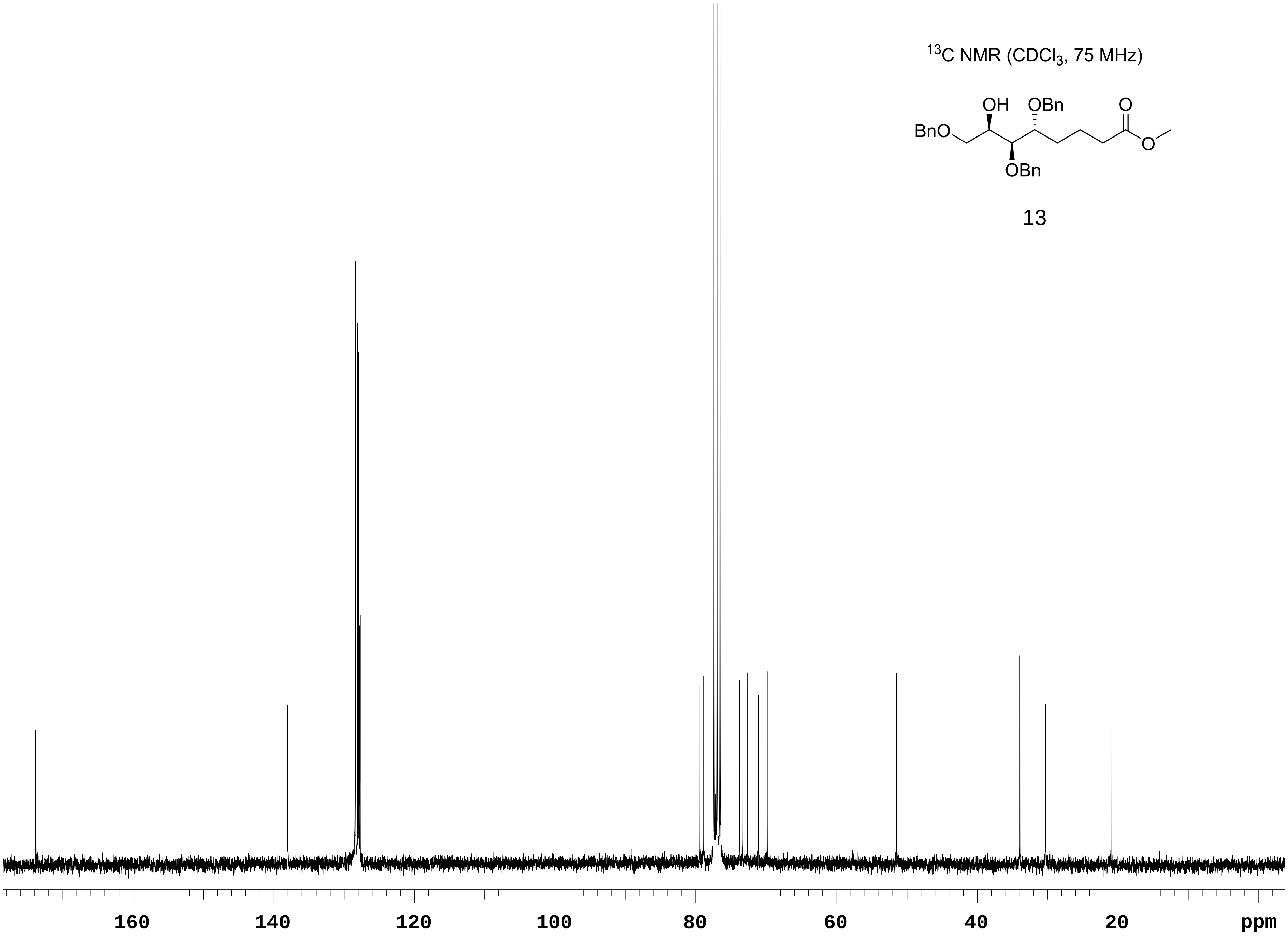

^{13}C NMR (CDCl$_3$, 75 MHz)
OH
OBn
O
BnO
O
OBn
13
160
140
120
100
80
60
40
20
ppm

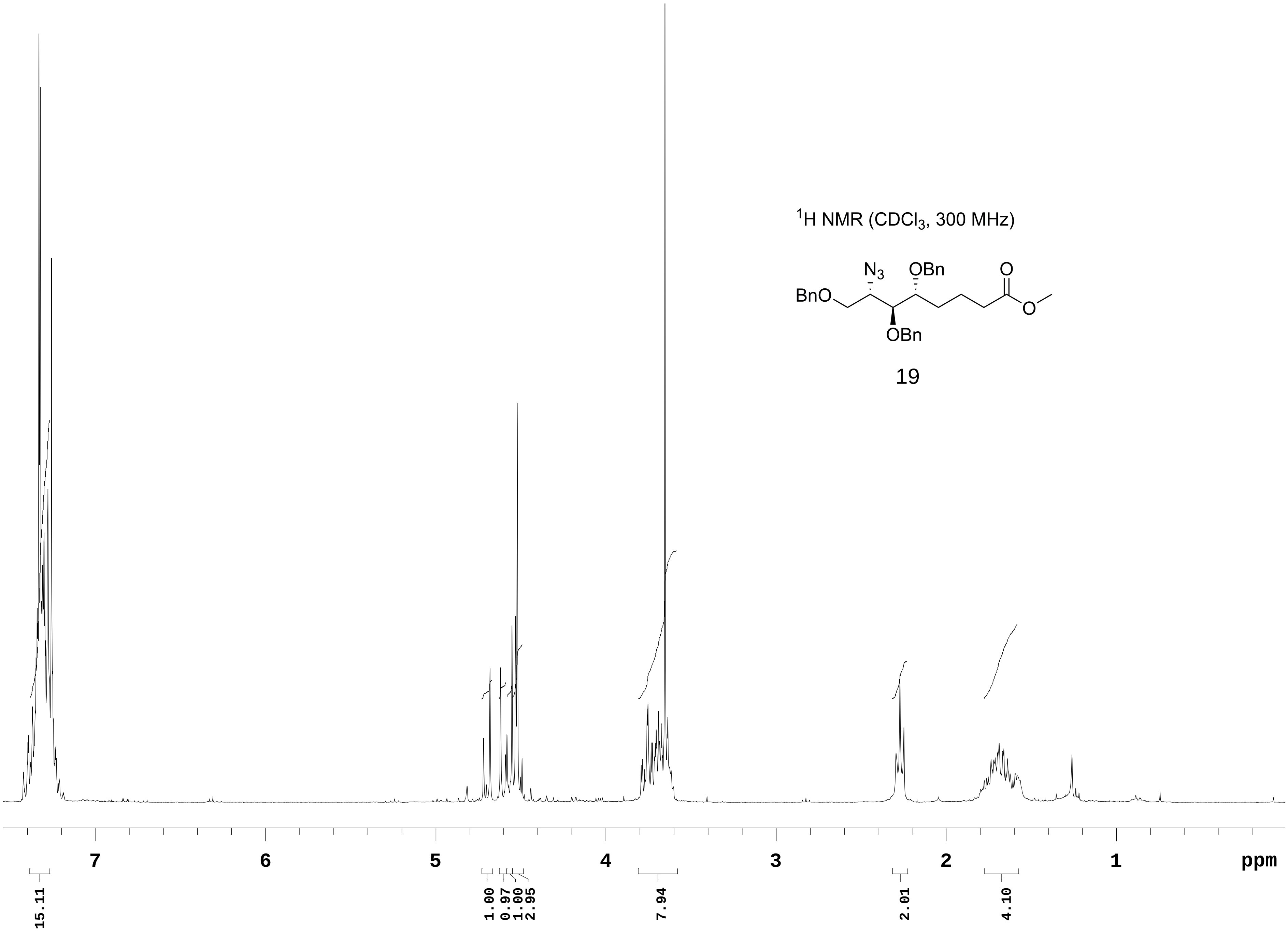
^{1}H NMR ($CDCl_3$, 300 MHz)
N3
OBn
O
BnO
O
OBn
19
7
6
5
4
3
2
1
ppm
15.11
1.00
0.97
1.00
2.95
7.94
2.01
4.10

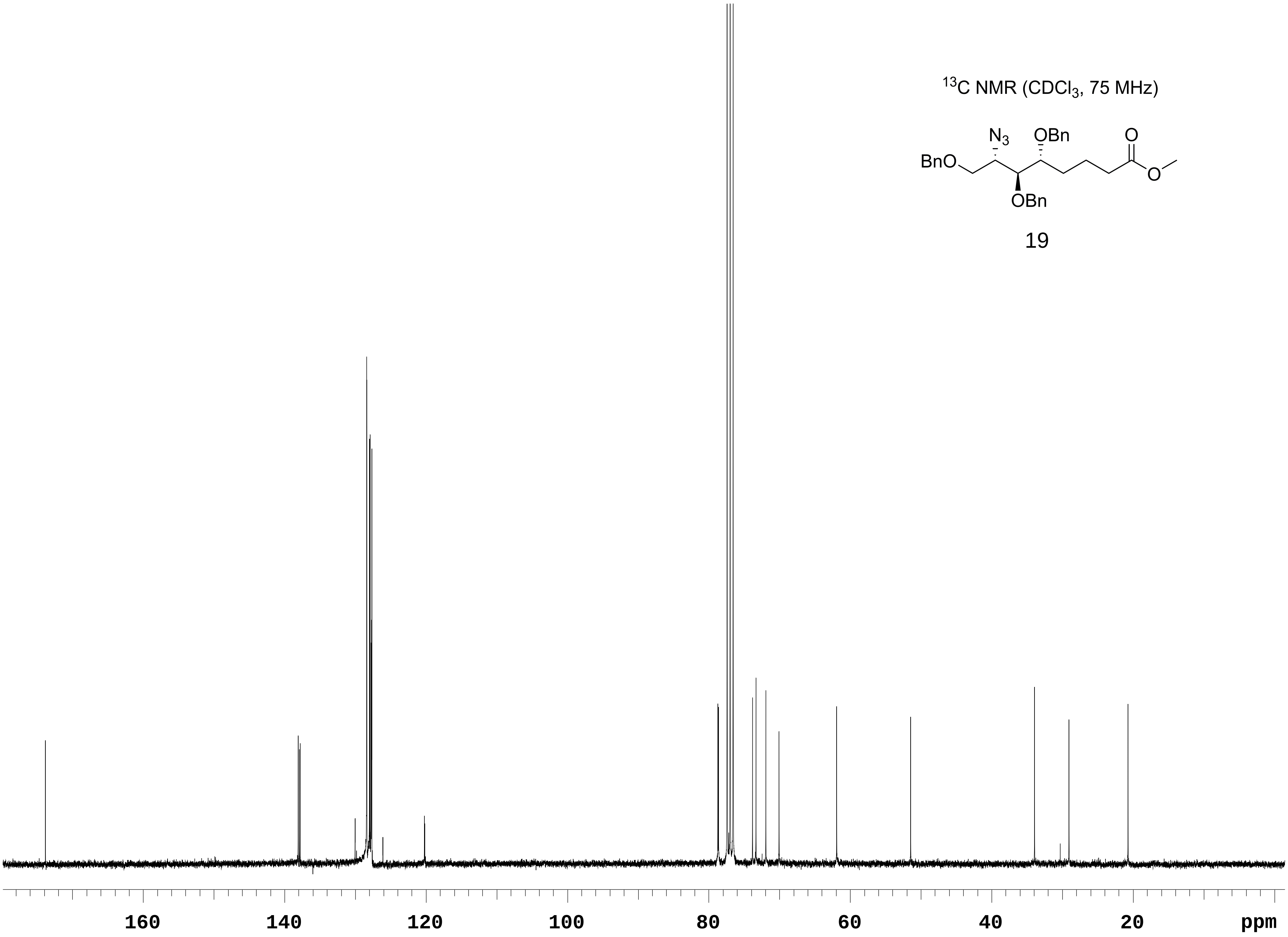

^{13}C NMR ($CDCl_3$, 75 MHz)
N_3
OBn
O
BnO
O
OBn
19
160
140
120
100
80
60
40
20
ppm

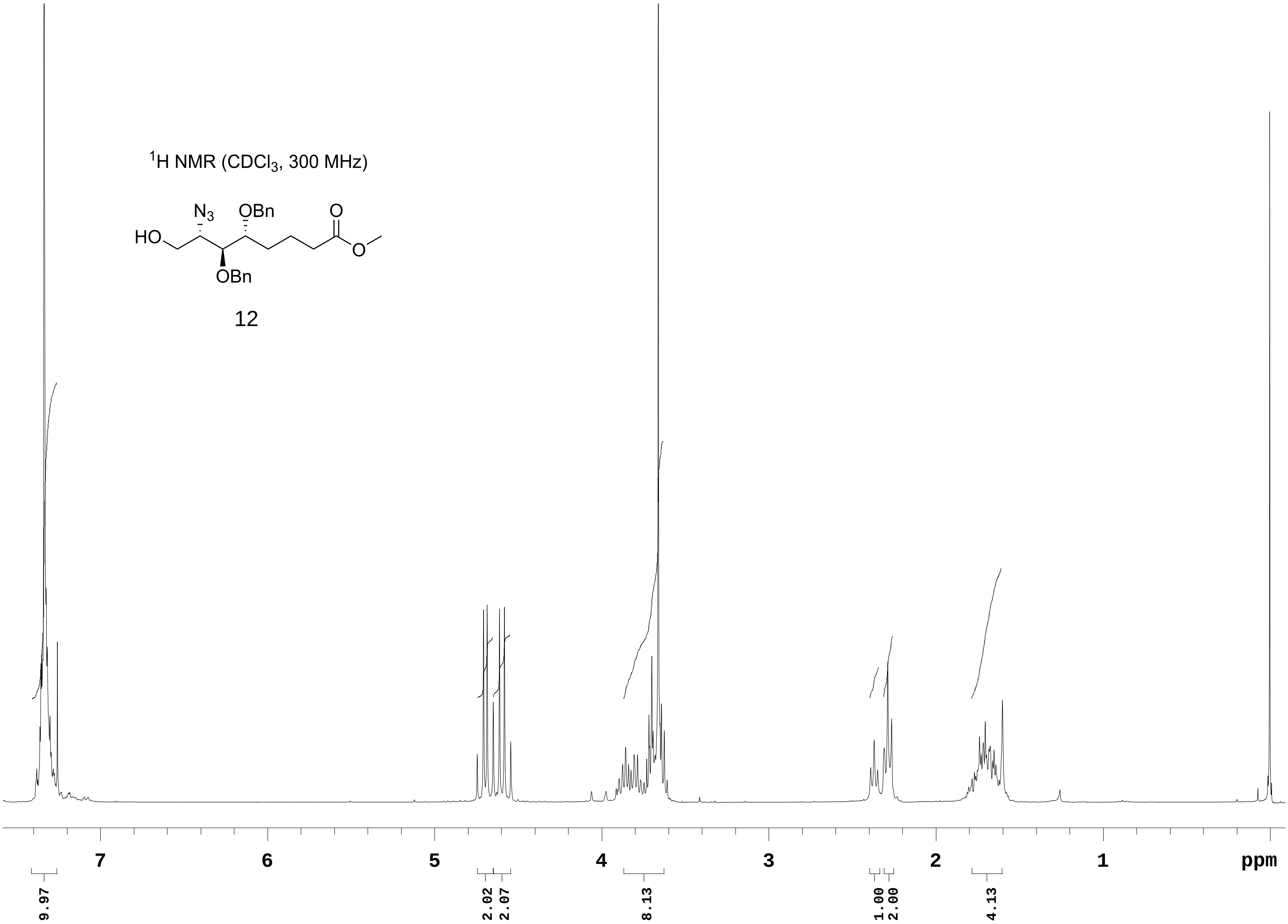

^1H NMR ($CDCl_3$, 300 MHz)
HO
N_3
OBn
OBn
O
O
12
7
6
5
4
3
2
1
ppm
9.97
2.02
2.07
8.13
1.00
2.00
4.13

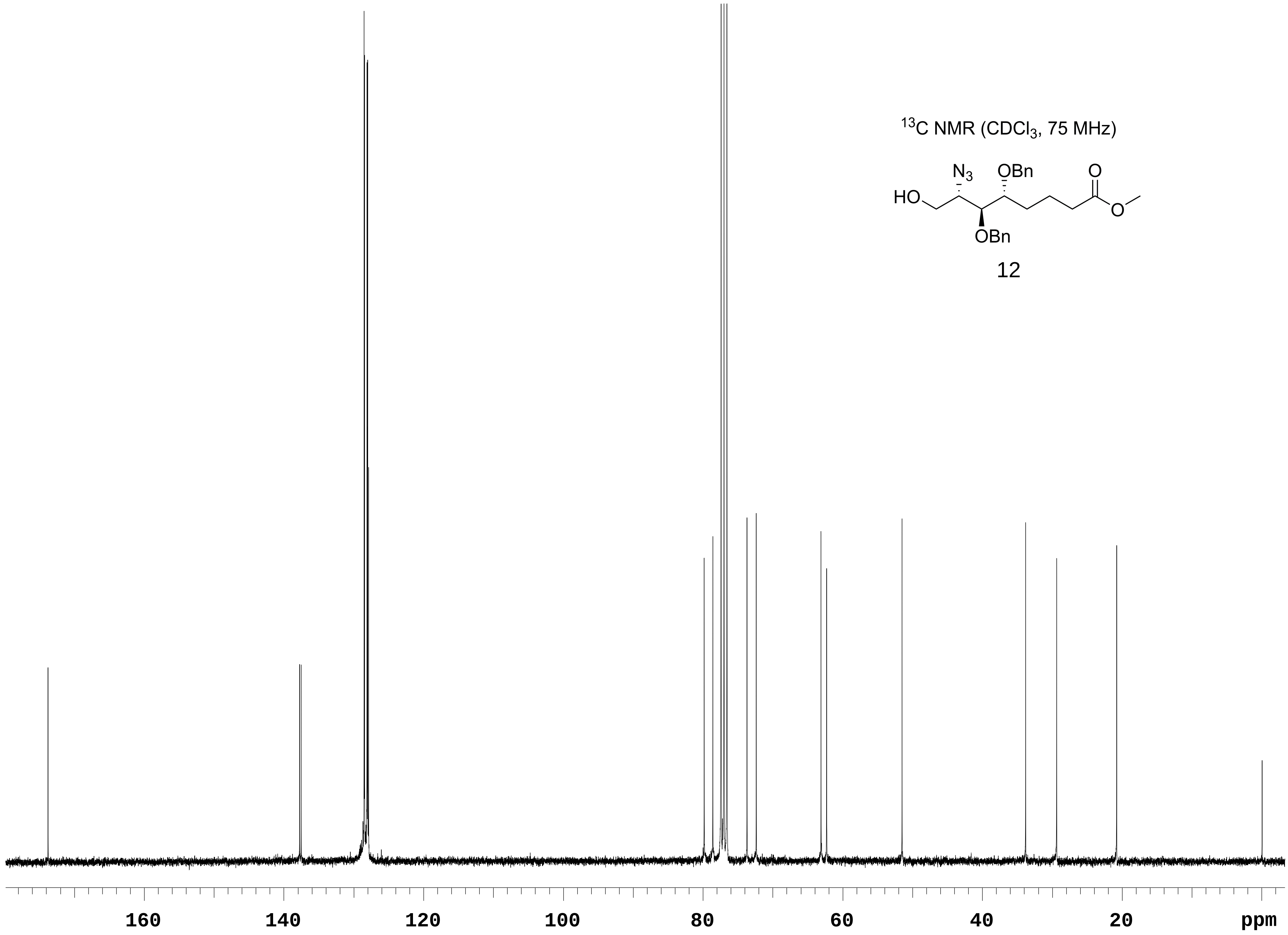

13C NMR (CDCl3, 75 MHz)
N3
OBn
O
HO
O
OBn
12
160
140
120
100
80
60
40
20
ppm

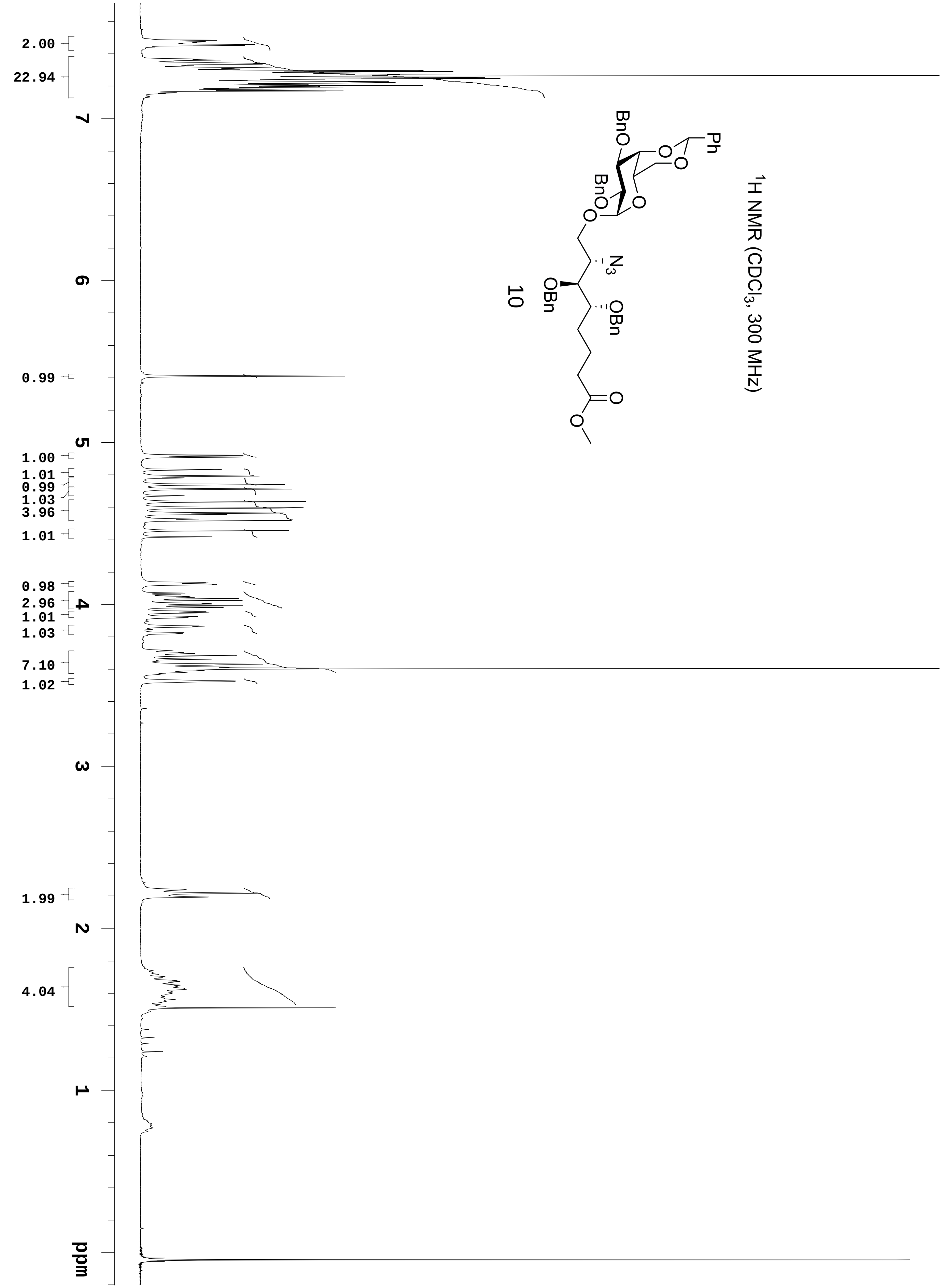

1H NMR (CDCl3, 300 MHz)
Ph
BnO
BnO
N3
OBn
OBn
O
10
2.00
22.94
0.99
1.00
1.01
0.99
1.03
3.96
1.01
0.98
2.96
1.01
1.03
7.10
1.02
1.99
4.04
7
6
5
4
3
2
1
ppm

^{13}C NMR ($CDCl_3$, 75 MHz)

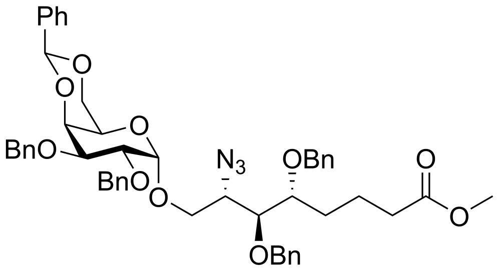

10

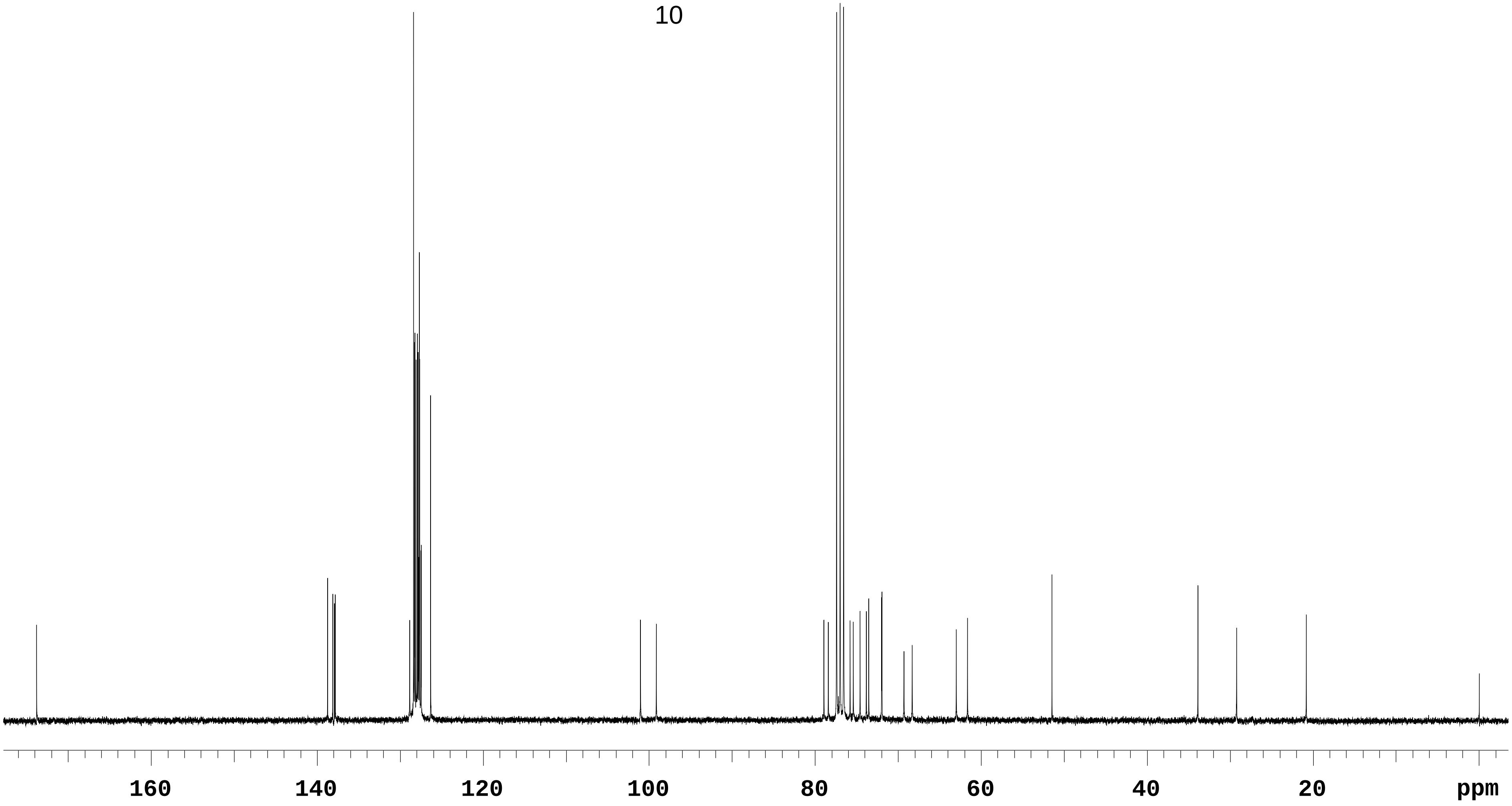

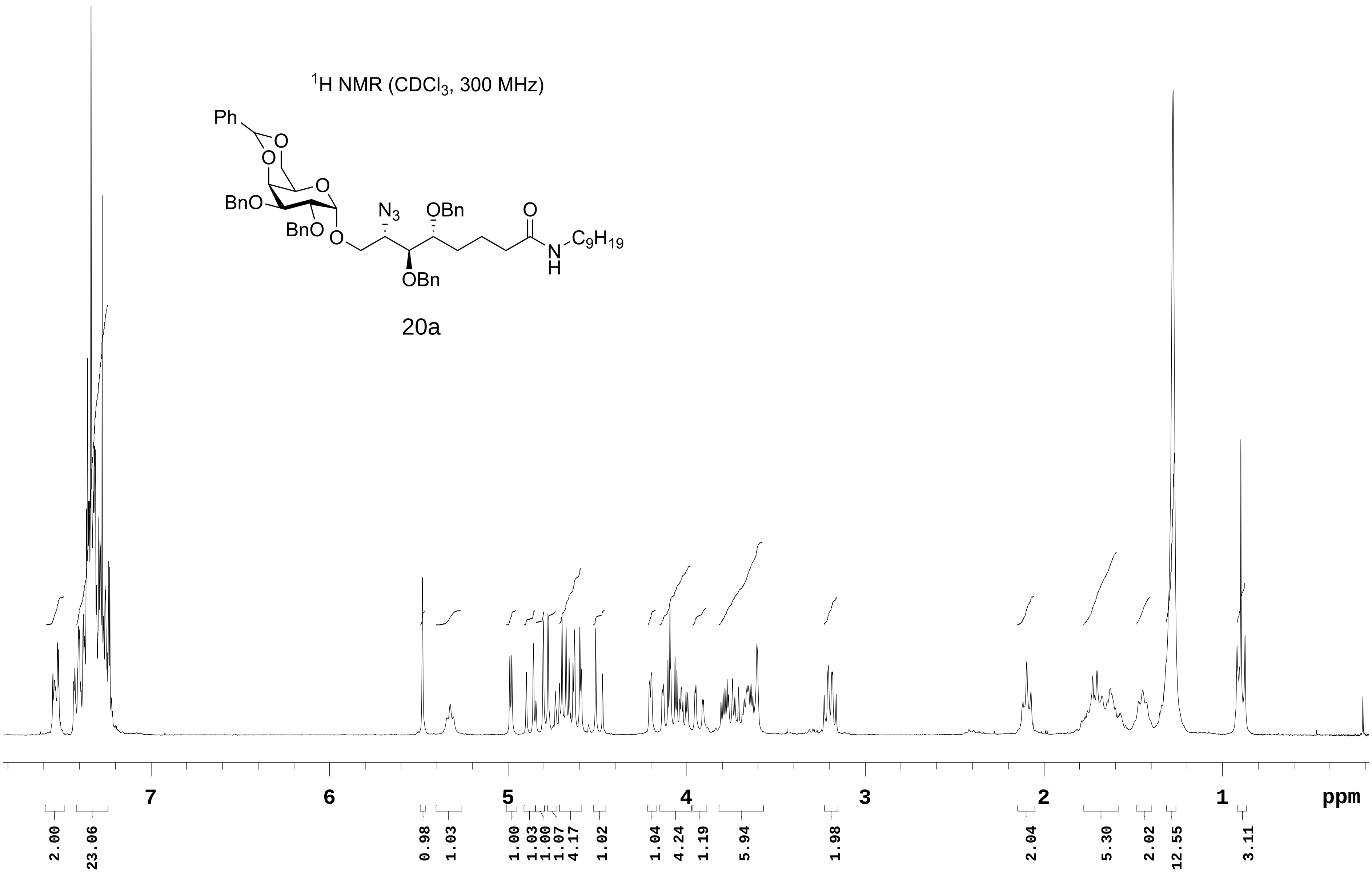

^{1}H NMR (CDCl$_3$, 300 MHz)
Ph
O
O
O
BnO
BnO
O
N$_3$
OBn
O
N
H
C$_9$H$_{19}$
OBn
20a
7
6
5
4
3
2
1
ppm
2.00
23.06
0.98
1.03
1.00
1.03
1.00
1.07
4.17
1.02
1.04
4.24
1.19
5.94
1.98
2.04
5.30
2.02
12.55
3.11

^{13}C NMR ($CDCl_3$, 75 MHz)

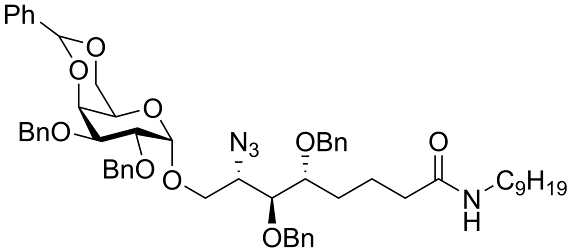

20a

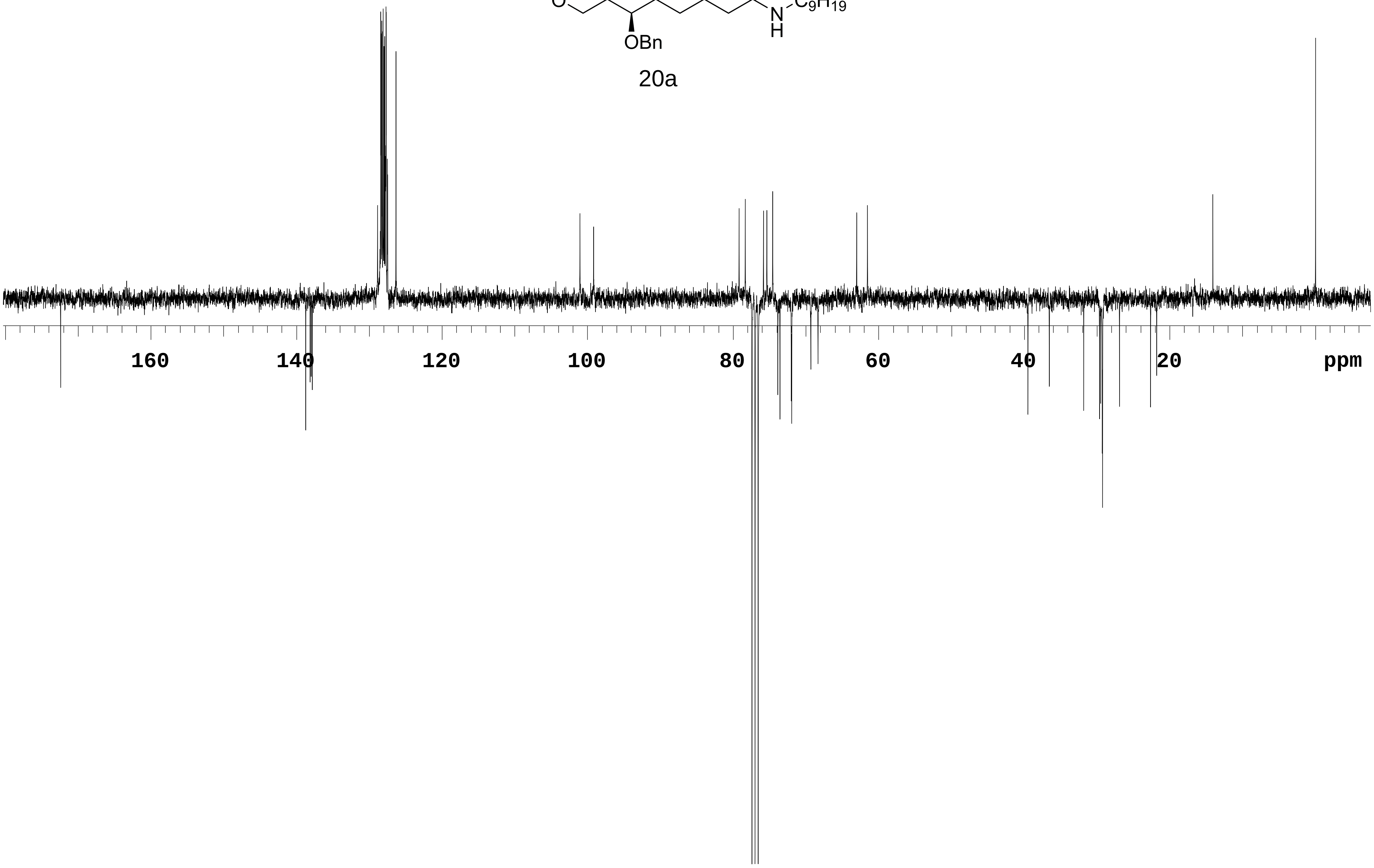

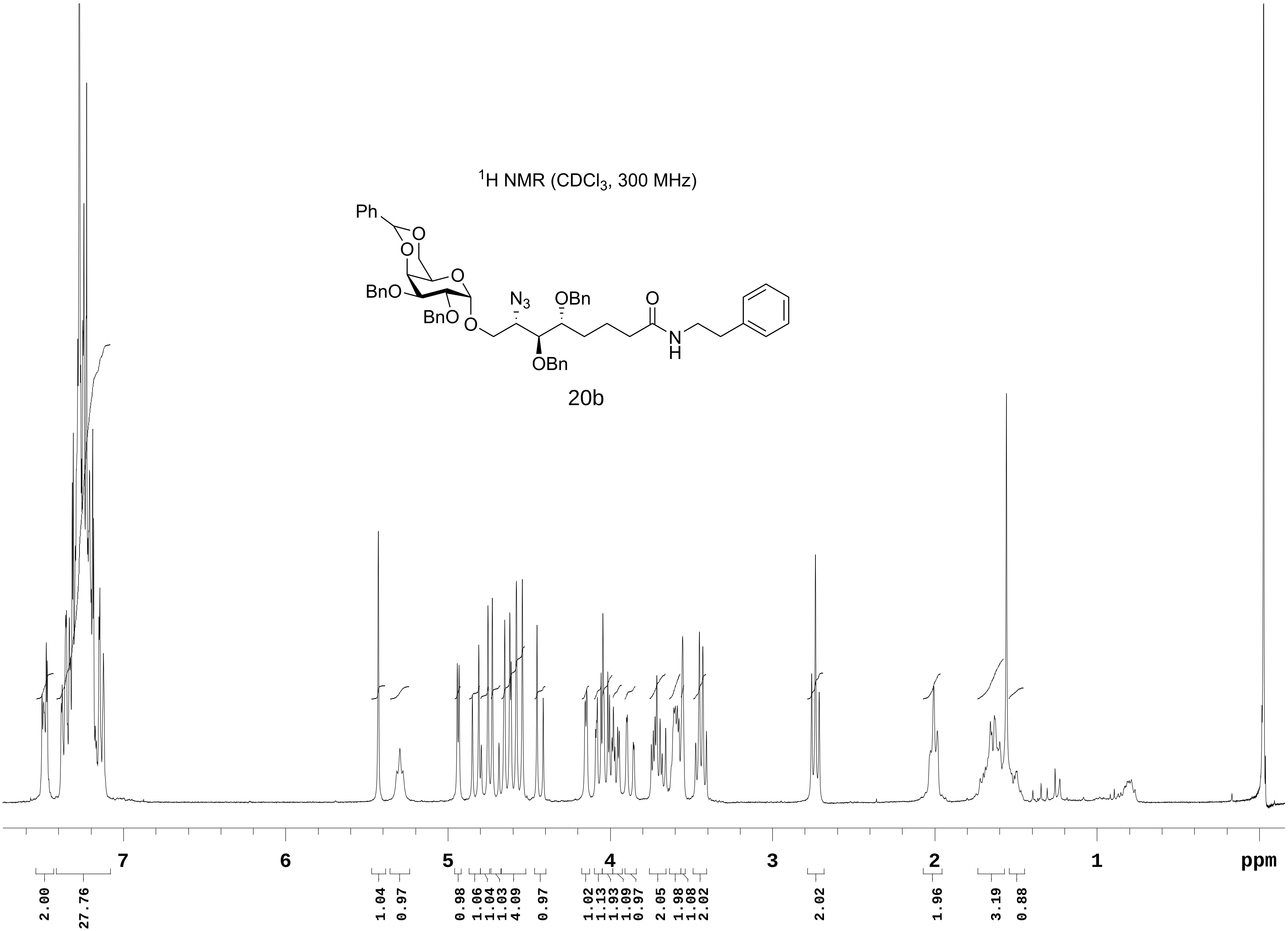

^{1}H NMR ($CDCl_3$, 300 MHz)
Ph
BnO
BnO
N_3
OBn
OBn
O
N
H
20b
7
6
5
4
3
2
1
ppm
2.00
27.76
1.04
0.97
0.98
1.06
1.04
1.03
4.09
0.97
1.02
1.13
1.93
1.09
0.97
2.05
1.98
1.08
2.02
2.02
1.96
3.19
0.88

^{13}C NMR ($CDCl_3$, 75 MHz)

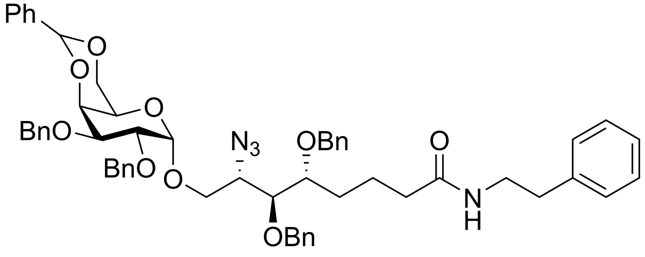

20b

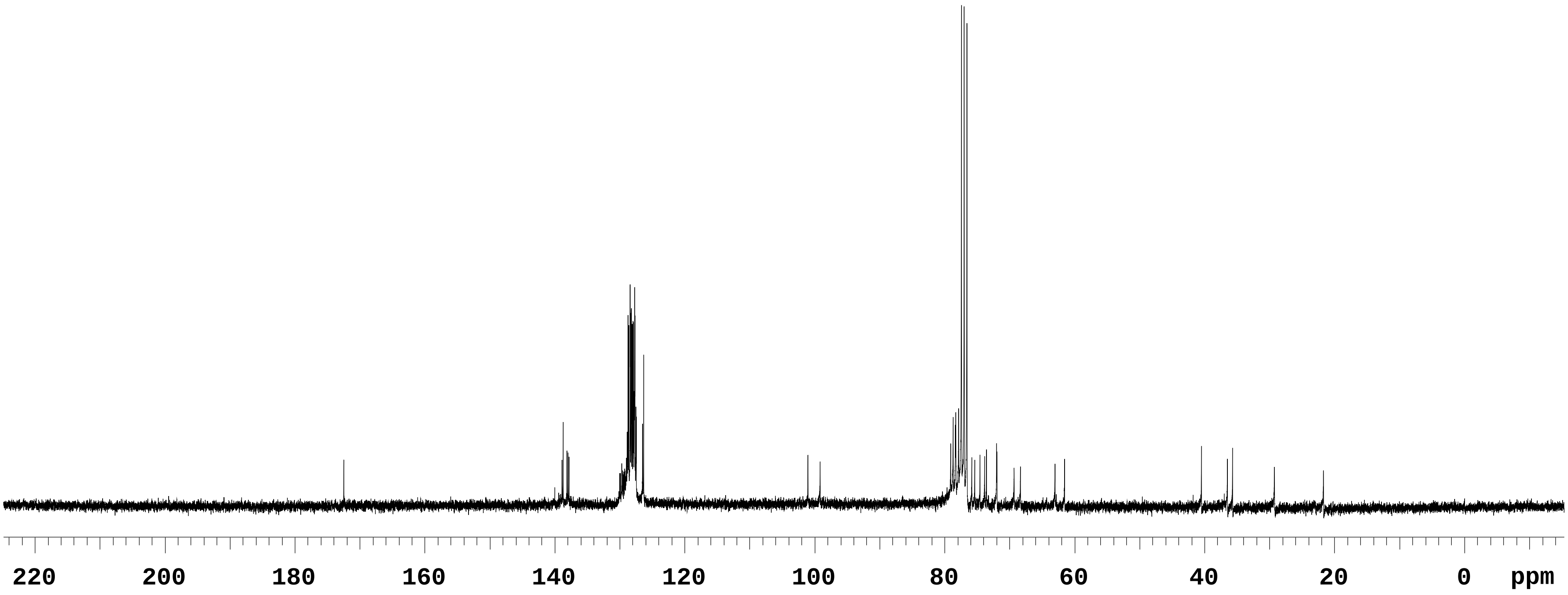

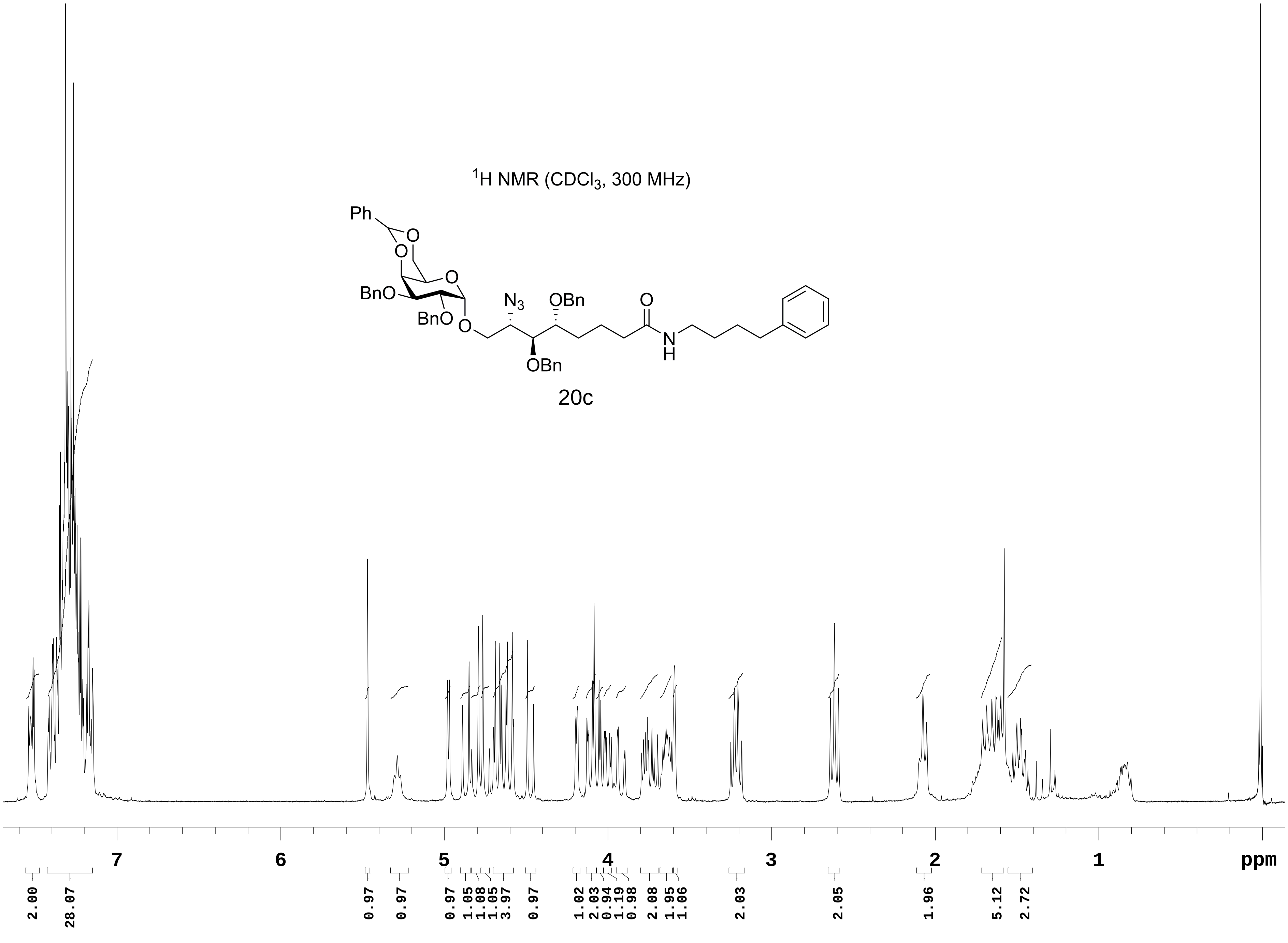

1H NMR (CDCl3, 300 MHz)
Ph
BnO
BnO
N3
OBn
OBn
O
N
H
20c
7
6
5
4
3
2
1
ppm
2.00
28.07
0.97
0.97
0.97
1.05
1.08
1.05
3.97
0.97
1.02
2.03
0.94
1.19
0.98
2.08
1.95
1.06
2.03
2.05
1.96
5.12
2.72

^{13}C NMR ($CDCl_3$, 75 MHz)

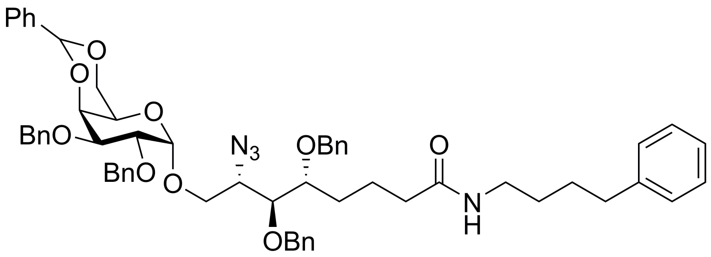

20c

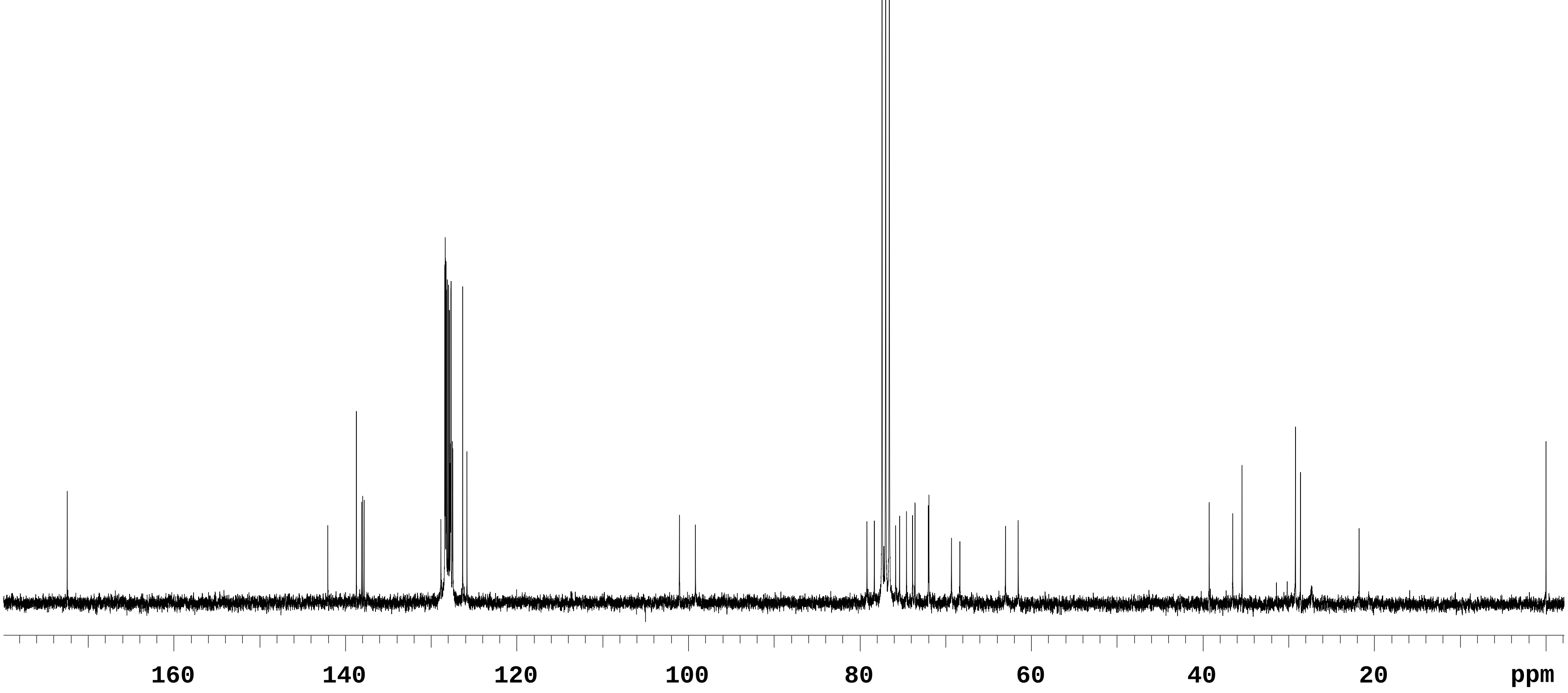

^1H NMR ($CDCl_3$, 300 MHz)

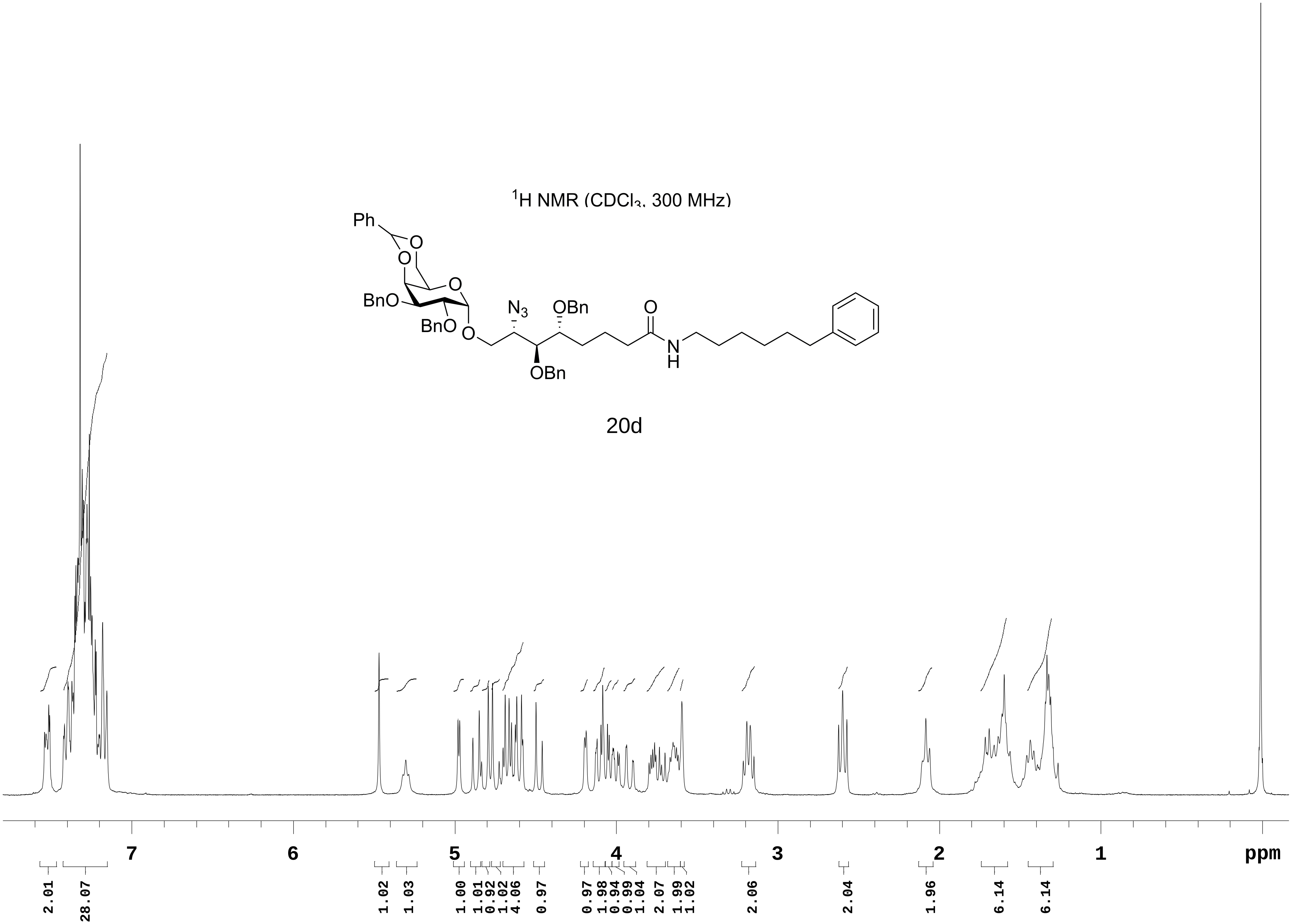

20d

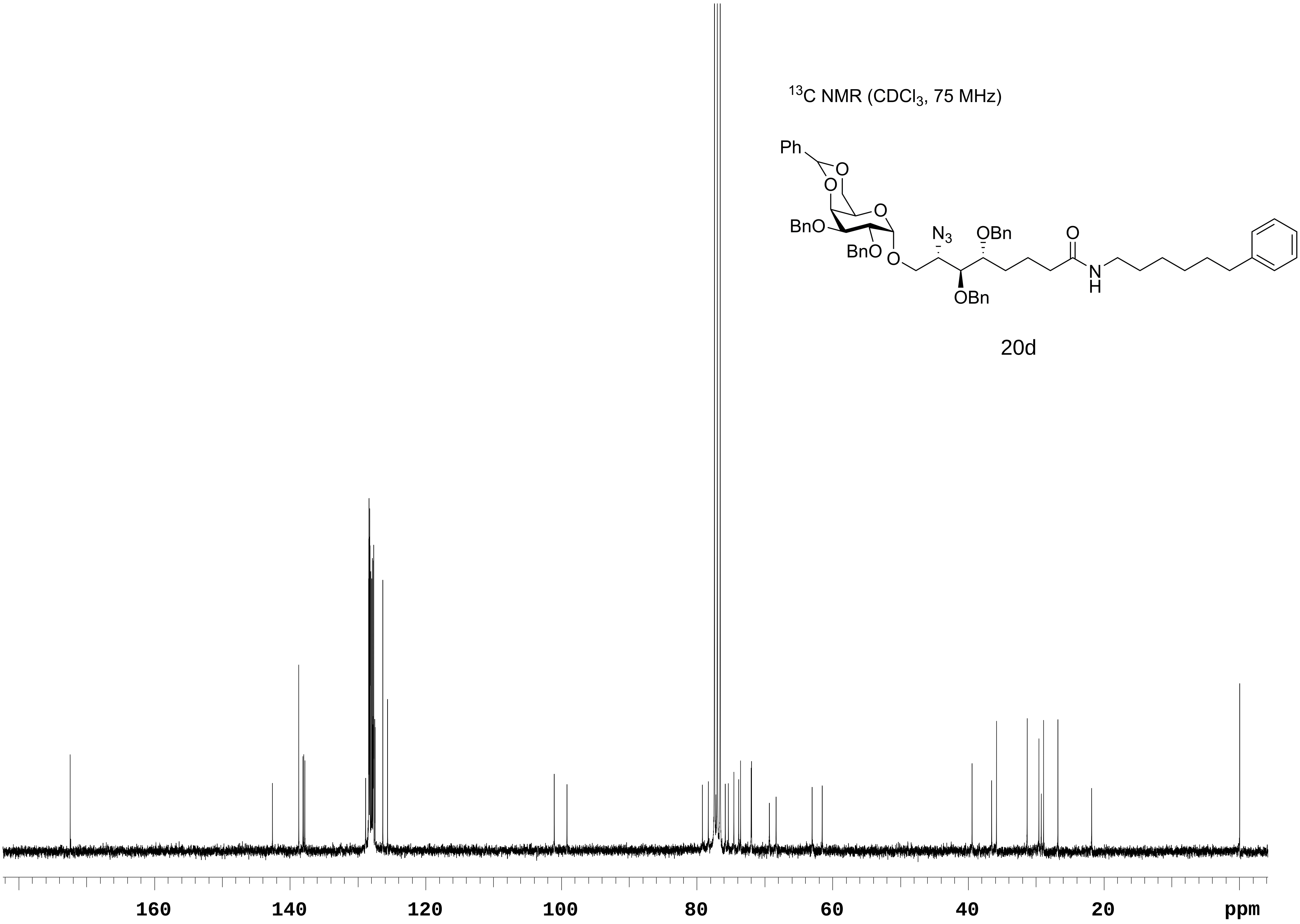

^{13}C NMR (CDCl$_3$, 75 MHz)
Ph
O
O
O
BnO
BnO
O
N$_3$
OBn
OBn
O
N
H
20d
160
140
120
100
80
60
40
20
ppm

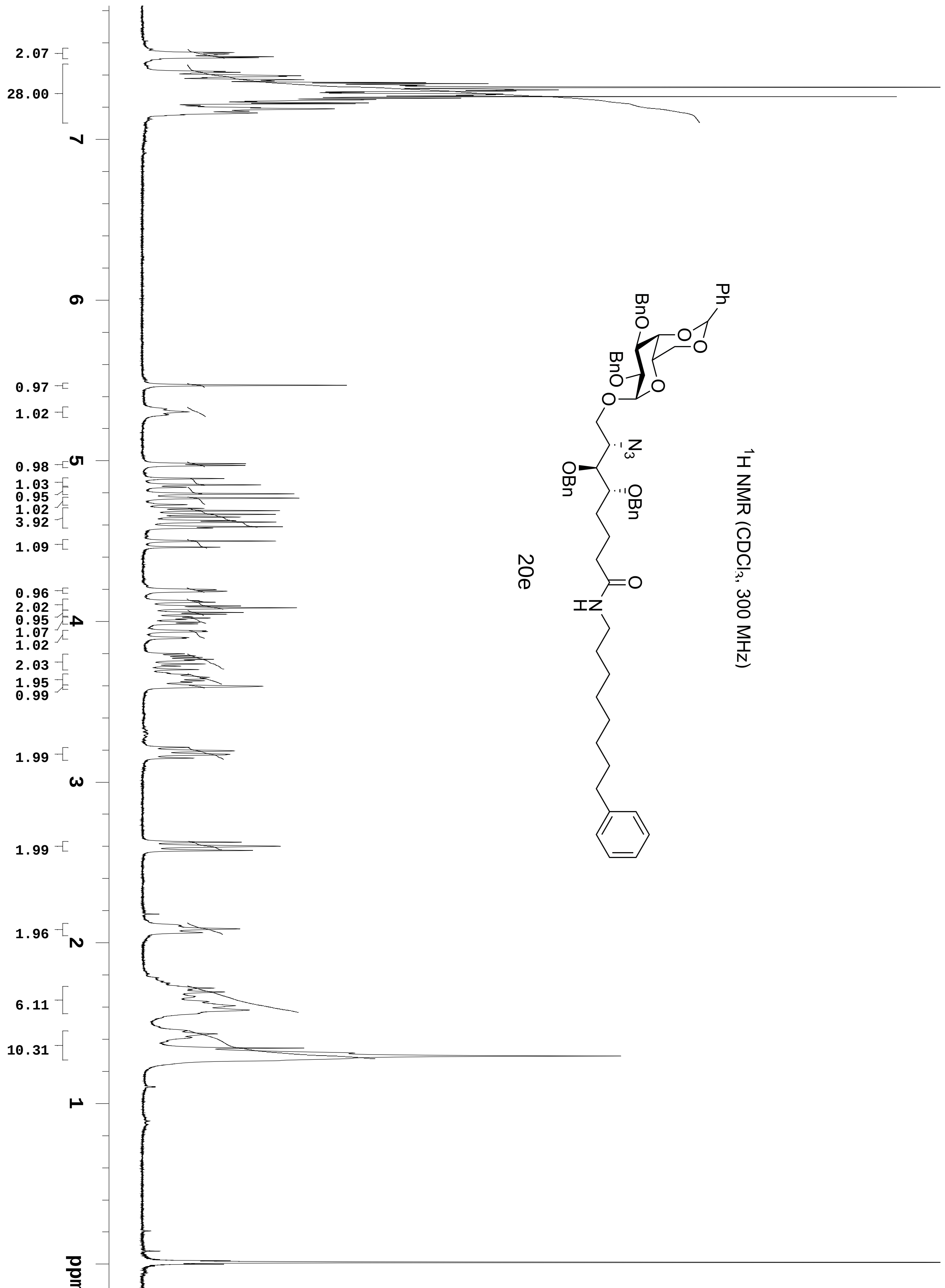

1H NMR (CDCl3, 300 MHz)
20e
Ph
BnO
BnO
N3
OBn
OBn
O
N
H
ppm
7
6
5
4
3
2
1
2.07
28.00
0.97
1.02
0.98
1.03
0.95
1.02
3.92
1.09
0.96
2.02
0.95
1.07
1.02
2.03
1.95
0.99
1.99
1.99
1.96
6.11
10.31

^{13}C NMR ($CDCl_3$, 75 MHz)

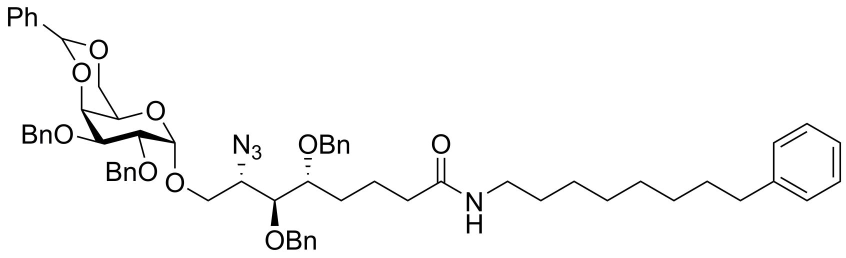

20e

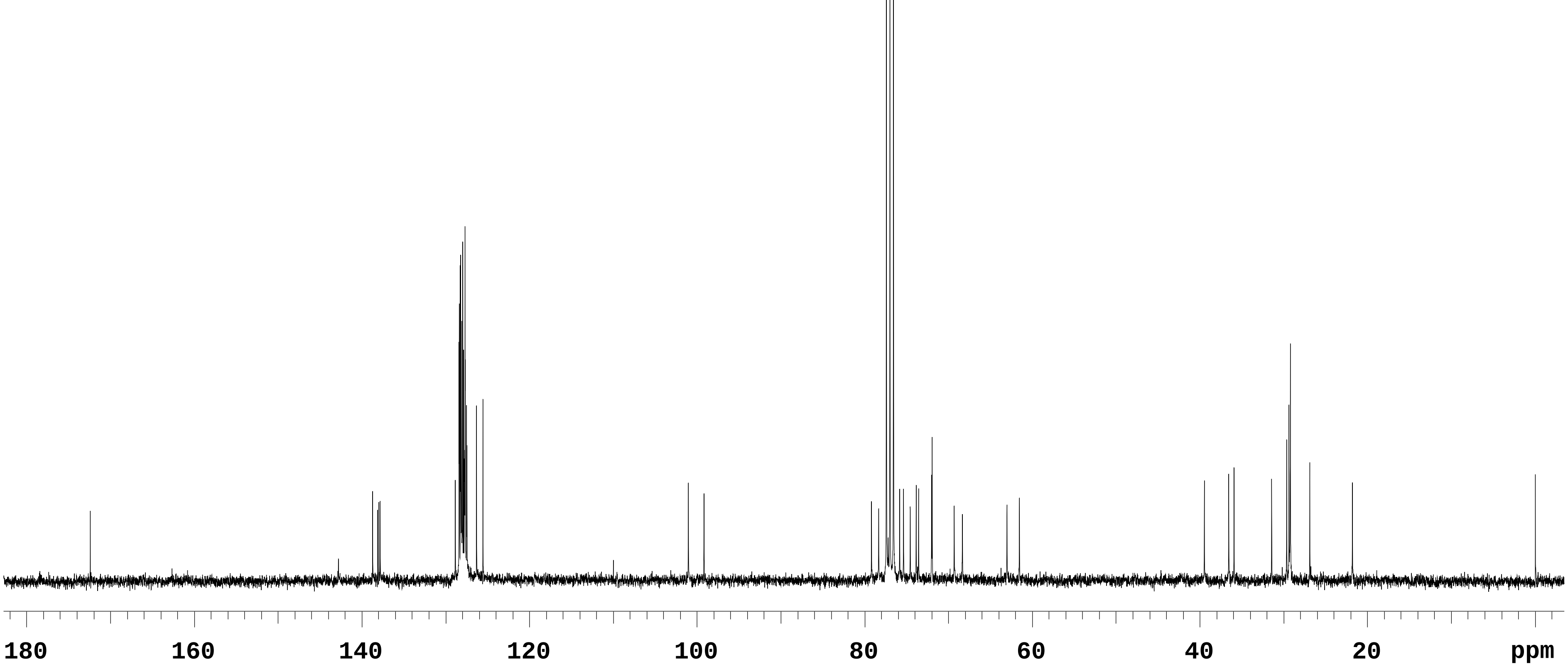

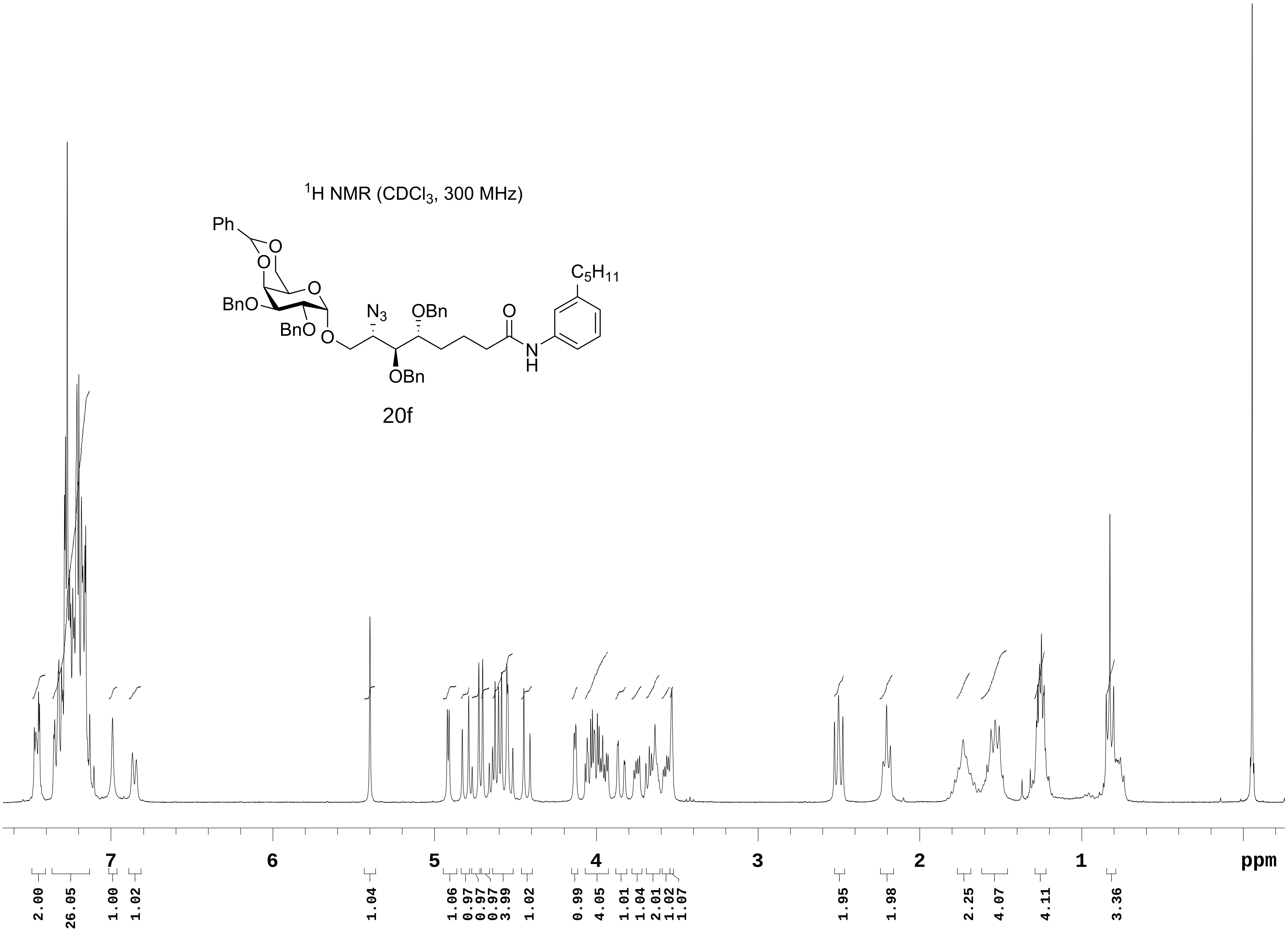

1H NMR (CDCl3, 300 MHz)
Ph
O
O
O
BnO
BnO
O
N3
OBn
OBn
O
N
H
C5H11
20f
7
6
5
4
3
2
1
ppm
2.00
26.05
1.00
1.02
1.04
1.06
0.97
0.97
0.97
3.99
1.02
0.99
4.05
1.01
1.04
2.01
1.02
1.07
1.95
1.98
2.25
4.07
4.11
3.36

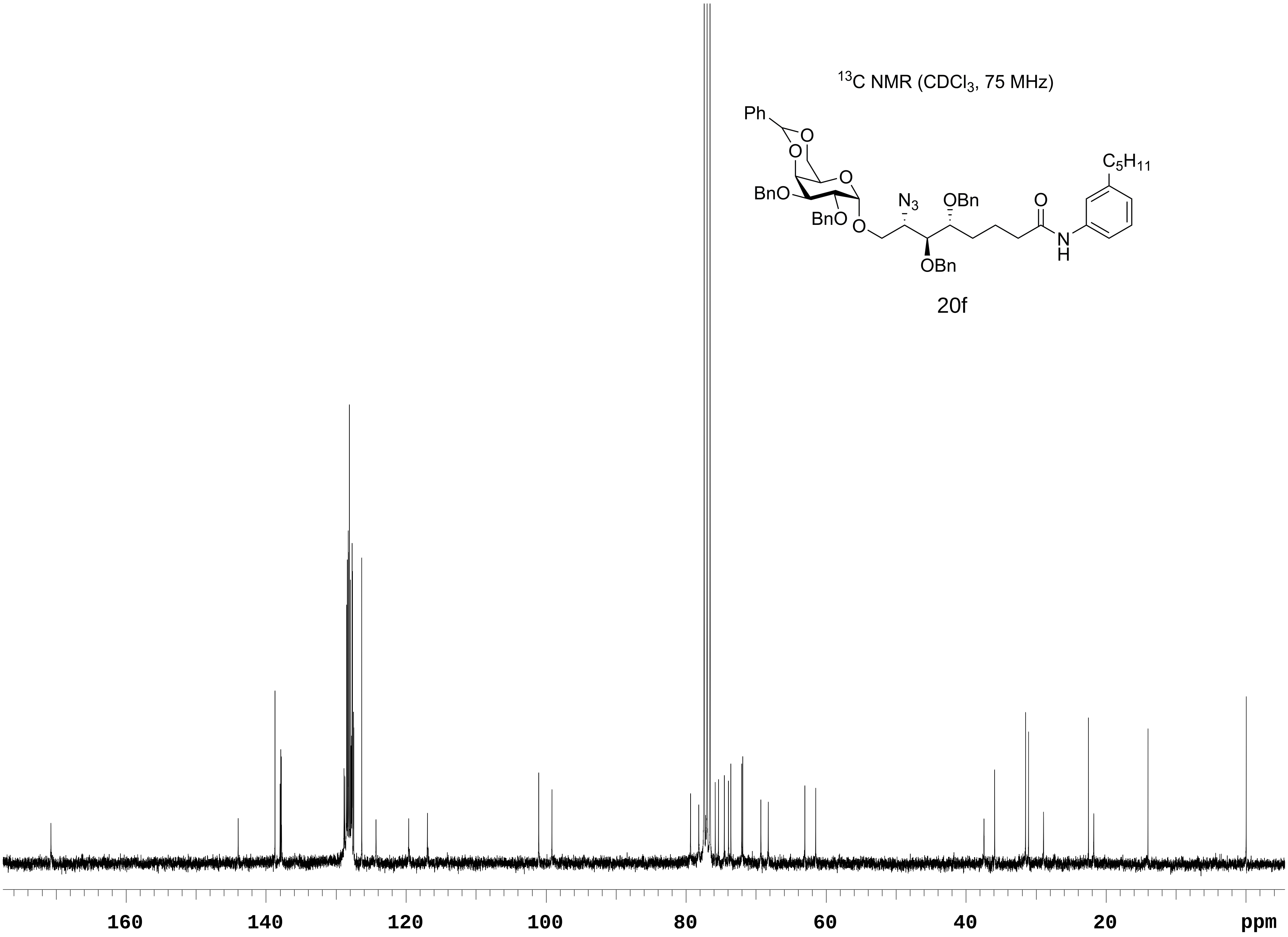

13C NMR (CDCl3, 75 MHz)
Ph
O
O
BnO
BnO
O
O
N3
OBn
OBn
O
N
H
C5H11
20f
160
140
120
100
80
60
40
20
ppm

^{1}H NMR ($CDCl_3$, 300 MHz)

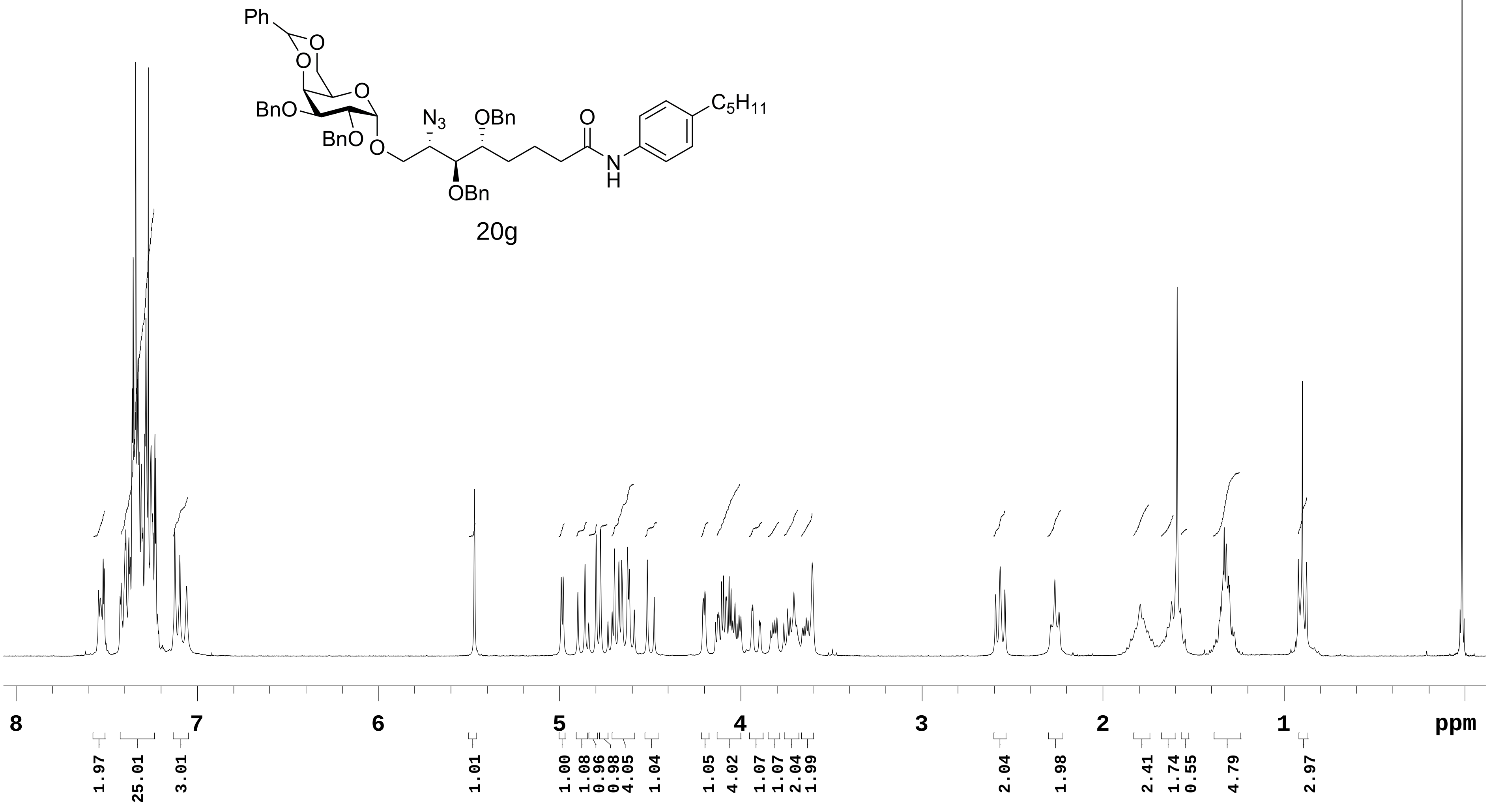

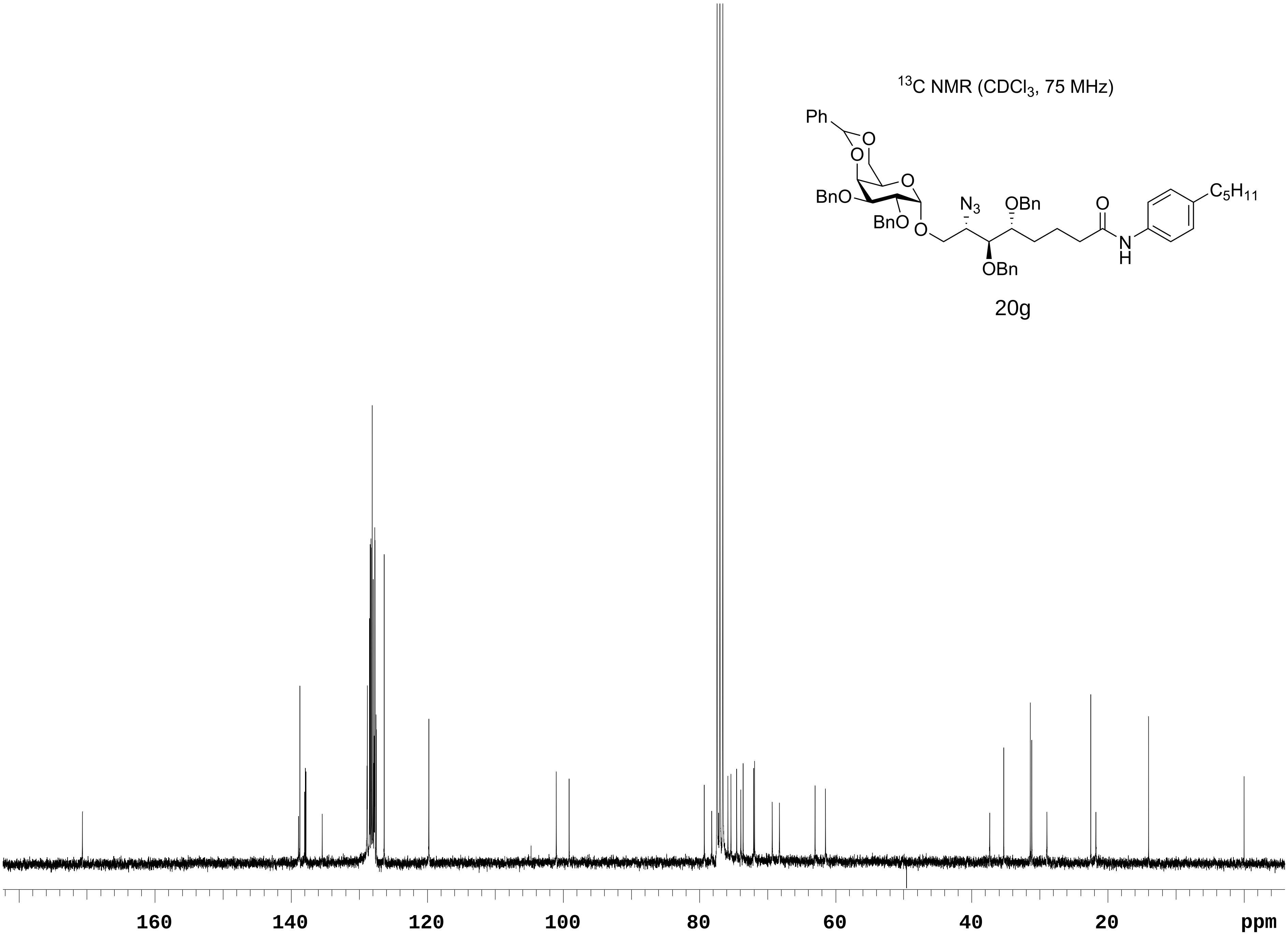

^{13}C NMR ($CDCl_3$, 75 MHz)
Ph
O
O
O
BnO
BnO
O
N_3
OBn
OBn
O
N
H
C_5H_{11}
20g
160
140
120
100
80
60
40
20
ppm

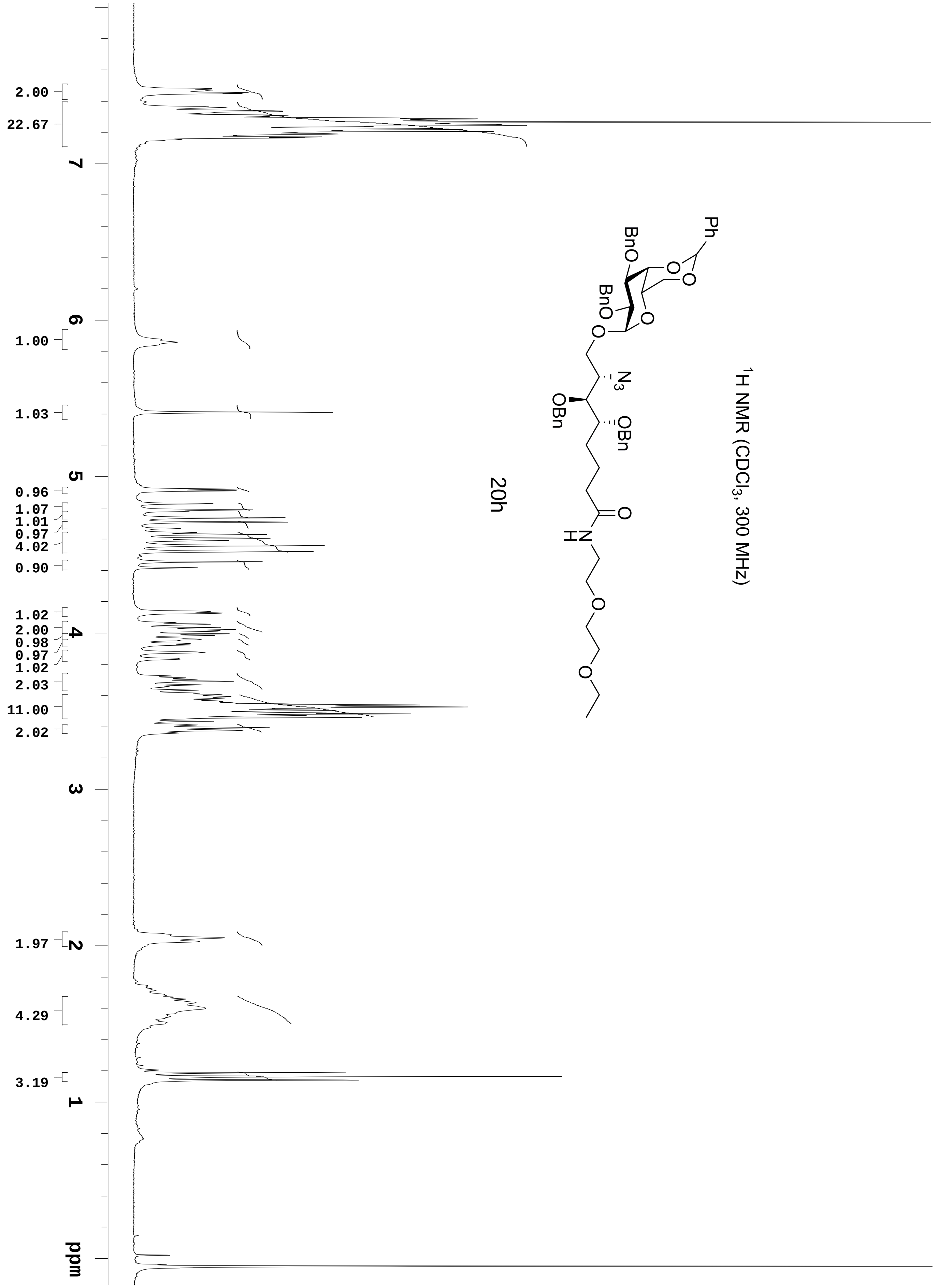

1H NMR (CDCl3, 300 MHz)
20h
Ph
BnO
BnO
N3
OBn
OBn
O
N
H
2.00
22.67
1.00
1.03
0.96
1.07
1.01
0.97
4.02
0.90
1.02
2.00
0.98
0.97
1.02
2.03
11.00
2.02
1.97
4.29
3.19
7
6
5
4
3
2
1
ppm

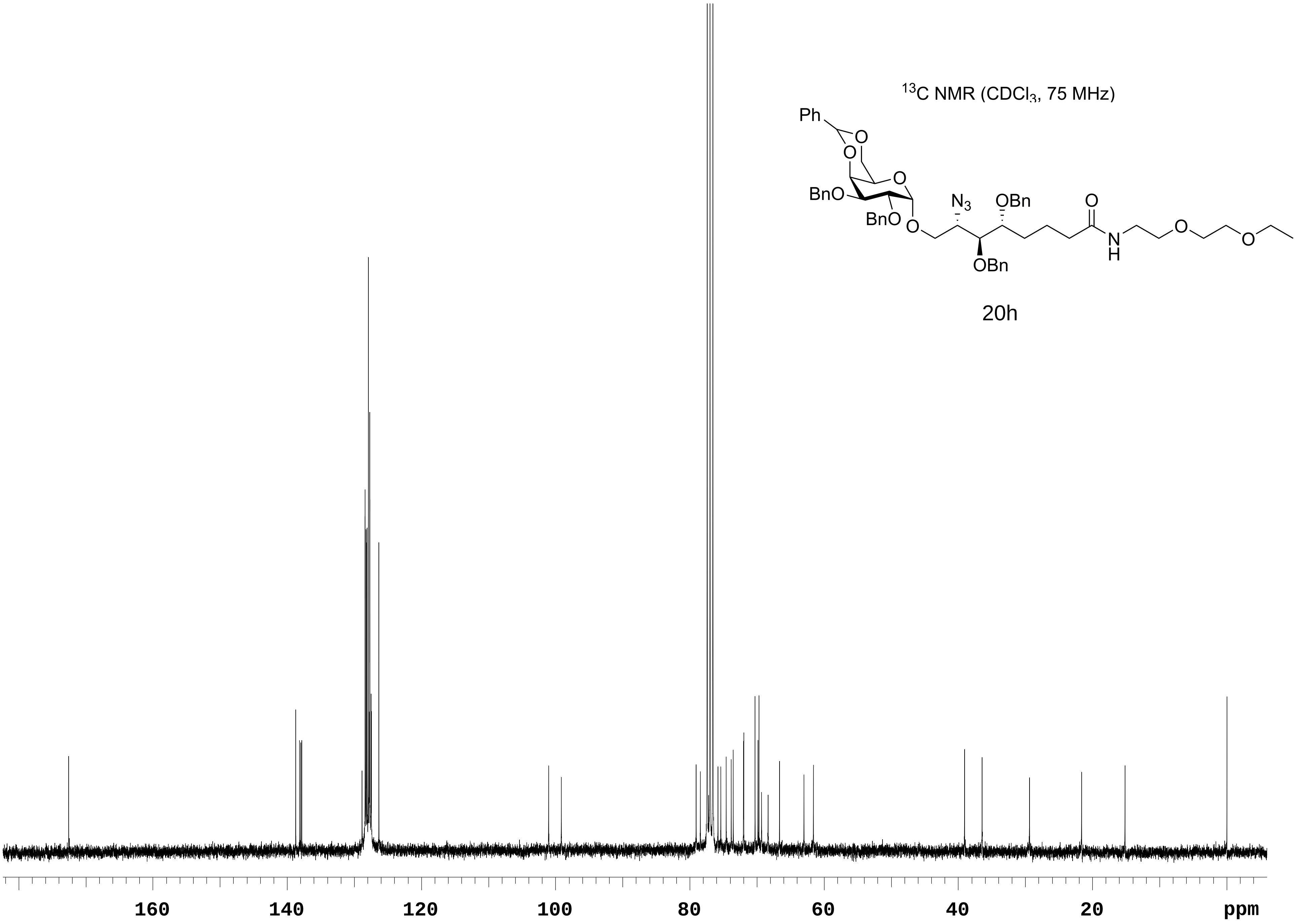
^{13}C NMR (CDCl$_3$, 75 MHz)
Ph
O
O
O
BnO
BnO
O
N$_3$
OBn
O
N
H
O
O
OBn
160
140
120
100
80
60
40
20
ppm
20h

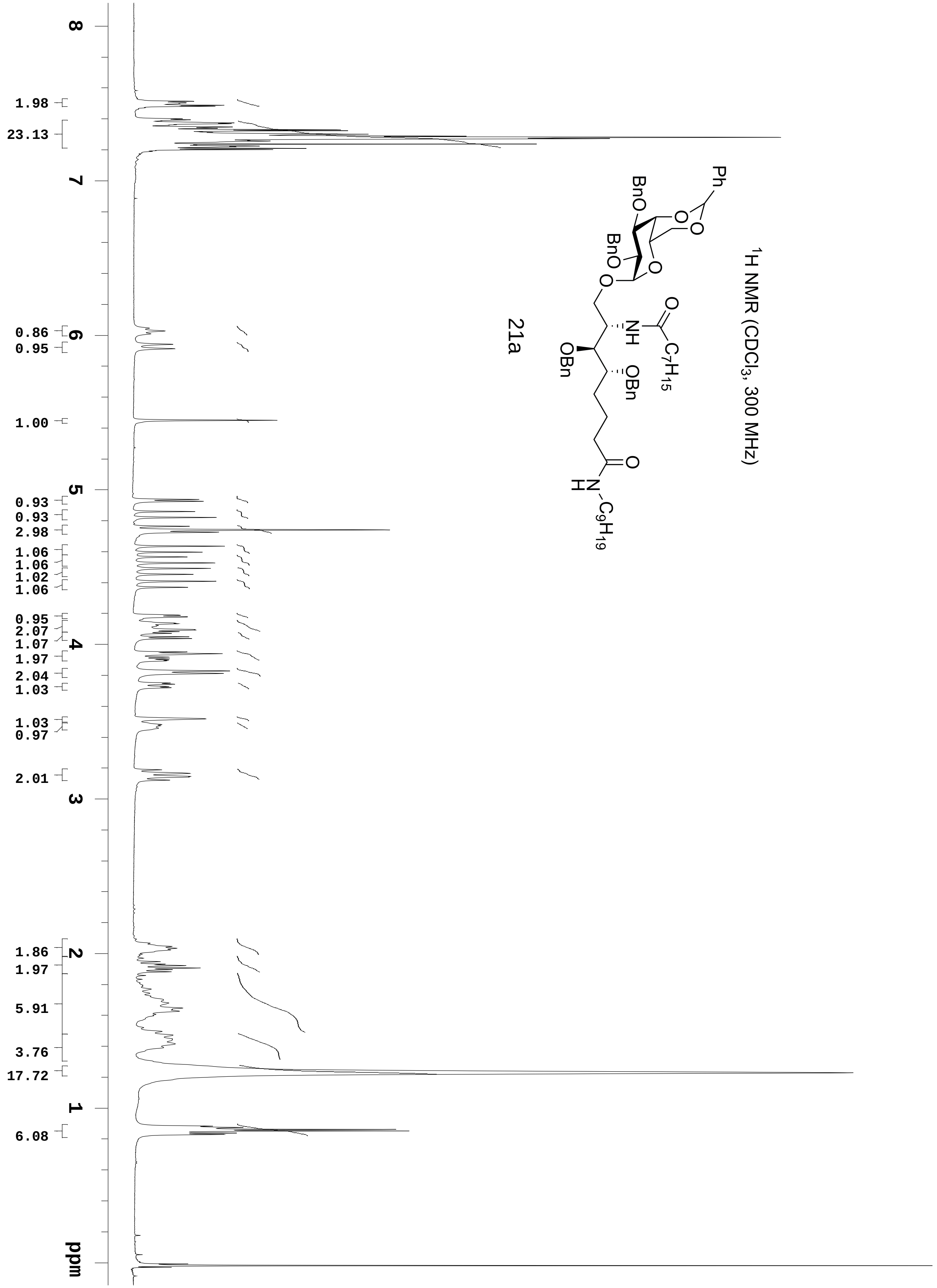

1H NMR (CDCl3, 300 MHz)
21a
Ph
BnO
BnO
OBn
OBn
NH
C7H15
C9H19
ppm
8
7
6
5
4
3
2
1
1.98
23.13
0.86
0.95
1.00
0.93
0.93
2.98
1.06
1.06
1.02
1.06
0.95
2.07
1.07
1.97
2.04
1.03
1.03
0.97
2.01
1.86
1.97
5.91
3.76
17.72
6.08

^{13}C NMR ($CDCl_3$, 75 MHz)

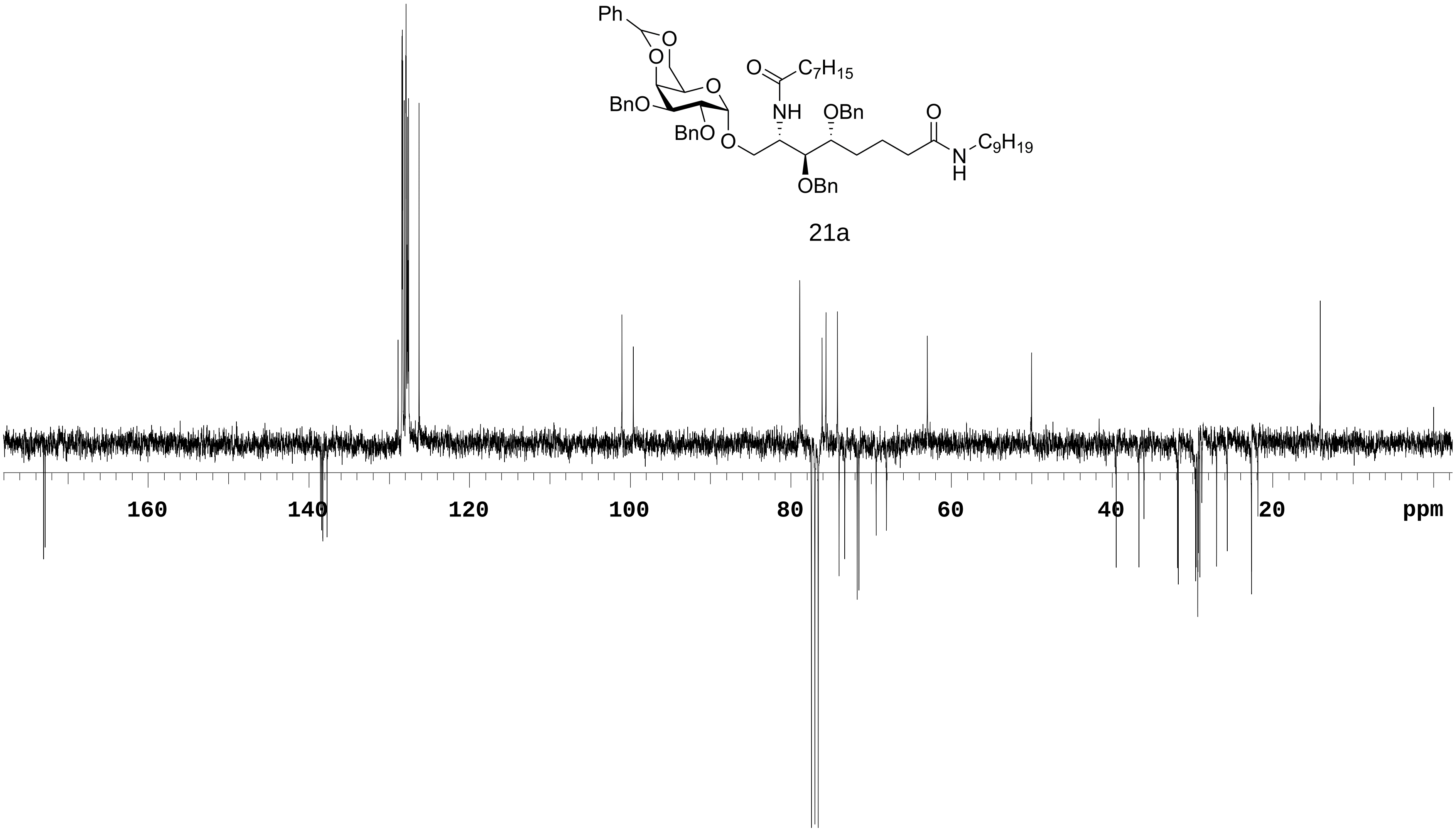

21a

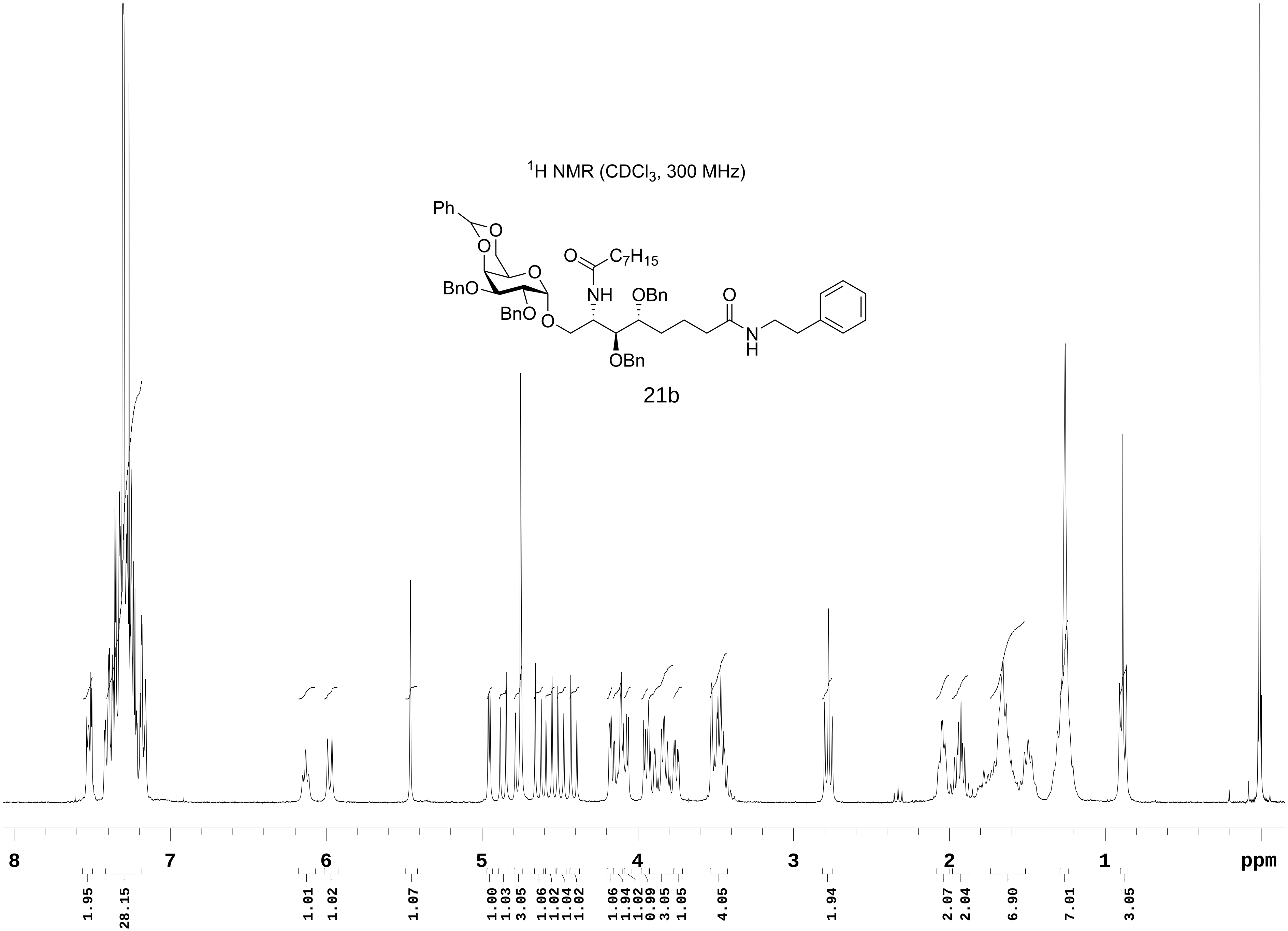
1H NMR (CDCl3, 300 MHz)
Ph
O
O
O
BnO
BnO
O
O
C7H15
NH
OBn
O
N
H
OBn
8
7
6
5
4
3
2
1
ppm
1.95
28.15
1.01
1.02
1.07
1.00
1.03
3.05
1.06
1.02
1.04
1.02
1.06
1.94
1.02
0.99
3.05
1.05
4.05
1.94
2.07
2.04
6.90
7.01
3.05

21b

^{13}C NMR ($CDCl_3$, 75 MHz)

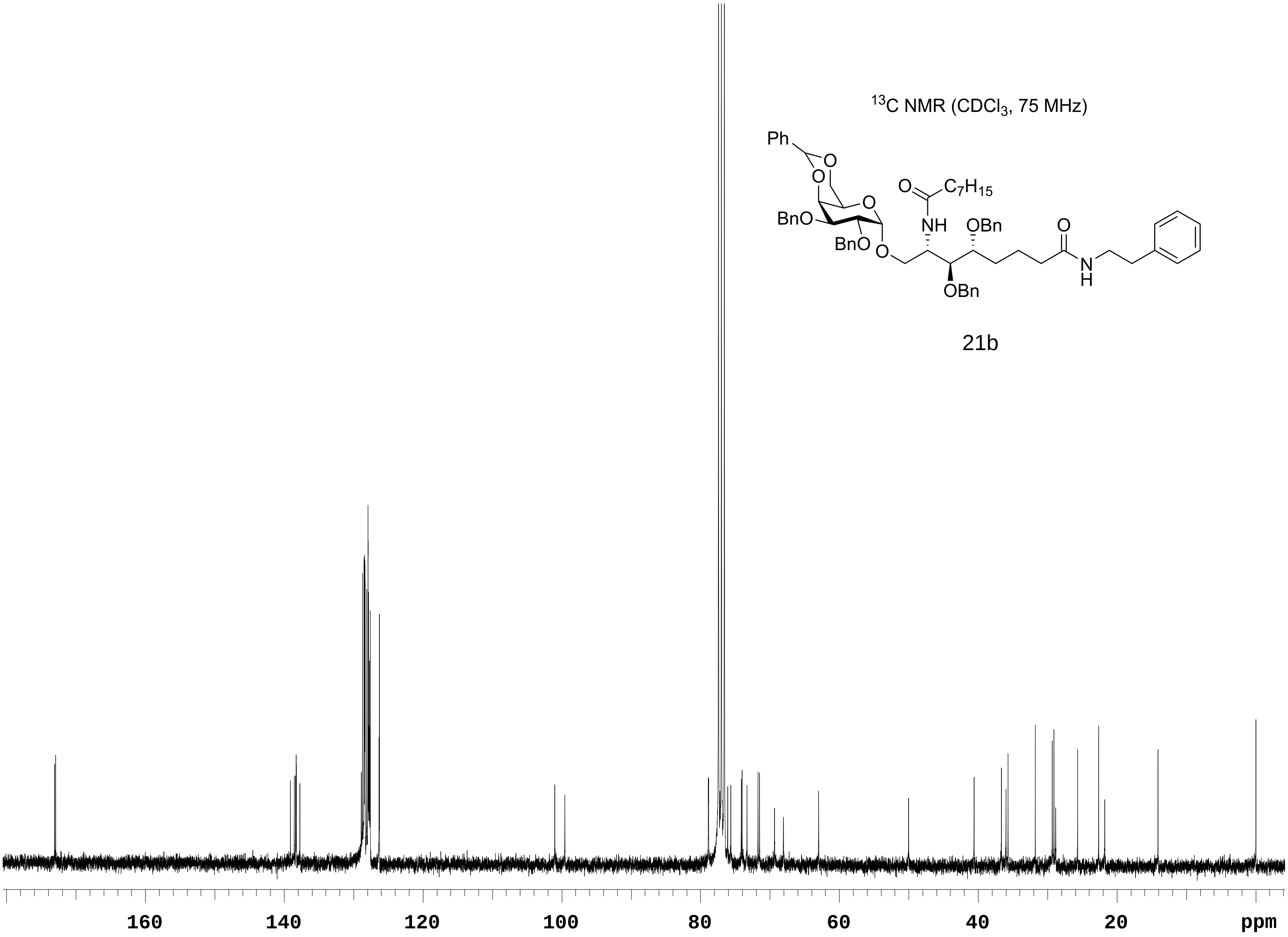

21b

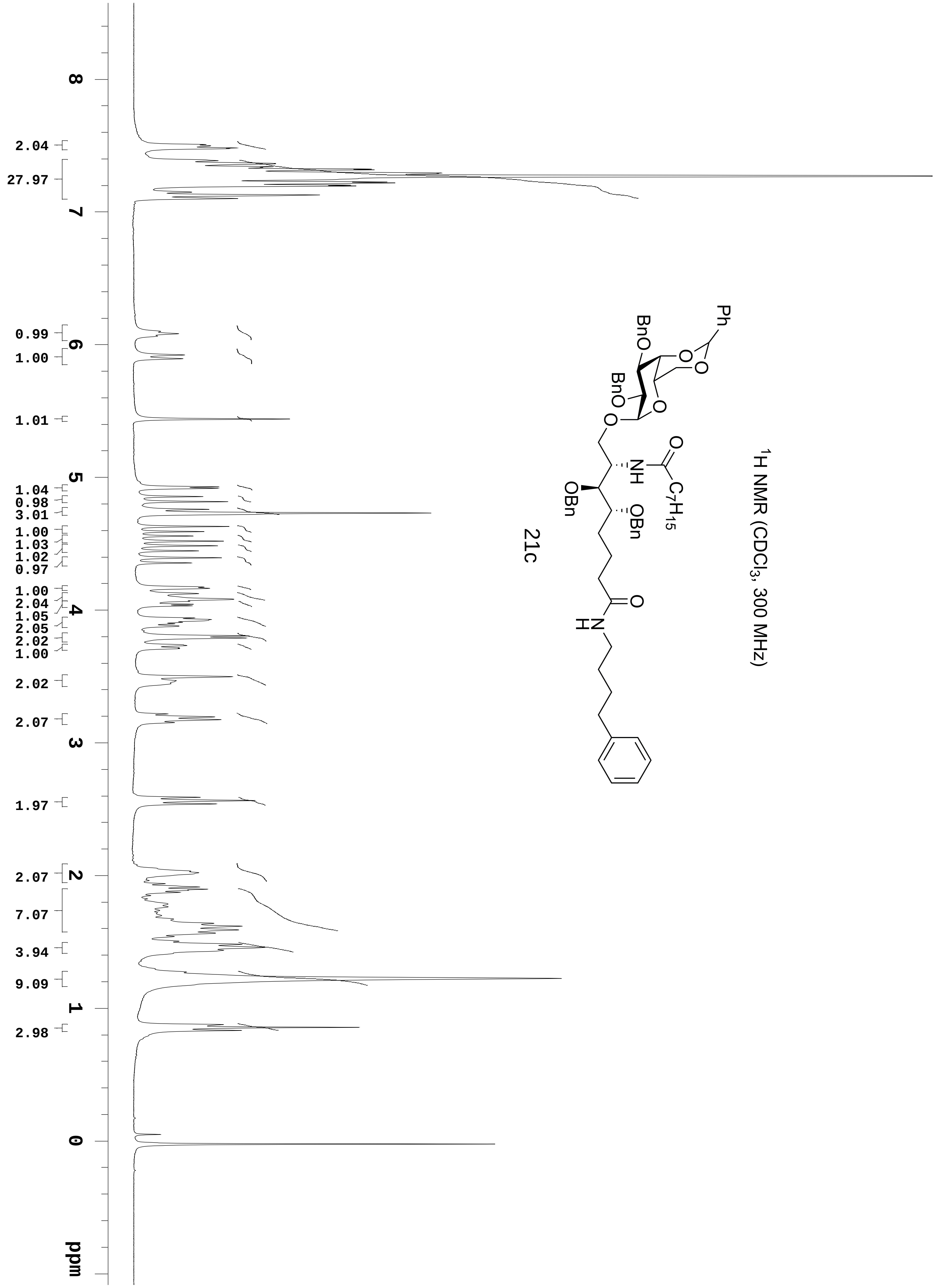

^{1}H NMR ($CDCl_3$, 300 MHz)
Ph
BnO
BnO
O
NH
C7H15
OBn
OBn
N
H
21c
2.04
27.97
0.99
1.00
1.01
1.04
0.98
3.01
1.00
1.03
1.02
0.97
1.00
2.04
1.05
2.05
2.02
1.00
2.02
2.07
1.97
2.07
7.07
3.94
9.09
2.98
8
7
6
5
4
3
2
1
0
ppm

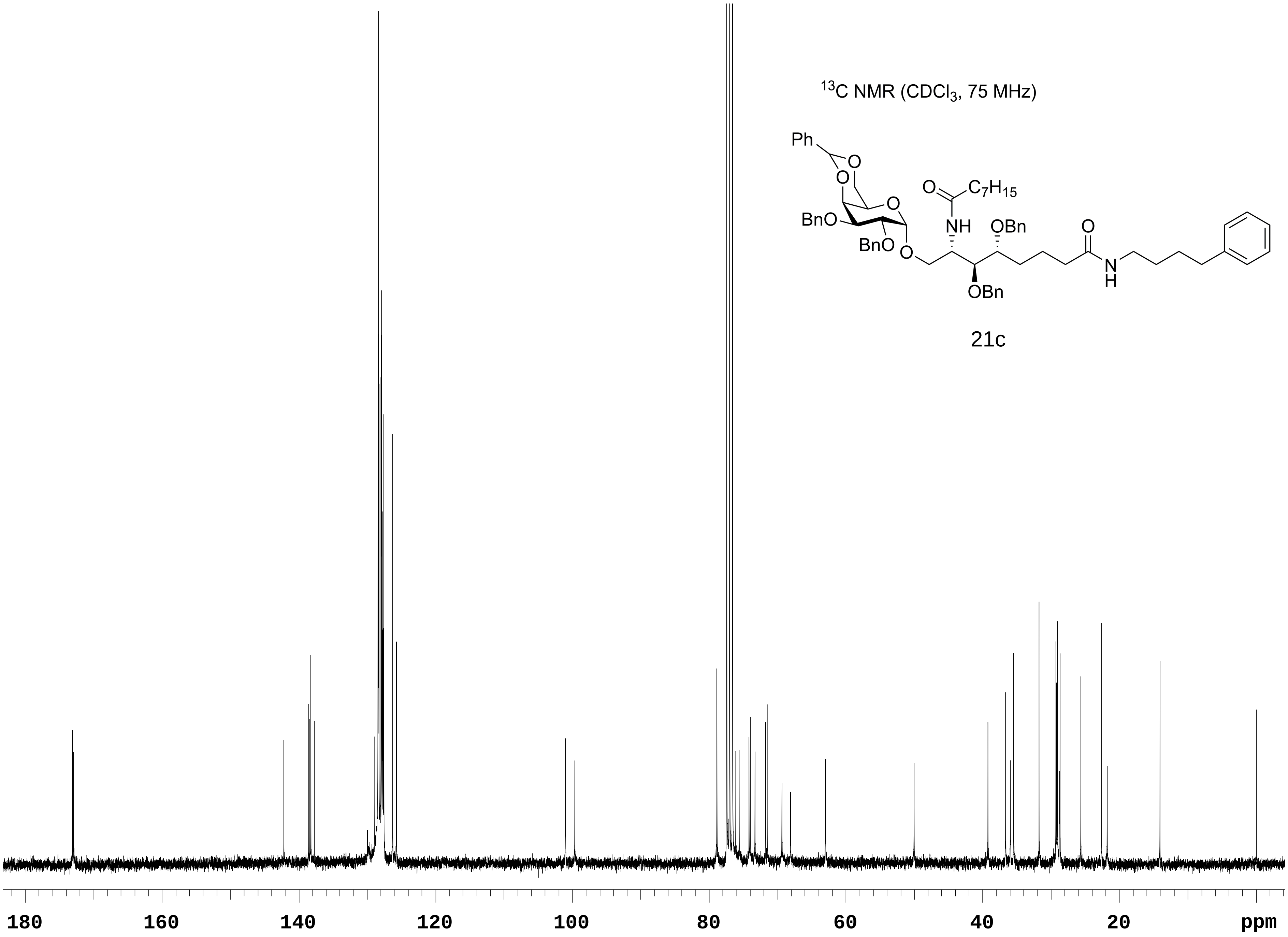

^{13}C NMR ($CDCl_3$, 75 MHz)
Ph
O
O
O
C_7H_{15}
BnO
NH
OBn
O
BnO
O
N
H
OBn
21c
180
160
140
120
100
80
60
40
20
ppm

^1H NMR ($CDCl_3$, 300 MHz)

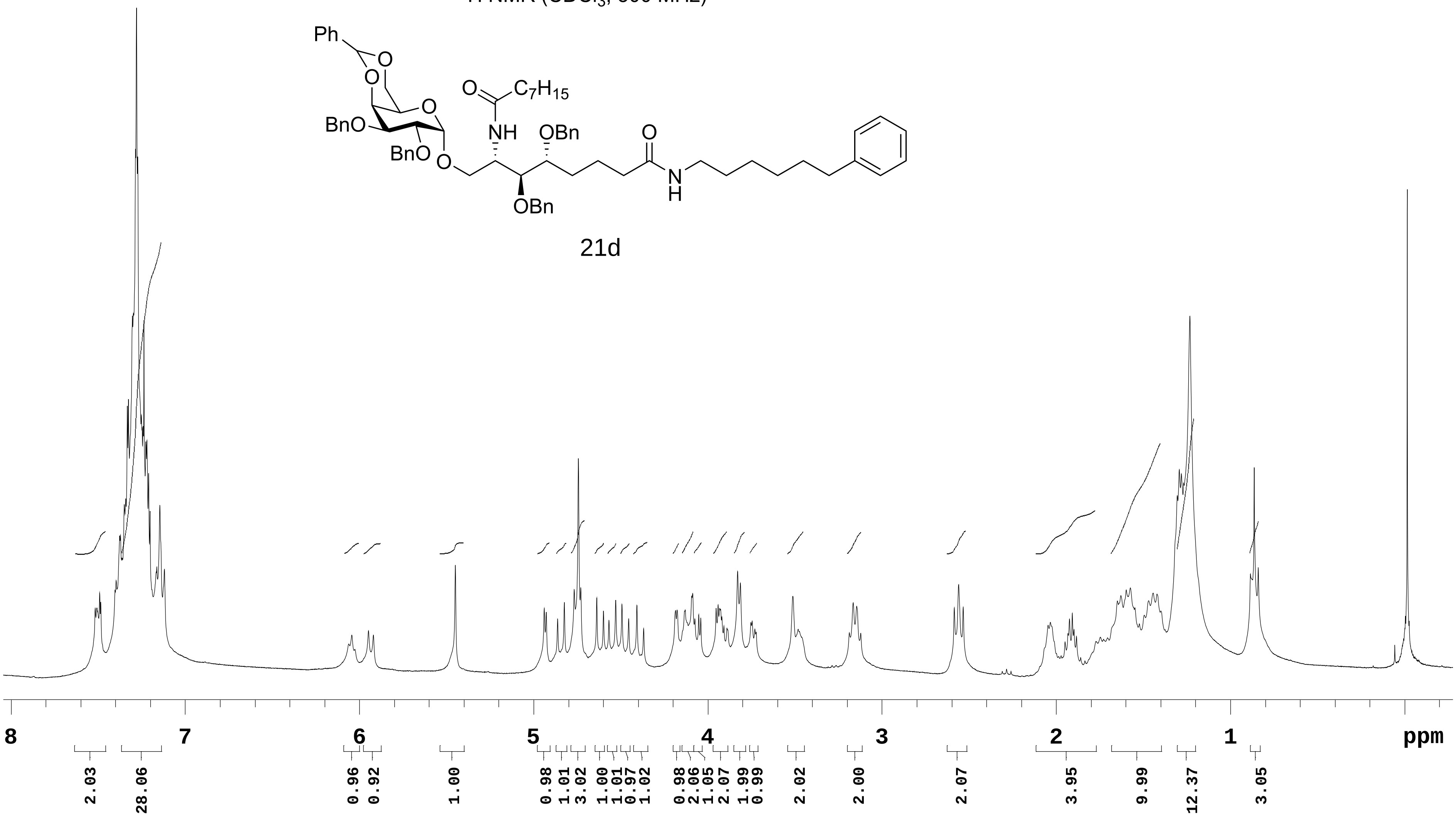

21d

^{13}C NMR ($CDCl_3$, 75 MHz)

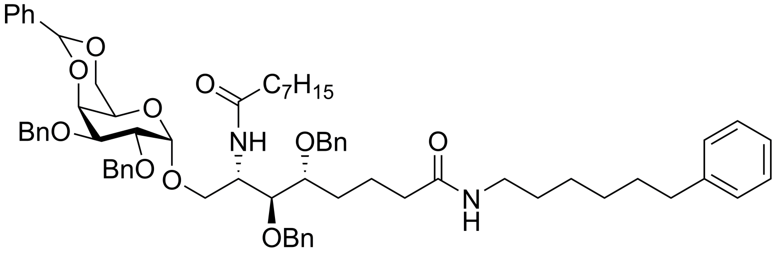

21d

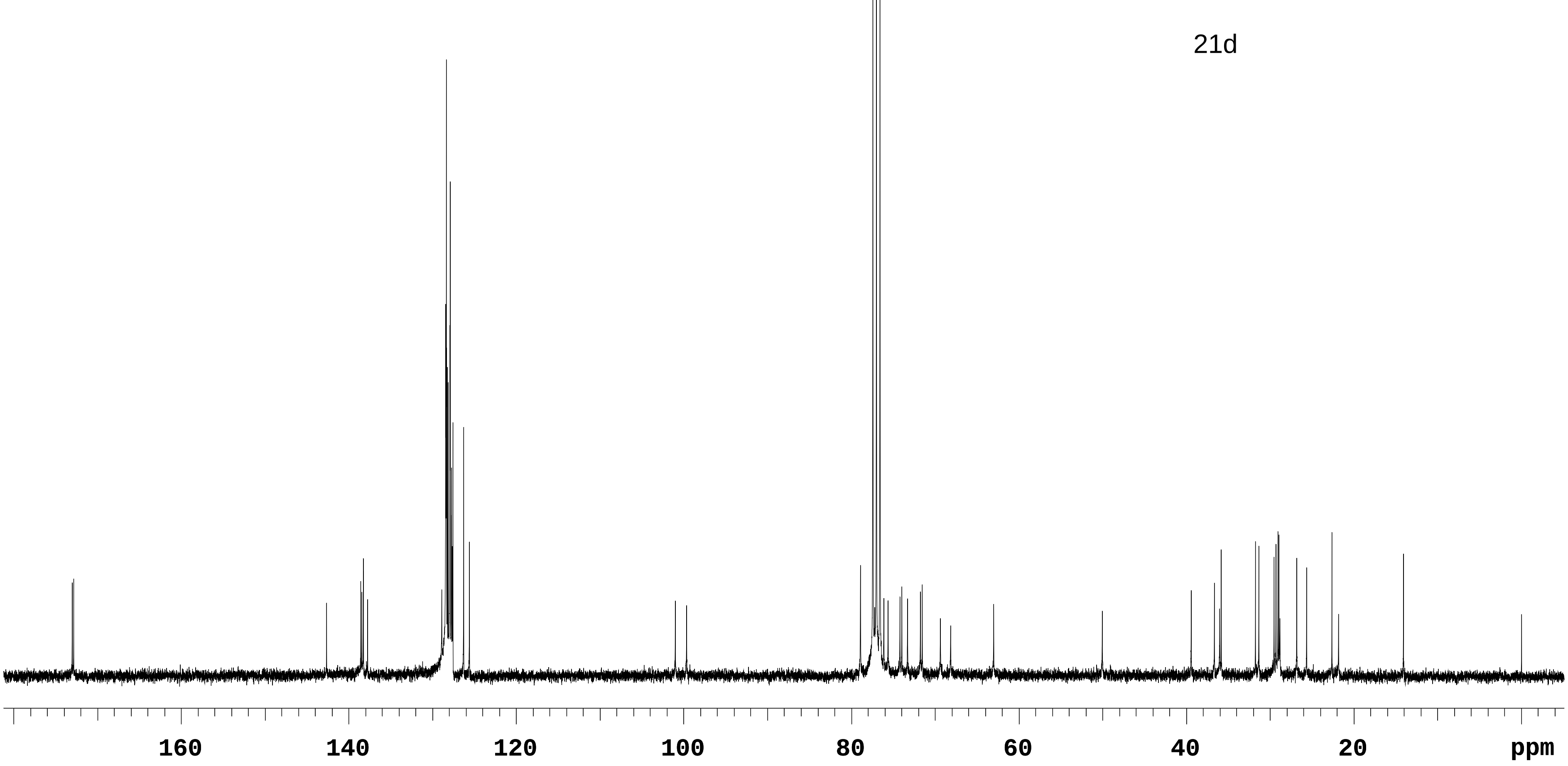

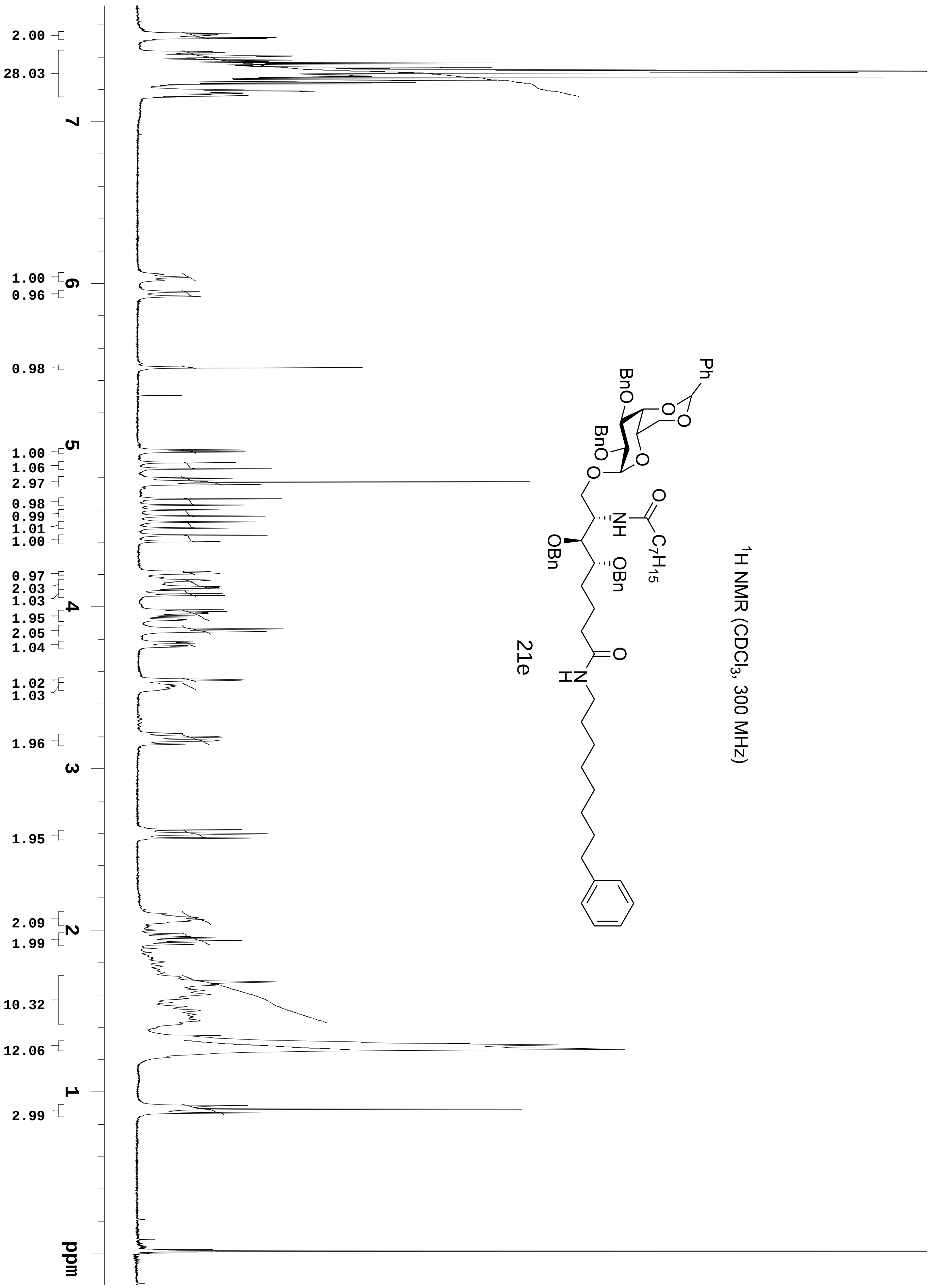

1H NMR (CDCl3, 300 MHz)
21e
Ph
BnO
BnO
O
NH
C7H15
OBn
OBn
N
H
7
6
5
4
3
2
1
ppm
2.00
28.03
1.00
0.96
0.98
1.00
1.06
2.97
0.98
0.99
1.01
1.00
0.97
2.03
1.03
1.95
2.05
1.04
1.02
1.03
1.96
1.95
2.09
1.99
10.32
12.06
2.99

^{13}C NMR ($CDCl_3$, 75 MHz)

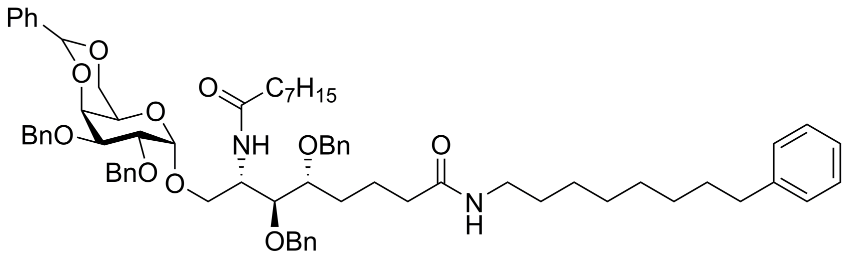

21e

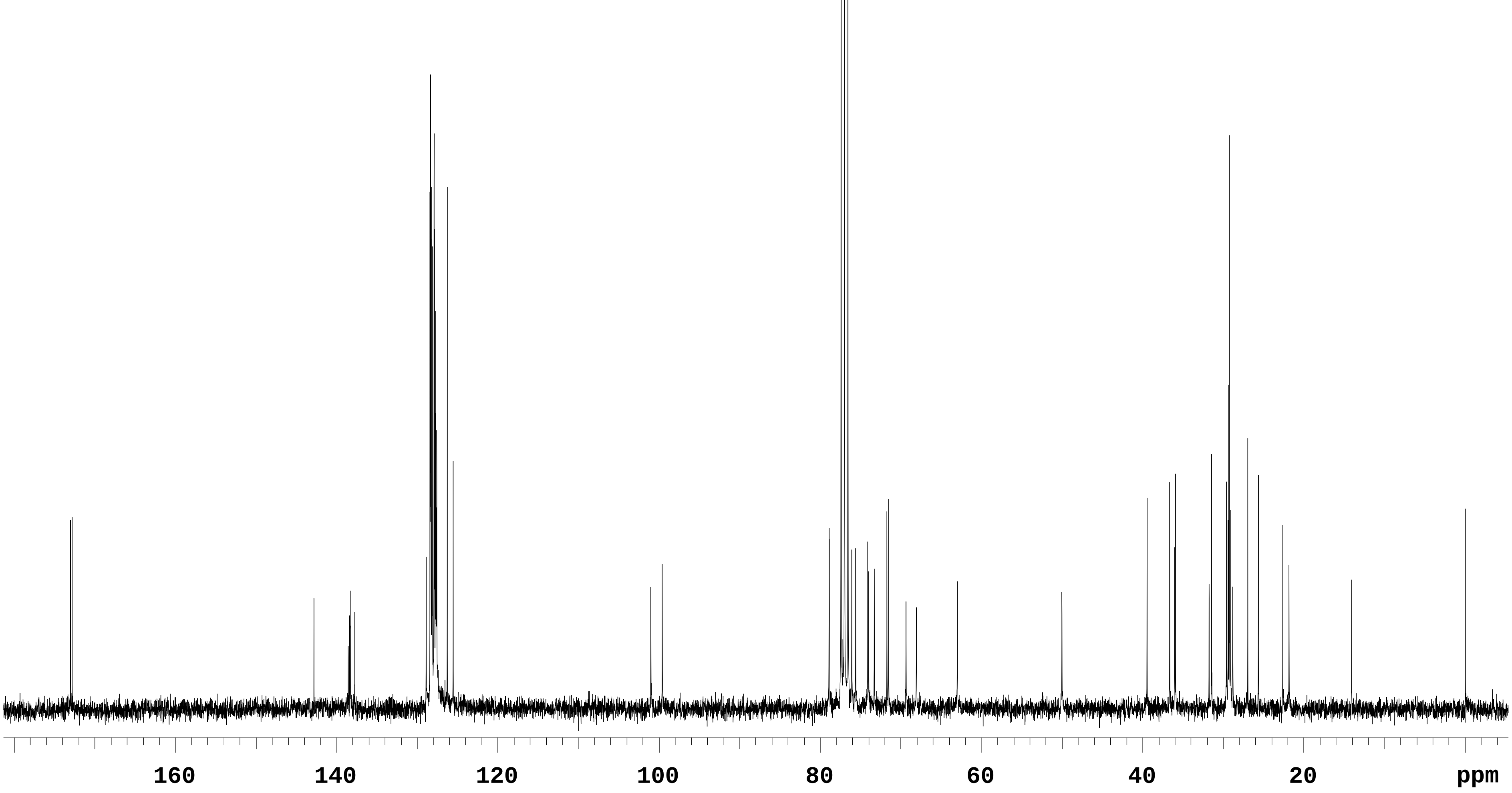

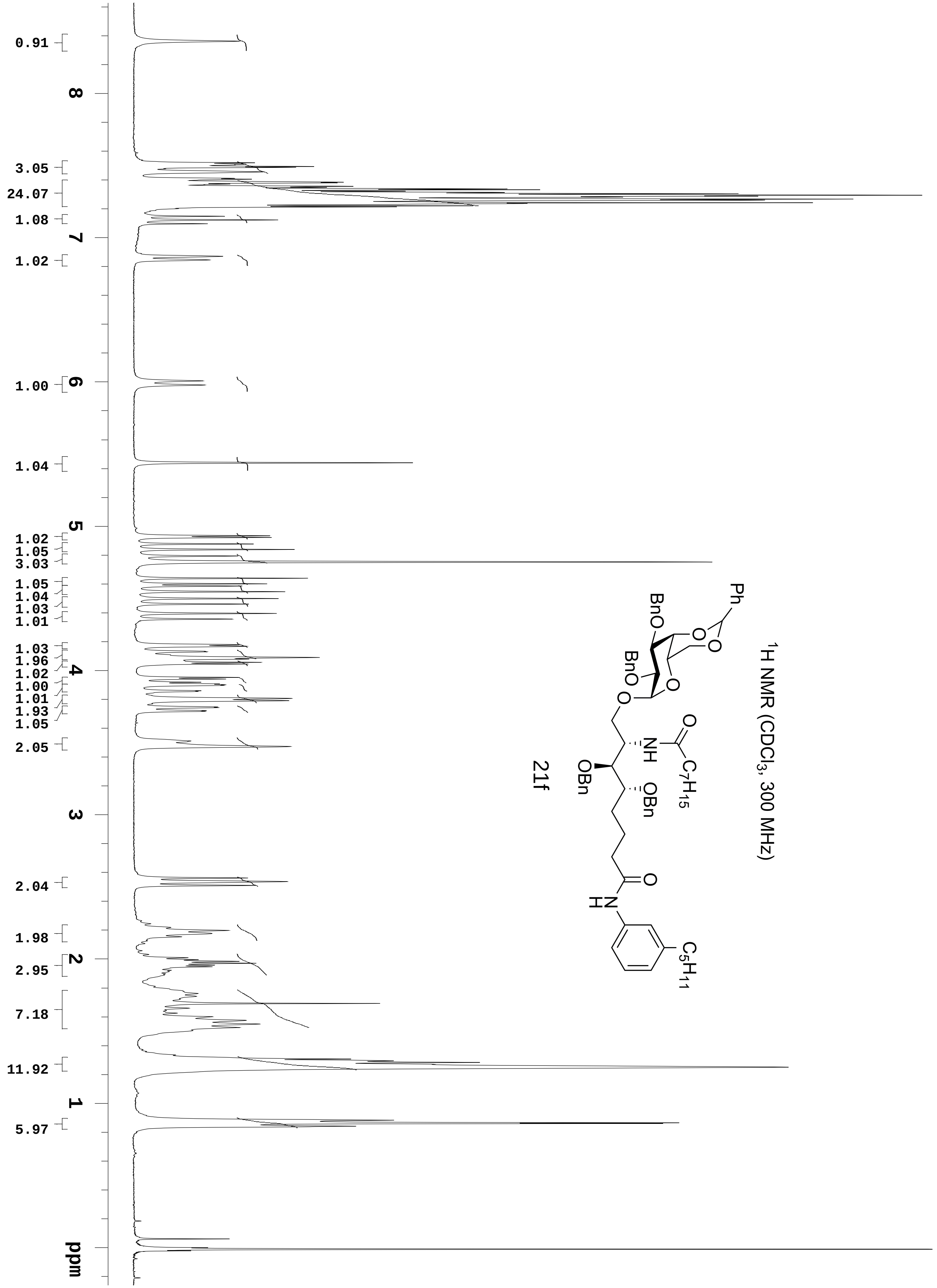
1H NMR (CDCl3, 300 MHz)
21f
Ph
BnO
BnO
NH
C7H15
OBn
OBn
N
H
C5H11
0.91
3.05
24.07
1.08
1.02
1.00
1.04
1.02
1.05
3.03
1.05
1.04
1.03
1.01
1.03
1.96
1.02
1.00
1.01
1.93
1.05
2.05
2.04
1.98
2.95
7.18
11.92
5.97
8
7
6
5
4
3
2
1
ppm

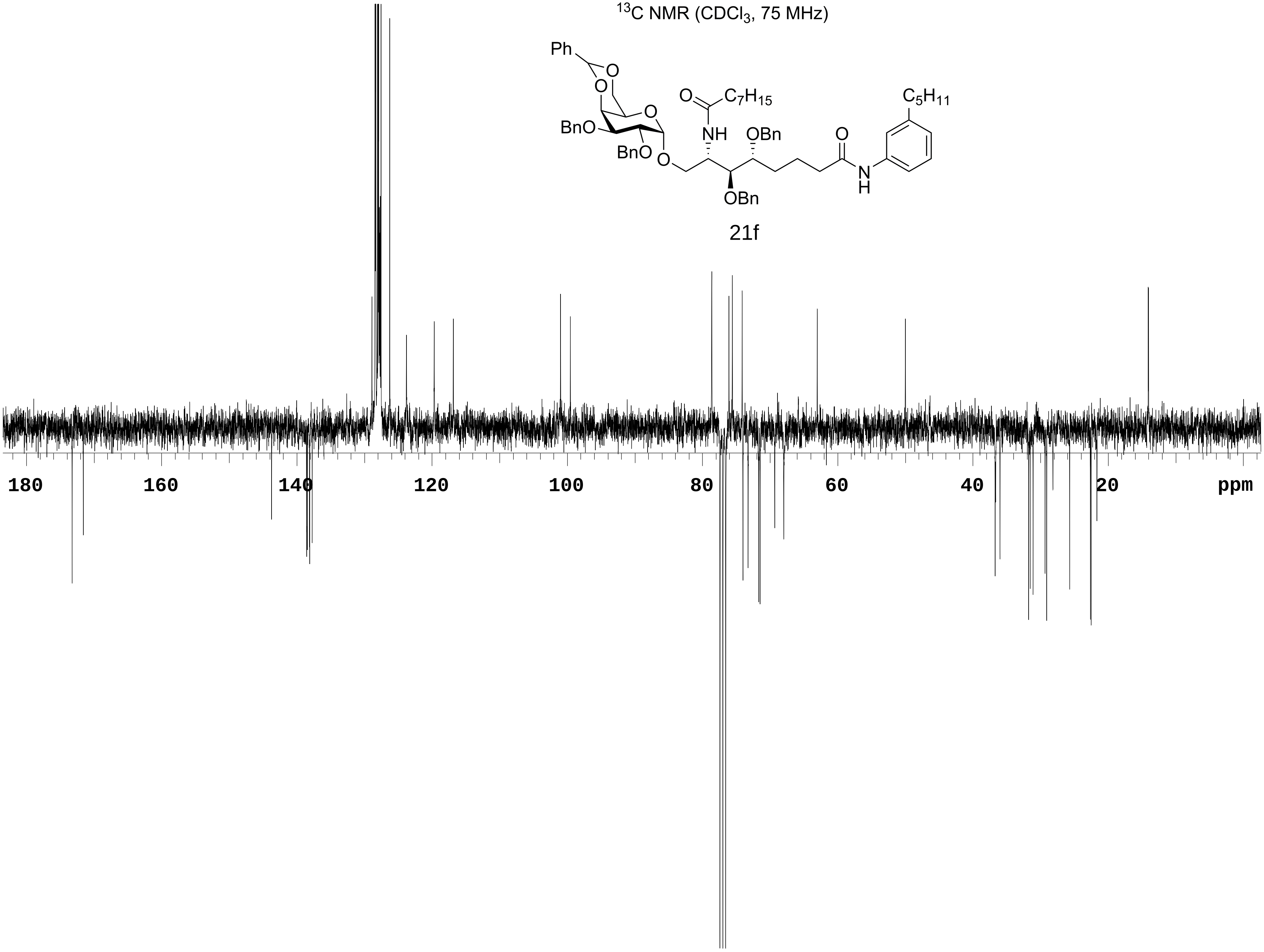

^{13}C NMR ($CDCl_3$, 75 MHz)
Ph
O
O
O
BnO
BnO
O
O
C_7H_{15}
NH
OBn
OBn
O
N
H
C_5H_{11}
21f
180
160
140
120
100
80
60
40
20
ppm

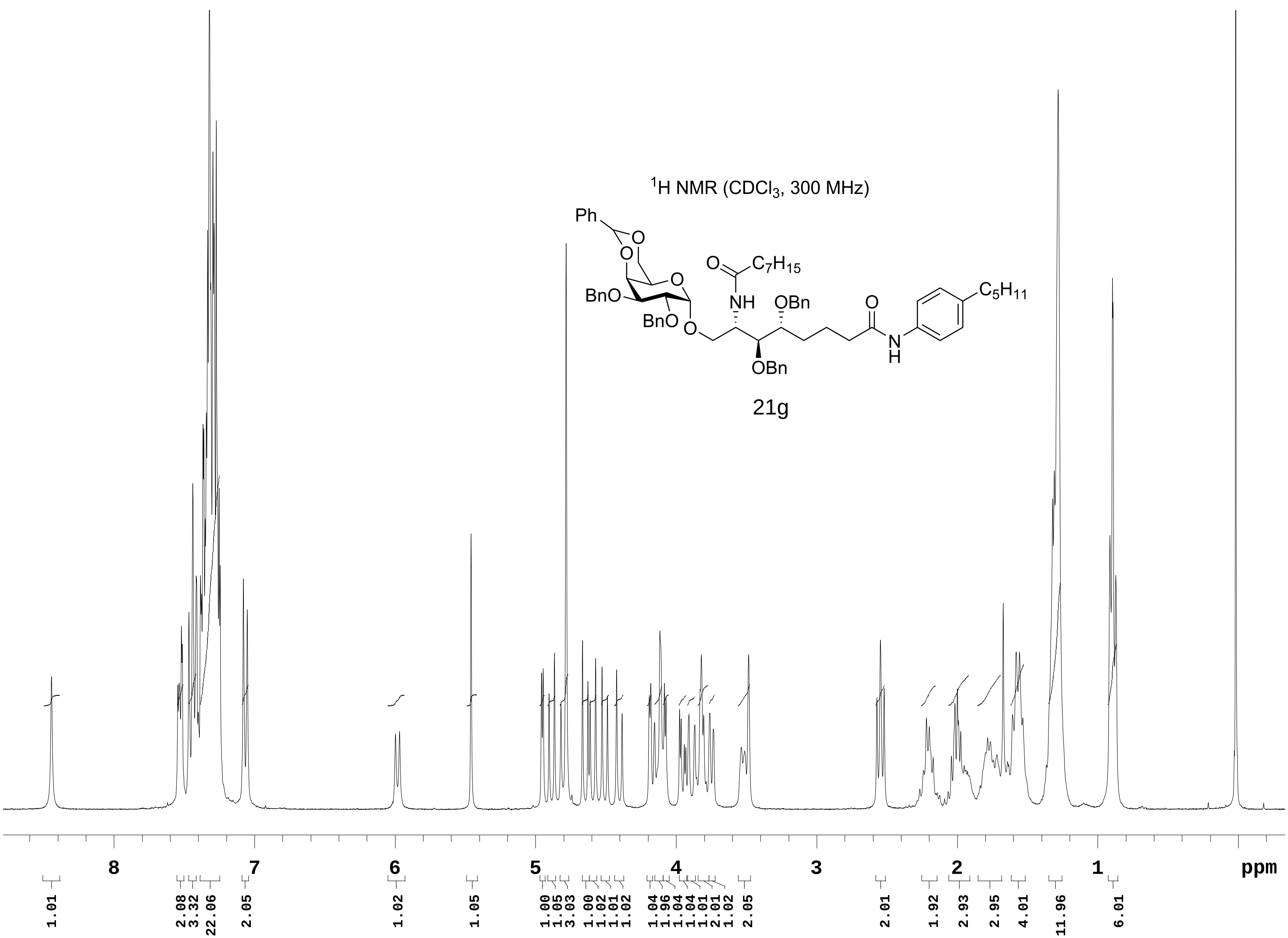

^{1}H NMR ($CDCl_3$, 300 MHz)
Ph
BnO
BnO
C_7H_{15}
NH
OBn
OBn
C_5H_{11}
21g
8
7
6
5
4
3
2
1
ppm
1.01
2.08
3.32
22.06
2.05
1.02
1.05
1.00
1.05
3.03
1.00
1.02
1.01
1.02
1.04
1.96
1.04
1.04
1.01
2.01
1.02
2.05
2.01
1.92
2.93
2.95
4.01
11.96
6.01

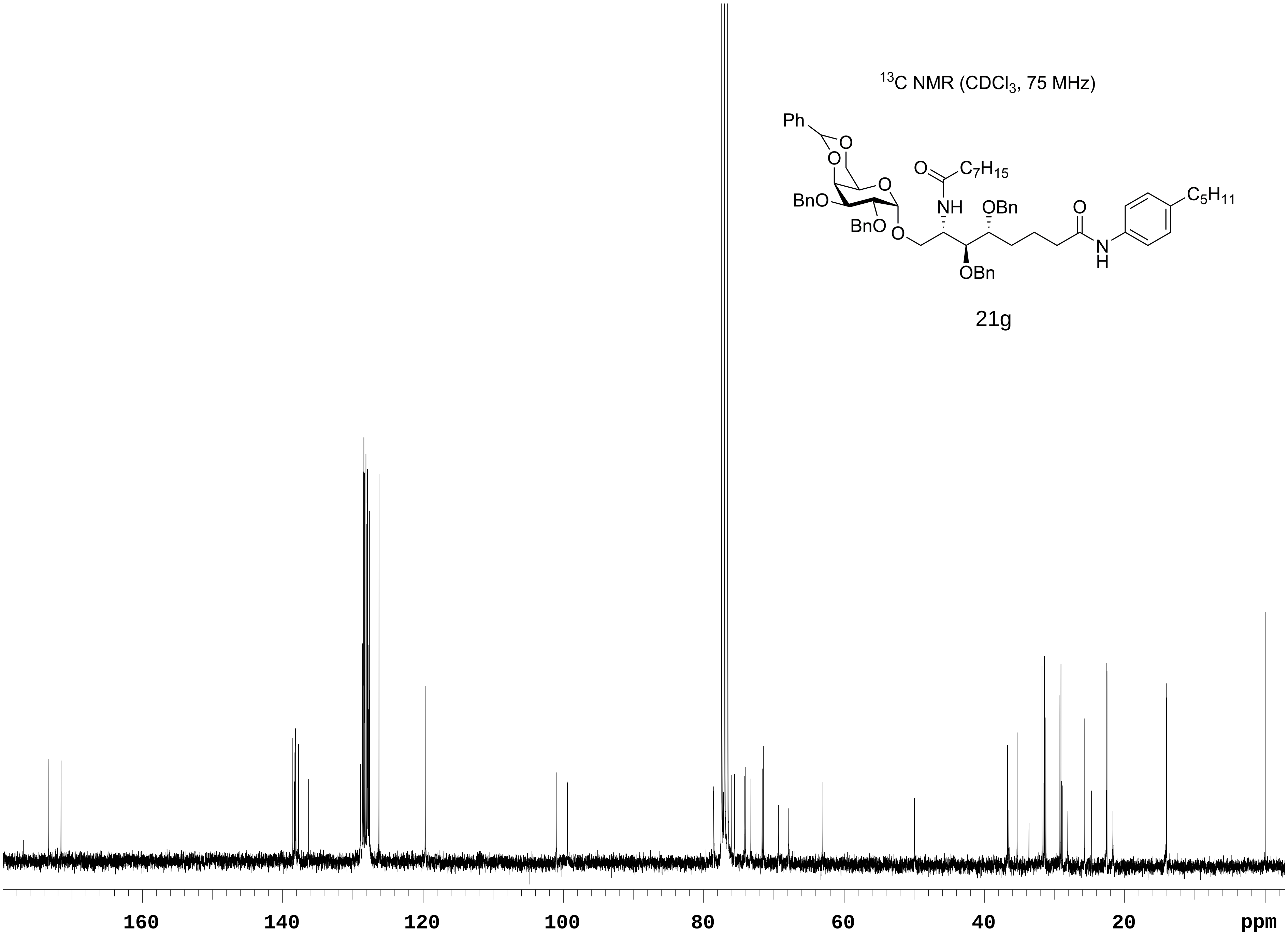

13C NMR (CDCl3, 75 MHz)
Ph
O
O
O
C7H15
BnO
O
NH
OBn
O
C5H11
BnO
O
N
H
OBn
21g
160
140
120
100
80
60
40
20
ppm

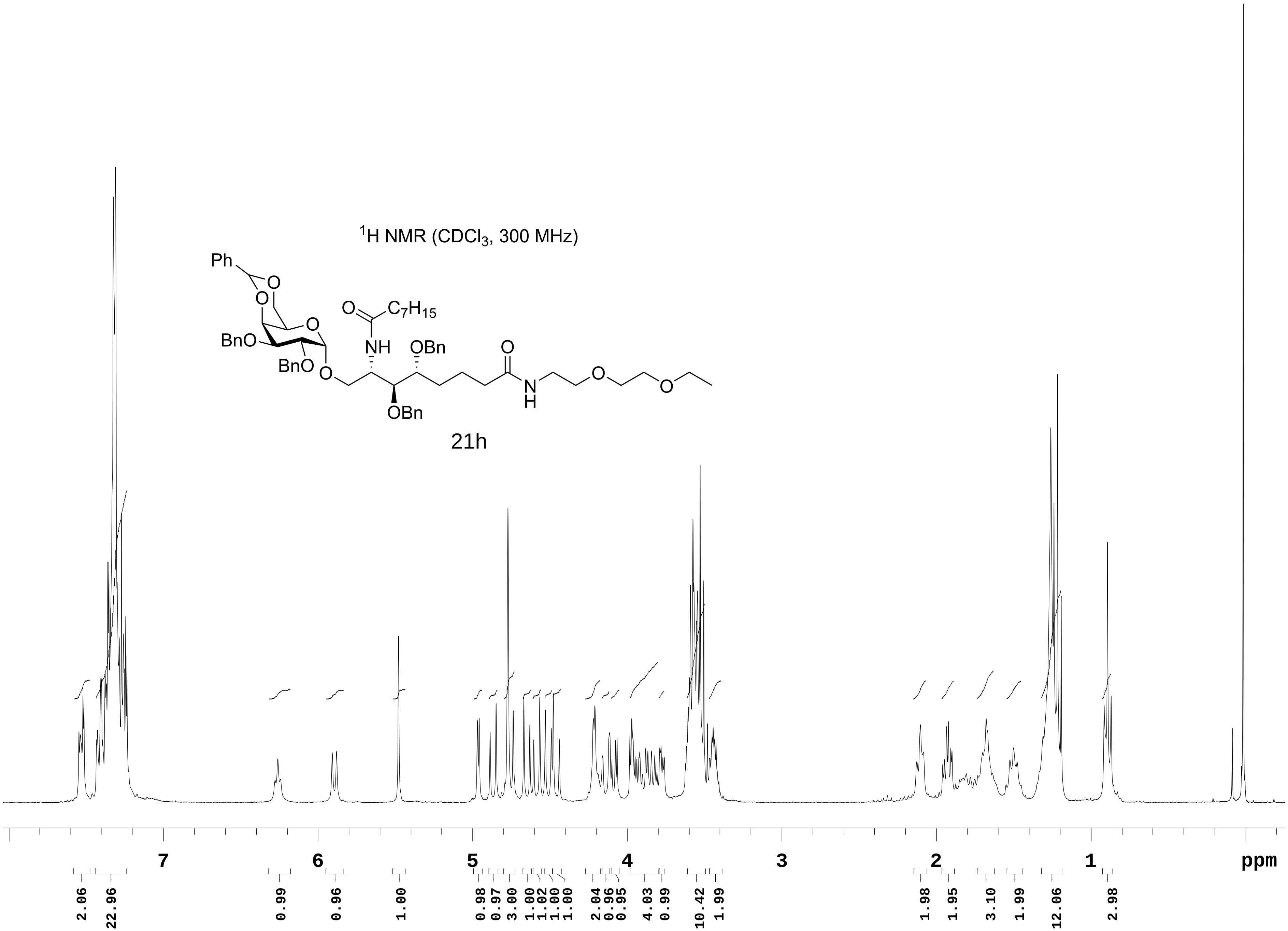

1H NMR (CDCl3, 300 MHz)
Ph
BnO
BnO
C7H15
NH
OBn
OBn
21h
7
6
5
4
3
2
1
ppm
2.06
22.96
0.99
0.96
1.00
0.98
0.97
3.00
1.00
1.02
1.00
1.00
2.04
0.96
0.95
4.03
0.99
10.42
1.99
1.98
1.95
3.10
1.99
12.06
2.98

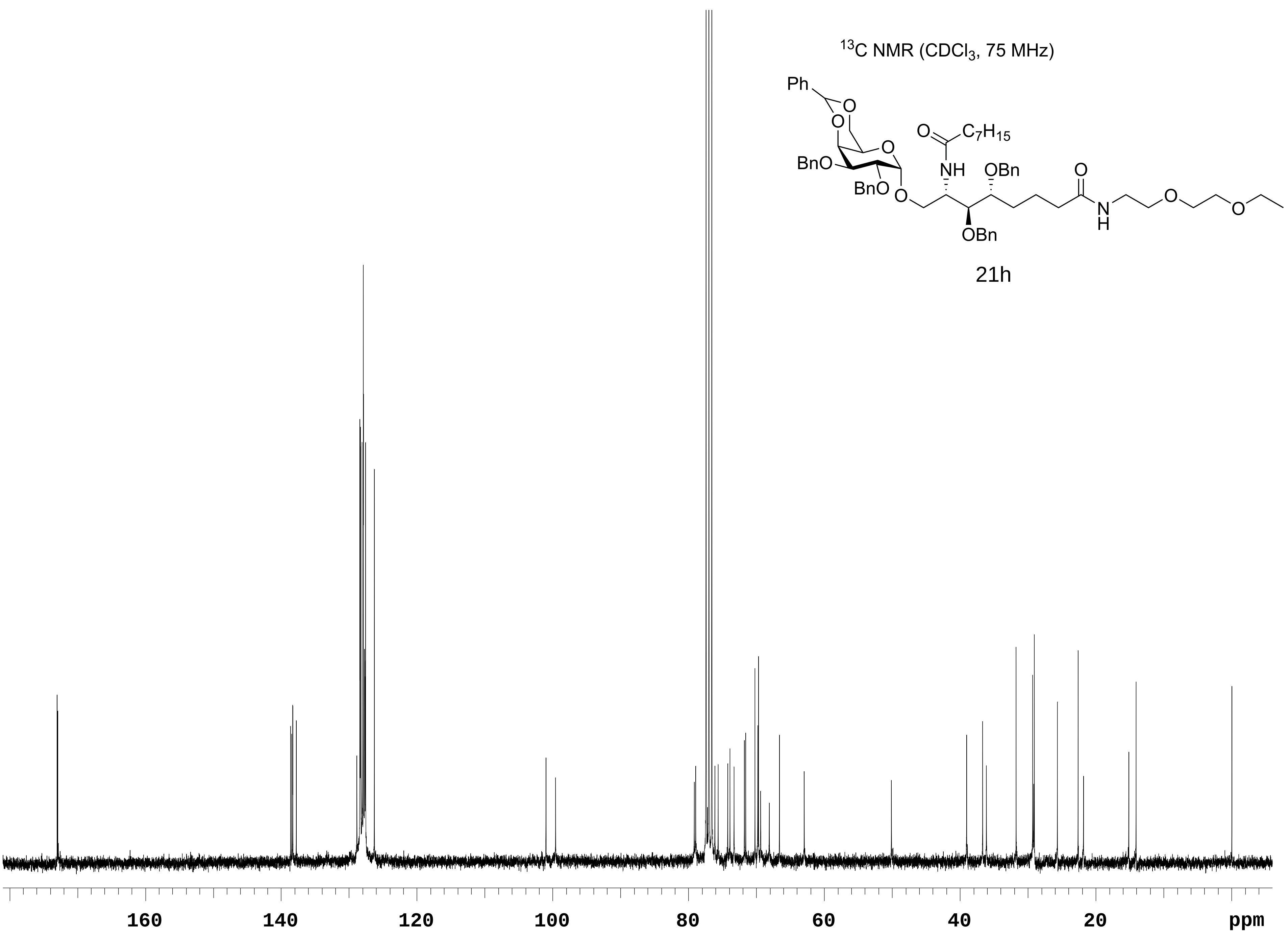
^{13}C NMR ($CDCl_3$, 75 MHz)
Ph
O
O
O
BnO
BnO
O
O
C_7H_{15}
NH
OBn
OBn
O
N
H
O
O
21h
160
140
120
100
80
60
40
20
ppm

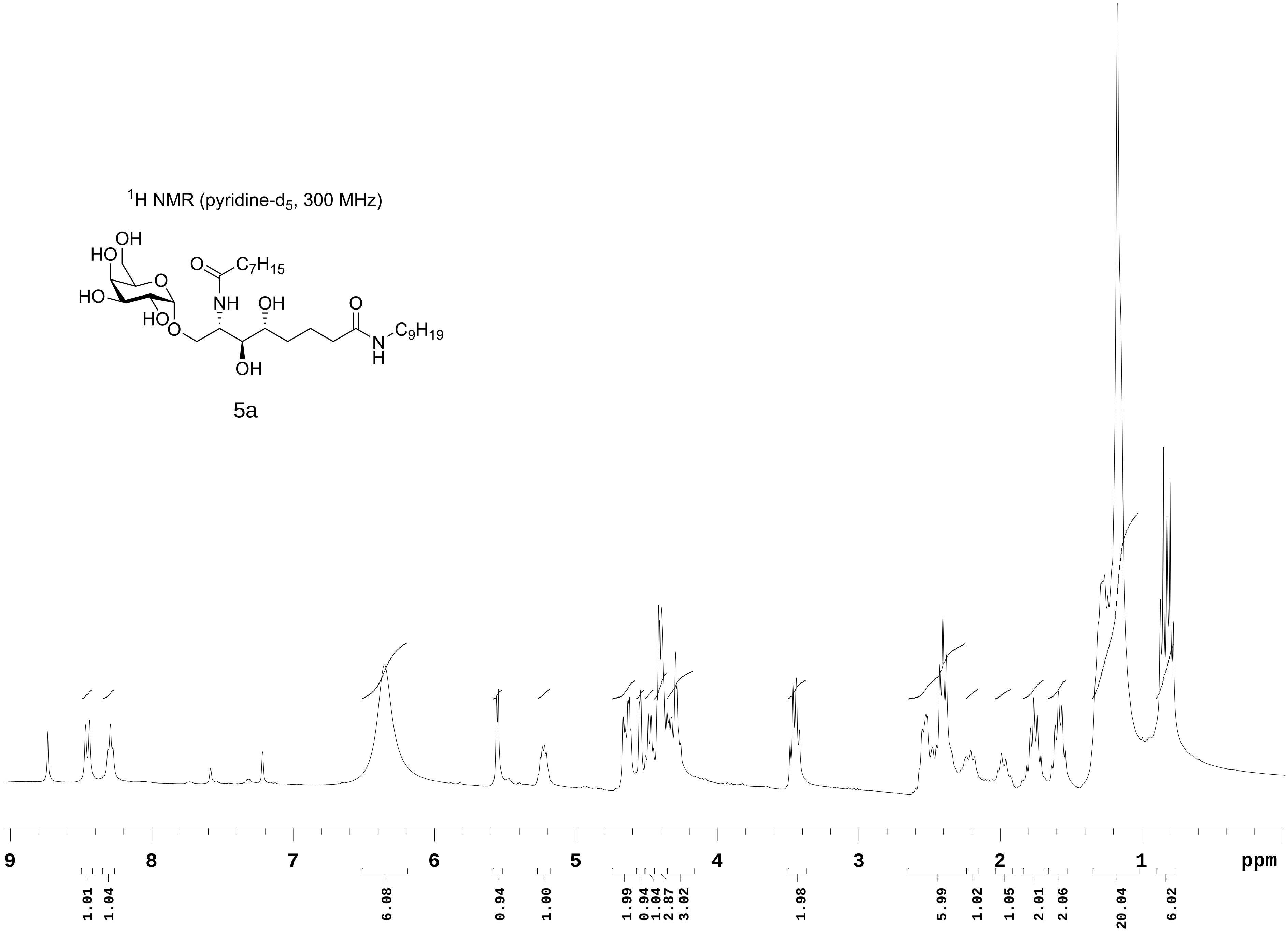

1H NMR (pyridine-d5, 300 MHz)
5a
9 8 7 6 5 4 3 2 1 ppm
1.01 1.04 6.08 0.94 1.00 1.99 0.94 1.04 2.87 3.02 1.98 5.99 1.02 1.05 2.01 2.06 20.04 6.02

^{13}C NMR (pyridine-d_5, 75 MHz)

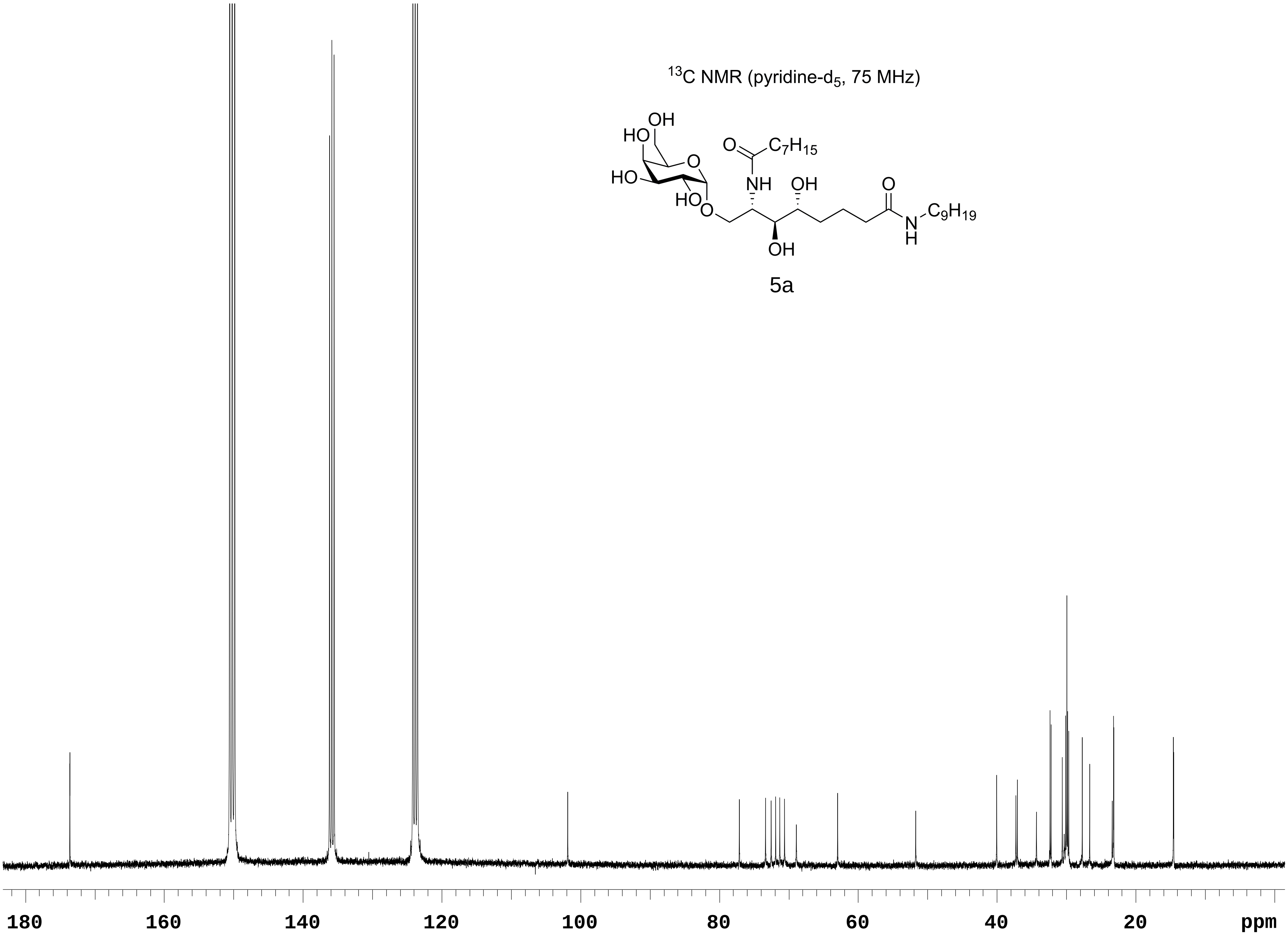

5a

^1H NMR (pyridine-d_5, 300 MHz)

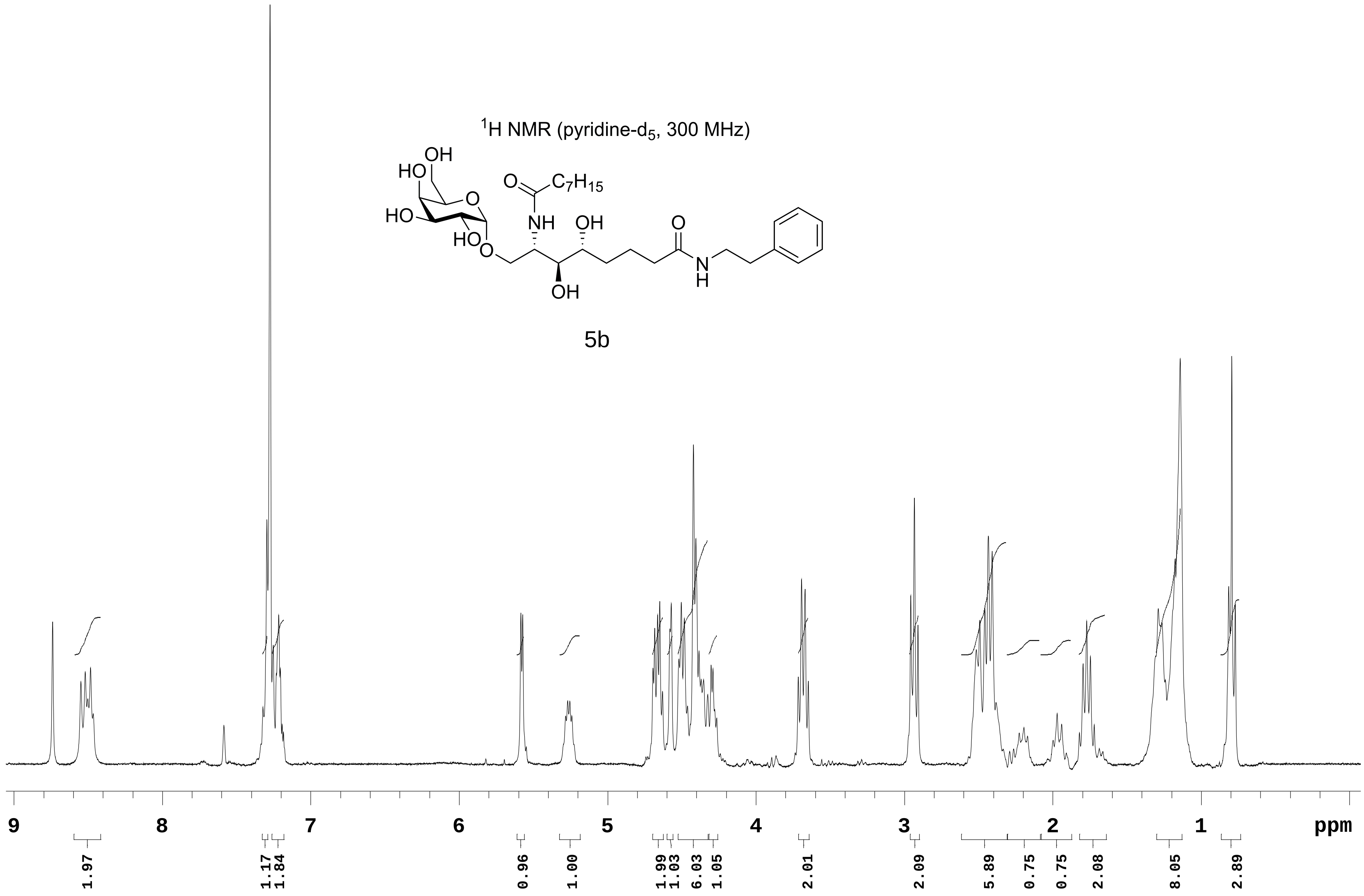

5b

^{13}C NMR (pyridine-d_5, 75 MHz)

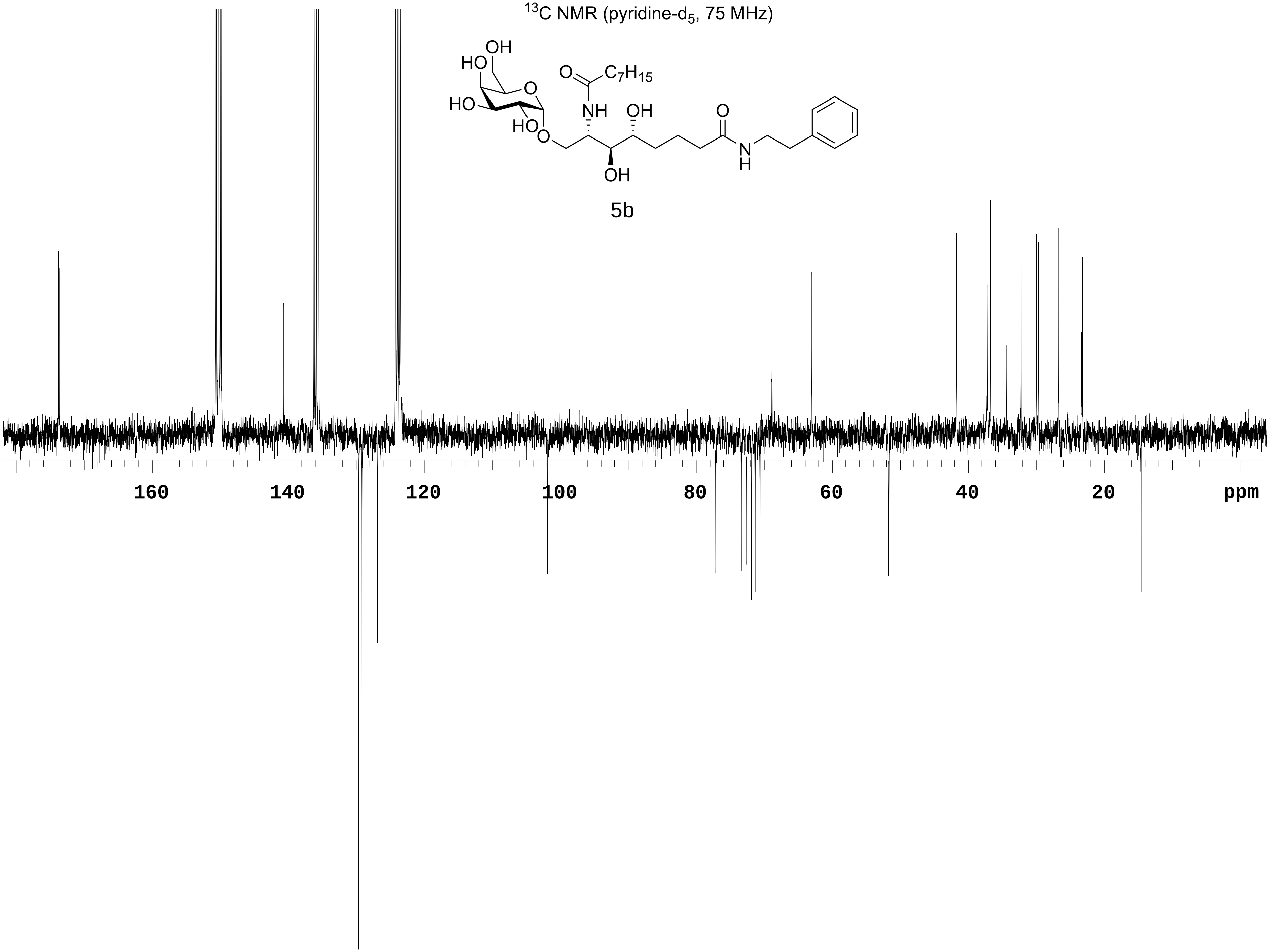

^{1}H NMR (pyridine-d_5, 300 MHz)

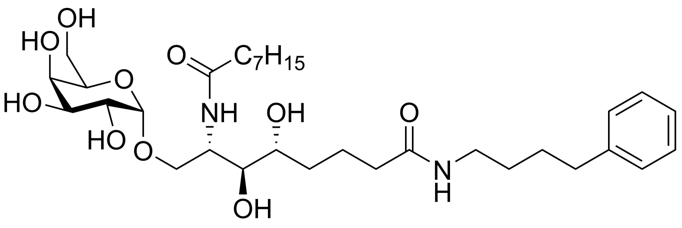

5c

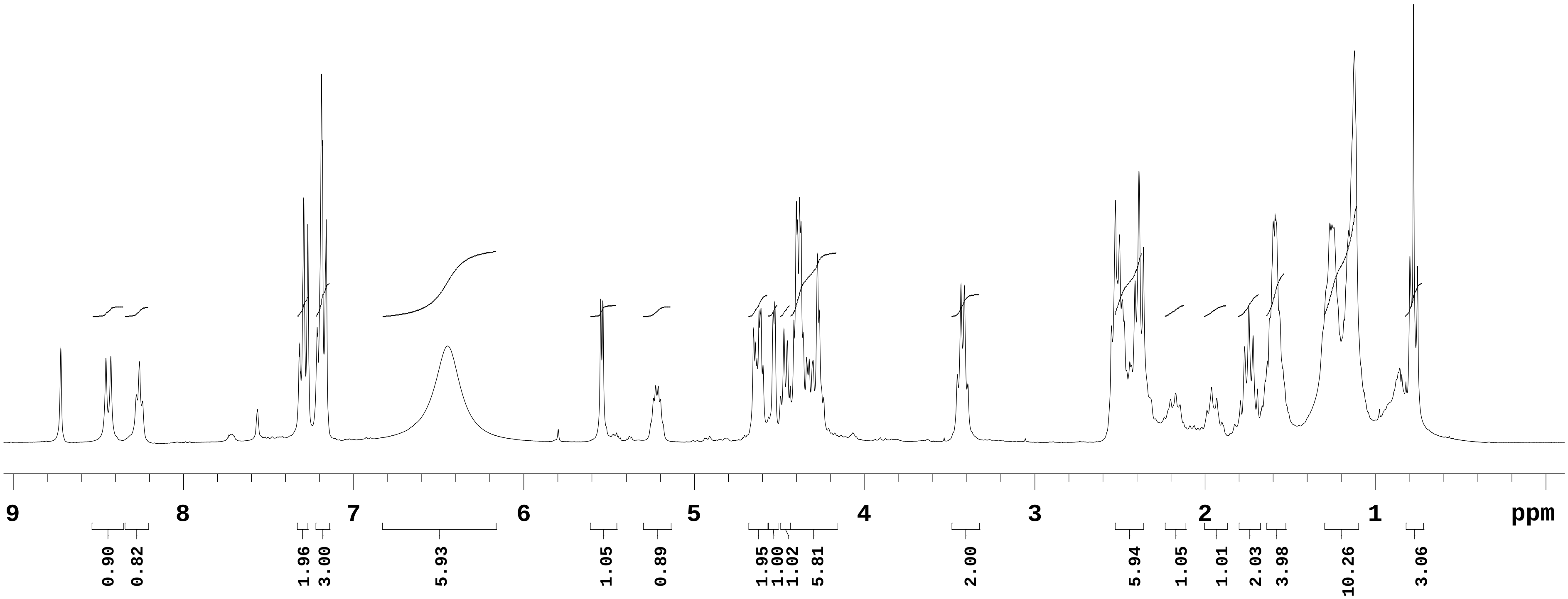

^{13}C NMR (pyridine-d_5, 75 MHz)

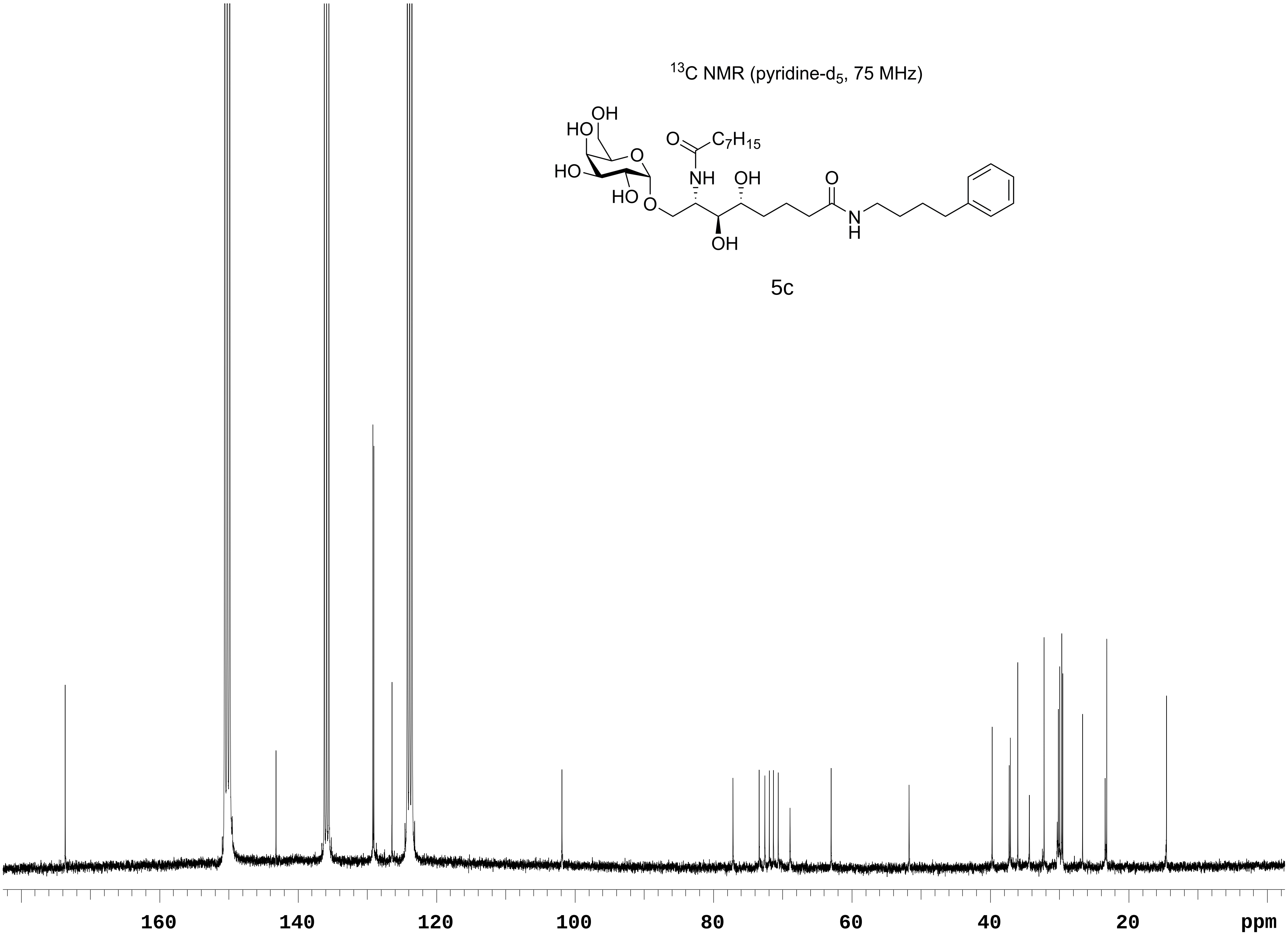

5c

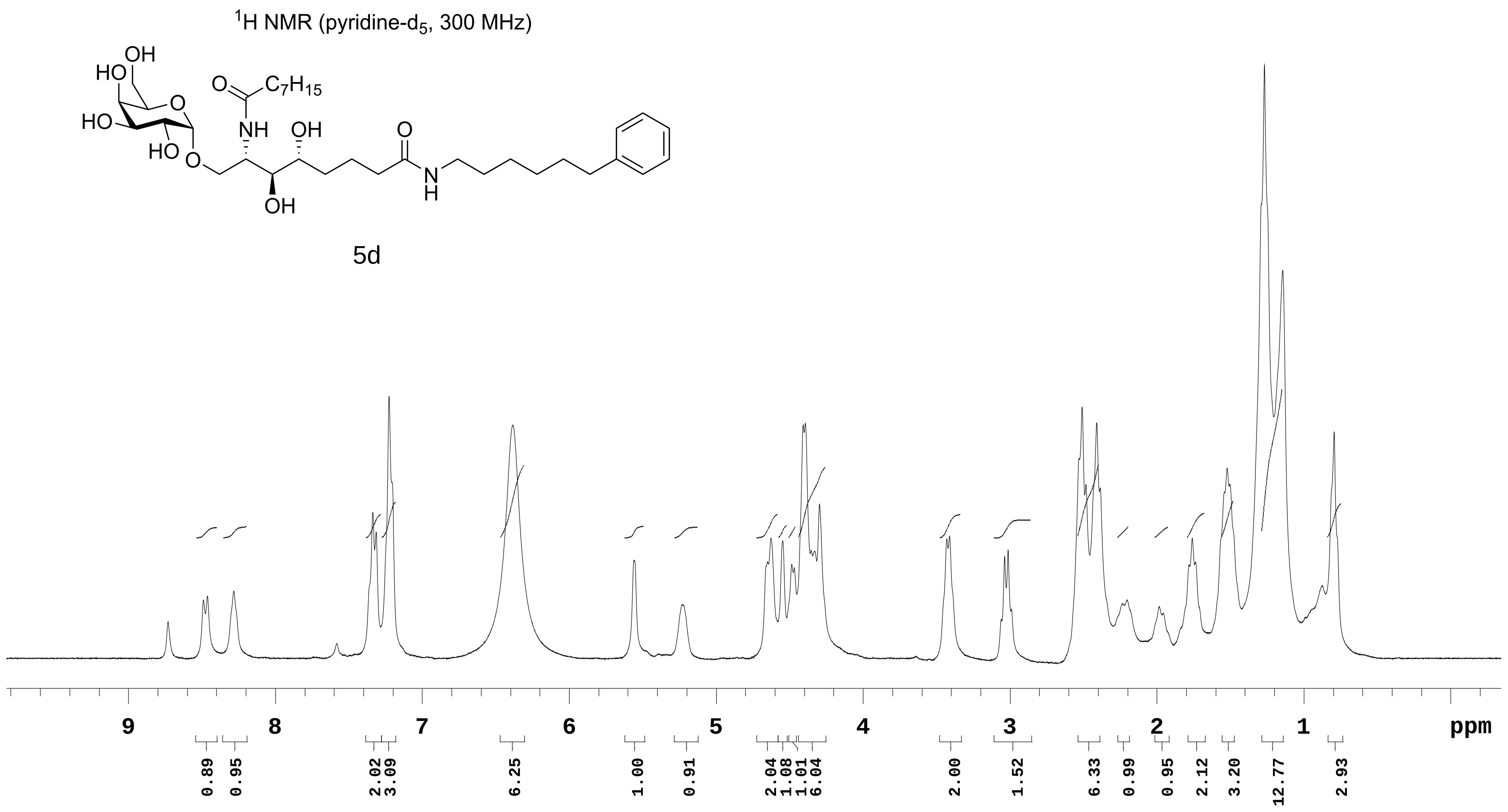

^1H NMR (pyridine-d_5, 300 MHz)
5d
9
8
7
6
5
4
3
2
1
ppm
0.89
0.95
2.02
3.09
6.25
1.00
0.91
2.04
1.08
1.01
6.04
2.00
1.52
6.33
0.99
0.95
2.12
3.20
12.77
2.93

^{13}C NMR (pyridine-d_5, 75 MHz)

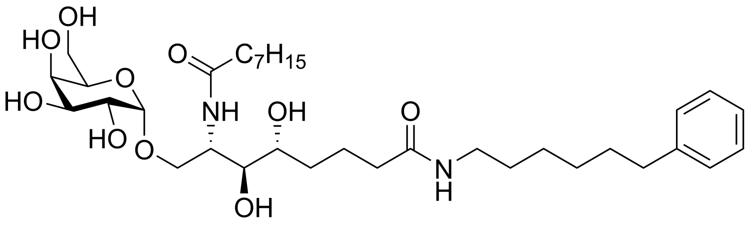

5d

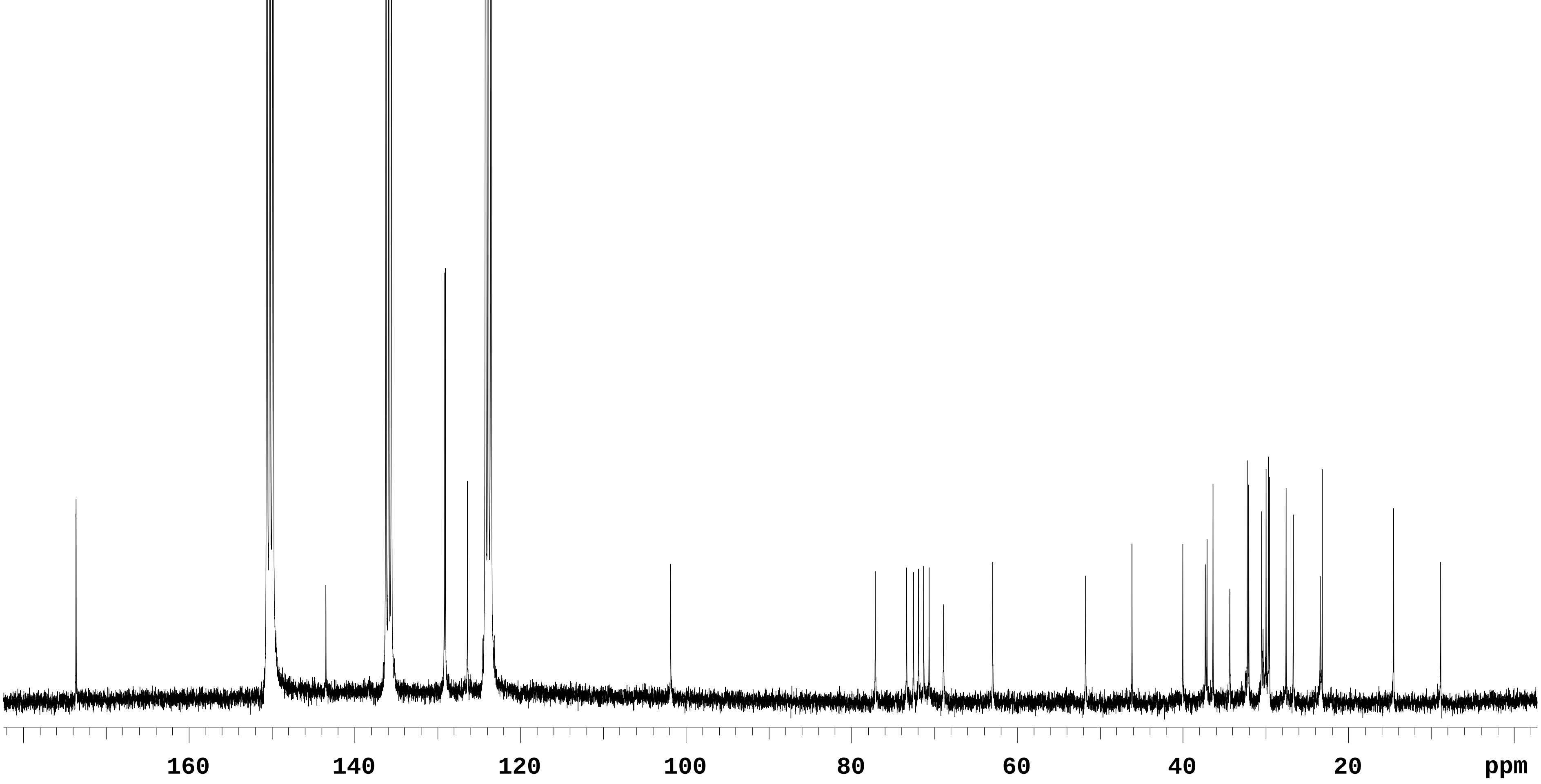

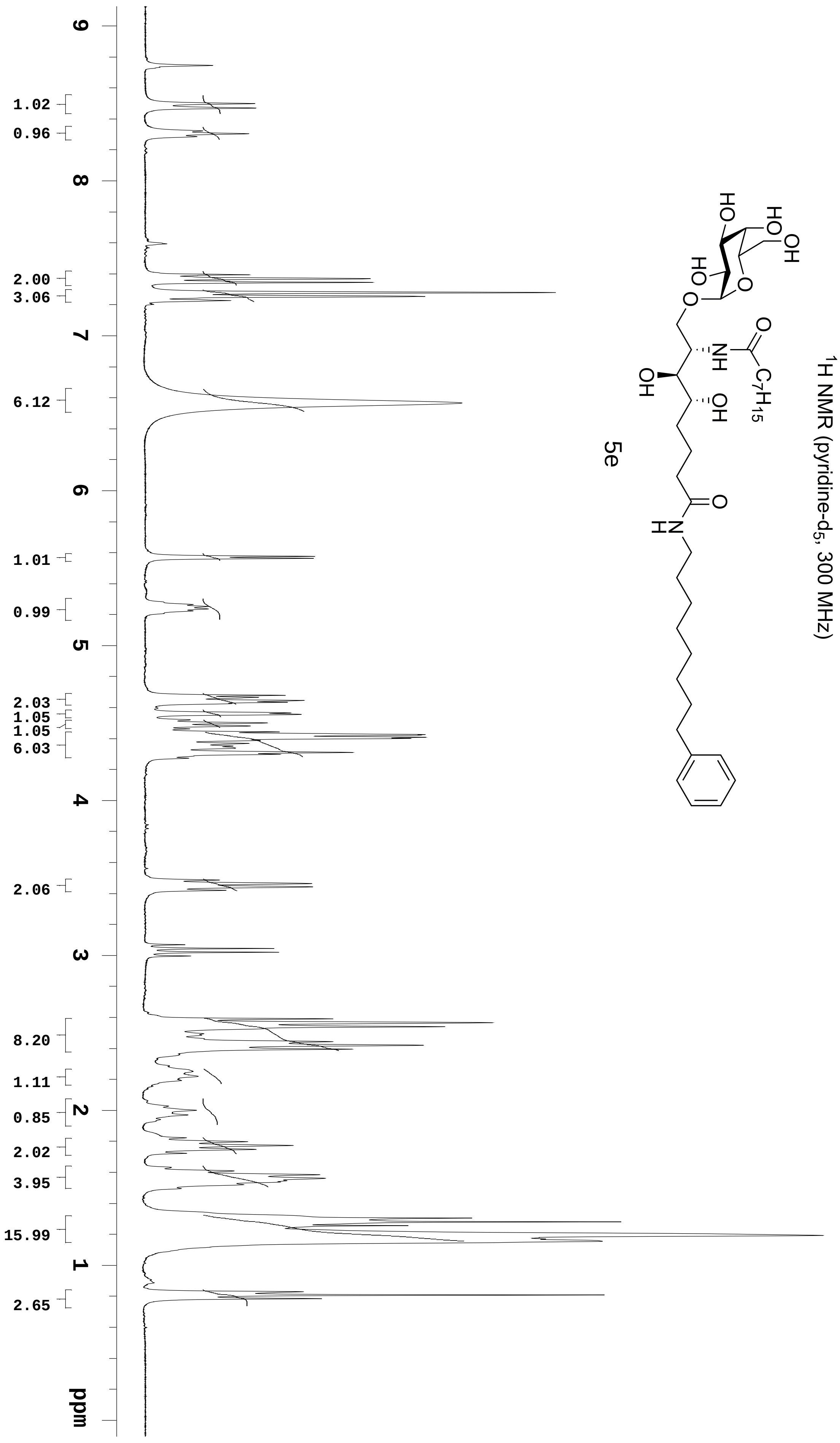

^{1}H NMR (pyridine-d$_5$, 300 MHz)
5e
HO
OH
O
NH
C_7H_{15}
N
H
ppm
9
8
7
6
5
4
3
2
1
1.02
0.96
2.00
3.06
6.12
1.01
0.99
2.03
1.05
1.05
6.03
2.06
8.20
1.11
0.85
2.02
3.95
15.99
2.65

^{13}C NMR (pyridine-d$_5$, 75 MHz)

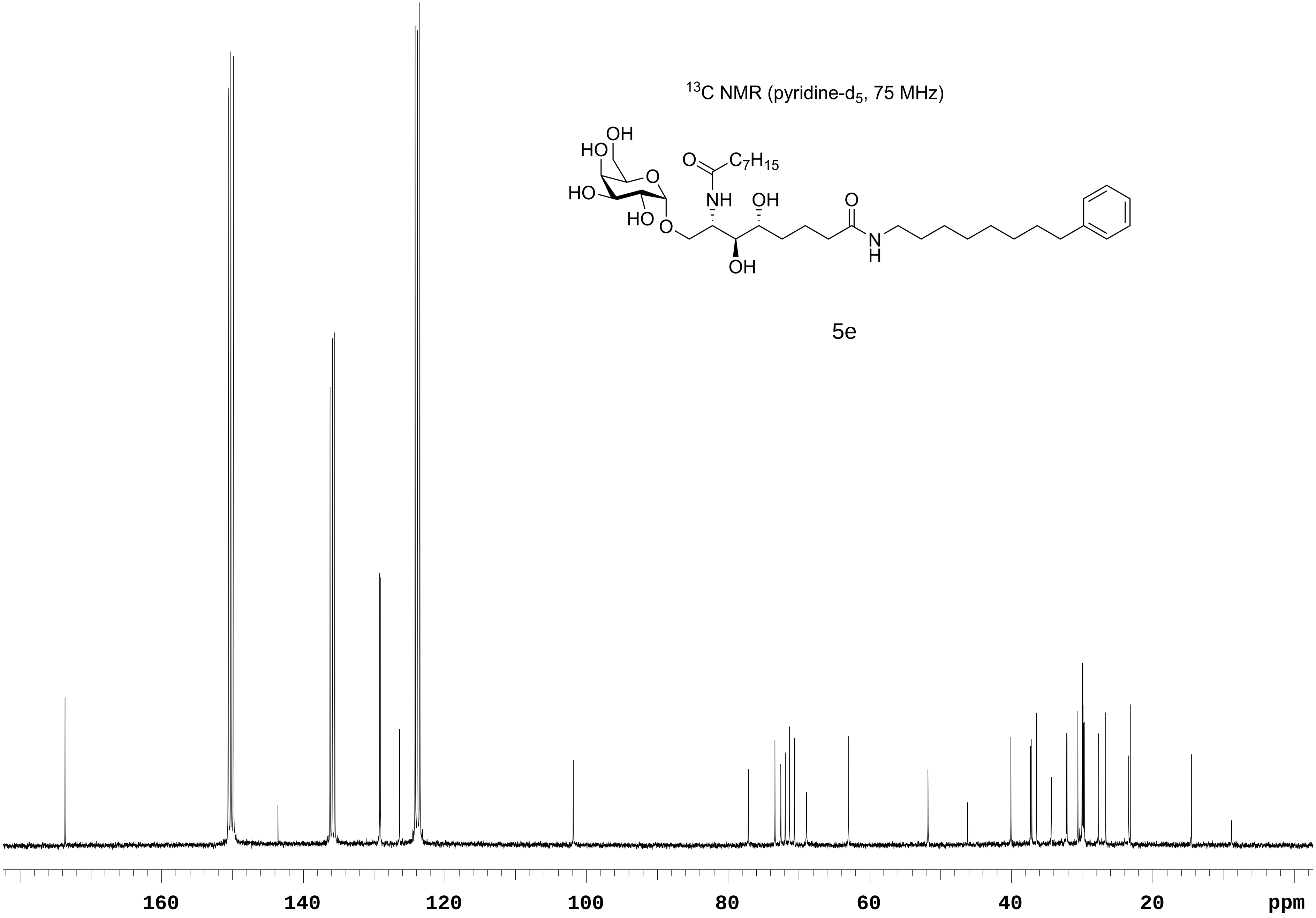

5e

^{1}H NMR (pyridine-d$_5$, 300 MHz)

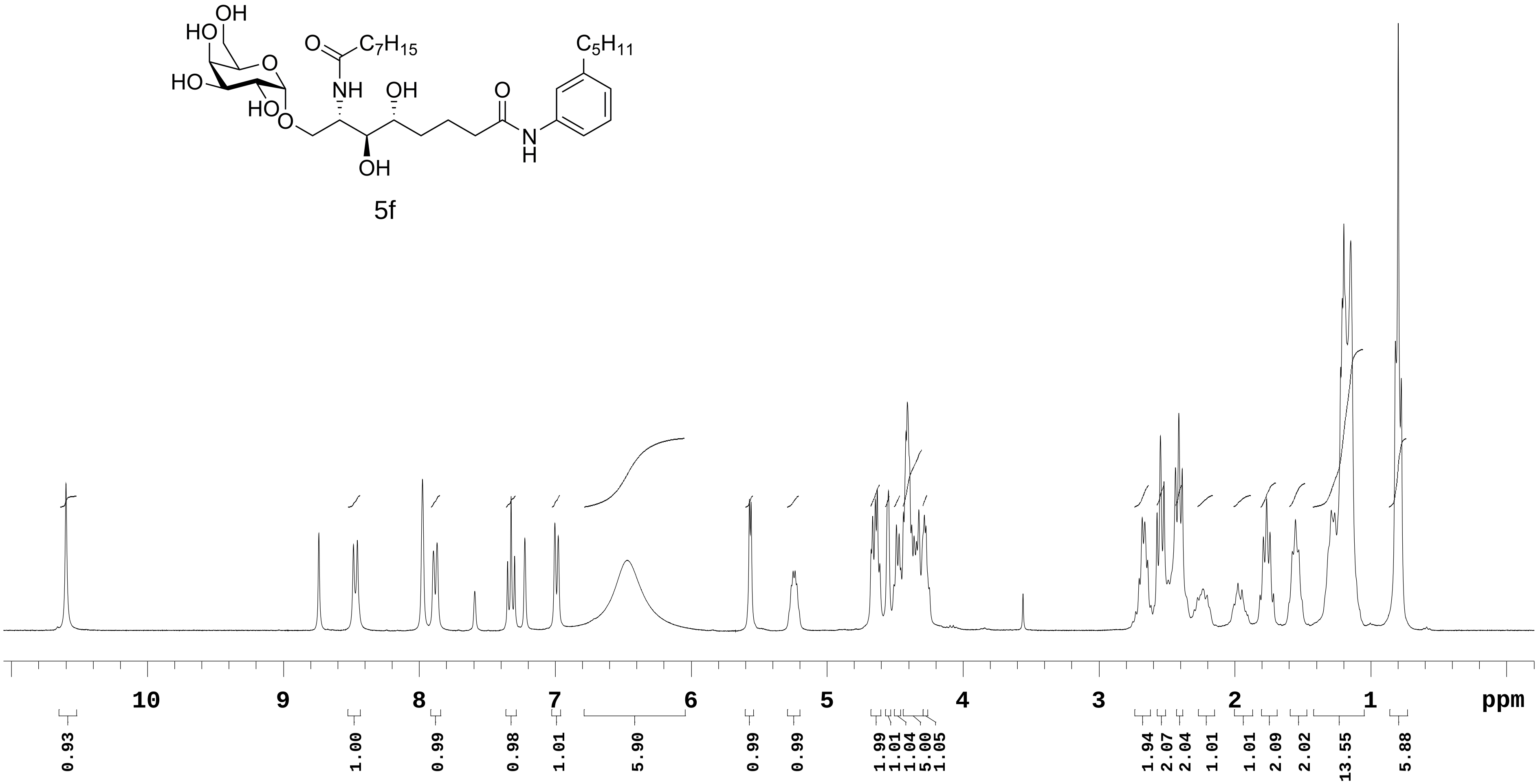

5f

^{13}C NMR (pyridine-d_5, 75 MHz)

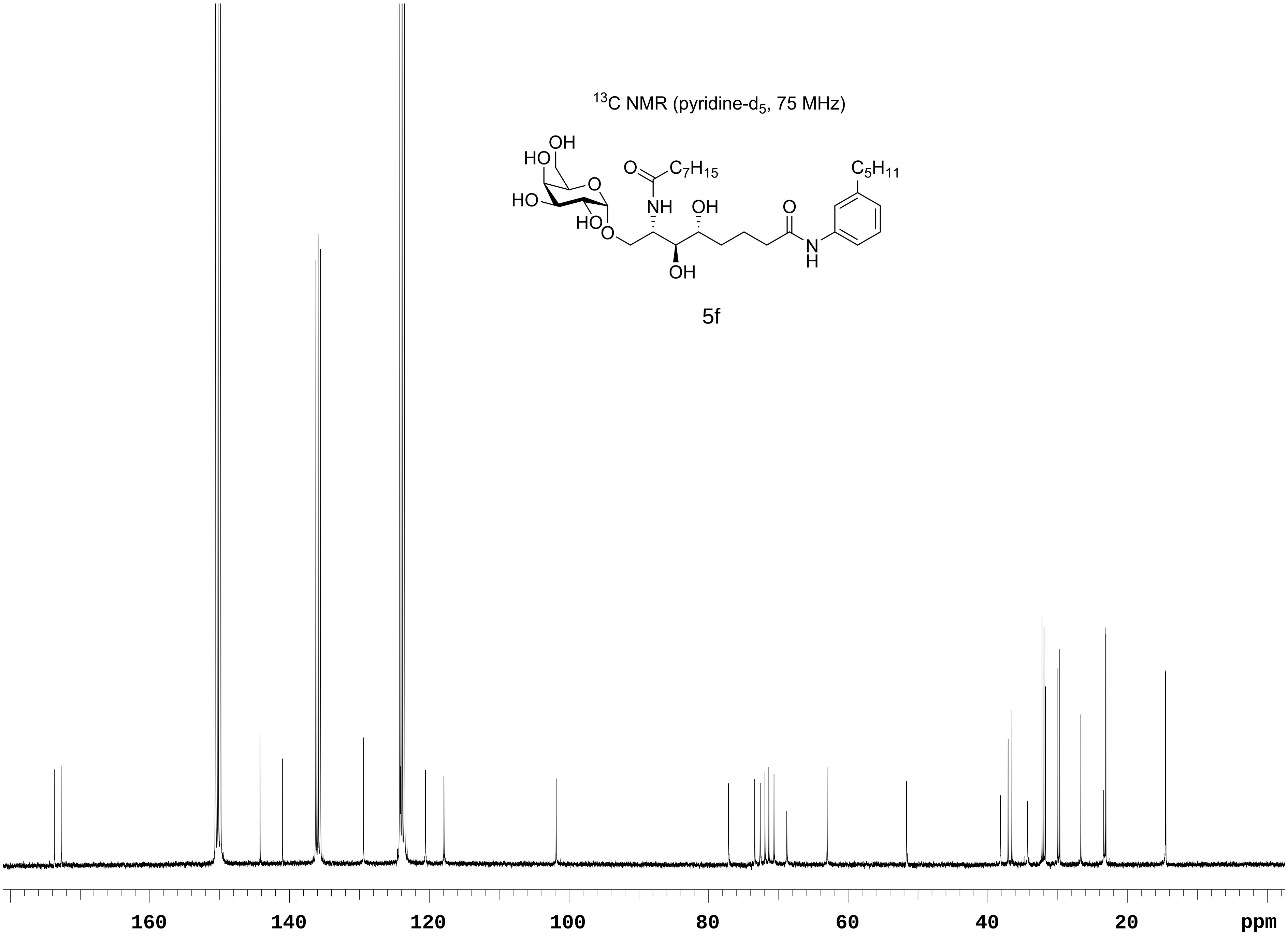

5f

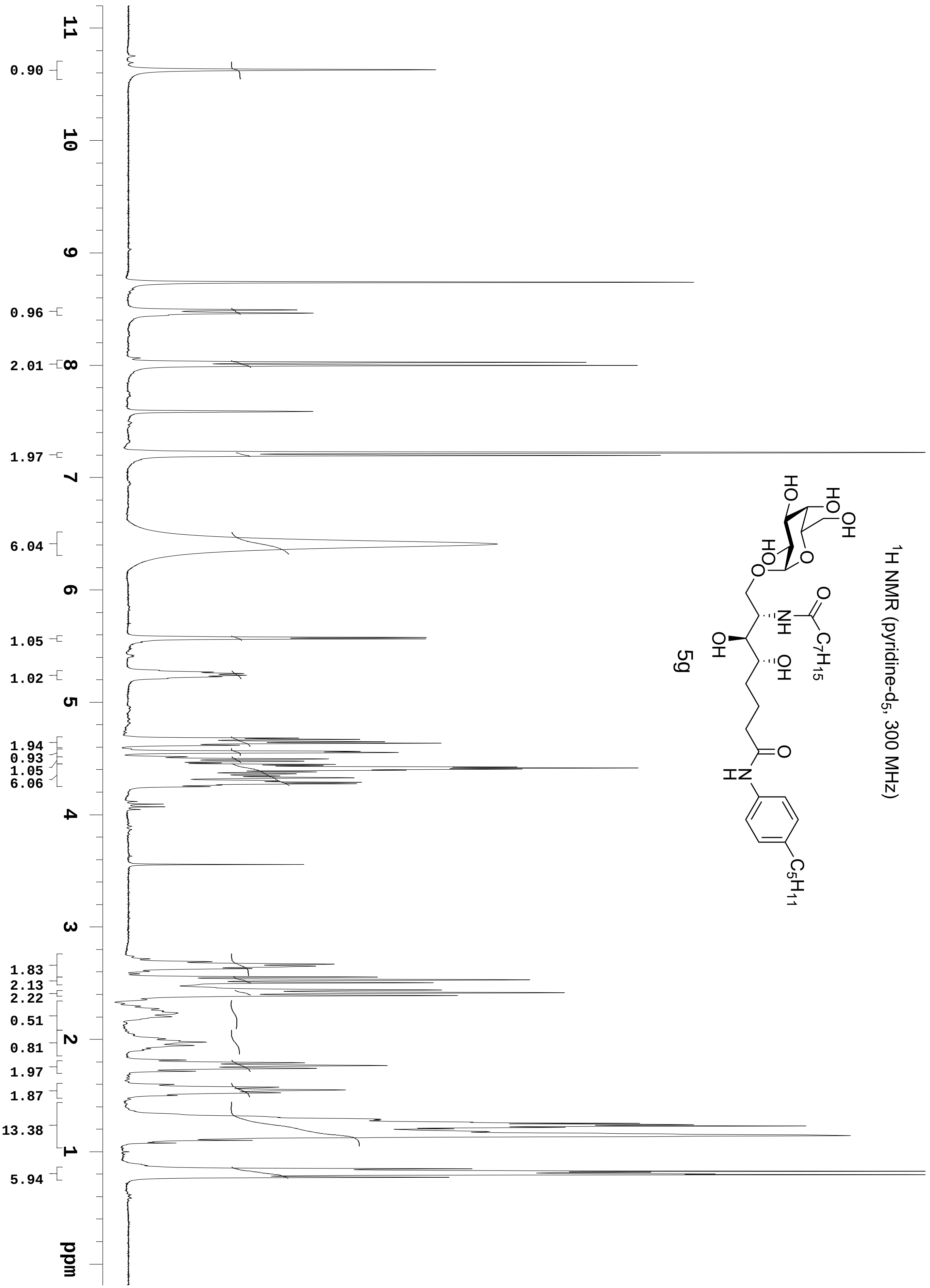

1H NMR (pyridine-d5, 300 MHz)
5g
C7H15
C5H11
11
10
9
8
7
6
5
4
3
2
1
ppm
0.90
0.96
2.01
1.97
6.04
1.05
1.02
1.94
0.93
1.05
6.06
1.83
2.13
2.22
0.51
0.81
1.97
1.87
13.38
5.94

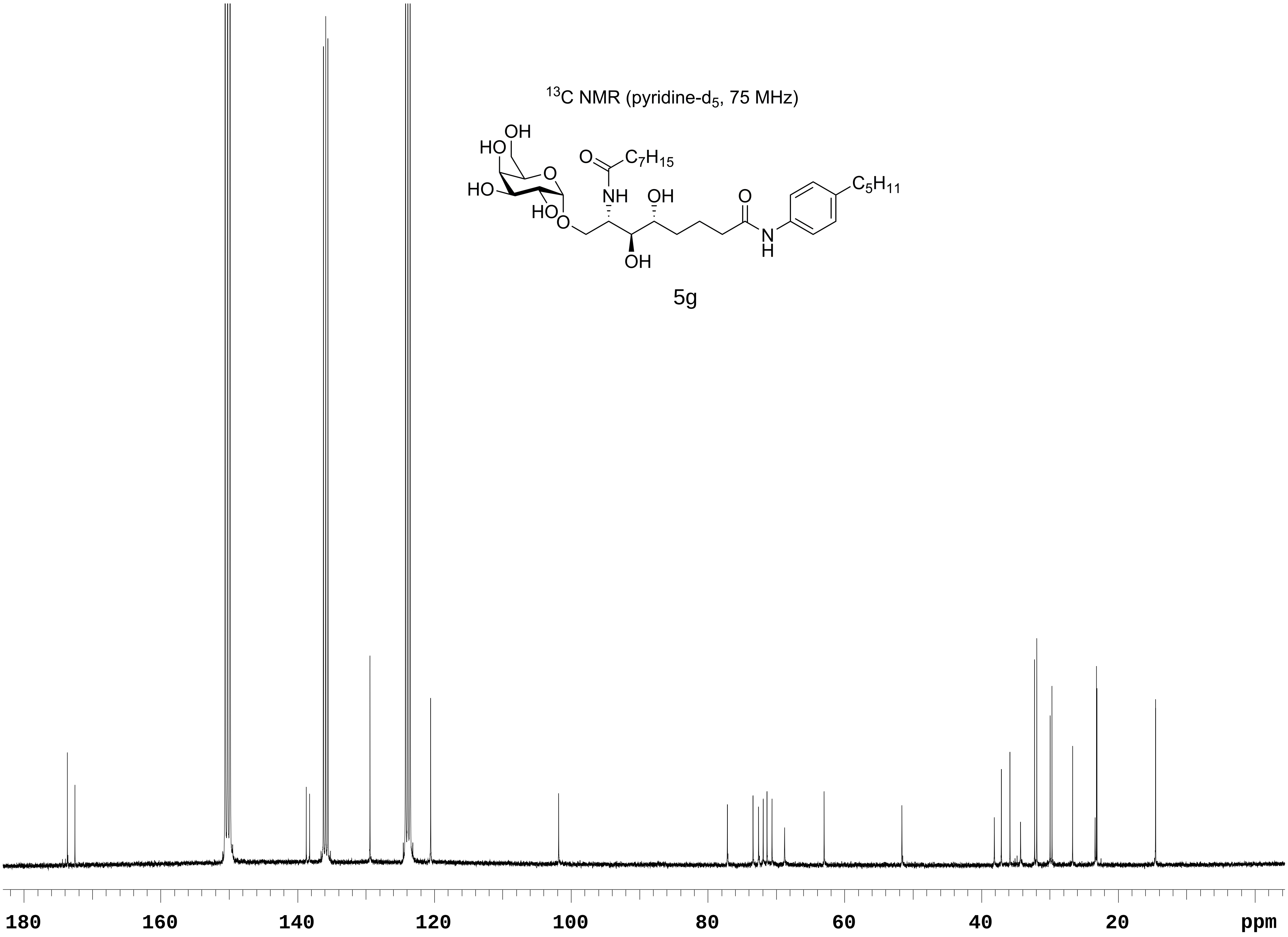
13C NMR (pyridine-d5, 75 MHz)
OH
HO
O
C7H15
O
HO
NH
OH
O
C5H11
HO
O
N
H
OH
5g
180
160
140
120
100
80
60
40
20
ppm

^{1}H NMR (pyridine-d_5, 300 MHz)

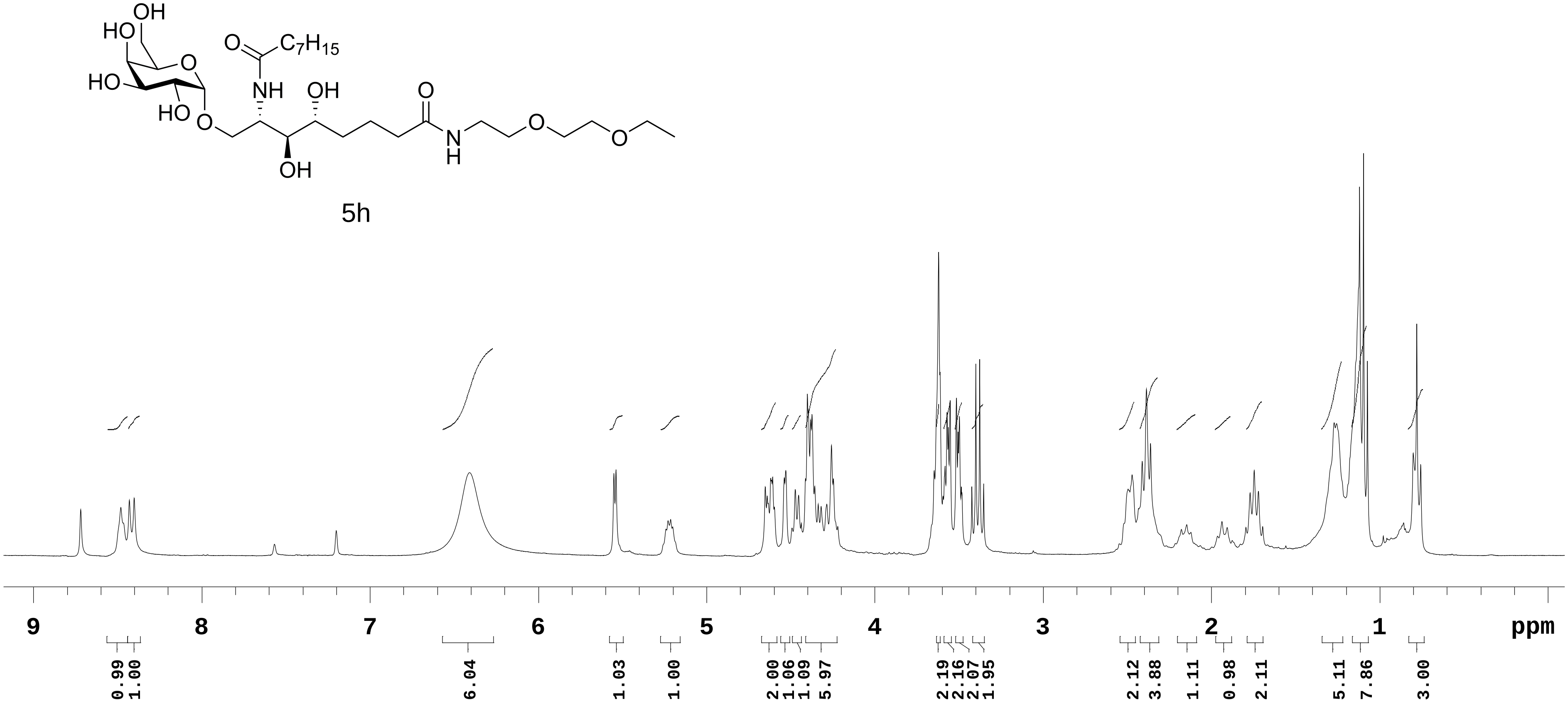

5h

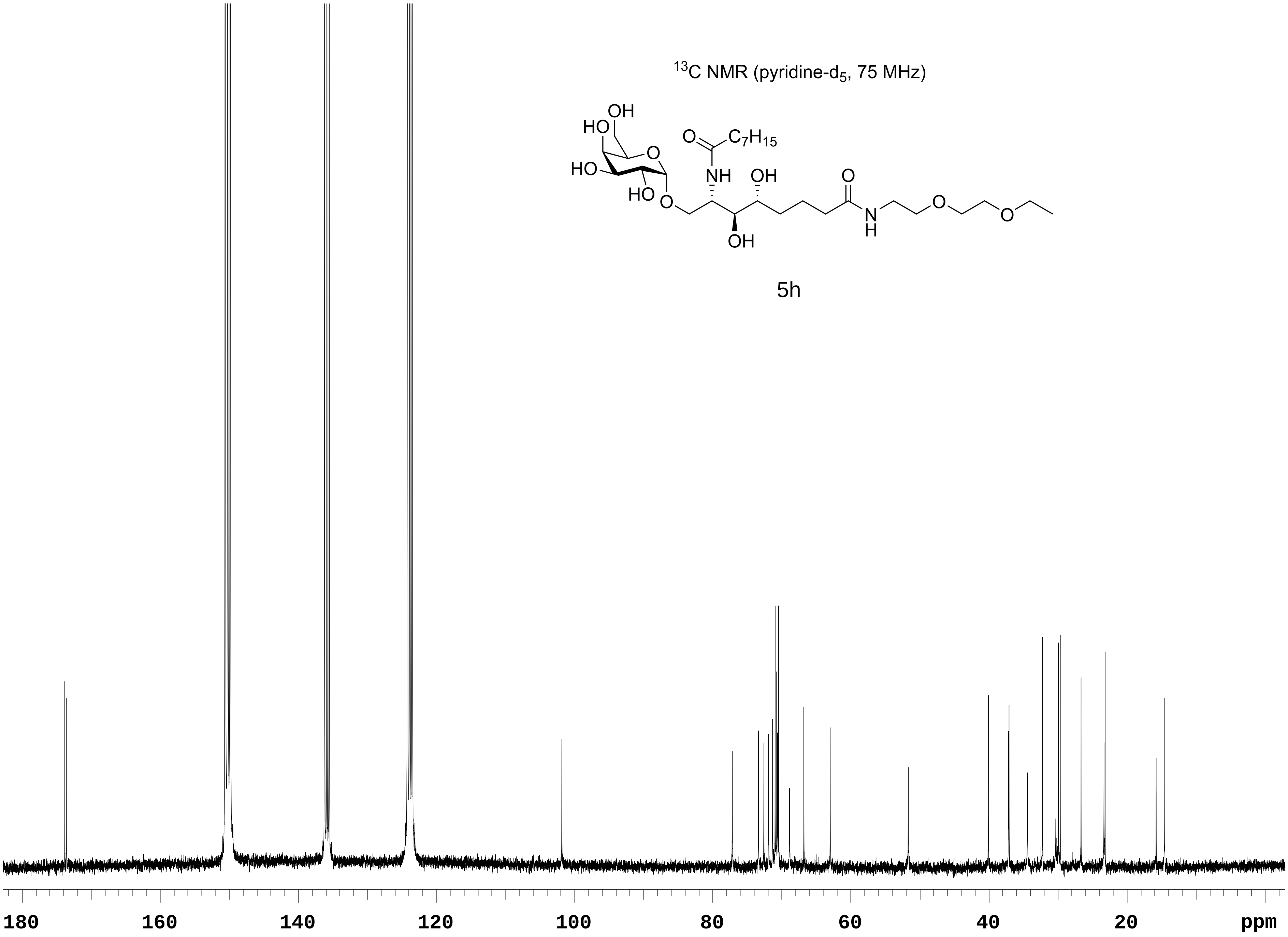

^{13}C NMR (pyridine-d$_5$, 75 MHz)
OH
HO
O
C7H15
HO
NH
OH
O
HO
O
N
H
O
O
OH
180
160
140
120
100
80
60
40
20
ppm

5h

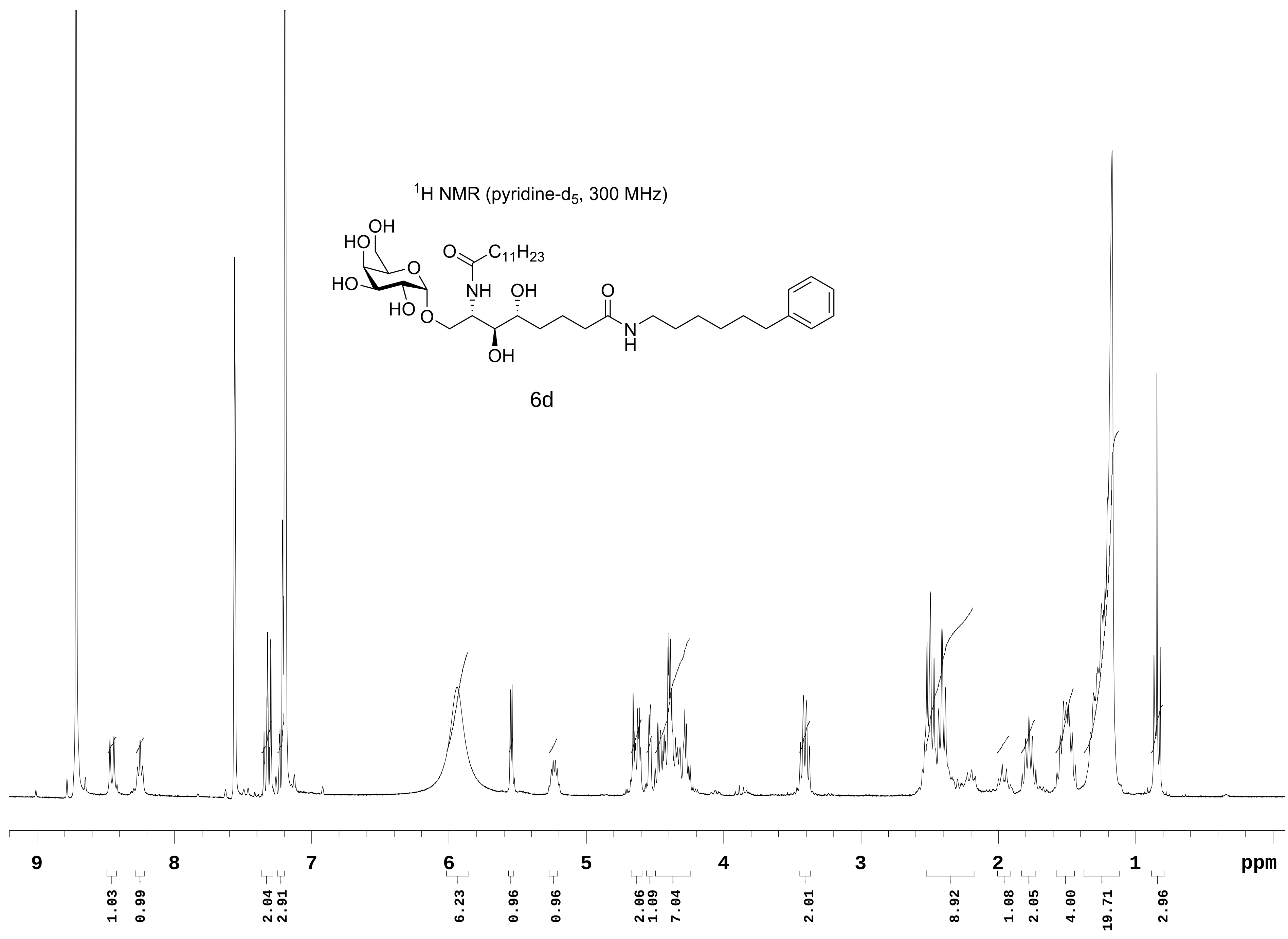

^{1}H NMR (pyridine-d$_5$, 300 MHz)
OH
HO
O
HO
HO
O
O
C$_{11}$H$_{23}$
NH
OH
OH
O
N
H
6d
9
8
7
6
5
4
3
2
1
ppm
1.03
0.99
2.04
2.91
6.23
0.96
0.96
2.06
1.09
7.04
2.01
8.92
1.08
2.05
4.00
19.71
2.96

^{13}C NMR (pyridine-d_5, 75 MHz)

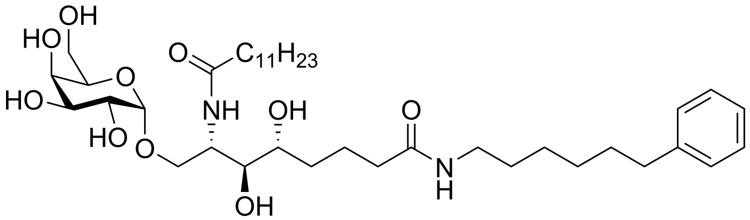

6d

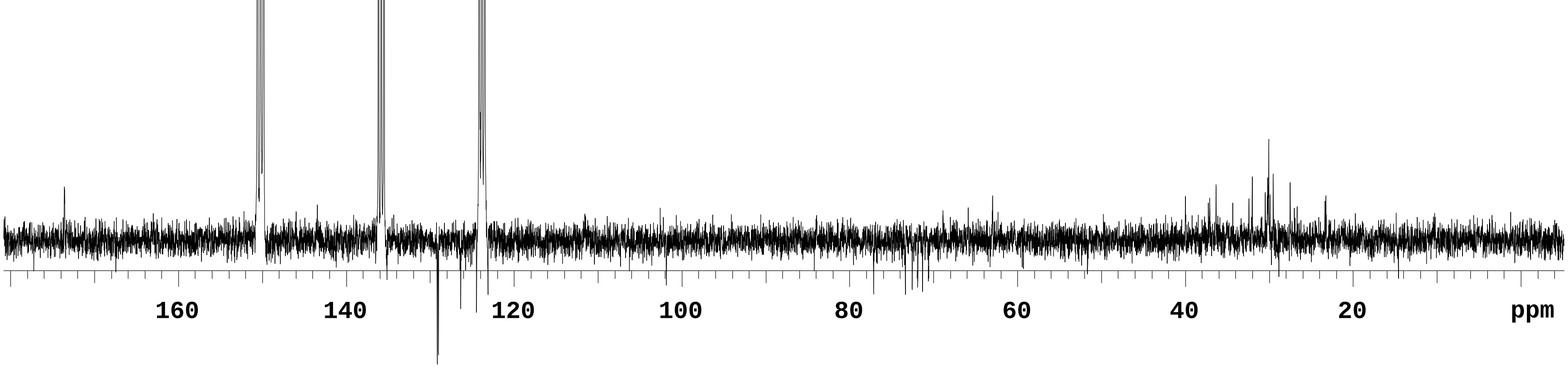

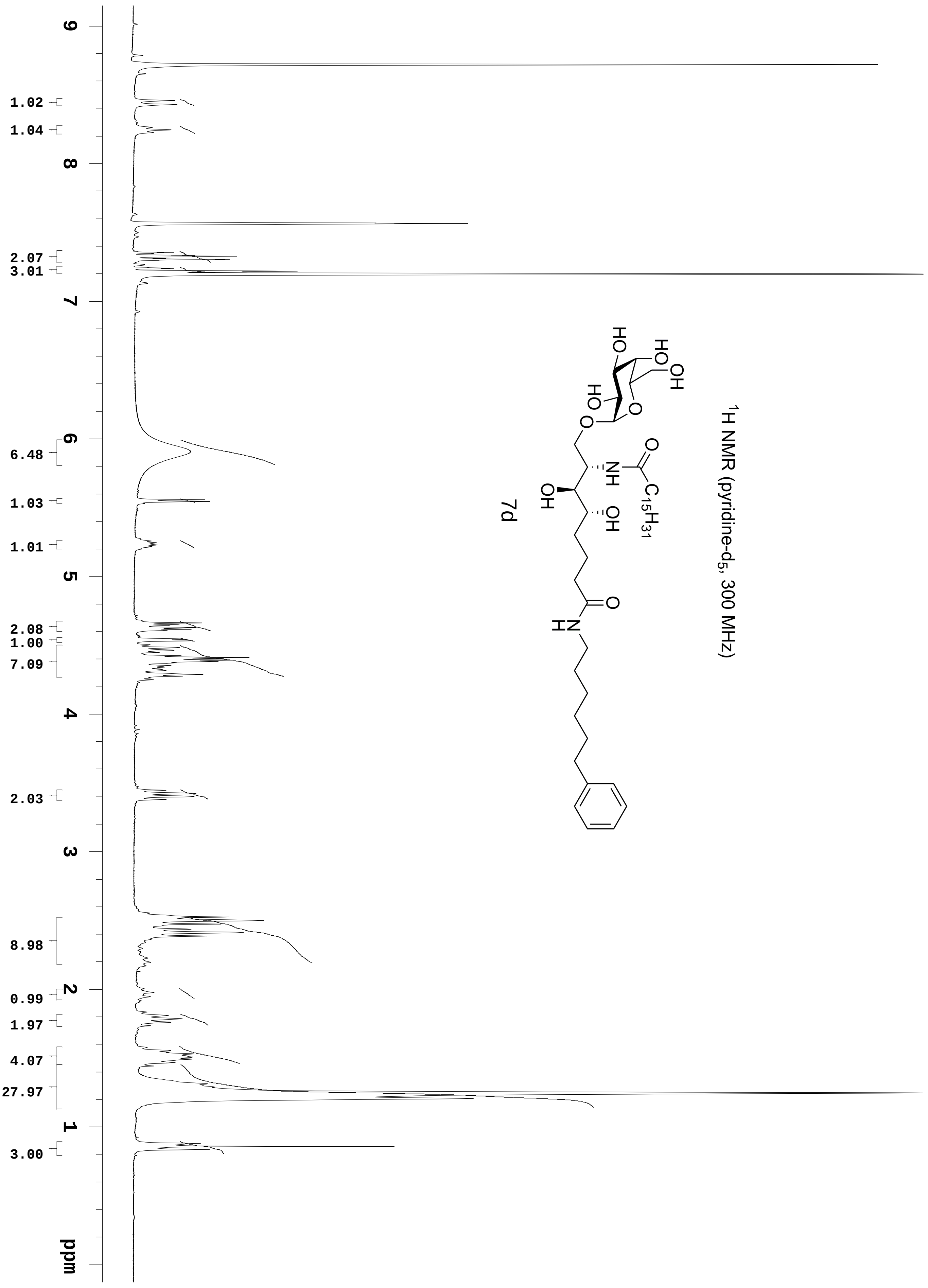

1H NMR (pyridine-d5, 300 MHz)
7d
ppm
9
8
7
6
5
4
3
2
1
1.02
1.04
2.07
3.01
6.48
1.03
1.01
2.08
1.00
7.09
2.03
8.98
0.99
1.97
4.07
27.97
3.00

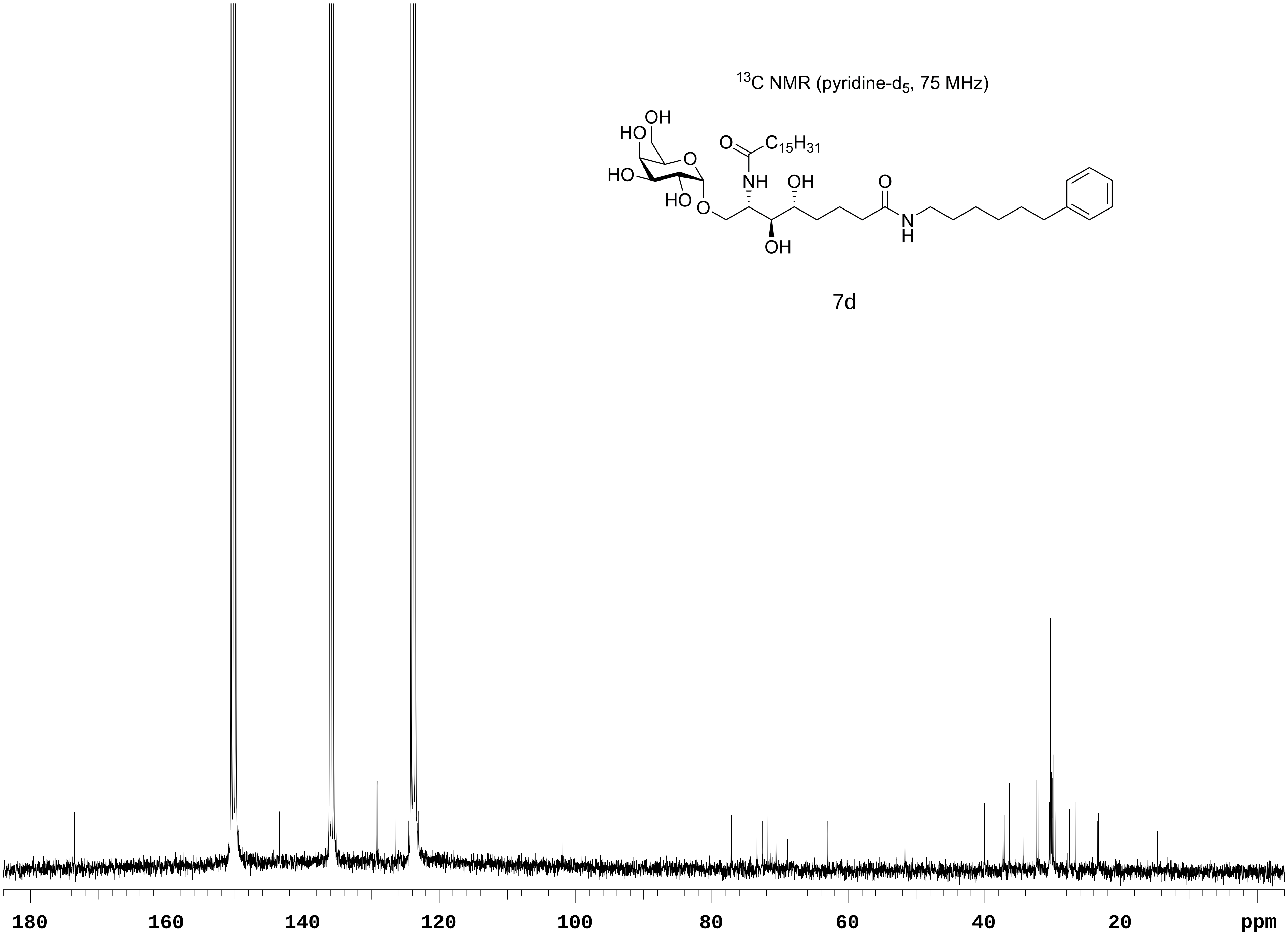

^{13}C NMR (pyridine-d5, 75 MHz)
HO
OH
O
HO
HO
O
NH
O
C15H31
OH
OH
O
N
H
7d
180
160
140
120
100
80
60
40
20
ppm

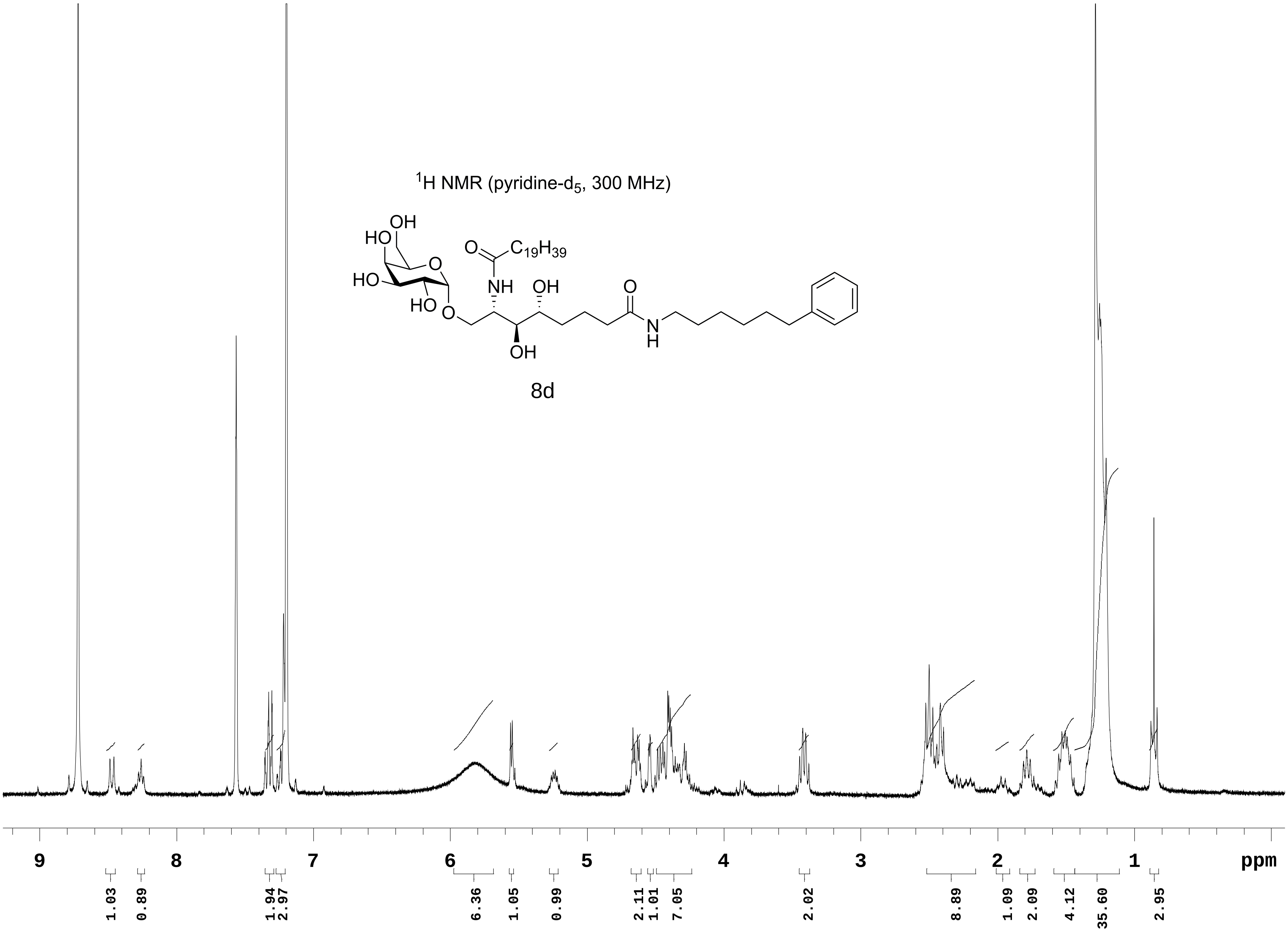

1H NMR (pyridine-d5, 300 MHz)
8d
9
8
7
6
5
4
3
2
1
ppm
1.03
0.89
1.94
2.97
6.36
1.05
0.99
2.11
1.01
7.05
2.02
8.89
1.09
2.09
4.12
35.60
2.95

^{13}C NMR (pyridine-d_5, 75 MHz)

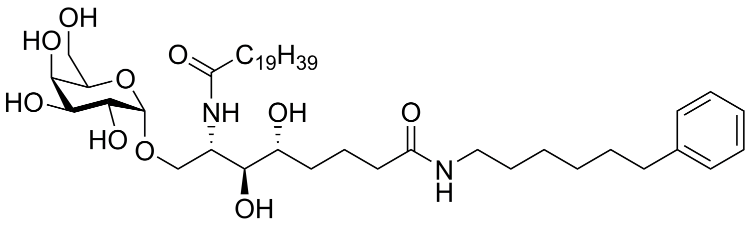

8d

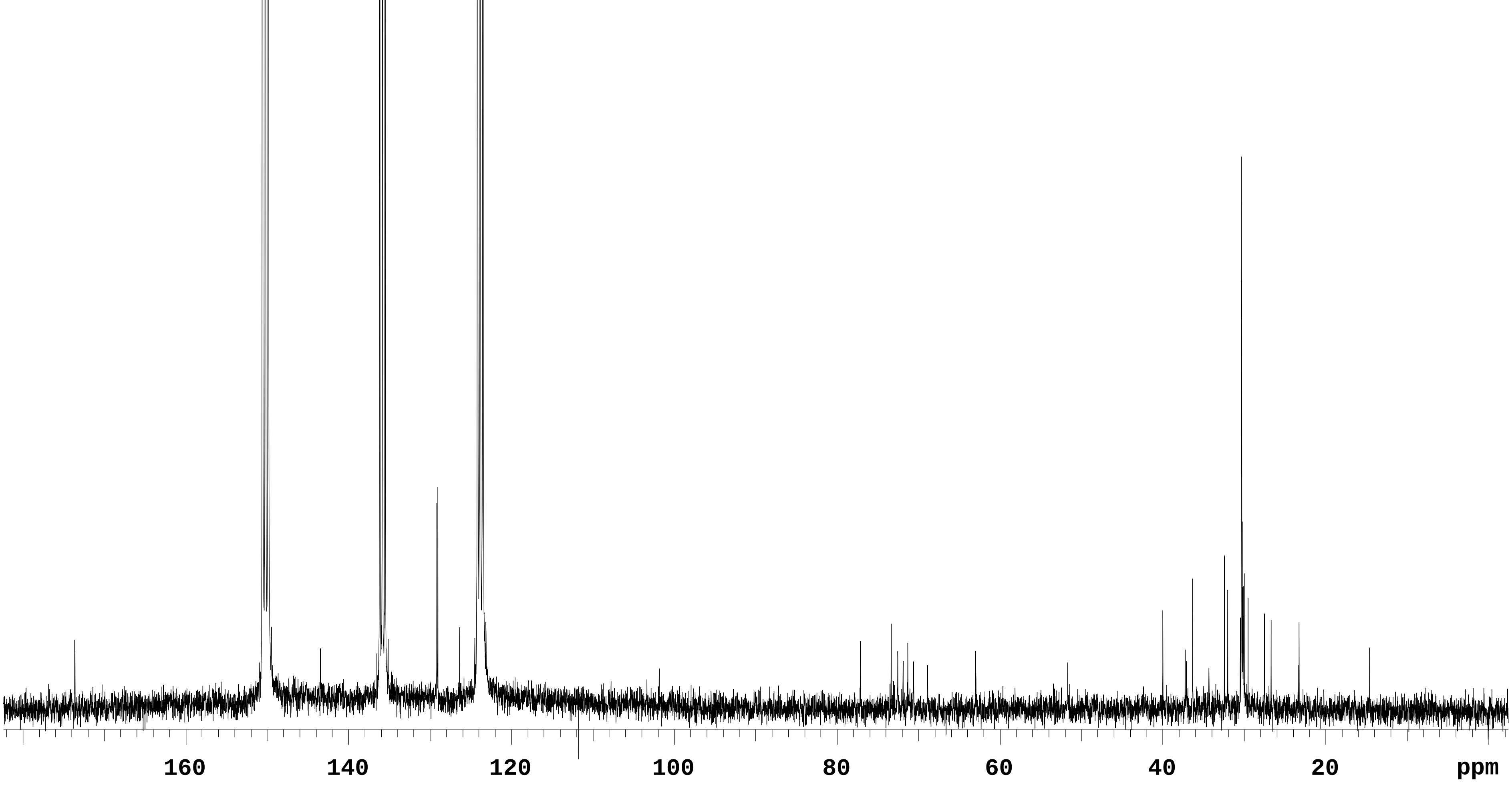

^{1}H NMR (pyridine-d_5, 300 MHz)

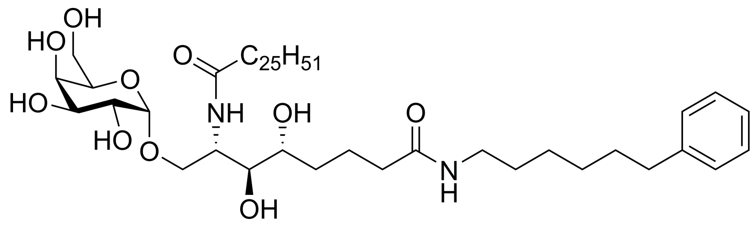

9d

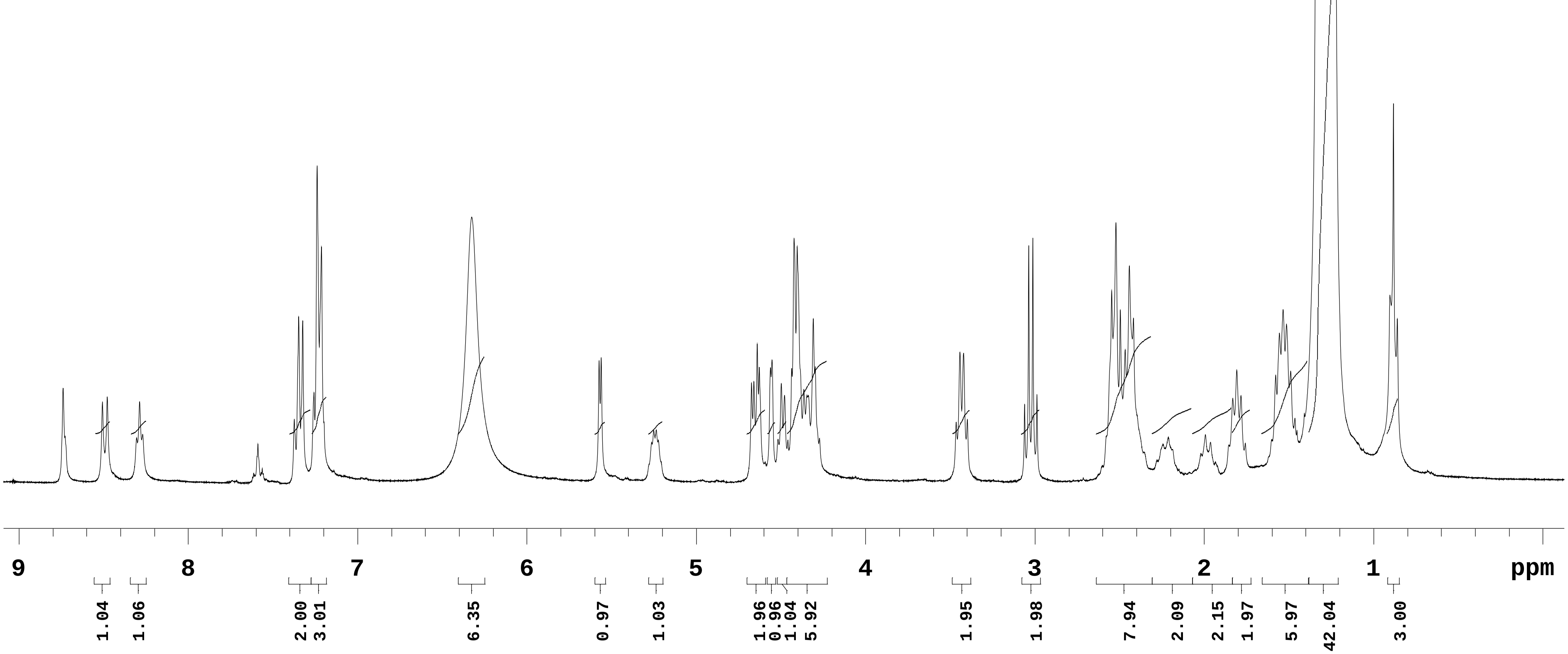

^{13}C NMR (pyridine-d_5, 75 MHz)

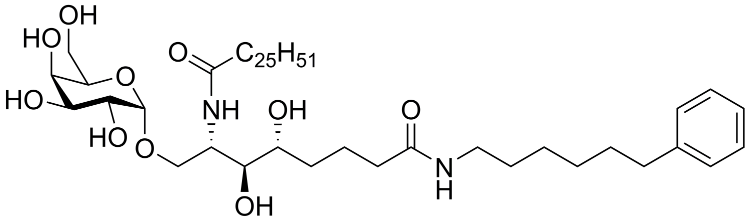

9d

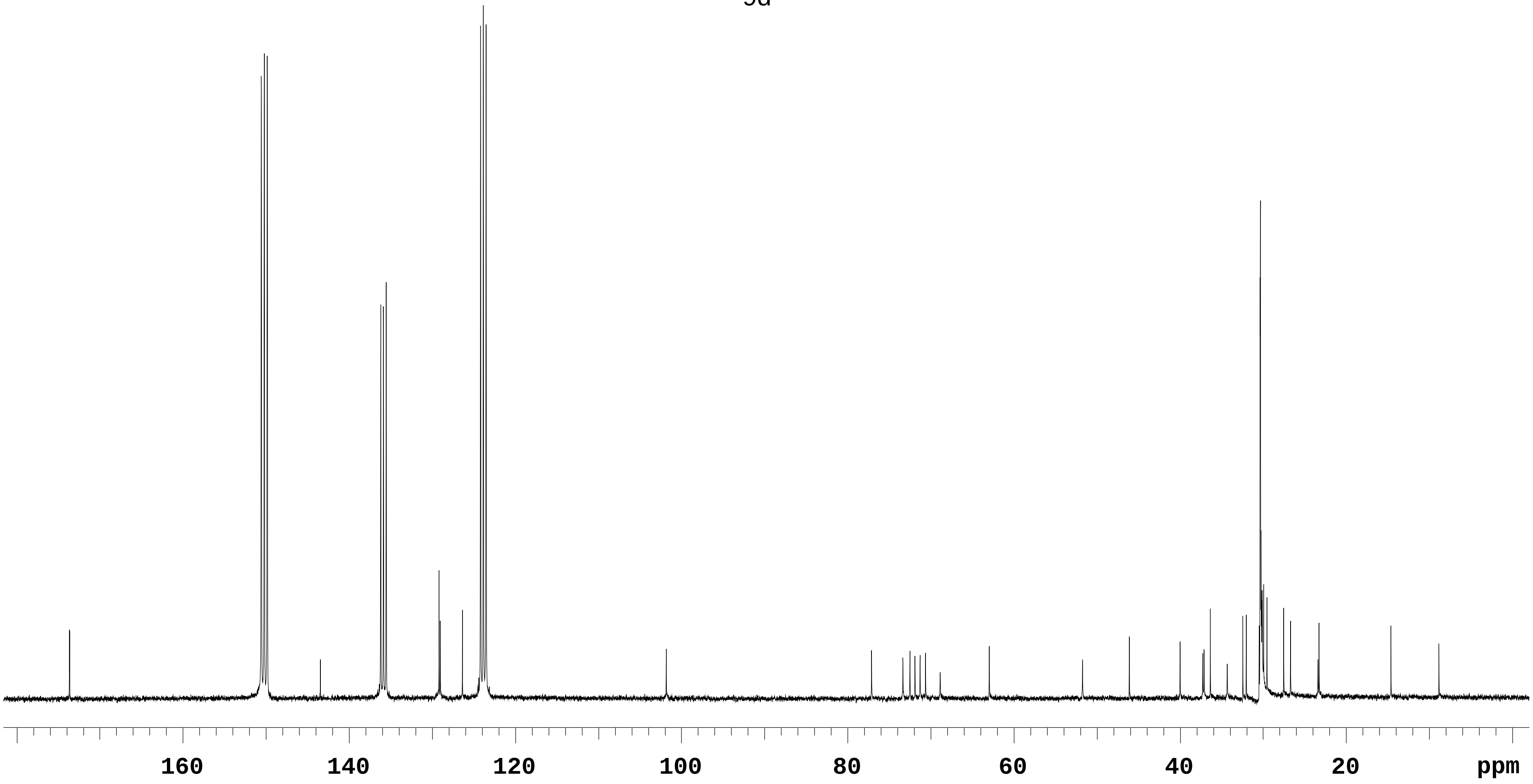

^{1}H NMR (pyridine-d_5, 300 MHz)

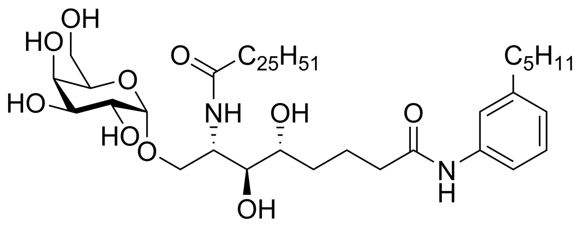

9f

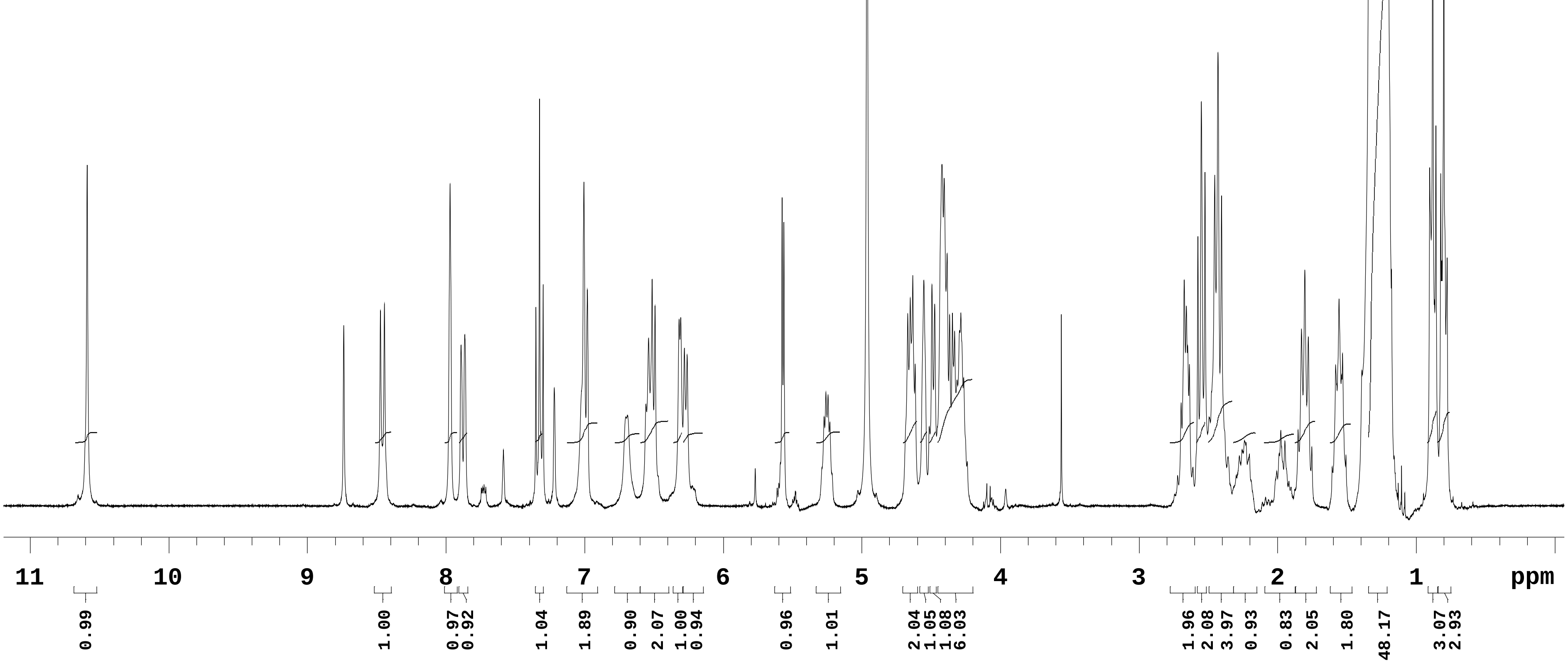

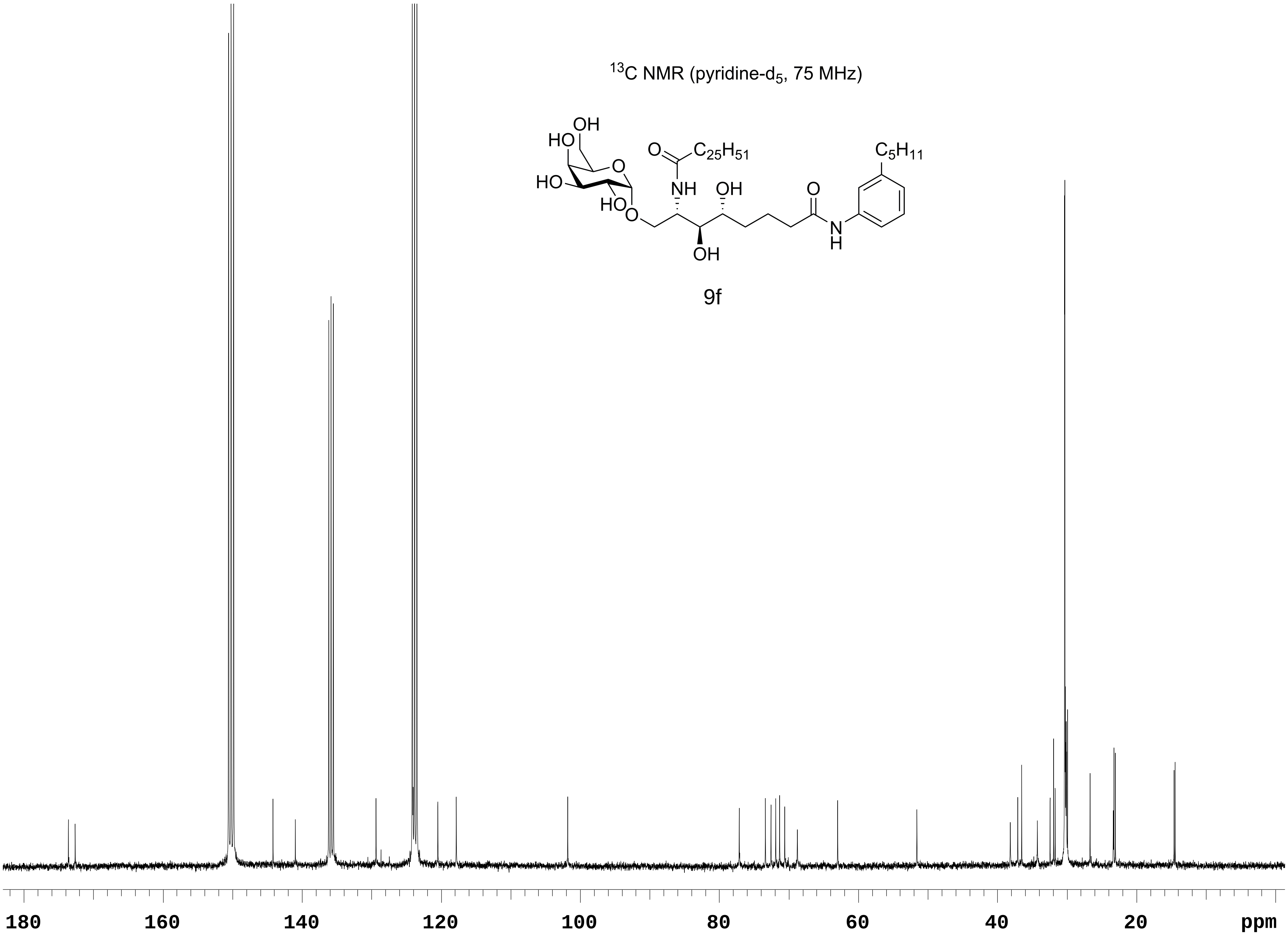

^{13}C NMR (pyridine-d$_5$, 75 MHz)
OH
HO
O
HO
HO
O
O
$C_{25}H_{51}$
NH
OH
OH
O
N
H
C_5H_{11}
9f
180
160
140
120
100
80
60
40
20
ppm

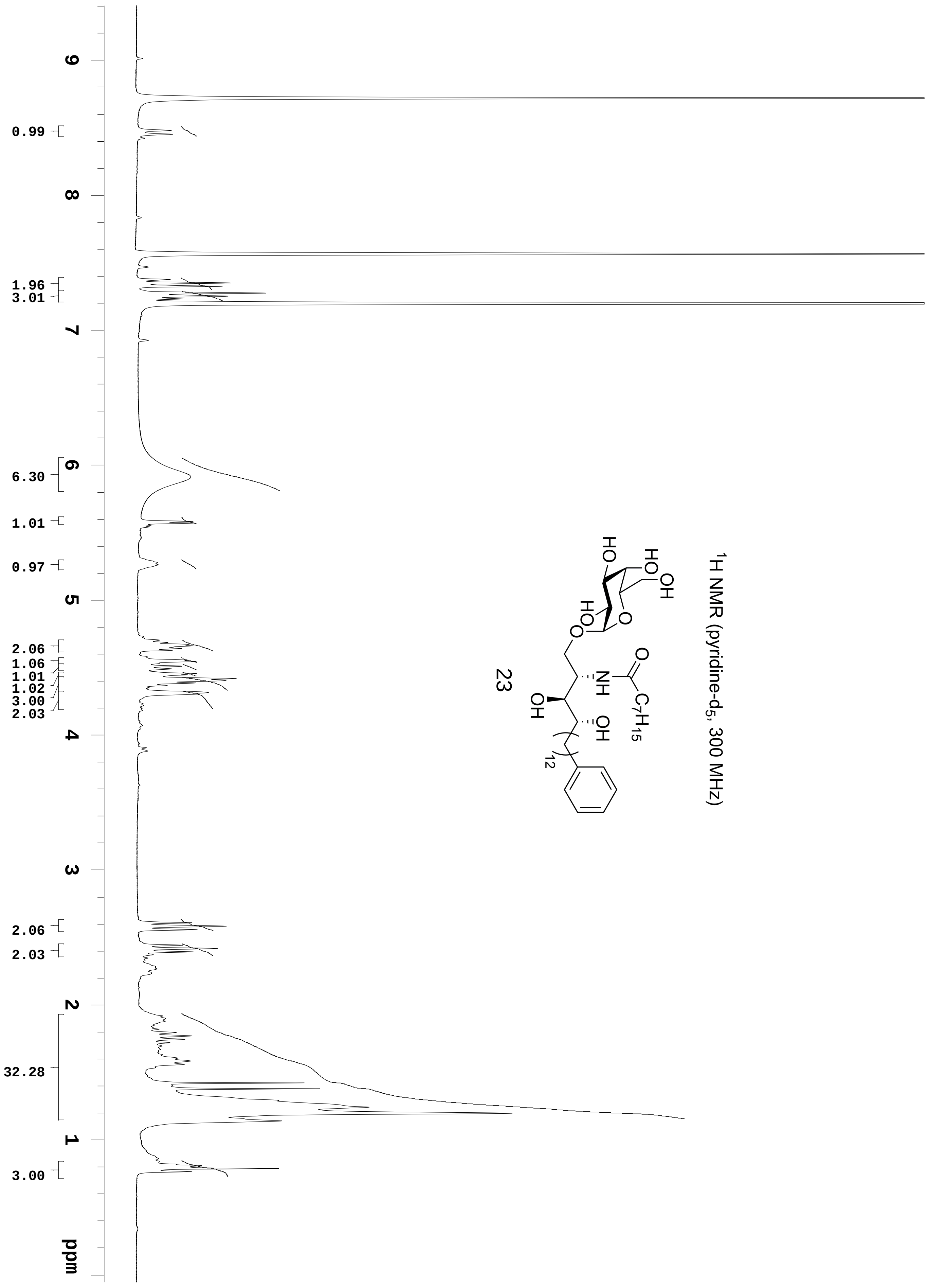

^{1}H NMR (pyridine-d$_5$, 300 MHz)
23
ppm

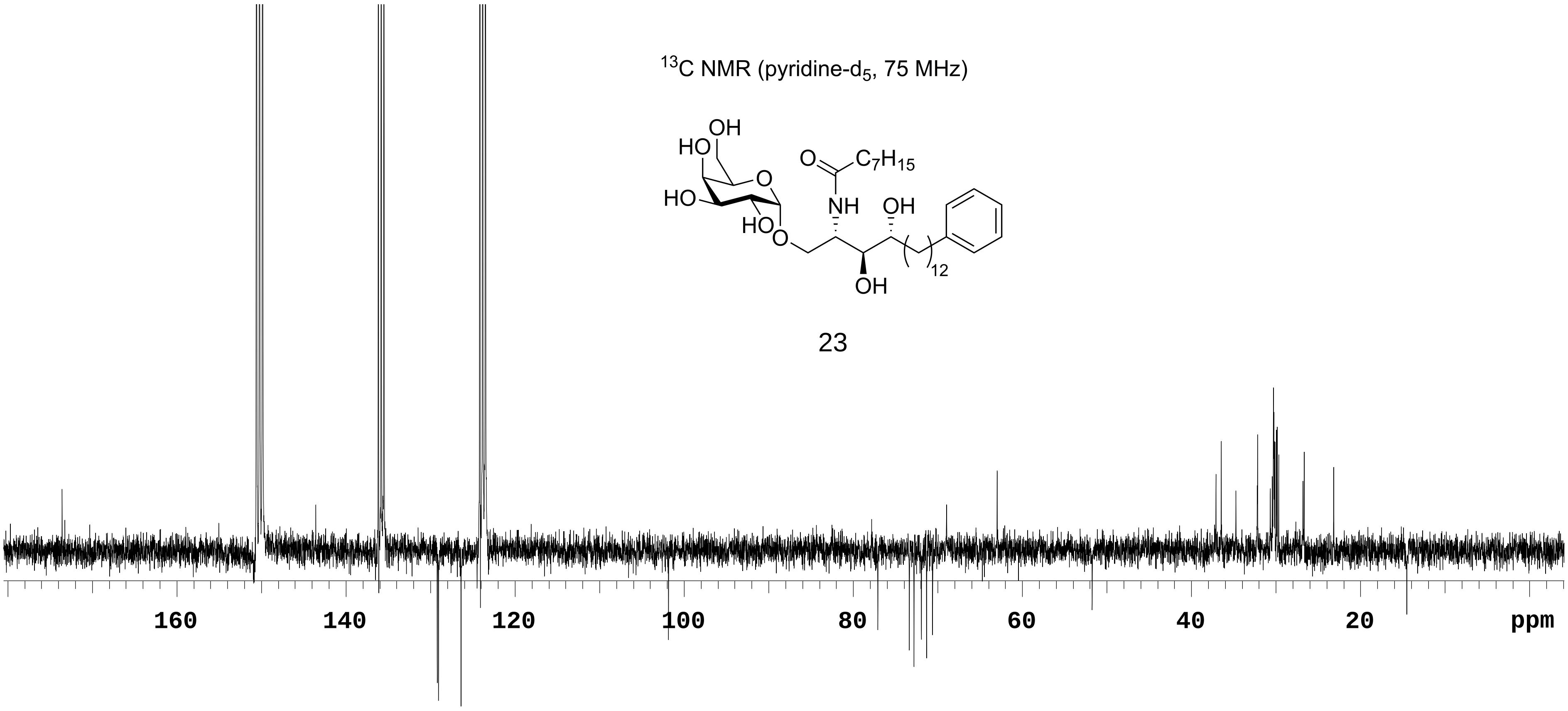

^{13}C NMR (pyridine-d5, 75 MHz)
OH
HO
O
HO
HO
O
O
C7H15
NH
OH
OH
12
23
160
140
120
100
80
60
40
20
ppm

Purity assessment

LC-MS analyses were carried out on a Waters Alliance 2695 XE separation Module by using a Phenomenex Kinetex EVO C18, 5um 100x2.1mm column and a gradient system of TFA in H_2O (0.05 %, v/v) / TFA in MeOH (0.05 %, v/v) at a flow rate of 0.6 mLmin-1, 95:05 to 00:100 (05 to 100 % MeOH) in 9 minutes. High-resolution spectra were recorded on a Waters LCT Premier XE Mass spectrometer.

1. **5a** (not UV active)

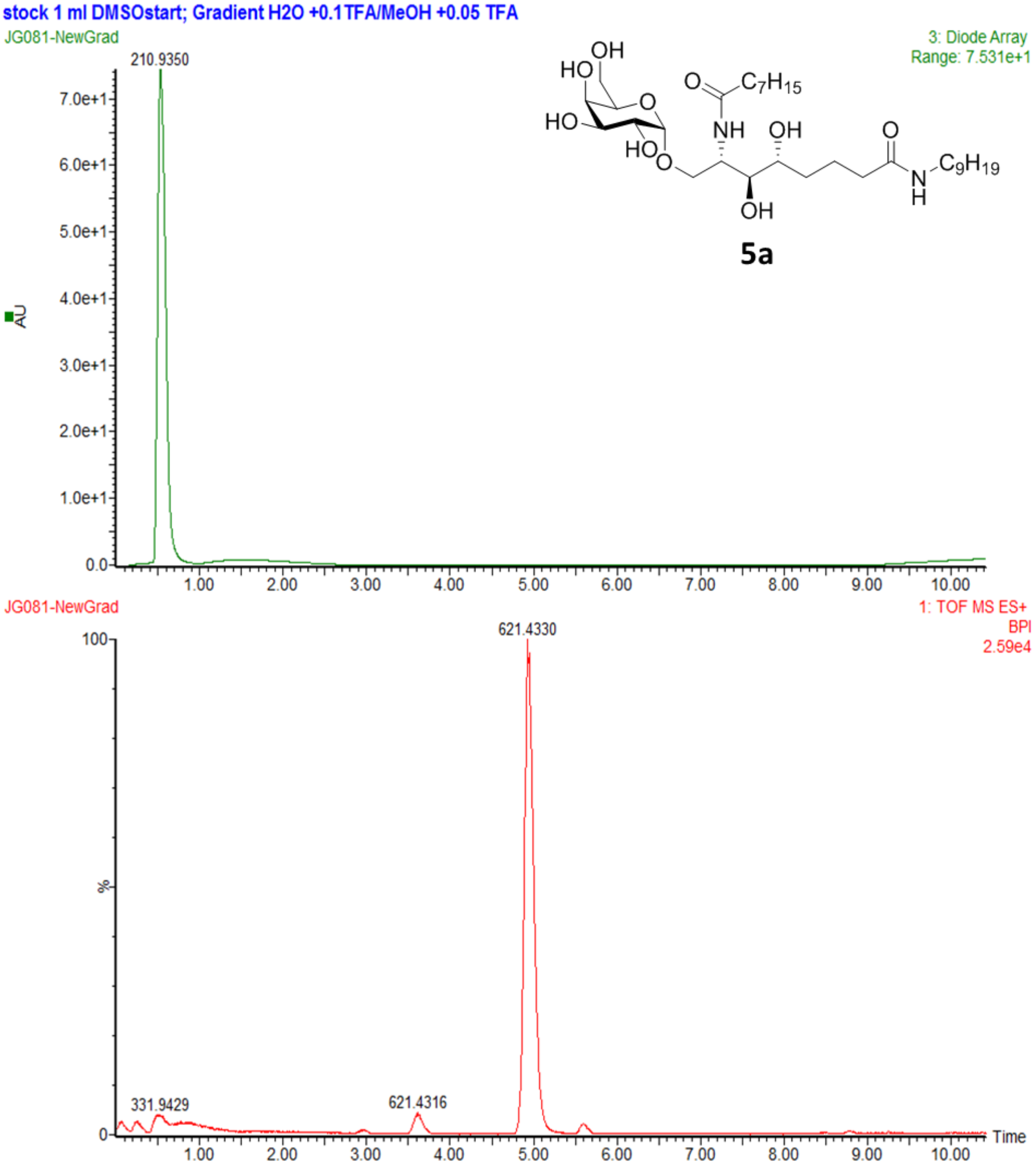

2. 5b

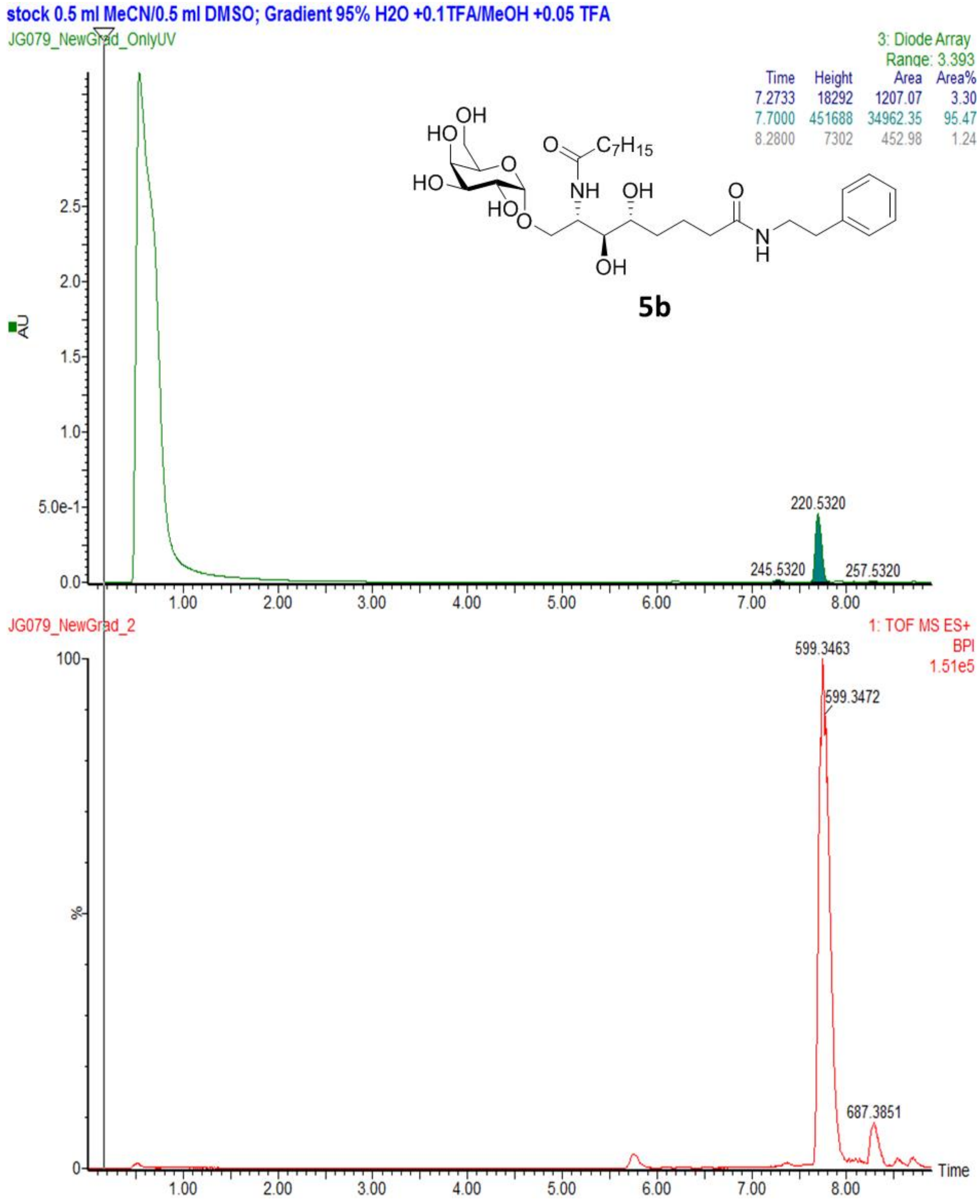

stock 0.5 ml MeCN/0.5 ml DMSO; Gradient 95% H2O +0.1TFA/MeOH +0.05 TFA
JG079_NewGrad_OnlyUV
3: Diode Array
Range: 3.393
Time Height Area Area%
7.2733 18292 1207.07 3.30
7.7000 451688 34962.35 95.47
8.2800 7302 452.98 1.24
5b
AU
220.5320
245.5320
257.5320
JG079_NewGrad_2
1: TOF MS ES+
BPI
1.51e5
599.3463
599.3472
687.3851
%
Time

3. 5c

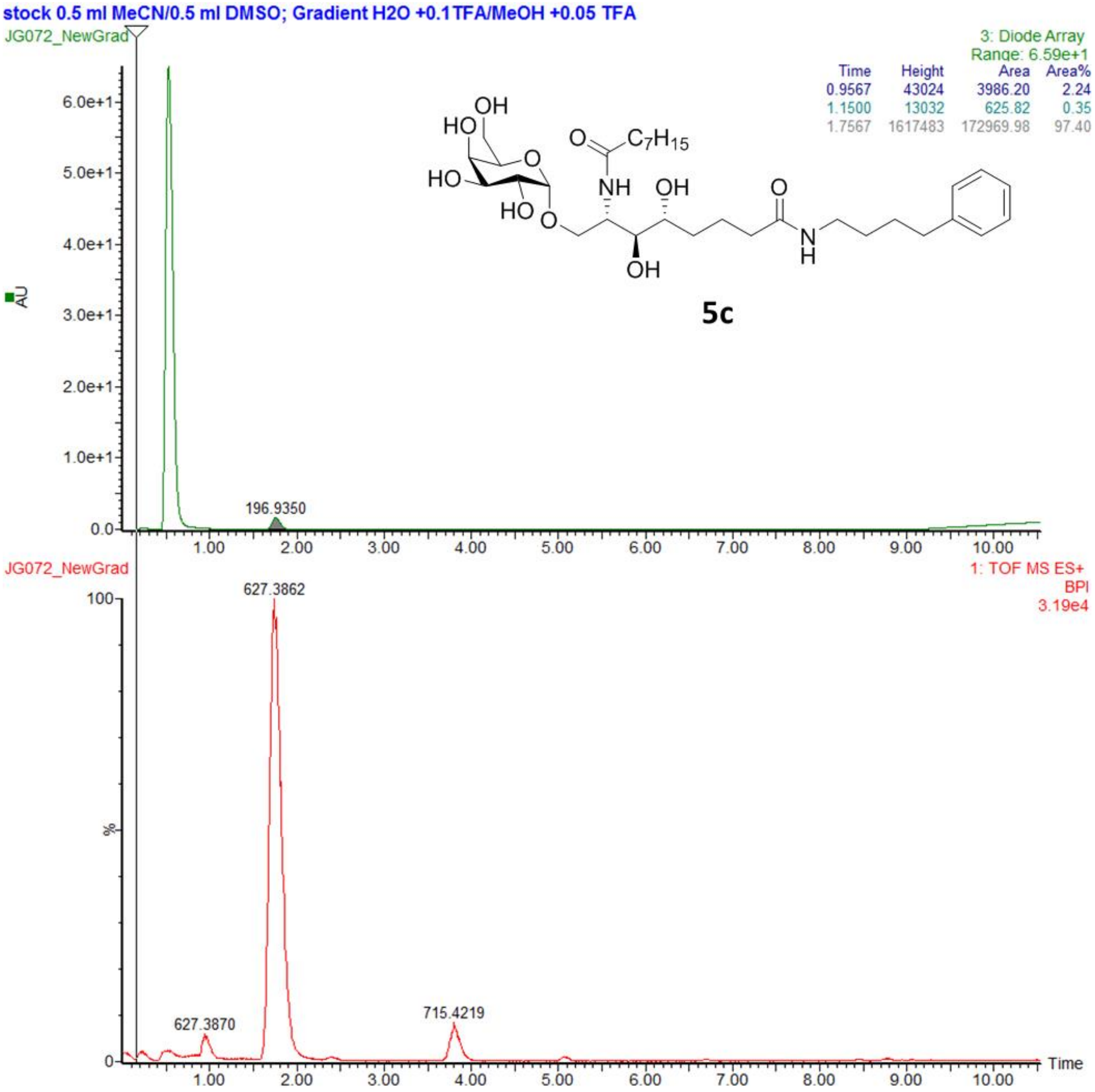
stock 0.5 ml MeCN/0.5 ml DMSO; Gradient H2O +0.1TFA/MeOH +0.05 TFA
JG072_NewGrad
3: Diode Array
Range: 6.59e+1
Time Height Area Area%
0.9567 43024 3986.20 2.24
1.1500 13032 625.82 0.35
1.7567 1617483 172969.98 97.40
AU
196.9350
5c
JG072_NewGrad
1: TOF MS ES+
BPI
3.19e4
627.3862
627.3870
715.4219
%
Time

4. 5d

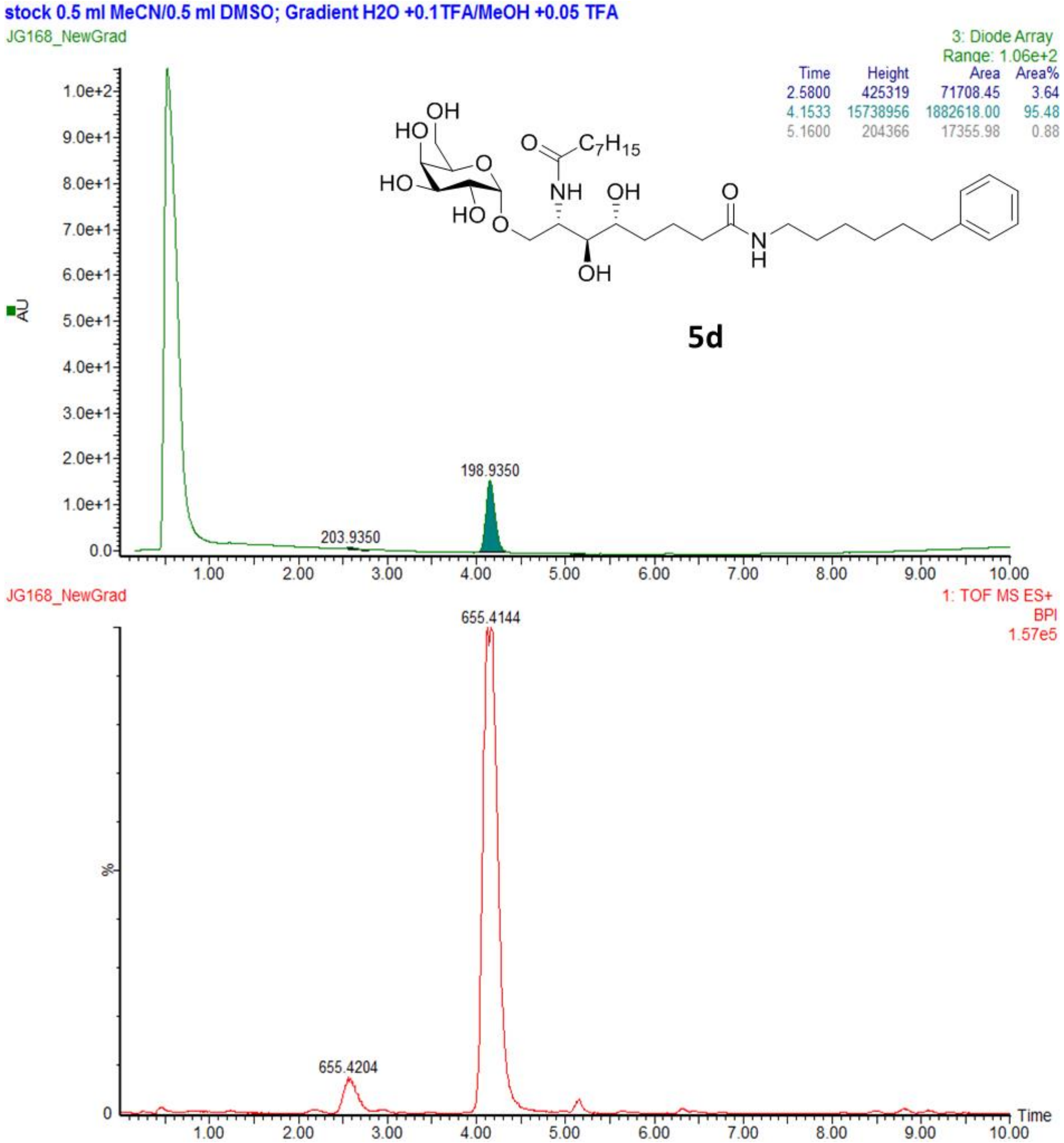

stock 0.5 ml MeCN/0.5 ml DMSO; Gradient H2O +0.1TFA/MeOH +0.05 TFA
JG168_NewGrad
3: Diode Array
Range: 1.06e+2
Time Height Area Area%
2.5800 425319 71708.45 3.64
4.1533 15738956 1882618.00 95.48
5.1600 204366 17355.98 0.88
AU
198.9350
203.9350
5d
JG168_NewGrad
1: TOF MS ES+
BPI
1.57e5
655.4144
655.4204
%
Time

stock 0.5 ml MeCN/0.5 ml DMSO; Gradient H2O +0.1TFA/MeOH +0.05 TFA

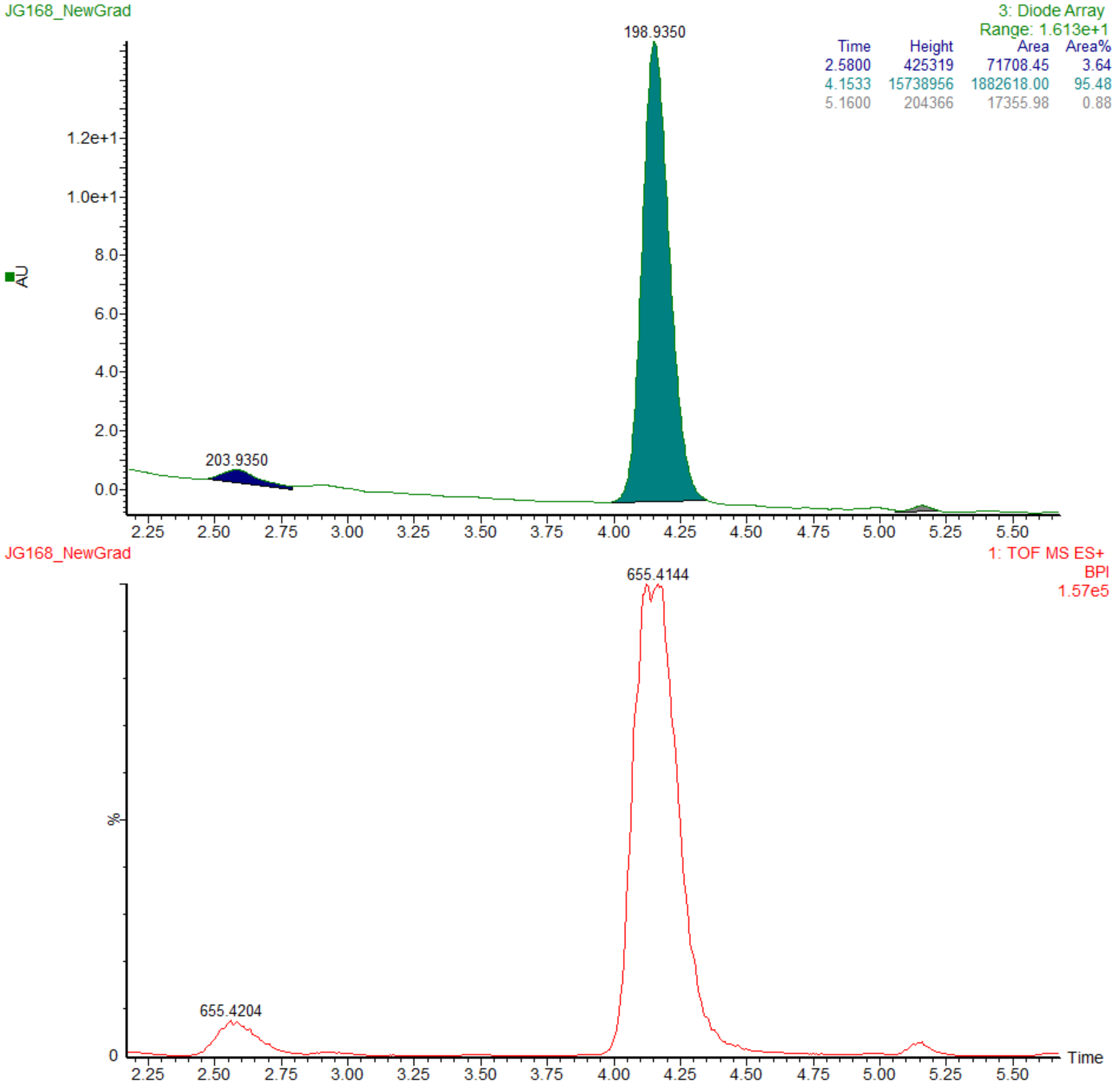

5. 5e

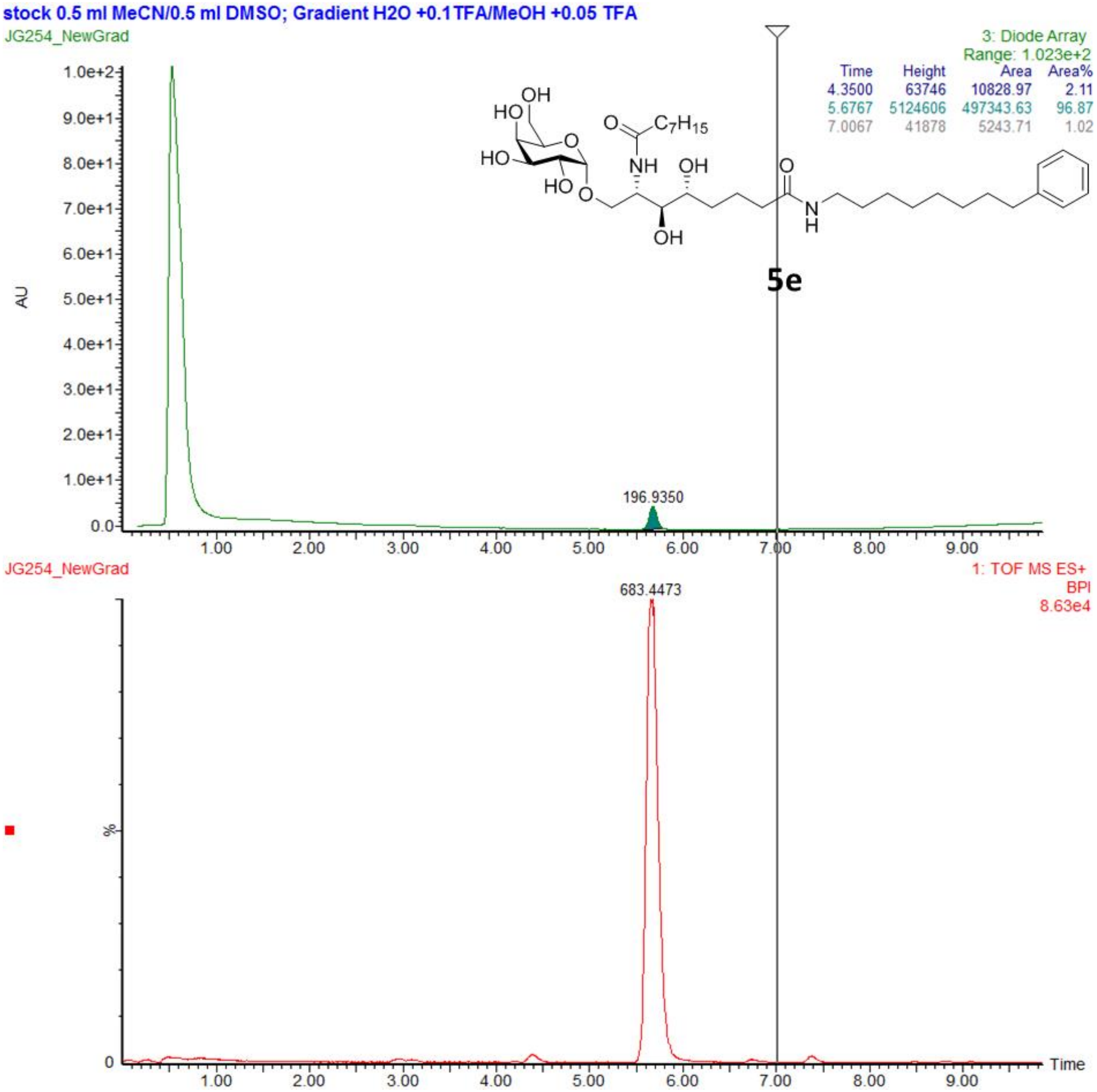

stock 0.5 ml MeCN/0.5 ml DMSO; Gradient H2O +0.1TFA/MeOH +0.05 TFA
JG254_NewGrad
3: Diode Array
Range: 1.023e+2
Time Height Area Area%
4.3500 63746 10828.97 2.11
5.6767 5124606 497343.63 96.87
7.0067 41878 5243.71 1.02
5e
AU
196.9350
JG254_NewGrad
1: TOF MS ES+
BPI
8.63e4
683.4473
%
Time

stock 0.5 ml MeCN/0.5 ml DMSO; Gradient H2O +0.1TFA/MeOH +0.05 TFA

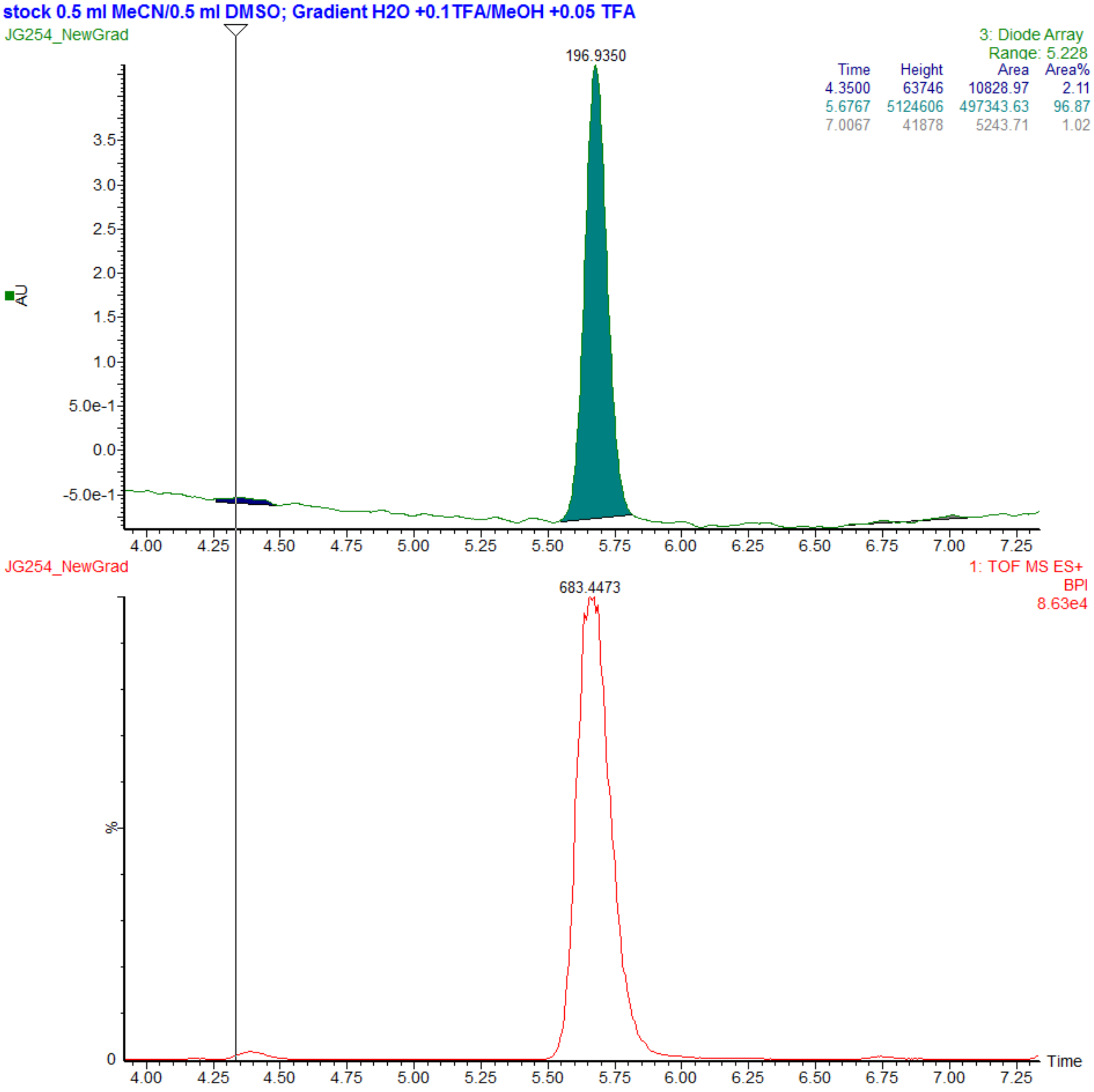

6. **5f**

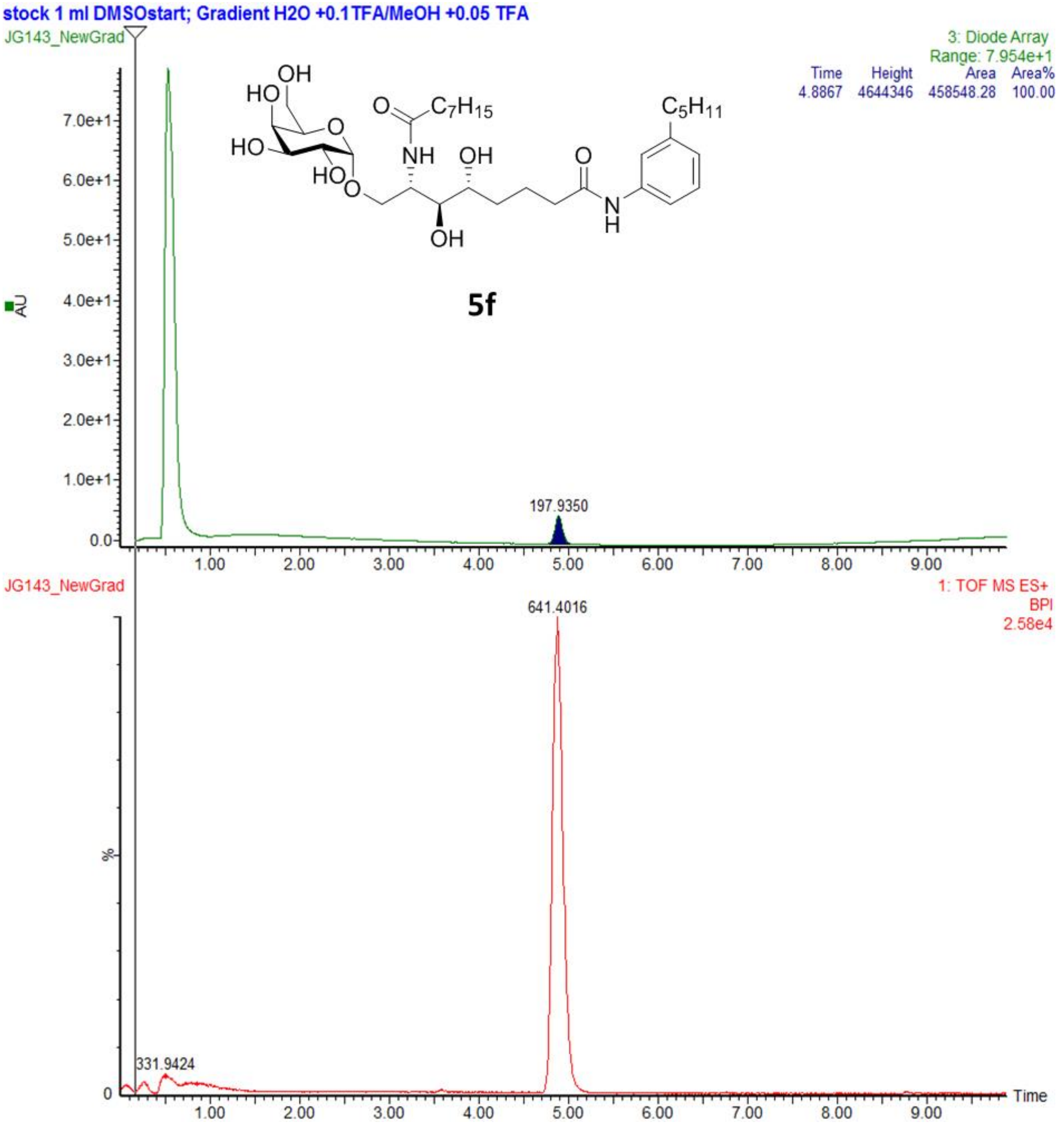

stock 1 ml DMSOstart; Gradient H2O +0.1TFA/MeOH +0.05 TFA
JG143_NewGrad
3: Diode Array
Range: 7.954e+1
Time Height Area Area%
4.8867 4644346 458548.28 100.00
AU
7.0e+1
6.0e+1
5.0e+1
4.0e+1
3.0e+1
2.0e+1
1.0e+1
0.0
197.9350
1.00
2.00
3.00
4.00
5.00
6.00
7.00
8.00
9.00
5f
JG143_NewGrad
1: TOF MS ES+
BPI
2.58e4
641.4016
%
331.9424
0
Time

stock 1 ml DMSOstart; Gradient H2O +0.1TFA/MeOH +0.05 TFA

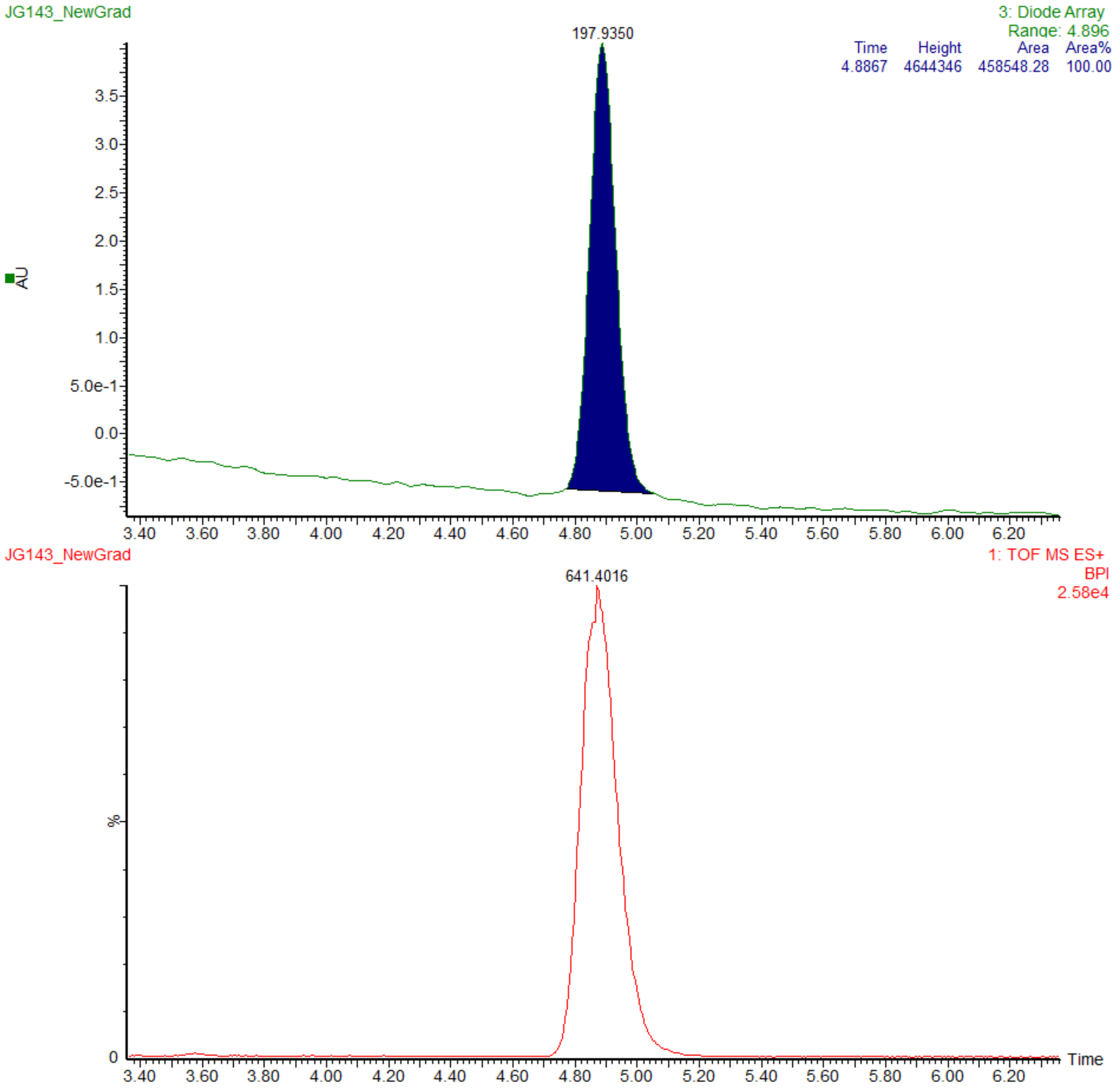

7. 5g

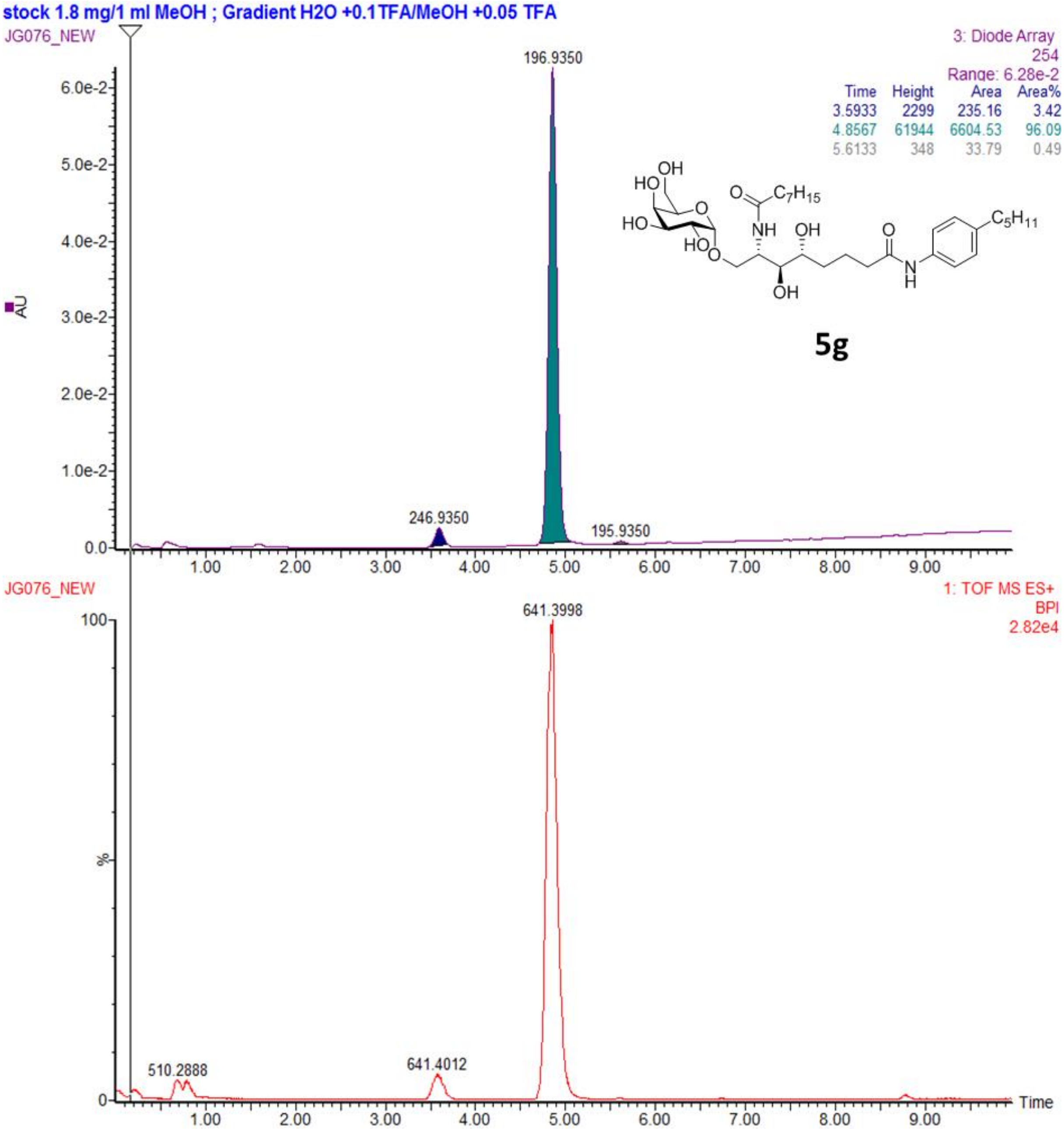

stock 1.8 mg/1 ml MeOH ; Gradient H2O +0.1TFA/MeOH +0.05 TFA
JG076_NEW
3: Diode Array
254
Range: 6.28e-2
Time Height Area Area%
3.5933 2299 235.16 3.42
4.8567 61944 6604.53 96.09
5.6133 348 33.79 0.49
196.9350
246.9350
195.9350
AU
6.0e-2
5.0e-2
4.0e-2
3.0e-2
2.0e-2
1.0e-2
0.0
1.00 2.00 3.00 4.00 5.00 6.00 7.00 8.00 9.00
OH
HO
O
HO
HO
O
O
C_7H_{15}
NH
OH
O
N
H
C_5H_{11}
OH
5g
JG076_NEW
1: TOF MS ES+
BPI
2.82e4
641.3998
641.4012
510.2888
100
%
0
Time

8. 5h

Purity assesment of **5h** was not possible due to decomposition of this galactosylsphingamide under the chromatographic conditions.

9. **6d**

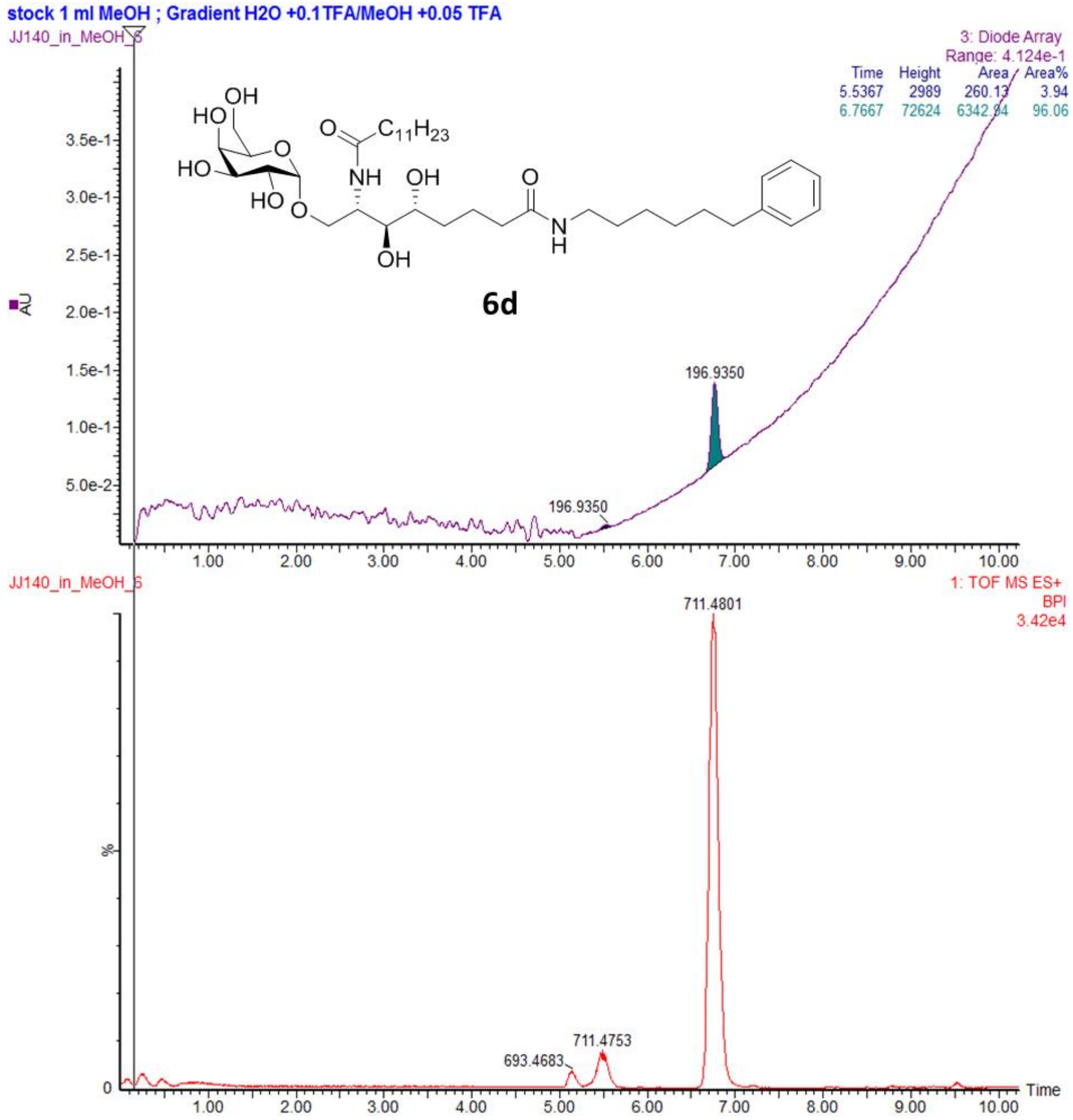

stock 1 ml MeOH ; Gradient H2O +0.1TFA/MeOH +0.05 TFA
JJ140_in_MeOH_6
3: Diode Array
Range: 4.124e-1
Time Height Area Area%
5.5367 2989 260.13 3.94
6.7667 72624 6342.94 96.06
6d
196.9350
196.9350
AU
JJ140_in_MeOH_6
1: TOF MS ES+
BPI
3.42e4
711.4801
711.4753
693.4683
%
Time

OnlyUV (higher concentration)

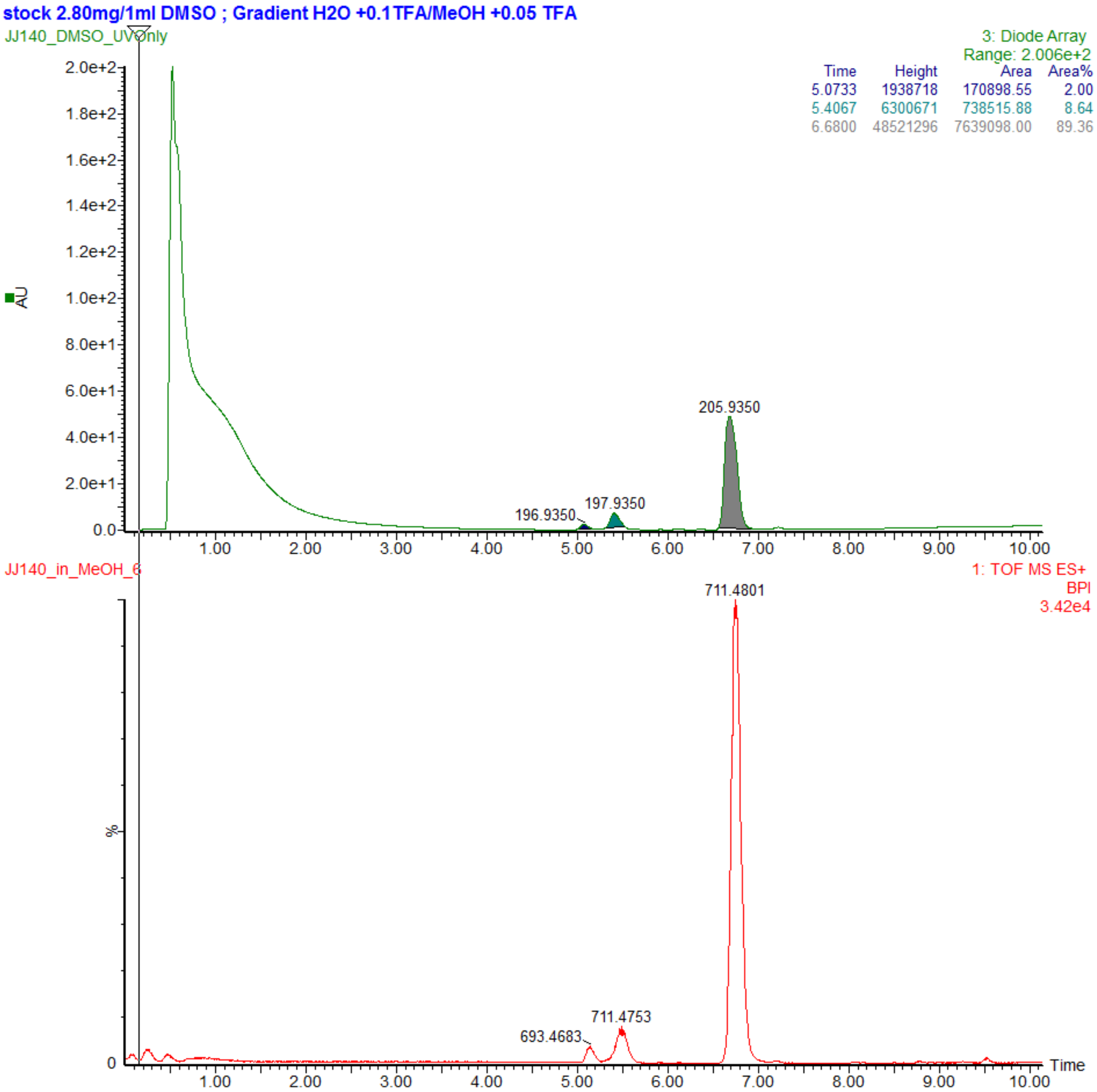

10. 7d

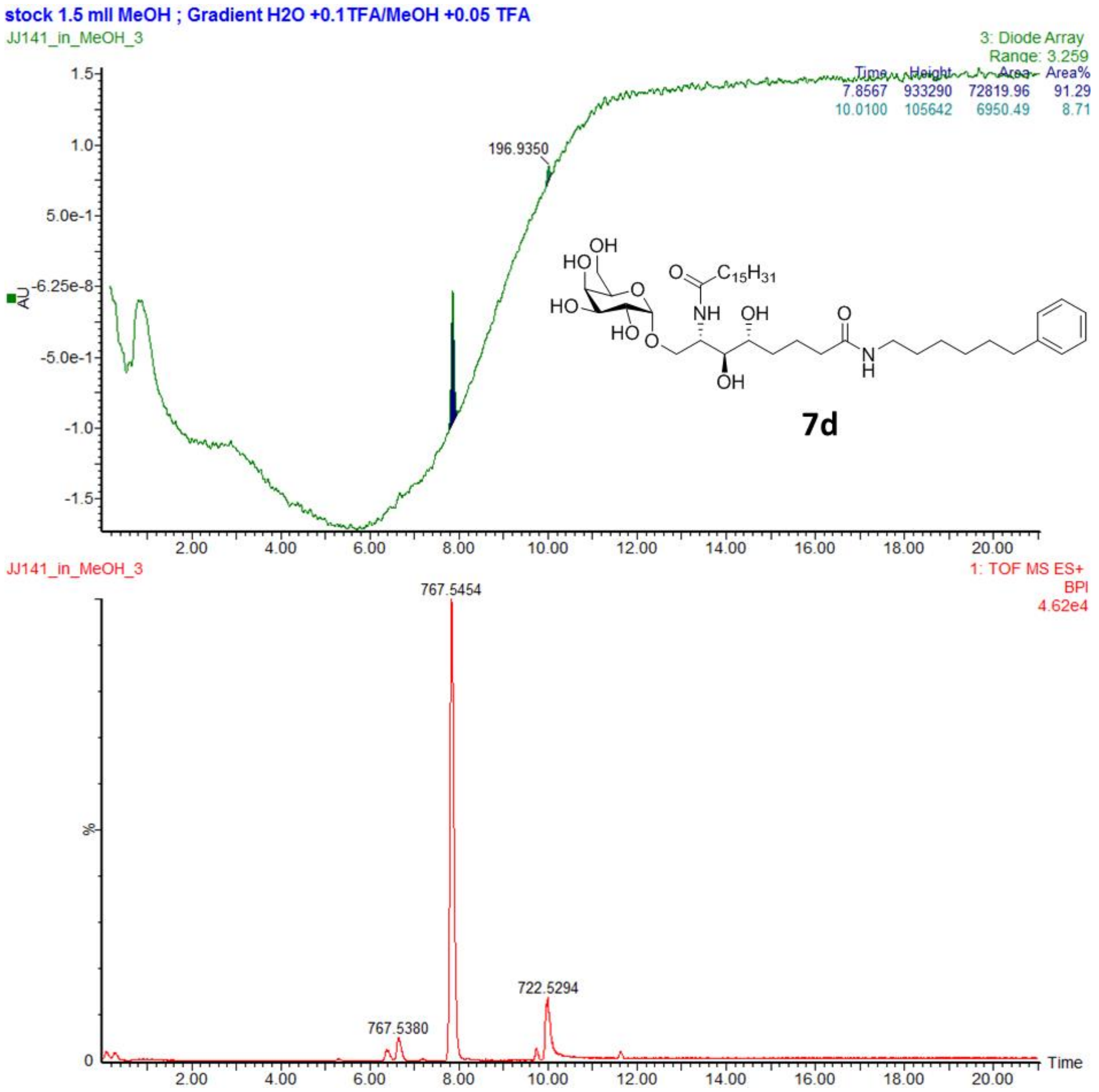

stock 1.5 mll MeOH ; Gradient H2O +0.1TFA/MeOH +0.05 TFA
JJ141_in_MeOH_3
3: Diode Array
Range: 3.259
Time Height Area Area%
7.8567 933290 72819.96 91.29
10.0100 105642 6950.49 8.71
196.9350
AU
1.5
1.0
5.0e-1
-6.25e-8
-5.0e-1
-1.0
-1.5
2.00
4.00
6.00
8.00
10.00
12.00
14.00
16.00
18.00
20.00
HO
OH
HO
O
HO
O
O
$C_{15}H_{31}$
NH
OH
OH
O
N
H
7d
JJ141_in_MeOH_3
1: TOF MS ES+
BPI
4.62e4
767.5454
722.5294
767.5380
%
0
Time

11. 8d (Peak 711.476 also in blanc)

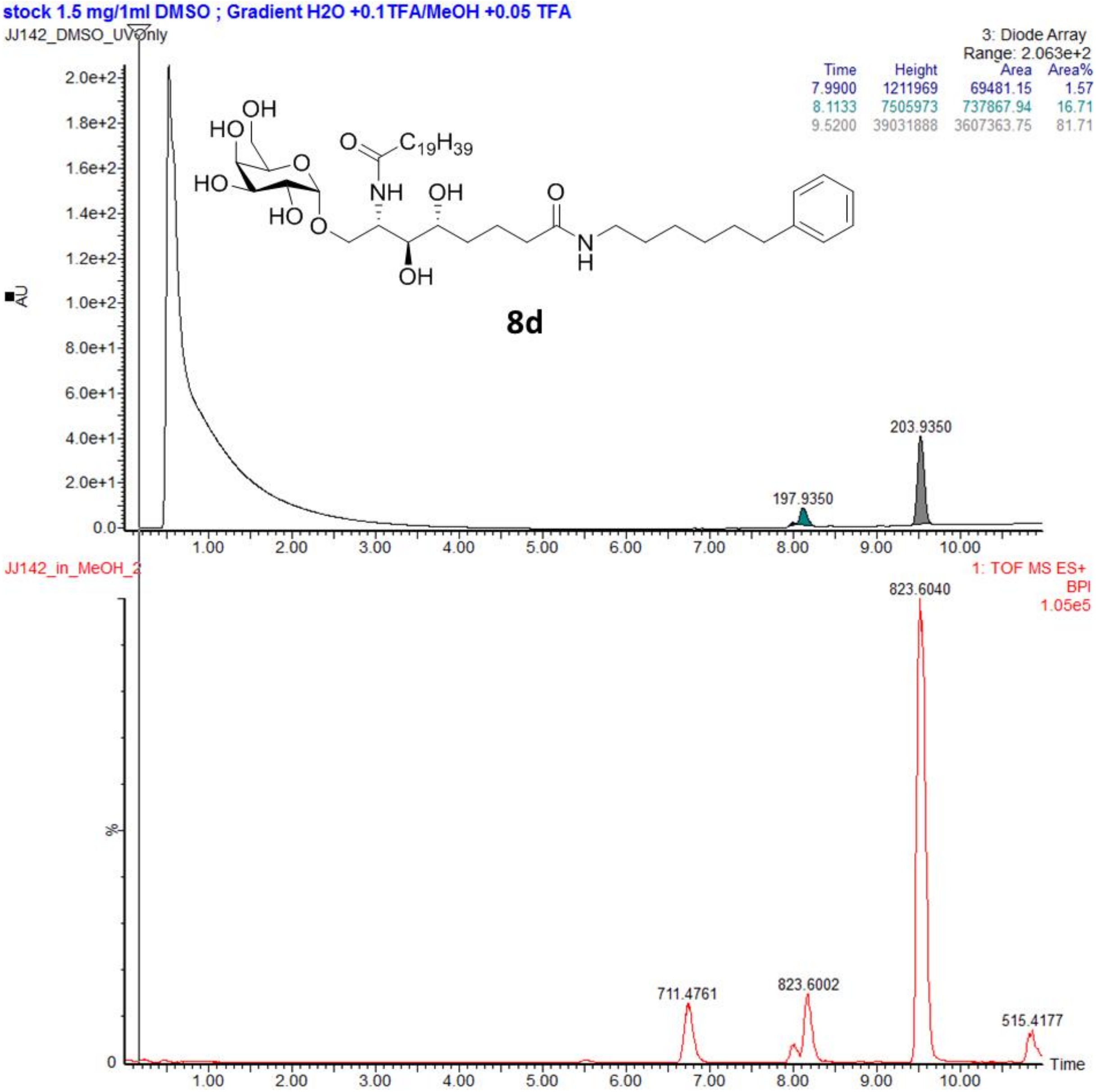

12. 9d

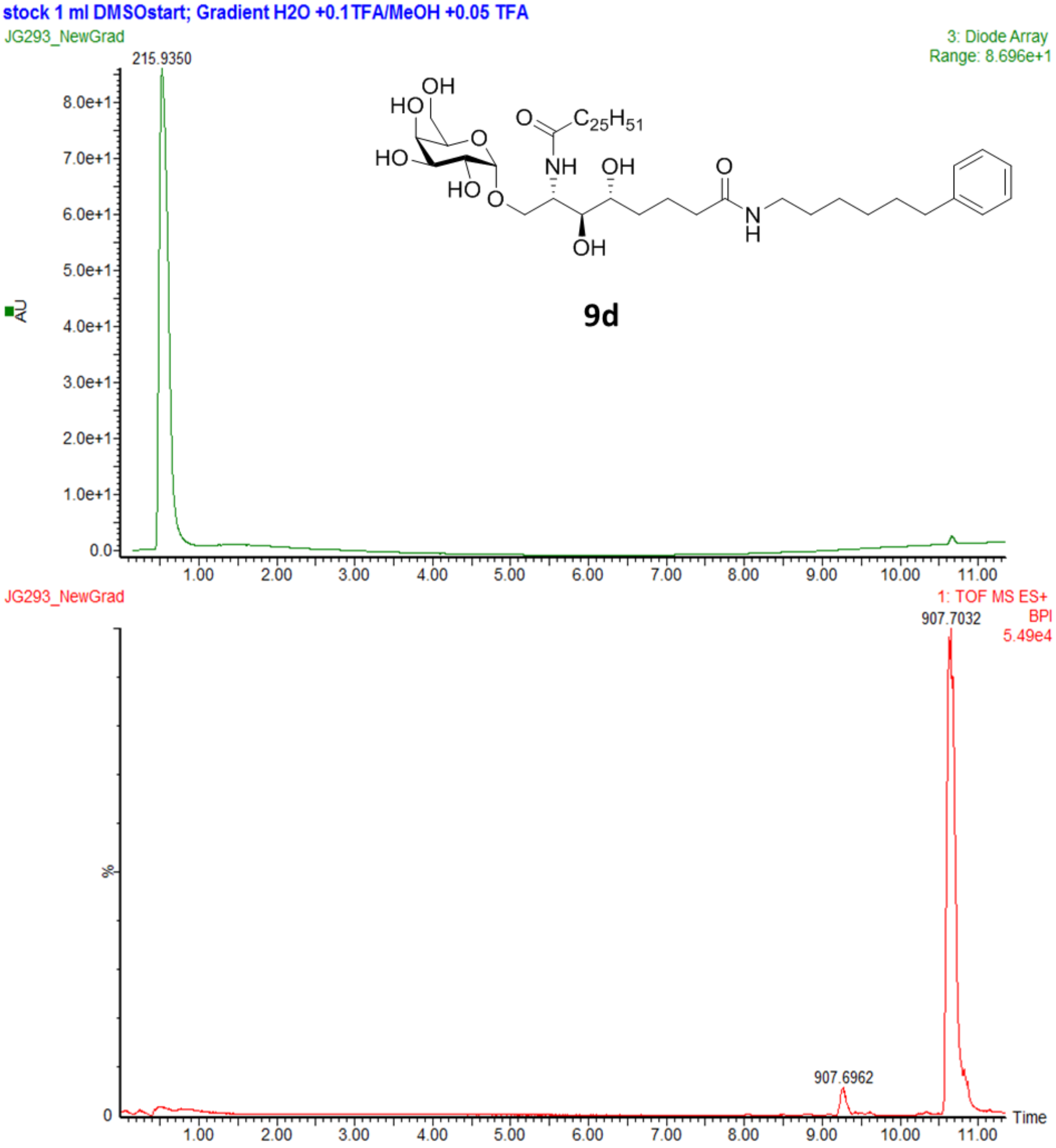

stock 1 ml DMSOstart; Gradient H2O +0.1TFA/MeOH +0.05 TFA
JG293_NewGrad
3: Diode Array
Range: 8.696e+1
215.9350
AU
9d
JG293_NewGrad
1: TOF MS ES+
BPI
5.49e4
907.7032
907.6962
%
Time

stock 1 ml DMSOstart; Gradient H2O +0.1TFA/MeOH +0.05 TFA

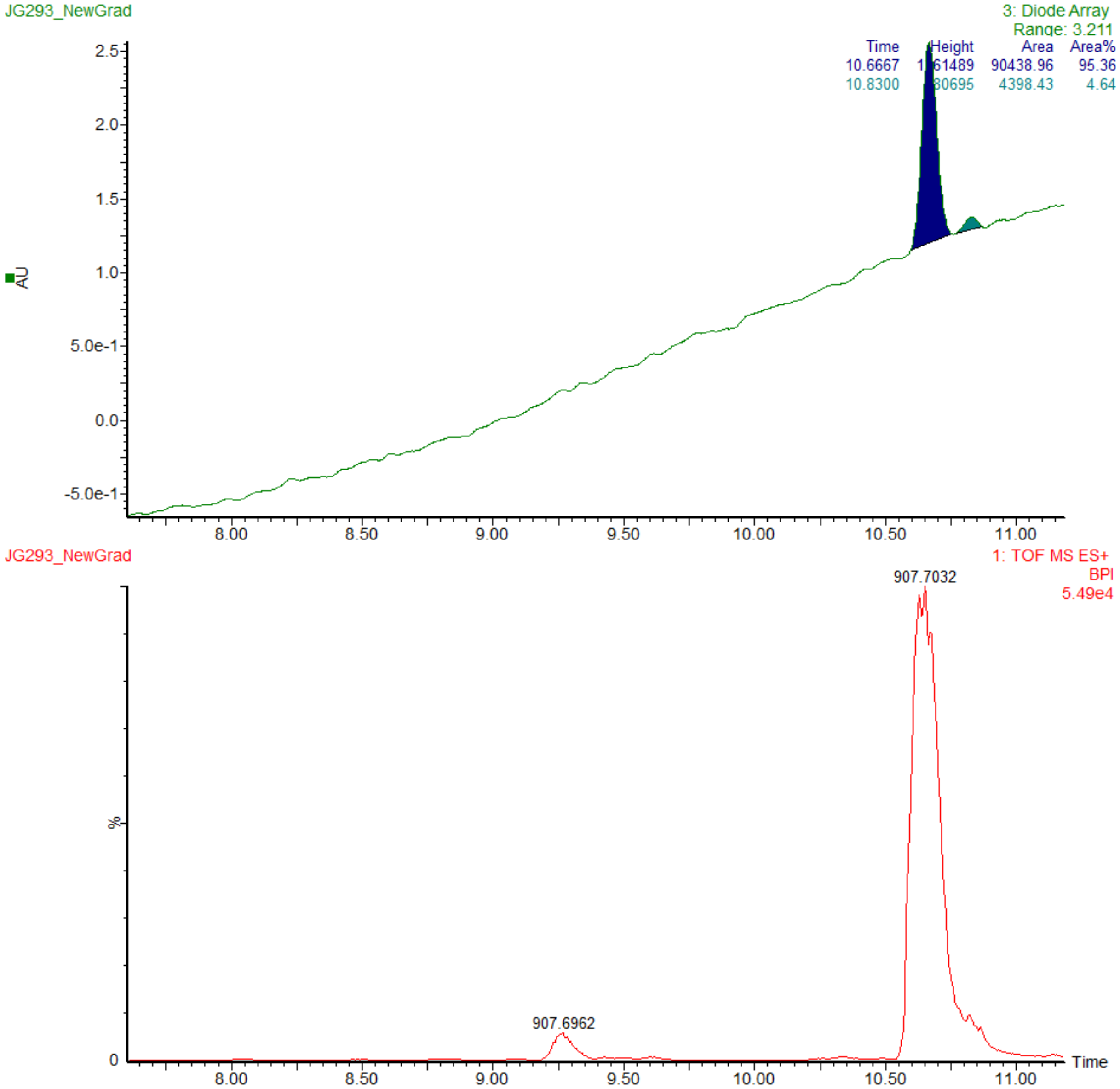

13. 9f

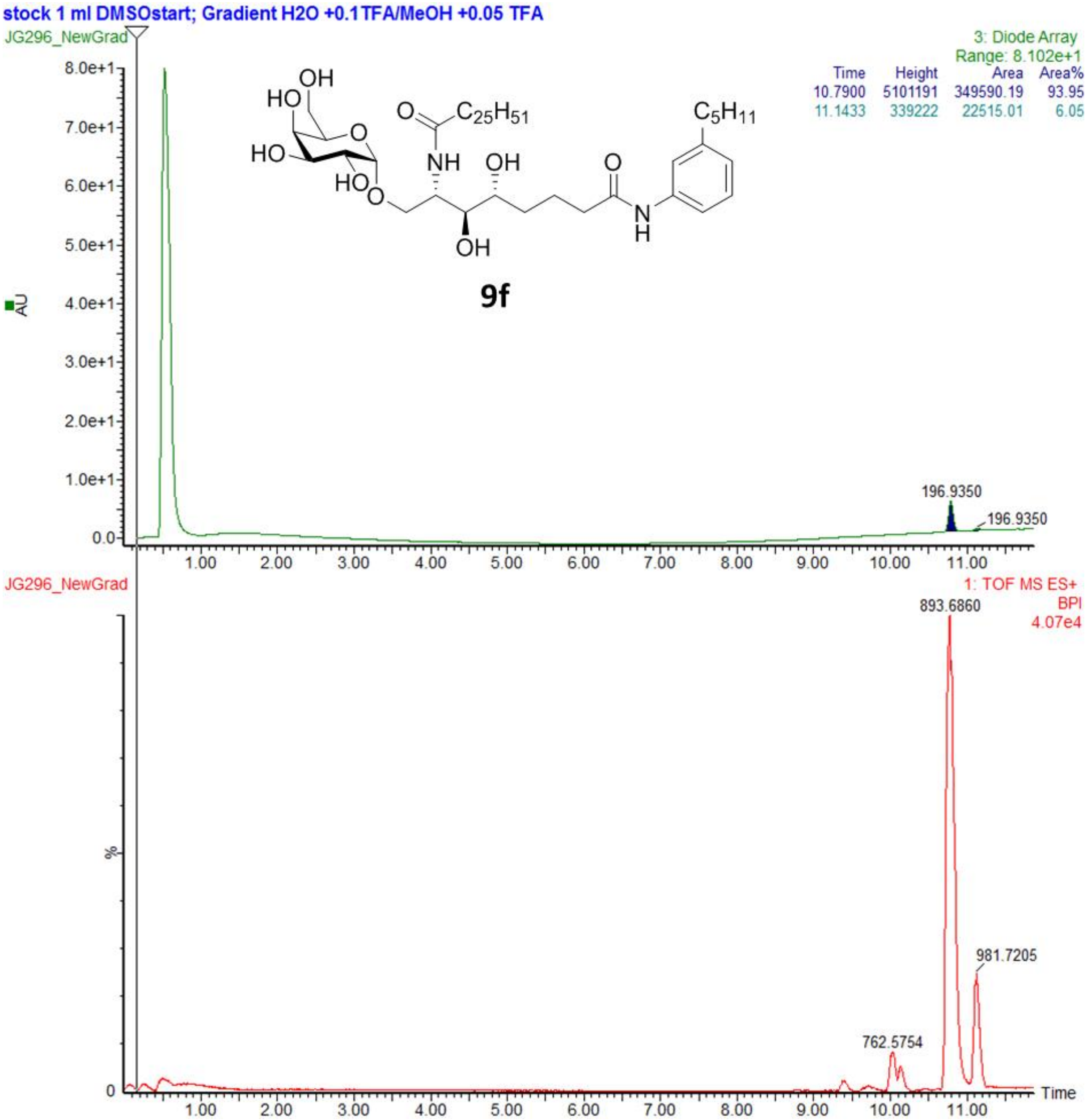

stock 1 ml DMSOstart; Gradient H2O +0.1TFA/MeOH +0.05 TFA
JG296_NewGrad
3: Diode Array
Range: 8.102e+1
Time Height Area Area%
10.7900 5101191 349590.19 93.95
11.1433 339222 22515.01 6.05
8.0e+1
7.0e+1
6.0e+1
5.0e+1
4.0e+1
3.0e+1
2.0e+1
1.0e+1
0.0
AU
196.9350
196.9350
1.00 2.00 3.00 4.00 5.00 6.00 7.00 8.00 9.00 10.00 11.00
9f
JG296_NewGrad
1: TOF MS ES+
BPI
4.07e4
893.6860
981.7205
762.5754
%
0
Time

stock 1 ml DMSOstart; Gradient H2O +0.1TFA/MeOH +0.05 TFA

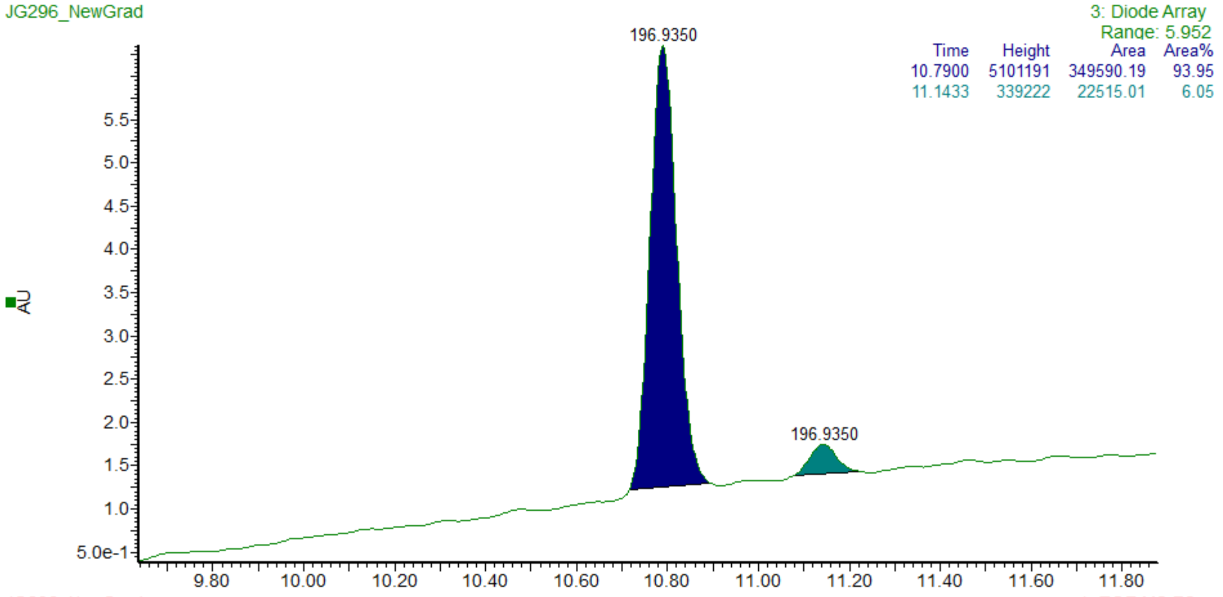

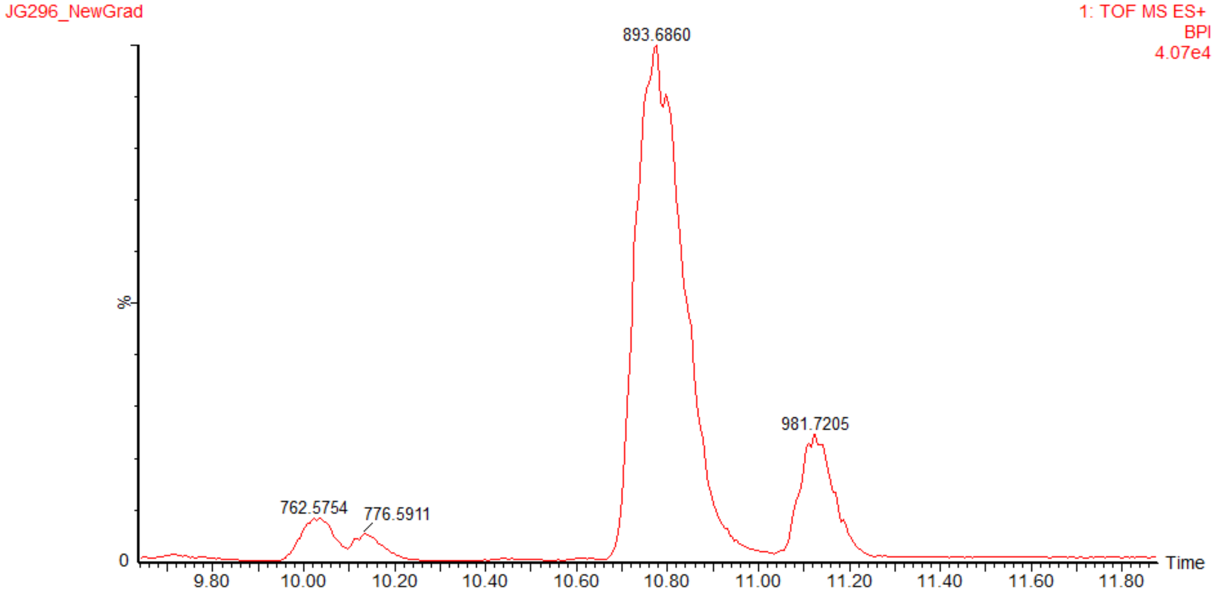

14. Blanc runs

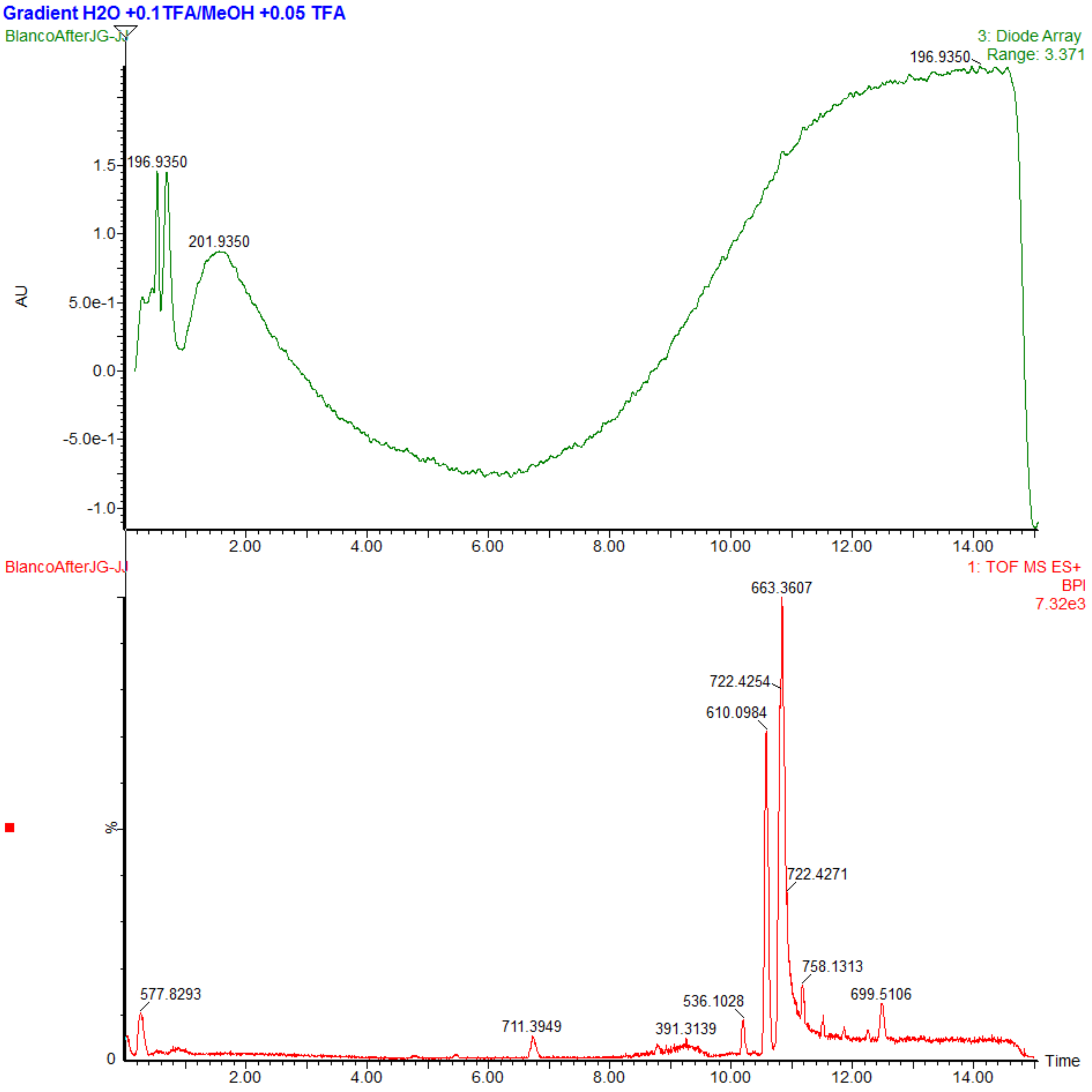

MeOH the same like for dilution of the samples

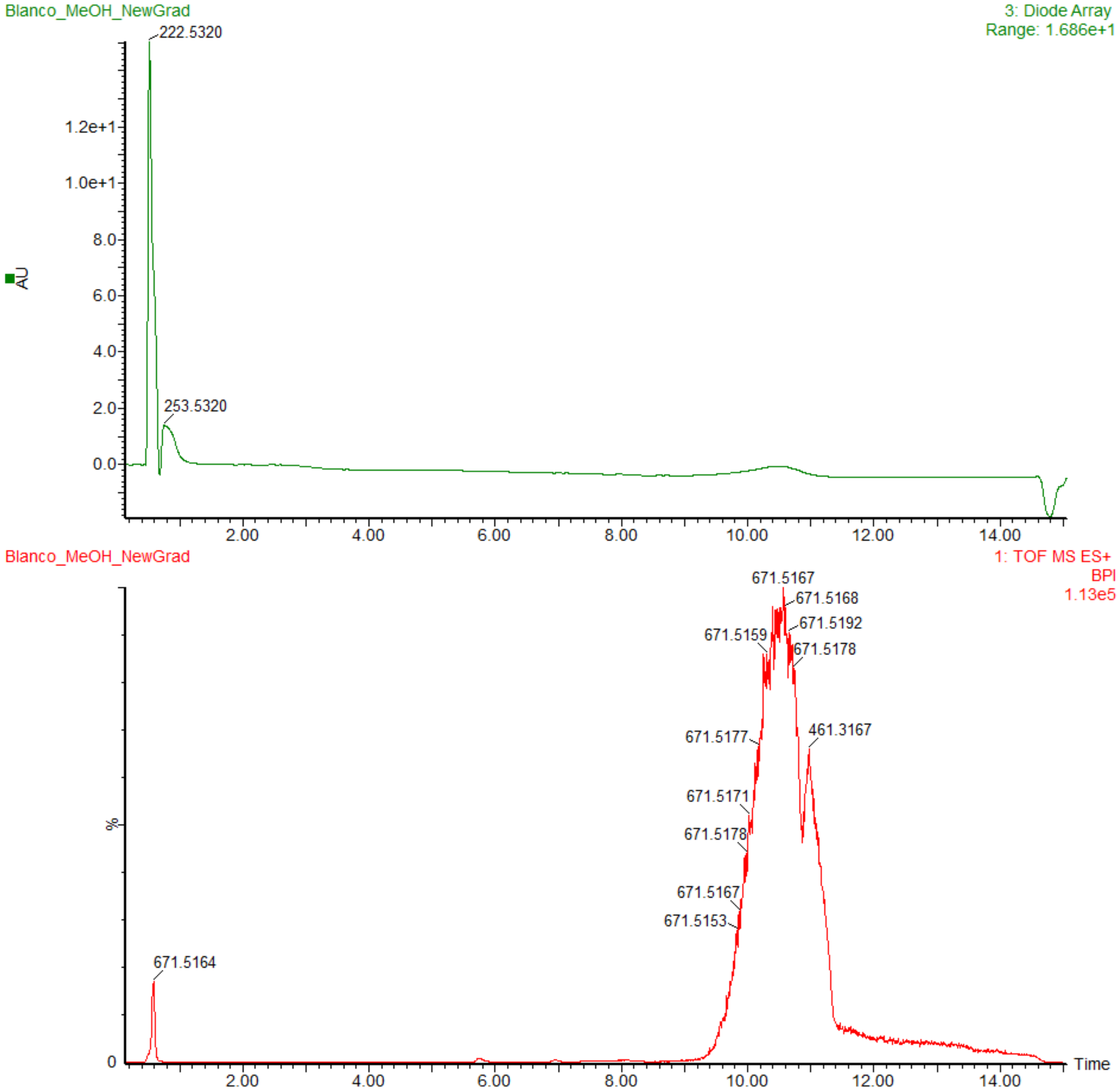

Supporting figure (S1)

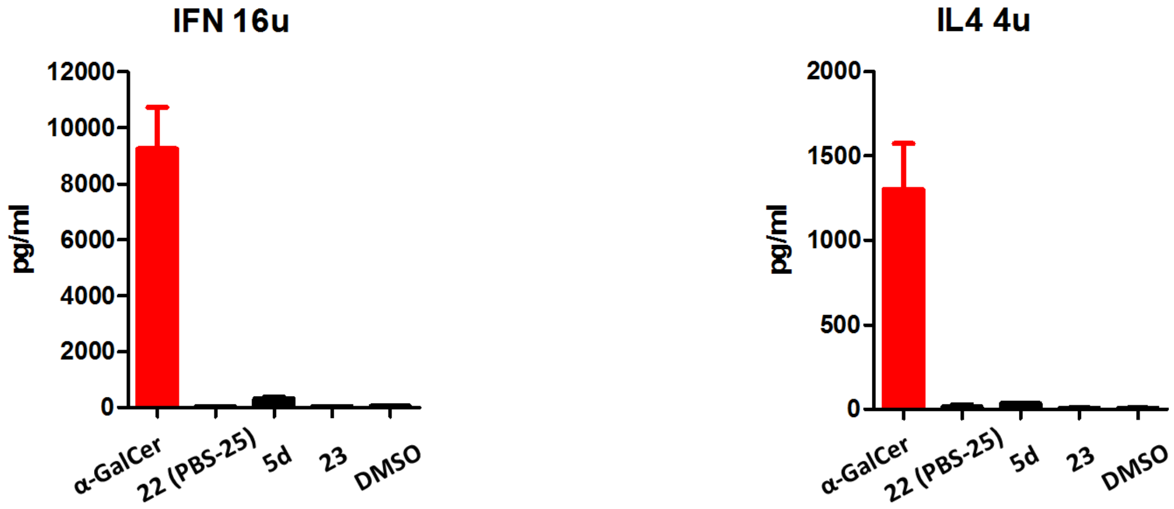

Figure S1: IFN-γ- and IL-4-secretion, measured at 16 h and 4 h respectively, after intraperitoneal injection of 5 µg of the glycolipids in mice. Data for one individual experiment using 8 mice for each glycolipid.
